# Gromomycins: An Unprecedented Class of Triterpene Antibiotics Produced by a Novel Biosynthetic Pathway

**DOI:** 10.1002/anie.202422270

**Published:** 2025-03-27

**Authors:** Stepan Tistechok, Dmytro Bratiichuk, Hilda Sucipto, Nils Gummerlich, Marc Stierhof, Oleksandr Gromyko, Franziska Fries, Victor Fedorenko, Rolf Müller, Josef Zapp, Maksym Myronovskyi, Andriy Luzhetskyy

**Affiliations:** ^1^ Department of Pharmaceutical Biotechnology Saarland University Bld. C2.3 66123 Saarbrücken Germany; ^2^ Department of Genetics and Biotechnology Microbial Culture Collection of Antibiotic Producers Ivan Franko National University of Lviv Hrushevskogo Street 4 Lviv 79005 Ukraine; ^3^ Helmholtz Institute for Pharmaceutical Research Saarland (HIPS) Helmholtz Centre for Infection Research (HZI) Saarland University 66123 Saarbrücken Germany; ^4^ German Center for Infection Research (DZIF) Partner Site Hannover‐Braunschweig 38124 Braunschweig Germany

**Keywords:** Antibiotics, Antimicrobials, Bioactivity, Biosynthesis, Terpenes

## Abstract

The current situation with drug‐resistant microbial pathogens is critical, dictating an acute need for novel efficient antibiotics. Herein, we report a new class of antibiotics named gromomycins with significant activity, especially against drug‐resistant Gram‐positive pathogens, including methicillin‐ and daptomycin‐resistant *Staphylococcus aureus*. Gromomycins are pentacyclic triterpenes with a cyclic guanidino group forming the fifth six‐membered ring. We have used transposon mutagenesis to identify the gromomycin biosynthetic gene cluster, since it could not be assigned by any available bioinformatics tools, highlighting its unique biosynthetic route. Using gene cluster engineering, feeding experiments, and LC‐MS and NMR analyses we have proposed the biosynthetic pathway for gromomycins, which are the first bacterial triterpenes synthesized independently of the squalene pathway. They also exhibit a so far unprecedented cyclization route that utilizes a hexaprenylguanidine linear precursor. Leveraging our understanding of their biosynthesis, we have identified additional gromomycin producers, resulting in the isolation of novel bioactive derivatives.

## Graphical abstract

Gromomycins represent a novel class of pentacyclic triterpene antibiotics with a unique biosynthetic pathway independent of the squalene route. These compounds exhibit potent activity against drug‐resistant Gram‐positive pathogens, including MRSA, and their unprecedented cyclization mechanism provides new insight into bacterial triterpene biosynthesis.

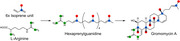

## Introduction

Natural products produced by living organisms, such as bacteria, fungi, plants, and marine organisms have been a valuable and fruitful reservoir for drug discovery, including antibiotics.^[^ ^1^, ^2^, ^3^, ^4^, ^5^ ^]^ Traditional antibiotics, like penicillin and tetracycline, have been derived from natural products and served as the foundation for many successful treatments.^[^ ^6^, ^7^, ^8^ ^]^ However, with the emergence of antibiotic resistance, there is a growing need for innovative antibiotics with novel chemical structures to combat multidrug‐resistant (MDR) pathogens.^[^ ^9^, ^10^ ^]^ Novel chemical scaffolds are more likely to target different bacterial vulnerabilities than conventional antibiotics, thereby overcoming existing resistance mechanisms and providing new options for treating infections that were previously difficult to manage.^[^ ^11^, ^12^ ^]^

Actinomycetes are a group of Gram‐positive bacteria that are known to produce a diverse array of bioactive compounds including antibiotics.^[^ ^13^, ^14^ ^]^ These bacteria are widely distributed in the soil and have been a rich source of lead structures for drug development, particularly in the field of antibiotics.^[^ ^15^, ^16^ ^]^ Many important antibiotics used in modern medicine, such as streptomycin, tetracycline, and erythromycin, were originally derived from actinomycetes.^[^ ^17^ ^]^ Recent advances in technology, such as genomics, transcriptomics, metabolomics, and synthetic biology, have enabled researchers to more efficiently screen for and identify new compounds.^[^ ^18^, ^19^, ^20^, ^21^ ^]^ These approaches have already led to the discovery of several new antibiotics such as teixobactin, which was discovered using a novel cultivation technique.^[^ ^22^ ^]^

While bioactivity screening of extracts from various sources is a standard approach in antibiotic discovery, focusing solely on bioactivity may lead to missed opportunities and limited success.^[^ ^23^ ^]^ The intricate composition of the extract can hinder the recognition of prospective antibiotic compounds by masking their activity within the complex mixtures.^[^ ^24^ ^]^ Looking into chemical diversity enables the exploration of a vast array of novel, potentially bioactive chemical structures that might otherwise be overlooked within the complex extract mixtures.^[^ ^25^ ^]^ In recent years, modern methods have shifted toward the heterologous expression of cryptic biosynthetic gene clusters (BGCs).^[^ ^26^ ^]^ This technique involves transferring BGCs from their native organisms into well‐characterized host organisms to produce new compounds. This approach holds significant promise, particularly for unlocking the potential of “silent” or “cryptic” BGCs that are not expressed under standard laboratory conditions.^[^ ^27^ ^]^

However, a significant challenge remains – identifying novel BGCs that have not been previously described. The current methods often rely on sequence homology to known BGCs,^[^ ^28^, ^29^ ^]^ which means that truly novel biosynthetic pathways might be overlooked if they do not resemble any known sequences. Consequently, this could lead to the inadvertent omission of unique natural products with potentially strong antibiotic properties. Herein, we describe the identification of a novel class of triterpenoid antibiotics, entitled gromomycins, by adopting a strategy focused on chemical diversity rather than solely on bioactivity, which has proven effective in discovering novel antibiotic structures. Structure elucidation via NMR revealed a novel chemical scaffold comprised of a pentacycle featuring a cyclic guanidino group. The only similar terpenoids, cybastacines, containing a guanidine group, have been isolated from cyanobacteria and belong to the sesterterpene family of natural products.^[^ ^30^ ^]^

Furthermore, we propose a so far unprecedented biosynthetic pathway that is independent of the squalene pathway^[^ ^31^ ^]^ and report the discovery of methylated gromomycin derivatives via a genome‐mining approach. Notably, we conducted initial activity and toxicity studies and found gromomycins to exhibit promising antibacterial activity against drug‐resistant pathogens.

## Results and Discussion

### Isolation, Identification, and Structure Elucidation of Gromomycins

The strain *Streptomyces sp*. Je 1–332, sourced from the rhizosphere soil of *Juniperus excelsa* M.‐Bieb.,^[^ ^32^ ^]^ yielded several distinct peaks in its crude extract (Figure S70). Mass spectral analysis of these peaks identified molecular ions [M + H^+^] with masses such as 496.39, 494.37, 494.42, 480.38, or 478.38 Da. Dereplication against the Dictionary of Natural Products (DNP)^[^ ^33^, ^34^ ^]^ database revealed no corresponding entries, suggesting these compounds are novel.

To elucidate their structures, strain 1–332 was cultivated in 10 liters of DNPM medium, and the metabolites were extracted from the culture supernatant using ethyl acetate. A total of nine compounds were isolated, yielding a couple of milligrams each, after a three‐step purification process. Compound **1** (496.39 Da) proved unstable, degrading to **2** (478.38 Da) during the purification process. This degradation, evidenced by an 18 Da mass difference, indicated the likely loss of H_2_O. We therefore initially focused on the structure of **2**. Its molecular formula was determined by HRESIMS (*m/z* 478.3804 ([M + H]^+^) as C_31_H_47_N_3_O with 10 degrees of unsaturation (DU). Its NMR data (Table S1) in CD_3_OD revealed seven methyls, eight methylenes, six methines, and ten quaternary carbons. Eight olefinic signals (DU = 4) appeared in the low field part of the ^13^C NMR together with a quaternary carbon at δ 152.53, which would fit very well with a guanidine unit (DU = 1). In addition, an alcohol function could be deduced from the signal at δ 79.78.

**Figure 1:**
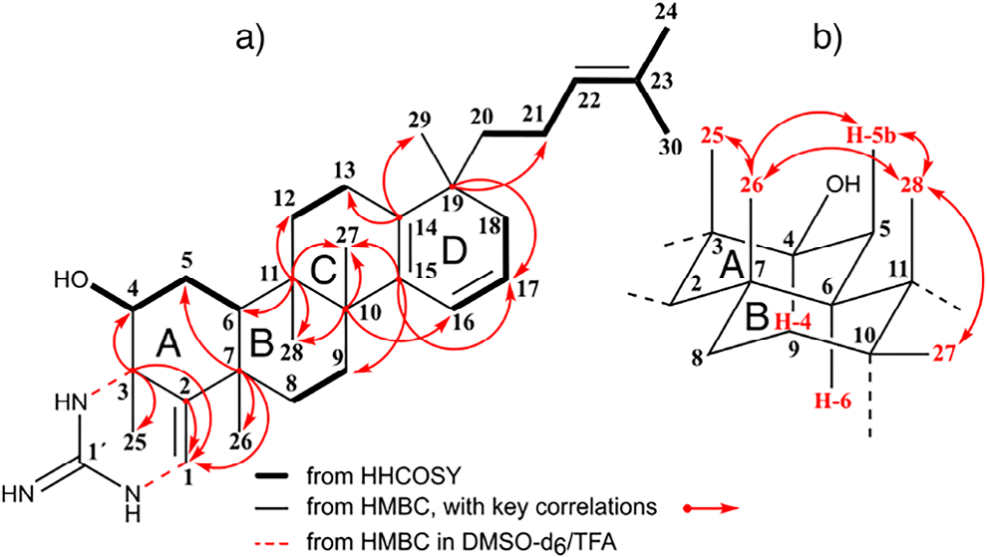
Structural features of Gromomycin A (**2**). a) Significant HHCOSY and HMBC correlations. b) Selected NOEs in rings A and B.

Based on these considerations, the remaining five degrees of unsaturation accounted for ring structures in the molecule. The ^1^H‐NMR showed seven singlet methyl resonances, two of them with a double bond shift (δ 1.54 and δ 1.64). Furthermore, four olefinic double bonds (δ 5.99, δ 5.85, δ 5.75, and δ 4.99) and a secondary alcohol (δ 3.66) were identified. HHCOSY correlations revealed a 2‐methyl‐pent‐2‐en‐5‐yl side chain together with several smaller C‐2 and C‐3 ring fragments, all located between quaternary carbons. After analyzing their correlations in the corresponding HMBC, these fragments could be linked with the quaternary carbons and the remaining five methyl groups to form four fused, six‐membered carbon rings A‐D, whose ring D bore the side chain and whose ring A should have fused with the remaining guanidine to form a 3,4‐dihydropyrimidin‐2(1*H*)‐imine ring (Figure 1a).

To learn more about the nature of this guanidine moiety, we repeated the NMR measurements in DMSO‐d_6_/TFA (Table S2). Its ^1^H‐NMR revealed signals for the NH protons at δ 9.15 (dd, *J = 5.0* and *2.0 *Hz, N_1_‐H), δ 7.92 (d, *J = 2.0* Hz, N_2_‐H) and δ 7.04 (s, 2H, N_3_‐H). For proton, N_1_‐H, a vicinal coupling with the olefinic hydrogen H‐1 was observed, which, in contrast to the spectrum in CD_3_OD, now appeared as a doublet (δ 5.86, *J = 5.0* Hz). ^1^H‐^13^C HMBC (C‐3/N_2_‐H, C‐2/N_1_‐H, C‐2/N_2_‐H) as well as ^1^H‐^15^ N HMBC correlations (N_1_/H‐1, N_2_/H‐25) proved the cyclic guanidine group. The relative stereochemistry of **2** was determined in CD_3_OD by analyzing the coupling patterns and evaluating the NOE effects of selected protons. In contrast to H‐4 (dd, *J = 12* and *4.5* Hz), the signals of H‐5a, H‐5b, and H‐6 were superimposed by other proton resonances. But with the help of a 1D selective TOCSY, they could be displayed separately and their coupling constants could be analyzed (Figure S7a).

Based on a vicinal coupling constant of 12 Hz for *J*_H‐4/H‐5b_ and *J*_H‐5b/H‐6_, an axial position could now be derived for each of the protons involved. The spatial orientations of the ring‐bound methyl groups were of particular interest for the relative configuration of **2**. Selective 1D NOESY spectra (Figure S7b) revealed NOE interactions between Me‐25, Me‐26, Me‐28, and H‐5b indicating that these protons are on the same side of the molecule in axial positions. This also meant that H‐6 and Me‐26, both in axial position, were located on different sides of the ring system, which required *trans*‐fused rings A and B (Figure 1b). NOE interactions between Me‐27 and Me‐28 suggested *cis*‐fused rings B and C. Only the stereochemistry at C‐19 could not yet be determined at this time. Neither the protons of the angular methyl group H‐29 and those from the side chain H‐21 – H‐24 nor those of rings C and D provided useful NOE effects here. Compound **2** (Figure 3), was named gromomycin A.

**Figure 2:**
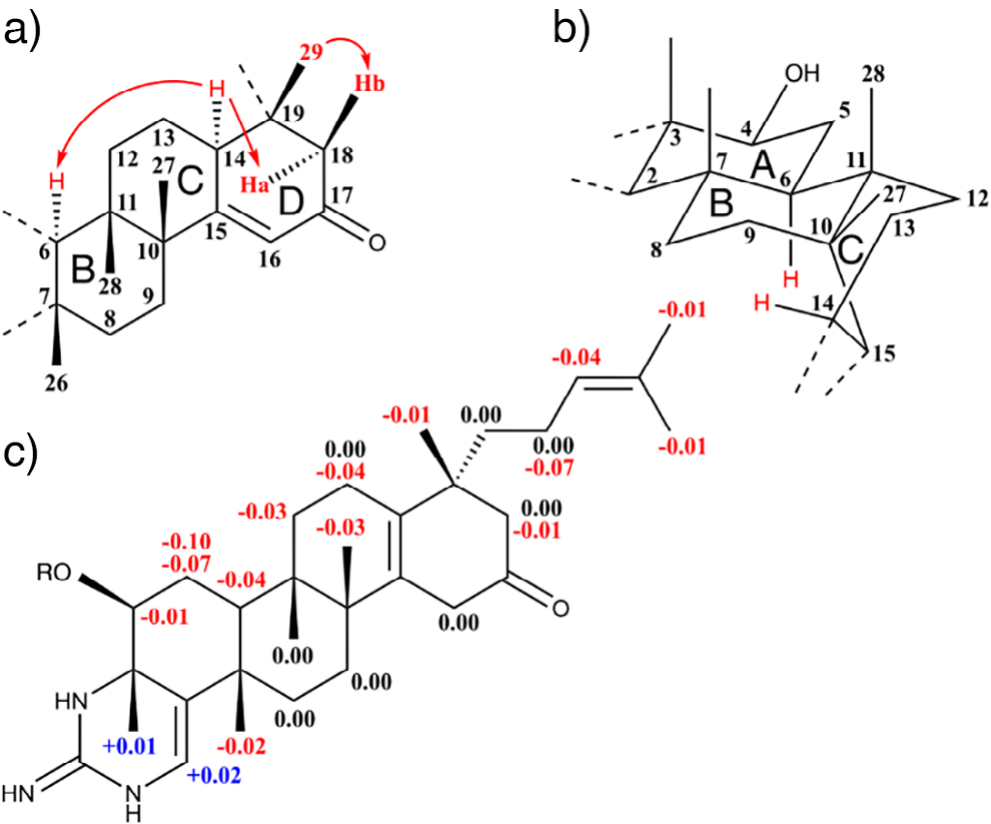
Structural features of Gromomycin B (**3**). a) Selected NOEs in rings B‐D. b) Conformations of ring A, B (both chair) and ring C (boat). c). ΔδS‐R values for MTPA derivatives derived from **3**.

The high‐resolution mass spectrum of gromomycin B (**3**) with *m/z* = 494.3752 for the ([M + H]^+^ ion peak provided an additional oxygen atom in the molecular formula, C_31_H_47_N_3_O_2_, when compared to **2**. A detailed examination of the NMR data (Table S3) revealed that all structural changes were restricted to ring D. According to the ^13^C NMR, the 14,16‐diene function had changed to a conjugated 15‐en‐17‐one. In the ^1^H‐NMR, the resonances at δ 6.15 for the double bond proton H‐16 and those of the doublets at δ 2.54 and 2.11 of the isolated methylene C‐18 (2.54 and 2.11) stood out. However, the proton resonance of the newly formed stereocenter methine C‐14 at δ 2.84 ppm proved to be particularly valuable, as it could be used to clarify the stereochemistry at C‐19. 1D selective NOESY spectra (Figure S20) were particularly helpful in this respect. Excitation of H‐14 showed an enhancement of H‐6 and H‐18a, which therefore must be on the same side of the molecule. Similarly, the NOE interactions between H‐18b and methyl‐H‐29 proved their localization on the side of the molecule facing away from H‐18a (Figure 2a).

The strong NOE between H‐6 and H‐14 was not easy to understand at first glance but could be explained with the help of a 3D model. If we assume that rings A and B are in chair conformation and ring C is in boat conformation both atoms are in close proximity to each other (Figure 2b). As the verification of the relative stereochemistry for the stereocenters already known from **2** did not lead to any changes, we were now able to determine the relative stereochemistry for the whole molecule.

In addition, a well‐founded proposal for the spatial position of Me‐29 in gromomycin A (2) could also be made.

The Mosher´s ester method was used to determine the absolute stereochemistry of C‐4 and by interference, the rest of the stereogenic centers in the entire molecule. Treatment of **3** with the (*R*)‐ and (*S*)‐MTPA chloride [α‐methoxy‐α‐trifluoromethylphenylacetyl chloride] in dry pyridine gave the (*S*)‐ and (*R*)‐MTPA esters in reasonable yields (see Experimental Section). This process also led to a migration of the double bond from C‐15/C‐16 to C‐14/C‐15 for both products, which was confirmed by a careful analysis of their ^1^H NMR, HHCOSY, and HSQC spectra (Figures S57–S62). The ^1^H NMR data of the Mosher ester derivatives (Table S11) led to the calculation of the Δδ_S‐R_ values (Figure 2c) and established the (*S*)‐configuration at C‐4, thus allowing the full absolute configuration of (3*S*,4*S*,6*S*,7*S*,10*R*,11*S*,14*S*,19*R*) to be assigned for gromomycin B (**3**).

Gromomycin C (**4**) displayed a ([M + H]^+^ ion peak at *m/z* 480.3953 in the HRESIMS corresponding to a molecular formula of C_31_H_49_NO. As already described for gromomycin B (**3**), a trisubstituted double bond between C‐15 (δ 143.48) and C‐16 (δ 120.27) could also be detected here. However, their resonances were shifted upfield and the signal for a keto group was completely missing in the ^13^C NMR (Table S4). It was therefore assumed that gromomycin C (**4**) is the 17‐deoxo derivative of **3**, which was confirmed by extensive 2D NMR measurements.

Gromomycin D (**5**) was obtained as a white powder. Its HRESIMS was identical to that of **3** and its NMR data (Table S5) proved that its structure was the same as that of the double bond isomer formed in the Mosher reaction of **3** with *R*‐ and *S*‐MTPA chloride.

**Figure 3:**
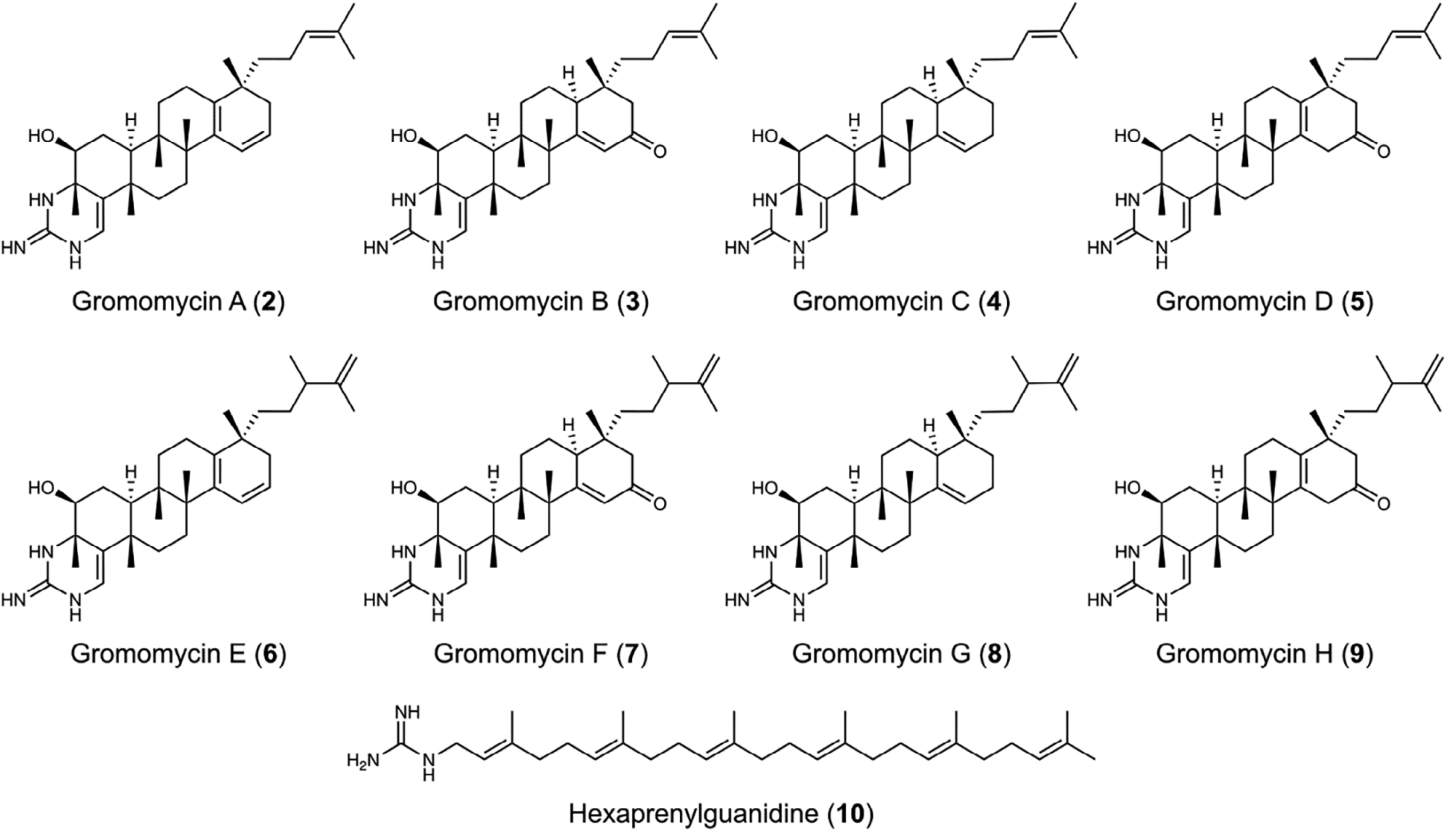
Structures of gromomycins A‐H and hexaprenylguanidine.

Gromomycin E (**6**, Figure 3) had a molecular formula of C_32_H_49_N_3_O as determined by HRESIMS (*m/z* 492.3950 [M + H]^+^). Its NMR data (Table S6) were very close to those of Gromomycin A (**2**), especially for the resonances of ring A‐D and the guanidine moiety. However, differences could be observed for the side chain. Here, the double bond has been moved from C‐22 to C‐23 at the end of the side chain and C‐22 now bore an additional methyl group C‐31. HHCOSY and HMBC correlations supported the 2,3‐dimethyl‐pent‐1‐en‐5‐yl side chain for gromomycin E.

HRESIMS data of **7–9** (see analytical data SI, p.12) showed that these compounds were also methylated gromomycins. Careful analysis of their NMR data (Tables S7–S9) revealed the same side chain for them as found for **6** and identical ring structures A‐D to those of **3–5**. However, due to the high flexibility of the side chain, the stereochemistry of the newly formed stereocenter at C‐22 could not be established.

The molecular formula of **10** was calculated as C_31_H_53_N_3_ with 7 degrees of unsaturation based on its ESIMS with *m/z* 468.4355 [M + H]^+^. The NMR data (Table S10) revealed an acyclic structure with six trisubstituted double bonds, bearing six protons (δ 5.06, 5H and 5.19, 1H), seven singlet methyls (δ 1.63, 5 Me and 1.55, 2 Me) and ten methylene groups (δ 1.93–2.02, 20H) similar to those of squalene. The resonance for a methylene group at δ 3.66 (2H) gave a hint of an additional polar substituent. Its composition, CH_4_N_3_, was deduced from the difference between parts of the ^1^H (δ 7.79, brs, 3H and 8.61, t, 1H) and ^13^C NMR the molecular formula of **10** and that of the triterpene unit, C_30_H_49_. The signals of this guanidyl moiety could be found in the lowfield (δ 157.29) spectra. 2D HHCOSY and HMBC proved the terminal position of the guanidyl residue in the polyprenyl chain but raised doubt about the squalene moiety. In contrast to a hypothetical 1‐guanidylsqualene, the methylene protons H‐1 showed vicinal correlations with the NH proton at δ 8.61 and the proton at δ 5.19 of the neighboured double bond proton.

Therefore, the terpene moiety was formed by six head‐tail‐linked isoprene units leading to the structure of 1‐((2*E*,6*E*,10*E*,14*E*,18*E*)‐3,7,11,15,19,23‐hexamethyltetracosa2,6,10,14,18,22‐hexaen‐1‐yl) guanidine that we named hexaprenylguanidine (Figure 3).

### Identification of the Gromomycin Biosynthetic Gene Cluster

To identify the gromomycin biosynthetic gene cluster (BGC) within *Streptomyces sp*. Je 1–332, we sequenced its genome and conducted an analysis using antiSMASH 6.0.^[^ ^29^ ^]^ The analysis via antiSMASH revealed 21 potential BGCs. Considering the terpene‐like core of gromomycins we have focused on five BGCs for terpene synthesis. Notably, two of these (antiSMASH Regions 14 and 15 (Table S17)) displayed low homology to known terpenes, suggesting they might be involved in gromomycin production.

**Figure 4:**
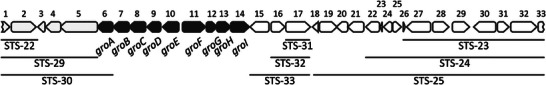
Diagram of the DNA segment containing the gromomycin biosynthetic gene cluster, depicted in black. The lines indicate the individual deletions that were made.

To investigate this hypothesis, we employed homologous recombination to replace the terpene cyclase genes in these regions with apramycin resistance cassettes. HPLC‐MS analysis of the resulting strains did not show any changes in gromomycin production, indicating these clusters were not responsible for its biosynthesis. Therefore, we have adopted the Tn5 and Himar1 transposon mutagenesis as our subsequent approach to uncovering the elusive gromomycin BGC (Figure S74). Over 1500 *Streptomyces sp*. Je 1–332 transposon mutants were screened for their ability to produce gromomycin by HPLC‐MS. Within this pool of transposon mutants, we found five mutants lacking gromomycin production (Figure S69b) and identified their transposon insertion sites. In the two mutants, Tn5_mut_148 and Tn5_mut_355, the insertions were found close to and surrounded by a gene that encodes for a putative farnesyl diphosphate synthase (*fdps*) (Figure S69a), which is identified as a *groD* gene in Figure 4.

The *fdps* gene was replaced by an apramycin resistance marker within the chromosome of *Streptomyces sp*. Je 1–332 to investigate its role in gromomycin biosynthesis. HPLC‐MS analysis indicated that the deletion of the *fdps (groD)* gene ceased gromomycin production. Consequently, we conducted additional deletions to confirm that this region harbors the gromomycin BGC. These deletions encompassed a gene annotated as a putative enduracidin beta‐hydroxylase (*groE*), a hypothetical protein (*groF*), a PAP2 superfamily protein (*groG*), another hypothetical protein (*groH*), and a cytochrome P450 (*groI*), and two genes annotated as putative aminopyrrolnitrin oxygenases (*groB* and *groC*). All resulting deletion mutants either failed to produce gromomycin or produced its derivatives (Figure S71). This led us to hypothesize the presence of a gromomycin BGC within the identified genome region (5778465 – 5804255 bp). The corresponding region carrying gromomycin biosynthetic genes has been cloned into a cosmid pHSU‐STS10 and transferred into the heterologous host strains *S. albus* Del14^[^ ^35^ ^]^ and *S. lividans* Del8^[^ ^36^ ^]^ via conjugation. We analyzed the metabolic profile of the resultant strains, *S. albus* STS10 and *S. lividans* STS10, using HPLC‐MS. Gromomycin production was detected in extracts from both heterologous host strains (Figure S65), confirming the identification of a gromomycin BGC through random transposon mutagenesis followed by HPLC MS screening of the corresponding mutant library.

The vector pHSU‐STS10, used for heterologous expression in *S. albus* Del14 and *S. lividans* Del8, harbors a 35.6 kb chromosomal fragment from *Streptomyces sp*. Je 1–332. This particular fragment, not recognized as a putative biosynthetic gene cluster (BGC) by the antiSMASH program, encompasses 33 open reading frames (ORFs) (Figure 4). To identify the minimal set of genes essential for gromomycin biosynthesis, we executed a series of gene deletion experiments. The genes previously deleted in the native gromomycin producer strain *Streptomyces sp*. Je 1–332, which demonstrated a significant impact on gromomycin production, were located at positions 6 to 14 and served as the focal point for systematic deletions. To ascertain all the specific genes essential for gromomycin biosynthesis, we initiated six deletions—STS22, STS29, and STS30 on the left arm, and STS23, STS24, and STS25 on the right arm (Figure 4). These deletions were generated using the *bla* marker to replace the desired fragment through RedET recombination. Subsequently, the *bla* marker was excised using the PmeI restriction enzyme to avoid a possible polar effect. The deletions STS22, STS23, STS24, and STS25 did not affect gromomycin production, indicating that genes 1, 2, and 18–33 are dispensable for its synthesis. However, the STS29 deletion resulted in a marked reduction in gromomycin levels, whereas the STS30 deletion completely halted production (Figure S72). The difference between STS29 and STS30 is the absence of gene 6 in the latter, suggesting that gene 6 encodes an enzyme crucial for gromomycin synthesis.

To pinpoint the genes that suppress gromomycin production in the STS29 deletion strain, we deleted genes 3–5 individually and assessed the effects. Deletions of genes 3 and 4 were associated with a substantial decrease in gromomycin levels, while deletion of gene 5 had no discernible impact, suggesting regulatory roles for genes 3 and 4. Additionally, to delineate the boundary of the gene cluster downstream of genes 6–14, we performed three more deletions—STS31, STS32, and STS33. No variation in gromomycin production was observed among *S. albus* STS31, STS32, and STS33 strains (Figure S72). Hence, we deduced that the minimal set of genes responsible for gromomycin synthesis encompasses genes 3 to 14. Within this range, it is probable that genes 6 to 14 are structural, designated *groA* to *groI*, while genes 3 to 5 likely play a regulatory role.

### Establishing Pyruvate and L‐ariginine as Gromomycin Biosynthetic Precursors

To uncover the precursors involved in gromomycin biosynthesis, we conducted a series of feeding experiments with [2–^13^C]‐ and [3–^13^C]‐labeled sodium pyruvate. We utilized the heterologous host strain *S. albus* STS10 to produce gromomycin. As the *S. albus* Del14 strain is devoid of the mevalonate pathway, we anticipated that the synthesis of isoprene units would occur through the non‐mevalonate (MEP) pathway. Following the feeding with [2–^13^C]‐sodium pyruvate, we isolated 0.9 mg of labeled gromomycin, and from the [3–^13^C]‐sodium pyruvate feeding, we isolated 1.2 mg. We employed ^13^C NMR spectroscopy to pinpoint the positions of the incorporated labeled carbons (Tables S12 and S13; Figures S63 and S64). The experiments revealed that each feeding resulted in the incorporation of six ^13^C atoms into the gromomycin molecule, confirming the use of these labeled precursors in its biosynthesis and establishing the art of incorporation.

Furthermore, to clarify the origin of the intriguing guanidine moiety in the structure of gromomycin, we conducted feeding experiments using variously labeled arginines. Specifically, we utilized arginine labeled on all carbon and nitrogen atoms, as well as arginine labeled solely on nitrogen atoms. When arginine labeled on both carbons and nitrogens was fed, a uniform mass increase of + 4 was detected across all identified peaks. Conversely, when we fed the strain arginine labeled only on nitrogen, we observed a mass increase of + 3 in the peaks. These observations lead us to propose that the guanidine moiety of gromomycin is directly derived from arginine (Figure S66).

### Proposed Biosynthetic Pathway of Gromomycin

The postulated minimal gene cluster for gromomycin synthesis comprises 12 open reading frames, spanning from *gro3* to *groI*. We conducted several gene inactivation experiments to elucidate their roles in gromomycin biosynthesis. BLAST analysis did not offer a clear function for these genes, except for *groD* and *groI* encoding a polyprenylsynthase and CYP450 monooxygenase, respectively (Table 1).

**Table 1:** The proposed function of genes in gromomycin gene cluster.

Gene	Proposed function
*groA*	Rieske 2Fe‐2S domain‐containing protein *S. azureus*
*groB*	Rieske 2Fe‐2S domain‐containing protein *S. azureus*
*groC*	Rieske 2Fe‐2S domain‐containing protein *S. azureus*
*groD*	Polyprenyl synthetase (Farnesyl diphosphate synthase)
*groE*	Reductase
*groF*	Hypothetical protein (cyclase)
*groG*	Phosphatase PAP2
*groH*	Hypothetical protein (prenyl transferase)
*groI*	Cytochrome P450 107B1 (Monooxygenase/Oxidoreductase)

Genes *groA* to *groC* and *groF* to groI were deleted in‐frame to prevent any polar effects, and their integrity was confirmed through construct sequencing. Gromomycin production in these modified strains was assessed using HPLC‐MS and produced derivatives and precursors have been purified and their structures elucidated with NMR. The deletion of the *groD* gene has been shown to completely abolish gromomycin synthesis. The GroD enzyme shows strong similarity to the class I terpene synthases having two conserved DDxD and DTE motifs for binding of Mg^2+^ ions that in turn bind the substrate's diphosphate. These enzymes synthesize various chain length (C10, C15, C20, C25, C30, C35, C40, C45, and C50) linear isoprenyl diphosphates from precursors, isopentenyl diphosphate (IPP) and dimethylallyl diphosphate (DMAPP). We suggest GroD combines six prenyl groups, forming a hexyprenyl diphosphate, a crucial intermediate in gromomycin biosynthesis.

**Figure 5:**
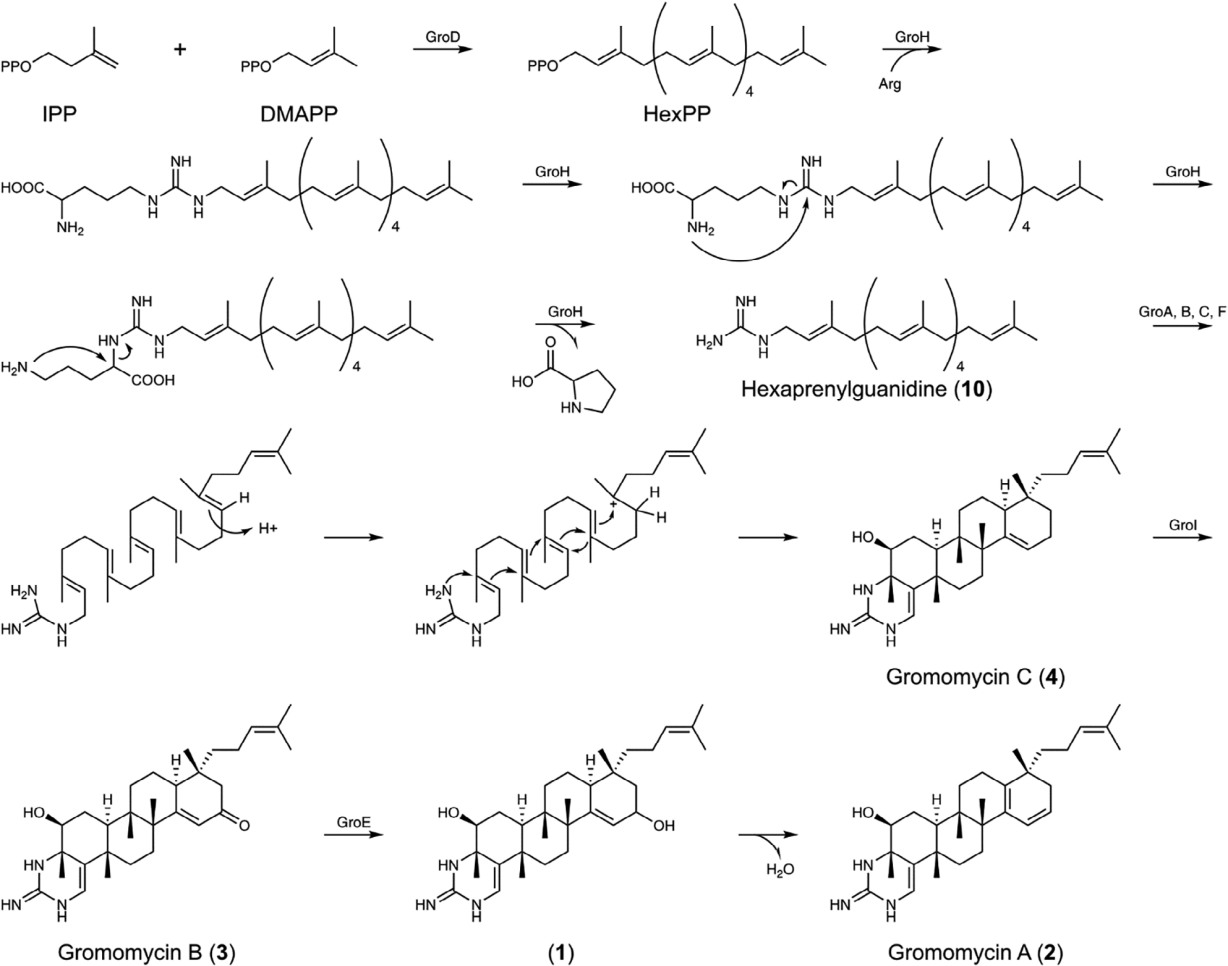
Proposed biosynthetic pathways of gromomycins. IPP, isopentenyl diphosphate; DMAPP, dimethylallyl diphosphate; HexPP, hexaprenyl diphosphate, Arg, L‐arginine.

The deletion of the *groH* gene completely abolished the gromomycin production, suggesting its involvement in the early biosynthetic stages. In‐depth analysis of the GroH protein, utilizing Swiss‐Prot for structure homology modeling (https://swissmodel.expasy.org), revealed its resemblance to several terpene synthases and a notable similarity to prenyltransferases. We propose that GroH transfers the hexaprenyl moiety to arginine, followed by the release of proline through the nucleophilic attack of the alpha‐amino group on the imino carbon of the guanidine moiety forming hexaprenylguanidine (Figure 5).

Inactivating the *groF* gene, identified as a hypothetical protein, led to a derivative with a mass of [M + H^+^] 468.38 Da. Following purification and structural analysis (Table S10 and Figures S51–S56), this compound was identified as hexaprenylguanidine (**10**), a linear precursor to gromomycins (Figure 3). It is characterized by a unique C1‐C4′ bond between two farnesyl groups, a feature unprecedented in bacterial triterpenes.

Advanced analysis of the GroF protein, using Swiss‐Prot for structure homology modeling, showed that it contains a terpene cyclase domain, similar to the TvTS cyclase from *Talaromyces verruculosus* ^[^ ^37^, ^38^ ^]^ and the FlvF cyclase‐like protein from *Aspergillus flavus*.^[^ ^39^ ^]^ Coupled with the isolation of the linear hexaprenylguanidine precursor, these findings suggest that GroF likely serves as a gromomycin cyclase. The protonation of C‐19 in hexaprenylguanidine by GroF cyclase likely follows a mechanism similar to that of squalene‐hopene cyclase (SHC) in bacteria.

In SHC, an aspartate‐rich DXDDTA motif initiates the polycyclization reaction by facilitating proton donation from aspartate.^[^ ^40^ ^]^ In GroF, we have identified a DLADPD motif, which is highly enriched in aspartates and may fulfill a comparable catalytic function. Upon protonation, the polycyclization reaction progresses through a sequence of rigidly held carbocation intermediates. Finally, the highly nucleophilic amino group of the guanidine moiety may attack the last C‐3 carbocation in the cascade, resulting in the formation of the cycloguanidine ring.

Inactivating the *groA, groB*, and *groC* genes, which are annotated as putative Rieske non‐heme iron oxygenases, also resulted in the accumulation of hexaprenylguanidine, a linear precursor to gromomycin. This suggests their involvement in the cyclization process of gromomycins. The C4‐OH group is most likely introduced by one of the Rieske oxygenases (GroA, GroB, or GroC), as these enzymes are known to catalyze hydroxylation reactions. Similar mechanisms have been described for the Rieske‐type oxygenases KshAB in *Mycobacterium tuberculosis*, which play a key role in cholesterol catabolism.^[^ ^41^ ^]^

Additionally, the formation of C1‐C2 and C15‐C16 double bonds may also be catalyzed by one of the three Rieske oxygenases, as these enzymes are known to introduce double bonds. A well‐documented example is the Rieske enzyme DAF‐36, which catalyzes the conversion of cholesterol to 7,8‐dehydrocholesterol, potentially via a monohydroxylated intermediate that subsequently undergoes dehydration.^[^ ^42^ ^]^

The inactivation of the *groI* gene led to the synthesis of a compound, possessing a mass of [M + H^+^] 480.44 Da. This substance was subsequently isolated, and its structure, confirmed through NMR spectroscopy appears to be gromomycin C (**4**) (Figure 3; Table S4 and Figures S21–S26). Given the absence of a hydroxy group in the fifth ring of gromomycin C (**4**) and the high similarity of the *groI* gene to genes encoding CYP450‐dependent oxygenases, we propose that the associated enzyme is responsible for incorporating a keto group at C‐17 of gromomycins, leading to the formation of gromomycin B (**3**). The groE gene mutants accumulated gromomycin B (**3**) suggesting that the *groE* gene product reduces the keto group to a hydroxyl group at position C‐17 leading to the hydroxylated gromomycin **1**, which we could not isolate due to its instability. This compound degrades to gromomycin A (**2**) during the purification process (Figure 5).

### Genome‐guided Isolation of New Gromomycins

After delineating the biosynthetic pathway of gromomycin, we adopted a genome mining approach to understand how widely is the novel gromomycins‐like BGCs distributed and to identify new derivatives. This method hinges on the concept that genes dictating the core chemical structure of gromomycin, in our instance *groD, groH*, and *groF*—serve as “hooks” for genome mining, guiding the search for related analogs. By utilizing this technique, we can expand the structural series of compounds, enhancing our understanding and potential optimization of the structure‐activity relationships. Prior research has shown that deleting these genes is critical for gromomycin production. We utilized the nucleotide sequences of these genes as probes in the NCBI protein BLAST database to identify new gromomycin‐like clusters. Our objective was to find clusters harboring these three genes in close proximity within the genome.

This search revealed seven *Streptomyces* and three rare actinobacteria strains — *Actinoplanes xinjiangensis*, *Saccharopolyspora phatthalungensis*, and *Frankia casuarinae* with all three genes situated closely together in their genomes. Further examination showed that three strains (*S. azureus, S. termitum*, and *S. albulus*) possess gromomycin‐like clusters nearly identical to the original. Meanwhile, two strains (*S. tendae* and *S. parvulus*) include an extra gene encoding a protein with a methyltransferase domain. Additionally, *S. pilosus* and *S. flavoviridis* feature gromomycin‐like clusters with a type II methyltransferase, absent in the original gromomycin cluster.

We constructed a cosmid library for the *S. flavoviridis* JCM 4372 strain and identified a gromomycin‐like biosynthetic gene cluster (BGC) on cosmid P03_G02 through PCR. This BGC was then introduced into the heterologous host strains *S. lividans* Del8 and *S. albus* Del14. High‐resolution HPLC‐MS analysis of both *S. albus* Del14 and *S. lividans* Del8 strains, containing the P03_G02 cosmid, revealed six distinct peaks (Figure S67). The mass spectra analysis showed molecular ions [M + H^+^] with masses of 458.37, 482.45, 492.39, 494.41, 496.43, and 508.39 Da. To determine the structure of the identified compounds, strain *S. albus* Del14 with *S. flavoviridis* gromomycin‐like cluster was grown in 10 L of DNPM medium and metabolites were extracted from the supernatant with ethyl acetate.

New gromomycins corresponding to the identified molecular ions [M + H^+^] with the masses 492.39 Da (gromomycin E (**6**)), 494.41 Da (gromomycin G (**7**)) and 508.39 Da (gromomycins F (**8**) and H (**9**)) were successfully purified from the extract and their structures have been determined by NMR (Tables S6–S9 and Figures S36–S50). Their structures and names, along with the position of the methyl group at C‐22, are shown in Figure 3.

Noteworthy, careful analysis of the extracts of *S. albus* Del14 with gromomycin‐like cluster from *S. flavoviridis* led to the identification of a linear methylated hexaprenylguanidine with a mass of 482.45 Da suggesting that methylation occurs prior to cyclization (Figure S68).

### Bioactivity Profiling of Gromomycins

In an effort to study the biological activity of gromomycins we screened derivatives gromomycins A and B against a preliminary panel of bacteria and fungi and found that they possess antibacterial activity in particular against Gram‐positive species with minimum inhibitory concentrations (MICs) in the low one‐digit µg mL^−1^ range. Consequently, we assessed the activities of gromomycins including the new methylated derivatives gromomycins F and G using a broad panel of high‐priority (according to the WHO priority list^[^ ^43^ ^]^ and ESKAPE (*Enterococcus faecium, Staphylococcus aureus, Klebsiella pneumoniae, Acinetobacter baumannii, Pseudomonas aeruginosa, Enterobacter* spp.)) pathogens (Table 2). All tested compounds except for gromomycin G showed remarkable activity against Gram‐positive bacteria. Gromomycins were not only active on the methicillin‐susceptible *S. aureus* strains Newman and Cowan 1, but also showed very promising activity against methicillin‐resistant *S. aureus* N315, the vancomycin‐intermediate (VISA) strain Mu50 and a laboratory daptomycin‐resistant *S. aureus*, indicating an absence of cross‐resistance to first‐line agents for MRSA treatment.^[^ ^44^ ^]^ Furthermore, gromomycins were active on penicillin‐resistant *Streptococcus pneumoniae* and exhibited substantial activity on *M. tuberculosis* and *A. baumannii*. Interestingly, gromomycin A shows the most potent activity against *A. baumannii*, while its methylated side‐chain variant, gromomycin E, completely loses activity. In general, the presence of a methyl group in the side chain of gromomycins appears to reduce activity in most cases. The remaining panel of Gram‐negative pathogens revealed only moderate activity against species from the Enterobacteriaceae.

**Table 2:** Antimicrobial activity spectrum of gromomycin derivatives.

		MIC [µg mL^−1^]						
Classification	Organism	GromA	GromB	GromE	GromF	GromG	GromH	REF^d)^
Gram‐positive	*S. aureus* ATCC29213	2	4	4	4	> 64	4	VAN: 2
	*S. aureus* Newman	2	4	4	4	16	4	VAN: 2
	*S. aureus* Cowan 1	2	4	4	4	> 64	8	VAN: 2
	*S. aureus* N315	2	4	4	4	> 64	8	VAN: 2, AMP: > 64
	*S. aureus* Mu50	2	16	4	8	16	8	VAN: 8, AMP: > 64
	*S. aureus* HG001 WT	2	4	4	4	> 64	4	DAP: 1
	*S. aureus* HG001 ^Dap[R]^	2	4	4	4	16	4	DAP: 64
	*S. pneumoniae* DSM11865	2	4	4	4	16	Nd^b)^	RIF: 0.03
	*E. faecalis* ATCC29212	2	8	4	8	16	8	RIF: 0.5
	*B. subtilis* DSM10	2	4	4	4	16	4	VAN: 1
Mycobacteria	*M. smegmatis* mc^2^155	16	8	16	16	64	Nd^b)^	RIF: 32
	*M. tuberculosis* H37Ra	8	16	8	16	32	8	RIF: 0.02
Gram‐negative	*E. coli* BW25113	> 64	32	> 64	64	> 64	64	CIP: 0.03
	*E. coli* K12 D*tolC* ^a)^	64	8	> 64	16	> 64	16	CIP: 0.01
	*E. coli* K12 D*tolC* ^a)^ + PMBN^c)^	2	4	8	4	> 64	Nd^b)^	CIP: 0.01
	*E. coli* WO153	2	8	8	4	> 64	Nd^b)^	CIP: 0.01
	*S. enterica* DSM5569	> 64	32	> 64	64	> 64	64	CIP: 0.02
	*C. freundii* DSM30039	> 64	32	> 64	64	> 64	64	CIP: 0.02
	*K. pneumoniae* DSM681	32	8	> 64	16	> 64	16	CIP: 0.01
	*A. baumannii* DSM30007	8	32	> 64	16	> 64	64	CIP: 2
	*A. baumannii* DSM30008	8	32	> 64	16	> 64	64	CIP: 0.5
	*A. baumannii* NCTC13301	16	32	> 64	32	> 64	64	CIP: > 64, COL: 1
	*P. aeruginosa* PA14	> 64	> 64	> 64	> 64	> 64	> 64	CIP: 0.25

^a)^ Keio collection mutant; efflux‐deficient.

^b)^ Not determined (nd) due to limited compound availability.

^c)^ 3 µg mL^−1^ polymyxin B nonapeptide (PMBN).

^d)^ Reference antibiotics: AMP, ampicillin; CIP, ciprofloxacin; COL, colistin; DAP, daptomycin; RIF, rifampicin; VAN, vancomycin. [R] Daptomycin resistance.

Notably, gromomycins display higher activity against *E. coli* WO153, which lacks the outer membrane porin TolC of the AcrAB‐TolC tripartite efflux pump, and has an impaired penetration barrier due to decreased amounts of lipopolysaccharide (LPS) in its outer membrane.^[^ ^45^ ^]^ Besides, we observed enhanced activities in the presence of sub‐inhibitory concentrations of the outer membrane permeabilizing agent polymyxin B nonapeptide (PMBN); thus, indicating that limited activity against Gram‐negative bacteria is mostly due to impaired penetration into the bacterial cell and to some extent also due to efflux, rather than the absence of the target. Interestingly, the activity against E. coli ΔtolC and K. pneumoniae strongly correlates with the presence of a keto group at position C‐17, with Gromomycin B, F, and H being the most active. Triterpenes often pose the risk of cytotoxicity. Thus, we performed thorough safety profiling and found that gromomycin derivatives gromomycins A, E, and H exert cytotoxic effects in vitro on HepG2 (IC50 2.2 to 19.4 µg mL^−1^) and CHO‐K1 cells (IC50 4.1 to 23.9 µg mL^−1^), whereas other derivatives showed no toxic effects up to concentrations of 37 µg mL^−1^. By contrast, we observed toxicity for every tested derivative in the highly sensitive zebrafish embryo model (Table S18 and Figure S73), which is more predictive of toxic effects as compared to standard cell culture systems.

Strikingly, the maximum tolerated concentrations (MTCs) determined in zebrafish embryos correlate with the antibacterial activity, hinting toward a target that is universally conserved amongst prokaryotes and eukaryotes. Considering the high toxicity correlated with their activity, gromomycins are unlikely to be further developed as antibiotics. However, they could be valuable tools for studying bacterial resistance mechanisms, understanding biosynthetic pathways, and mechanisms, or serving as lead structures for the development of modified derivatives with improved therapeutic potential.

## Conclusion

The discovery of gromomycins marks a significant advancement in the fight against antibiotic‐resistant bacteria. Our newly identified natural compounds, derived from *Streptomyces* bacteria, offer potent activity against some of the most challenging pathogens, including methicillin‐resistant *Staphylococcus aureus* (MRSA) and *Mycobacterium tuberculosis*, the bacteria responsible for tuberculosis. What makes gromomycins particularly exciting is their unique biosynthesis, which follows a previously unknown pathway not linked to typical triterpene production pathways. Additionally, the gromomycin biosynthetic gene cluster was identified through transposon mutagenesis, as bioinformatics tools failed to detect it, emphasizing its novel biosynthetic pathway. This opens the door to new ways of understanding and developing antibiotics. As drug resistance continues to rise worldwide, new antibiotics like gromomycins are urgently needed to ensure effective treatment options. By mining bacterial genomes, we also found additional gromomycin variants, increasing the potential diversity and strength of this new class of antibiotics. This discovery not only enhances our understanding of triterpene diversity but also paves the way for a number of promising bioactive triterpenes.

## Author Contributions

A.L. and M.M. conceived the study and designed experiments. S.T. and D.B. performed compound isolation dereplication and genetic experiments. N.G., M.S., and J.Z. performed analytic and structure elucidation. H.S. and S.T. carried transposon mutagenesis and heterologous expression. F.F. and R.M. designed and performed bioactivity and toxicity assays. O.G. and V.F. isolated a gromomycin producer strain. S.T., D.B., J.Z., M.M., and A.L. prepared the manuscript. All coauthors proofread and corrected the manuscript.

## Conflict of Interests

The authors declare no conflict of interest.

## Supporting information



Supporting Information

## Data Availability

The data that support the findings of this study are available in the supplementary material of this article.
