# Supplementary material for: Gromomycins: An Unprecedented Class of Triterpene Antibiotics Produced by a Novel Biosynthetic Pathway
Source: Angew Chem Int Ed Engl. 2025 Mar 27;64(22):e202422270. doi: 10.1002/anie.202422270 (PMC12105684; doi:10.1002/anie.202422270)
Supplement: Supplementary file 1 — Supporting Information [file ANIE-64-e202422270-s001.docx]

Supplementary Information

**Gromomycins: An unprecedented class of triterpene antibiotic produced by a novel biosynthetic pathway**

Stepan Tistechok^1,2^*, Dmytro Bratiichuk^1,3^*, Hilda Sucipto^1^, Nils Gummerlich^1^, Marc Stierhof^1^, Oleksandr Gromyko^2^, Franziska Fries^3,4^, Victor Fedorenko^2^, Rolf Müller^3,4^, Josef Zapp^1^, Maksym Myronovskyi^1^, Andriy Luzhetskyy^1,3#^

*equally contributed to this work

^1^Universität des Saarlandes, Pharmazeutische Biotechnologie, Bld. C2.3, 66123 Saarbrücken, Germany; Helmholtz-Institute for Pharmaceutical Research Saarland, UdS Campus, Bld. E8.1, 66123 Saarbrücken, Germany.

^2^Ivan Franko National University of Lviv, Department of Genetics and Biotechnology, Hrushevskogo Street 4, 79005 Lviv, Ukraine.

^3^Helmholtz-Centre for Infection Research, Inhoffenstrasse 7, 38124 Braunschweig, Germany.

^4^German Center for Infection Research (DZIF), Partner Site Hannover-Braunschweig, 38124 Braunschweig, Germany.

# Corresponding author a.luzhetskyy@mx.uni-saarland.de

Table of contents

[Experimental Procedures 6](#_Toc191566521)

[Bacterial Strains, Plasmids and Culture Conditions 6](#_Toc191566522)

[Metabolite Extraction and Analysis 6](#_Toc191566523)

[Isolation and Purification of Gromomycins 6](#_Toc191566524)

[Sequencing and analysis of Streptomyces sp. Je 1-332 genome 7](#_Toc191566525)

[Cosmid Library Construction 7](#_Toc191566526)

[Transposon mutagenesis in Streptomyces sp. Je 1-332 8](#_Toc191566527)

[Genes deletion in Streptomyces sp. Je 1-332 8](#_Toc191566528)

[Heterologous expression of gromomycin gene cluster and systematic gene deletion 8](#_Toc191566529)

[Feeding experiment with labeled L-arginine for LC-MS analysis 9](#_Toc191566530)

[Feeding experiments with ^13^C-labeled pyruvates 10](#_Toc191566531)

[Genome-quided Identification of new gromomycins 10](#_Toc191566532)

[Antibiotic activity (minimum inhibitory concentrations) 10](#_Toc191566533)

[Cytotoxic activity (IC50) 11](#_Toc191566534)

[Maximum tolerated concentration 11](#_Toc191566535)

[Structure elucidation 12](#_Toc191566536)

[General 12](#_Toc191566537)

[Isolated compounds and their analytical data 12](#_Toc191566538)

[Mosher Analysis of Gromomycin B 13](#_Toc191566539)

[Isotopic enrichment of 13C-labelled gromomycin B 13](#_Toc191566540)

[List of Tables 14](#_Toc191566541)

[Table S1. NMR Data for Gromomycin A (2) in CD_3_OD. 14](#_Toc191566542)

[Table S2. NMR Data for Gromomycin A (2) in DMSO-*d_6_* + 1 drop TFA. 16](#_Toc191566543)

[Table S3. NMR Data for Gromomycin B (3) in CD_3_OD. 18](#_Toc191566544)

[Table S4. NMR Data for Gromomycin C (4) in DMSO-*d_6._* 20](#_Toc191566545)

[Table S5. NMR Data for Gromomycin D (5) in CD_3_OD. 22](#_Toc191566546)

[Table S6. NMR Data for Gromomycin E (6) in CD_3_OD. 24](#_Toc191566547)

[Table S7. NMR Data for Gromomycin F (7) in CD_3_OD. 26](#_Toc191566548)

[Table S8. NMR Data for Gromomycin G (8) in CD_3_OD. 28](#_Toc191566549)

[Table S9. NMR Data for Gromomycin H (9) in CD_3_OD. 30](#_Toc191566550)

[Table S10. NMR Data for Hexaprenylguanidine (10) in DMSO-*d_6._* 32](#_Toc191566551)

[Table S11. ^1^H NMR (CDCl_3_) data of S-MTPA ester (5a) and R-MTPA ester (5b) derived from Gromomycin B (3) and their Δδ_S-R_ values. 34](#_Toc191566552)

[Table S12. Incorporation of [2-13C]pyruvate in gromomycin B (3). 35](#_Toc191566553)

[Table S13. Incorporation of [3-13C]pyruvate in gromomycin B (3). 35](#_Toc191566554)

[Table S14. Bacterial strains used in this work. 36](#_Toc191566555)

[Table S15. Plasmids used in this work. 38](#_Toc191566556)

[Table S16. Primers used in this work. 40](#_Toc191566557)

[Table S17. Clusters of secondary metabolism genes identified in the genome of Streptomyces sp. strain Je 1-332 by the type of biosynthesis and corresponding profile using antiSMASH 6.0 software. 44](#_Toc191566558)

[Table S18. Toxicity assessment of gromomycin derivatives. 45](#_Toc191566559)

[List of Figures 46](#_Toc191566560)

[Figure S1. ^1^H NMR spectrum (500 MHz) of Gromomycin A (2) in CD_3_OD. 46](#_Toc191566561)

[Figure S2. ^13^C NMR spectrum (125 MHz) of Gromomycin A (2) in CD_3_OD. 46](#_Toc191566562)

[Figure S3. COSY spectrum (500 MHz) of Gromomycin A (2) in CD_3_OD. 47](#_Toc191566563)

[Figure S4. ^1^H-^13^C HSQC spectrum (500 MHz) of Gromomycin A (2) in CD_3_OD. 47](#_Toc191566564)

[Figure S5. ^1^H-^13^C HMBC spectrum (500 MHz) of Gromomycin A (2) in CD_3_OD. 48](#_Toc191566565)

[Figure S6. NOESY spectrum (500 MHz) of Gromomycin A (2) in CD_3_OD. 48](#_Toc191566566)

[Figure S7. Selective 1D experiments of gromomycin (2). a) selective TOCSY with excitation of H-4 (δ_H_ 3.66). b) 1D selective NOESY´s with excitations of H-25 (δ_H_ 1.40), H-26 (δ_H_ 1.20) and H-28 (δ_H_ 0.88). 49](#_Toc191566567)

[Figure S8. ^1^H NMR spectrum (700 MHz) of Gromomycin A (2) in DMSO-d_6_/1 drop TFA-d_1._ 50](#_Toc191566568)

[Figure S9. ^13^C NMR spectrum (175 MHz) of Gromomycin A (2) in DMSO-d_6_/1 drop TFA. 50](#_Toc191566569)

[Figure S10. COSY spectrum (700 MHz) of Gromomycin A (2) in DMSO-d_6_/1 drop TFA. 51](#_Toc191566570)

[Figure S11. ^1^H-^13^C HSQC spectrum (700 MHz) of Gromomycin A (2) in DMSO-d_6_/1 drop TFA. 51](#_Toc191566571)

[Figure S12. ^1^H-^13^C HMBC spectrum (700 MHz) of Gromomycin A (2) in DMSO-d_6_/1 drop TFA. 52](#_Toc191566572)

[Figure S13. 1H-^15^N HSQC spectrum of Gromomycin A (2) in DMSO-d_6_/1 drop TFA. 52](#_Toc191566573)

[Figure S14. 1H-^15^N HMBC spectrum of Gromomycin A (2) in DMSO-d_6_/1 drop TFA. 53](#_Toc191566574)

[Figure S15. ^1^H NMR spectrum (500 MHz) of Gromomycin B (3) in CD_3_OD. 54](#_Toc191566575)

[Figure S16. ^13^C NMR spectrum (125 MHz) of Gromomycin B (3) in CD_3_OD. 54](#_Toc191566576)

[Figure S17. COSY spectrum (500 MHz) of Gromomycin B (3) in CD_3_OD. 55](#_Toc191566577)

[Figure S18. ^1^H-^13^C HSQC spectrum (500 MHz) of Gromomycin B (3) in CD_3_OD. 55](#_Toc191566578)

[Figure S19. ^1^H-^13^C HMBC spectrum (500 MHz) of Gromomycin B (3) in CD_3_OD. 56](#_Toc191566579)

[Figure S20. 1D selective NOESY´s of gromomycin B (3) with excitations of H-14 (δ_H_ 2.84) and H-29 (δ_H_ 0.85). 56](#_Toc191566580)

[Figure S21. ^1^H NMR spectrum (500 MHz) of Gromomycin C (4) in DMSO-d_6._ 57](#_Toc191566581)

[Figure S22. ^13^C NMR spectrum (125 MHz) of Gromomycin C (4) in DMSO-d_6._ 57](#_Toc191566582)

[Figure S23. COSY spectrum (500 MHz) of Gromomycin C (4) in DMSO-d_6._ 58](#_Toc191566583)

[Figure S24. ^1^H-^13^C HSQC spectrum (500 MHz) of Gromomycin C (4) in DMSO-d_6._ 58](#_Toc191566584)

[Figure S25. ^1^H-^13^C HMBC spectrum (500 MHz) of Gromomycin C (4) in DMSO-d_6._ 59](#_Toc191566585)

[Figure S26. ROESY spectrum (500 MHz) of Gromomycin C (4) in DMSO-d_6._ 59](#_Toc191566586)

[Figure S27. ^1^H NMR spectrum (500 MHz) of Gromomycin D (5) in CD_3_OD. 60](#_Toc191566587)

[Figure S28. ^13^C NMR spectrum (125 MHz) of Gromomycin D (5) in CD_3_OD. 60](#_Toc191566588)

[Figure S29. COSY spectrum (500 MHz) of Gromomycin D (5) in CD_3_OD. 61](#_Toc191566589)

[Figure S30.^1^H-^13^C HSQC spectrum (500 MHz) of Gromomycin D (5) in CD_3_OD. 61](#_Toc191566590)

[Figure S31. ^1^H NMR spectrum (500 MHz) of Gromomycin E (6) in CD_3_OD. 62](#_Toc191566591)

[Figure S32. ^13^C NMR spectrum (125 MHz) of Gromomycin E (6) in CD_3_OD. 62](#_Toc191566592)

[Figure S33. COSY spectrum (500 MHz) of Gromomycin E (6) in CD_3_OD. 63](#_Toc191566593)

[Figure S34. ^1^H-^13^C HSQC spectrum (500 MHz) of Gromomycin E (6) in CD_3_OD. 63](#_Toc191566594)

[Figure S35. ^1^H-^13^C HMBC spectrum (500 MHz) of Gromomycin E (6) in CD_3_OD. 64](#_Toc191566595)

[Figure S36. ^1^H NMR spectrum (500 MHz) of Gromomycin F (7) in CD_3_OD_._ 65](#_Toc191566596)

[Figure S37. ^13^C NMR spectrum (125 MHz) of Gromomycin F (7) in CD_3_OD. 65](#_Toc191566597)

[Figure S38. COSY spectrum (500 MHz) of Gromomycin F (7) in CD_3_OD. 66](#_Toc191566598)

[Figure S39. ^1^H-^13^C HSQC spectrum (500 MHz) of Gromomycin F (7) in CD_3_OD. 66](#_Toc191566599)

[Figure S40. ^1^H-^13^C HMBC spectrum (500 MHz) of Gromomycin F (7) in CD_3_OD. 67](#_Toc191566600)

[Figure S41. ^1^H NMR spectrum (500 MHz) of Gromomycin G (8) in CD_3_OD. 68](#_Toc191566601)

[Figure S42. ^13^C NMR spectrum (125 MHz) of Gromomycin G (8) in CD_3_OD. 68](#_Toc191566602)

[Figure S43. COSY spectrum (500 MHz) of Gromomycin G (8) in CD_3_OD. 69](#_Toc191566603)

[Figure S44. ^1^H-^13^C HSQC spectrum (500 MHz) of Gromomycin G (8) in CD_3_OD. 69](#_Toc191566604)

[Figure S45. ^1^H-^13^C HMBC spectrum (500 MHz) of Gromomycin G (8) in CD_3_OD. 70](#_Toc191566605)

[Figure S46. ^1^H NMR spectrum (500 MHz) of Gromomycin H (9) in CD_3_OD. 71](#_Toc191566606)

[Figure S47. ^13^C NMR spectrum (125 MHz) of Gromomycin H (9) in CD_3_OD. 71](#_Toc191566607)

[Figure S48. COSY spectrum (500 MHz) of Gromomycin H (9) in CD_3_OD. 72](#_Toc191566608)

[Figure S49. ^1^H-^13^C HSQC spectrum (500 MHz) of Gromomycin H (9) in CD_3_OD. 72](#_Toc191566609)

[Figure S50. ^1^H-^13^C HMBC spectrum (500 MHz) of Gromomycin H (9) in CD_3_OD. 73](#_Toc191566610)

[Figure S51. ^1^H NMR spectrum (500 MHz) of Hexaprenylguanidine (10) in DMSO-d_6._ 74](#_Toc191566611)

[Figure S52. ^13^C NMR spectrum (125 MHz) of Hexaprenylguanidine (10) in DMSO-d_6._ 74](#_Toc191566612)

[Figure S53. COSY spectrum (500 MHz) of Hexaprenylguanidine (10) in DMSO-d_6._ 75](#_Toc191566613)

[Figure S54. ^1^H-^13^C HSQC spectrum (500 MHz) of Hexaprenylguanidine (10) in DMSO-d_6._ 76](#_Toc191566614)

[Table S55. ^1^H-^13^C HMBC spectrum (500 MHz) of Hexaprenylguanidine (10) in DMSO-d_6._ 76](#_Toc191566615)

[Table S56. ^1^H-^15^N HSQC spectrum (500 MHz) of Hexaprenylguanidine (10) in DMSO-d_6._ 77](#_Toc191566616)

[Figure S57. ^1^H NMR spectrum (500 MHz) of Gromomycin D (*R*)-MTPA ester (5a) in CDCl_3._ 78](#_Toc191566617)

[Figure S58. COSY spectrum (500 MHz) of Gromomycin D (*R*)-MTPA ester (5a) in CDCl_3._ 78](#_Toc191566618)

[Figure S59. ^1^H-^13^C HSQC spectrum (500 MHz) of Gromomycin D (*R*)-MTPA ester (5a) in CDCl_3._ 79](#_Toc191566619)

[Figure S60. ^1^H NMR spectrum (500 MHz) of Gromomycin D (*S*)-MTPA ester (5b) in CDCl_3._ 80](#_Toc191566620)

[Figure S61. COSY spectrum (500 MHz) of Gromomycin D (*S*)-MTPA ester (5b) in CDCl_3._ 80](#_Toc191566621)

[Figure S62. ^1^H-^13^C HSQC spectrum (500 MHz) of Gromomycin D (*S*)-MTPA ester (5b) in CDCl3. 81](#_Toc191566622)

[Figure S63. ^13^C NMR spectrum (125 MHz) of Gromomycin B (3) in CD_3_OD from feeding experiment with [2-^13^C] pyruvate. 82](#_Toc191566623)

[Figure S64. ^13^C NMR spectrum (125 MHz) of Gromomycin B (3) in CD_3_OD from feeding experiment with [3-^13^C] pyruvate. 82](#_Toc191566624)

[Figure S65. Heterologous expression of the cosmid P04_E01 with gromomycin cluster into *S. albus* Del14. Peaks induced by gromomycin are indicated by asterisks. 83](#_Toc191566625)

[Figure S66. Incorporation of labelled L-arginine into gromomycins. 83](#_Toc191566626)

[Figure S67. Heterologous expression of the P03_G02 cosmid with gromomycin-like cluster into *S. albus* Del14 strain. 83](#_Toc191566627)

[Figure S68. Linear methylated hexaprenylguanidine (marked with asterisks). 84](#_Toc191566628)

[Figure S69. Deletion into *Streptomyces sp.* Je 1-332 strain. 84](#_Toc191566629)

[Figure S70. HPLC-MS chromatogram of crude extract of *Streptomyces sp.* Je 1-332 strain. 85](#_Toc191566630)

[Figure S71. HPLC-MS chromatograms of the *S. albus* with the *gro*-gene deletion. 85](#_Toc191566631)

[Figure S73. Kaplan-Meier curves of zebrafish embryos exposed to different concentrations of gromomycin B (A), gromomycin A (B), gromomycin F (C), gromomycin G (D) and gromomycin E (E). 87](#_Toc191566632)

[Figure S74. Scheme for identifying gene cluster involved in gromomycin biosynthesis. 88](#_Toc191566633)

# Experimental Procedures

## Bacterial Strains, Plasmids and Culture Conditions

All strains and plasmids used in this work are listed in Supplementary Tables 14 and 15. *Streptomyces* sp. strain Je 1-332 was isolated from the rhizosphere soil of *Juniperus excelsa*.^[1]^ *S. albus* Del14^[2]^ and *S. lividans* Del8^[3]^ were used as hosts for heterologous expression of the gromomycin biosynthetic gene clusters. *E. coli* strains were grown in Luria-Bertani (LB) broth (Sigma-Aldrich, St. Louis, MO, USA). *Streptomycetes* strains were grown on MS agar medium (Soy flour 20 g, Mannitol 20 g, tap water 1 l, pH 7.2 prior autoclaving) and in liquid tryptic soy broth medium (TSB; Sigma-Aldrich, St. Louis, MO, USA). If necessary, the following antibiotics were added: apramycin (50 μg ml^-1^), hygromycin (120 μg ml^-1^), chloroamphenicol (12.5 µg ml^-1^), phosphomycin (100 μg ml^-1^), thiostrepton (0.5 μg ml^-1^), kanamycin (50 μg ml^-1^) and nalidixic acid (50 μg ml^-1^) (Sigma-Aldrich, St. Louis, MO, USA; Roth, Karlsruhe, Germany). X-gluc (5-bromo-4-chloro-3-indolyl-beta-D-glucuronic acid) (ThermoFisher scientific, Waltham, MA, USA) at a concentration of 50 µg ml^-1^ was used to determine β-glucuronidase activity.

For conjugation, the *Streptomycetes* strains were grown on MS agar for sporulation. For gromomycins production, S*treptomyces* strains were grown in liquid DNPM medium (40 g l^-1^ dextrin, 7.5 g l^-1^ soytone, 5 g l^-1^ baking yeast, and 21 g l^-1^ MOPS, pH 7.2), SG (20 g l^-1^ glucose, 10 g l^-1^ soy peptone, and 2 g l^-1^ CaCO_3_, pH 7.2), ISP-2 (4 g l^-1^ yeast extract, 10 g l^-1^ malt extract, 4 g l^-1^ dextrose, pH 7.2) and minimal medium (MM)^[4]^. For feeding experiments, a MM was used.

## Metabolite Extraction and Analysis

*Streptomyces* sp. Je 1-332 or *S. albus* strains were cultivated in 25 ml TSB medium for 48 h at 28 °C to obtain a pre-culture. The main cultures containing 100 ml of DNPM, SG or MM were inoculated with 1 ml of pre-culture. After 7 days of cultivation at 28 °C, the gromomycins were extracted with ethyl acetate from the supernatant, followed by solvent evaporation. One μl of sample was separated using a Dionex Ultimate 3000 UPLC (Thermo Fisher Scientific, Waltham, MA, USA), a 10-cm ACQUITY UPLC® BEH C18 column, 1.7 μm (Waters, Milford, MA, USA) and a linear gradient 5% to 95% of 0.1% formic acid (FA) solution in acetonitrile versus 0.1% FA solution in water for 18 min at a flow rate of 0.6 ml min^-1^ and 45 °C. Samples were analyzed using an amaZon speed mass spectrometer or maXis high-resolution LC-QTOF system (Bruker, USA). Data were collected and analyzed with the Bruker Compass Data Analysis software, version 4.2 (Bruker, Billerica, MA, USA).

## Isolation and Purification of Gromomycins

For gromomycins production, cultures were grown in 10 L of DNPM or SG medium 7 days at 28 °C with stirring at 180 rpm. Gromomycins were extracted with ethyl acetate from the culture supernatant and with a mixture of methanol acetone at a 1:1 ratio from the biomass. The obtained extracts were dissolved in methanol and used for purification of the gromomycin an Isolera^TM^ One flash purification system (Biotage, Uppsala, Sweden). For this purpose, SNAP Ultra 50 g (Biotage, Uppsala, Sweden) was used as stationary phase and n-hexane (A)/ chloroform (B)/ ethyl acetate (C)/ methanol (D) as mobile phase in a linear gradient A/B 10 column volumes (CV), B/C 15 CV, C/D 15 CV at a flow rate of 100 ml min^-1^. The fractions containing gromomycins were pooled together, concentrated and used for the Size-Exclusion Chromatography on the Sephadex LH-20 column (Sigma-Aldrich, Louis, MO, USA) with methanol as a mobile phase. Fractions were collected every 10 min at a flow rate of 0.8 ml min^-1^. The fractions containing gromomycins were pooled together, concentrated and dissolved in methanol. The second purification stage was Reversed Phase (RP) HPLC (Waters AutoPuruficationTM System), separation on preparative C18 column Nucleodur HTec, 5 µm, 250 mm x 21 mm (Macherey-Nagel, Germany) using a water solution containing 0.1%(v/v) formic acid (solvent A), and an acetonitrile solution containing 0.1% (v/v) formic acid (solvent B) as a mobile phase. The following gradient at a flow rate of 20 mL/min was used for compounds separation: 0 min—5% B, 0.5 min—5%, 12 min—65% B, 25 min—65%B, 27 min—95% B, 28 min—95% B, 29 min—5% B, 31 min—5% B. The fractions were collected using MS detector (WatersTM SQ Detector 2). The gromomycin-containing fractions were pooled together, evaporated and used for the last purification step.

The last purification stage was reversed-phase High performance liquid chromatography (HPLC), separation on semipreparative C18 column SynergiTM 4 μm Fusion-RP 80 Å 250×10 (Phenomenex, Torrance, CA, USA) using water + 0.1% formic acid (A) and acetonitrile + 0.1% formic acid (B) as a mobile phase. The following gradient at a flow rate of 4 mL/min was used for separation: 0 min – 5% B, 0.5 min – 5%, 10 min – 65%, 12 min – 65%, 15 min – 95%, 17 min – 95% B, 18 min – 5% B. Fractions containing pure compound were pooled together and evaporated.

## Sequencing and analysis of Streptomyces sp. Je 1-332 genome

Genomic DNA of *Streptomyces* sp. Je 1-332 was isolated from 50 ml culture grown in TSB medium at 28°C on a rotary shaker (180 rpm) for 72 hours. The salting out procedure was used to obtain total DNA, followed by RNAse treatment.^[4]^ The purity and concentration of genomic DNA were determined using 1% agarose gel electrophoresis and a Nanodrop 2000 spectrophotometer (Thermo Fisher Scientific, USA). The Illumina paired-end sequencing library (TruSeq sample preparation kit; Illumina, USA) was created for genome sequencing according to the manufacturer's protocol. The genome sequence of *Streptomyces* sp. Je 1-332 was obtained on the Illumina MiSeq system in rapid run mode (2x250 nt) with a pair spacing of about 500 bp. After sequencing and processing the obtained data, a *de novo* assembly was performed using the SPAdes Assembler program (version 3.8.1) with default settings.^[5]^ Genome annotation was performed using the prokka v1.11 platform and GenDB 2.0.^[6-7]^ AntiSMASH 6.0 was used to identify clusters of secondary metabolites.^[8]^ The assembled and annotated sequence of the *Streptomyces* sp. Je 1-332 was deposited in the GenBank database under accession number NZ_CP160402.1.

## Cosmid Library Construction

A cosmid libraries of streptomycetes were prepared using the EpiCentre CopyControl™ Fosmid Library Production Kit in pCos15A_gusA or pCos15A_AmInt vectors by adapting the protocol from Lucigen. A library of 30−40 kb fragments was constructed according to the manufacturer’s protocol using genomic DNA partially digested with MssI. The purified genomic DNA fragments were ligated into linearized pCos15A_gusA or pCos15A_AmInt vectors. The ligation reactions were packaged into λ phage for *E.coli* EPI300 infection. The packaged library was plated on LB agar plates containing 12.5 µg ml^-1^ chloroamphenicol, and grown overnight at 37 °C. Approximately 1800 single colonies were picked and inoculated into individual wells of 96-well plates. This arrayed cosmid library was stored with 20% glycerol and kept at −80 °C.

## Transposon mutagenesis in Streptomyces sp. Je 1-332

Spores of streptomycetes exconjugants transformed with a pTNM plasmid,^[9]^ that contains Tn5 transposon were scraped from the MS agar plate by sterile MQ water. One ml of the spores transferred in 100 ml Erlenmeyer shake flask containing 20 ml TSB medium with 200 μg ml^-1^ of phosphomycin and 25 μg ml^-1^ of apramycin for a plasmid selection. After 24 hours of cultivation, 0.5 μg ml^-1^ of thiostrepton was added to induce Tn5 through the thiostrepton-induced promoter. After reaching the stationary phase, 1 ml of culture was transferred into a 100 ml Erlenmeyer flask with 20 ml of TSB medium and phosphomycin and incubated at 37 °C, 180 rpm until the stationary phase was reached. This step was repeated twice. After three passages, 1 ml of the transposon mutant’s culture was plated on MS agar plate with phosphomycin and an apramycin. The spores were collected and serial dilutions were made. 1 ml of each dilution, starting from 10^-6^, was plated on MS agar plate and incubated for 5-7 days at 28 °C. The obtained colonies were selected for plasmid loss testing.

To generate rescue plasmids, genomic DNAs of strains *Streptomyces* sp. Je 1-332 with Tn5 transposon were isolated and digested with SacII restriction enzyme (Thermo Fisher Scientific, Waltham, MA, USA) for overnight at 37°C, then the chromosomal DNA was precipitated with ethanol, dissolved in 16 μl of MQ water and, after 2 μl of ligation buffer and 2 μl of T4-DNA ligase (Thermo Fisher Scientific, Waltham, MA, USA) were added, selfligated overnight at 16 °C. 3, 5 or 7 μl of selfligated DNA was transformed into the *E. coli* TransforMaxTM EC100DTM pir-116 electrocompetent cells and plated on selective LB medium. The rescue plasmids were isolated and sequenced by GATC-Biotech or by Genewiz using the sequencing primers pMODseq-f and pMODseq-r (Supplementary Table 16).

## Genes deletion in Streptomyces sp. Je 1-332

Red/ET recombination was used to delete the desired genes in the genome of *Streptomyces* Je 1-332 strain. A linear DNA fragment containing the apramycin resistance and origin of transfer (*oriT*) flanked by suitable homology arms was generated by PCR using specific primers. The primers used for the deletions are listed in the Supplementary Table 16. PCR was carried out with Dream Taq DNA polymerase (Thermo Fisher Scientific, Waltham, MA, USA), according to the manufacturer’s protocol. The respective cosmids containing the appropriate gene for deletion were transformed together with the amplified cassette into the *E. coli* GB05-red strain. Transformants were selected for resistance to apramycin and chloramphenicol. Correct transformants were verified by the restriction analysis and sequencing of the isolated cosmid DNA using the specific primers.

The cosmids with gene deletion was transferred from *E. coli* ET12567 pUB307 cells into *Streptomyces* sp. Je 1-332 cells by means of conjugation.^[4]^ Transconjugants were selected for resistance to apramycin and glucuronidase activity. For the generation of the *Streptomyces* sp. Je 1-332 deletion mutants, single-crossover apramycin and blue mutants were screened for the loss of glucuronidase activity (blue pigmentation) as the result of a double-crossover event. The replacement of the genes was confirmed by PCR using the specific primer pair (Supplementary Table 16).

## Heterologous expression of gromomycin gene cluster and systematic gene deletion

The constructs used in this study for gene disruption and heterologous expression are summarized in the Supplementary Table 15. The constructs were introduced into *S. albus* Del14 and *S. lividans* Del8 by conjugation using donor strain E. coli ET12657/pUB307 on MS plates. After incubating at 30°C for 16 hours, exconjugants were selected with 50 μg ml^-1^ apramycin and and 30 μg ml^-1^ nalidixic acid. Single colonies from this plate were patched onto MS plates 50 μg ml^-1^ apramycin. Candidate colonies were used for further identification by PCR analysis.

The antibiotic cassette apramycin together with *oriT* and integrase genes were amplified by PCR with primers OTC60 and OTC61 using pCos15A_gus_AmInt plasmid as template. Utilizing PCR-based λ-red recombination technique, the amplified cassettes was integrated into cosmid P04_E01 forming pHSU-STS10. pHSU-STS10 was subsequently introduced into *S. albus* Del14 by conjugation and was selected for apramycin resistance. The cluster integration was confirmed by PCR using primers STS40 and STS41, and the resulting strain is named *S. albus* STS10.

To define the minimal gene cluster of gromomycin, a systematic genes deletion was conducted from the downstream and upstream of the pHSU-STS10. In general, the genes that were intended to be deleted were initially replaced by ampicillin resistance (*amp*^R^) cassette using RedET recombination. For the construction of the pHSU-STS22, pHSU-STS23, pHSU-STS24, pHSU-STS25, pHSU-STS29 and pHSU-STS30 the *amp*^R^ cassette was amplified with the pairs of primers STS51 and STS52, STS47 and STS49, STS48 and STS49, STS50 and STS49, STS51 and STS72, respectively. For the construction of the pHSU-STS31, pHSU-STS32 and pHSU-STS33 the *amp*^R^ cassette was amplified with the pairs of primers STS67 and STS68, STS69 and STS68, STS70 and STS68, respectively. Subsequently, the *amp*^R^ was removed by digestion using PmeI or SnaBI and religated to generate appropriate constructs. STS40 and STS41 primers were used to test for mutant integration by PCR.

To determine the role of the genes in the identified gromomycin gene cluster, a series of gene inactivations were performed using the pHSU-STS33 vector. The derivatives of pHSU-STS33 with gene deletions were constructed using the RedET approach. For this, the antibiotic resistance marker was amplified by PCR with primers harbouring overhang regions complementary to the boundaries of the DNA to be deleted. The amplified fragment was used for recombineering of the pHSU-STS33. The recombinant BACs were analysed by PCR. The primers used for recombineering purposes are listed in Supplementary Table 16. For the construction of the pHSU-STS33_KOgroA, pHSU-STS33_KOgroB, pHSU-STS33_KOgroC, pHSU-STS33_KOgroD, pHSU-STS33_KOgroE, pHSU-STS33_KOgroF, pHSU-STS33_KOgroG, pHSU-STS33_KOgroH and pHSU-STS33_KOgroI, the hygromycin marker from phygattB^[2]^ was amplified with the primers KO_groA_F/KO_groA_R, KO_groB_F/KO_groB_R, KO_groC_F/KO_groC_R, KO_groD_F/KO_groD_R, KO_groE_F/KO_groE_R, KO_groF_F/KO_groF_R, KO_groG_F/KO_groG_R, KO_groH_F/KO_groH_R and KO_groI_F/KO_groI_R, respectively. Next, the antibiotic resistance gene was excised with the AscI restriction enzyme to yield marker-free mutants. The marker excision was verified using the appropriate primers listed in Supplementary Table 16.

## Feeding experiment with labeled L-arginine for LC-MS analysis

Feeding experiments for LC-MS analysis were performed using 1 mM of L-[^13^C_6_, ^15^N_4_]-Arg (Sigma-Aldrich) and L-[^15^N_4_]-Arg (Sigma-Aldrich) in 100 mL liquid culture. Precursors were dissolved in 1 ml MQ H_2_O and added to the culture in 4 portions (53.65 µl) at 24, 48, 72 and 96 h after inoculation of preculture. Supernatant was separated from culture biomass by centrifugation and extracted with ethyl acetate. Solvent was removed under vacuo, resuspended in 500 µl methanol and resulting crude extract was analyzed by LC-MS.

## Feeding experiments with ^13^C-labeled pyruvates

Feeding experiments were performed using 10mM of [2-¹³C]-, [3-^13^C] sodium pyruvate (Sigma-Aldrich) in total of 3 l liquid culture. In all cases, 3.3 g precursors were dissolved in 14 mL sterile water. A 100 μl aliquot was fed to 100 ml growing culture (total 30 flasks) at 24, 48, 72 and 96 h after inoculation. The fermentation was continued for six days and the culture was extracted as described above. Purification of carbon enriched gromomycin was performed as described above. ^13^C-NMR data of individual compounds have been acquired.

## Genome-quided Identification of new gromomycins

Three genes involved in the biosynthesis of gromomycins were used for genome-wide quantitative screening of new gromomycins: gene 5 (*groD*), gene 7 (*groF*) and gene 9 (*groH*). The nucleotide sequences of these genes were used as probes in the NCBI protein BLAST database to identify new gromomycin-like clusters.^[10]^ The strains with all three of these genes identified in the genome were selected. In this way, several actinomycetes strains were identified, including *S. flavoviridis* strain ISP-5153 (GenBank accession number ASM1464951v1). The new gromomycin-like cluster was isolated from the genome of the *S. flavoviridis* ISP-5153 strain through the cosmid library construction according to the manual (CopyControl™ Fosmid Library Production Kit). Cosmid P03_G02 containing the whole gromomycin-like cluster was identified by pool PCR with two pairs of primers (Left_flavoridis_F/ Left_flavoridis_R and Right_flavoridis_F/ Right_flavoridis_R) designed to amplify regions to the left or right side of the cluster. The P03_G02 cosmid was transferred to *S. albus* Del14 and *S. lividans* Del8 strains for heterologous expansion. The ability of heterologous strains to produce gromomycin was determined by HPLC.

## Antibiotic activity (minimum inhibitory concentrations)

Gromomycin stock solutions were prepared in dimethyl sulfoxide (DMSO). All microorganisms used in this study were obtained from the German Collection of Microorganisms and Cell Cultures (DSMZ), the American Type Culture Collection (ATCC), the Coli Genetic Stock Center, or were part of our internal strain collection. *Staphylococcus aureus* strains Newman, N315, Mu50 and Cowan 1 were obtained from M. Bischoff, Saarland University Hospital, Homburg. *S. aureus* wild type and Dap^R^ HG001^[11]^ were provided by T. Schneider, University of Bonn. *E. coli* WO153 was provided by K. Lewis, Northeastern University, Boston, USA. Minimum inhibitory concentrations (MICs) were determined using the broth microdilution method according to EUCAST guidelines (ISO 20776-1:2019). In short, serial two-fold dilutions of gromomycins (0.03125 to 64 µg/mL) were prepared in 75 µL of cation-adjusted Mueller-Hinton broth (MHB2) in sterile 96-well plates. Equal volume of the bacterial suspension was added and the plates were incubated at 37 °C for 18 h. For *Streptococcus pneumoniae*, MHF broth (MHB2 supplemented with 5% lysed horse blood and 20 mg/L β-NAD) was used and plates were incubated at 37 °C with 5% CO_2_. The MIC was defined as the lowest concentration of the antibiotic causing complete inhibition of visible growth of the microorganism. The same method was used for testing *Mycobacterium smegmatis*, but with the use of Middlebrook 7H9 complete medium supplemented with oleic acid, albumin, dextrose and catalase (OADC, 10%). *M. smegmatis* plates were incubated for 48 h at 37 °C. For assessing activity against *Mycobacterium tuberculosis,* an adapted resazurin microtitre assay (REMA) was performed as previously described.^[12]^ In short, *M. tuberculosis* single cells were prepared and added to compound dilutions in M7H9. Plates were incubated for 6 d at 37 °C, followed by addition of 50 µL of resazurin and incubation for another day at 37 °C. The MIC was determined visually and additionally confirmed by measuring fluorescence (excitation at 530 nm, emission at 590 nm).

## Cytotoxic activity (IC50)

HepG2 cells (human hepatoblastoma cell line; ACC 180) and CHO-K1 (chinese hamster ovary cells; ACC 110) were obtained from the German Collection of Microorganisms and Cell Cultures (DSMZ) and cultured under the conditions recommended by the depositor. Cells were propagated in Roswell Park Memorial Institute (RPMI) 1640 medium and Ham’s F12 medium, respectively, supplemented with 10% fetal bovine serum (FBS), and seeded at 6×10^3^ cells per well of 96-well plates in 120 μL of complete medium. After 2 h of equilibration (37 °C, 5% CO_2_), the cells were treated with a serial dilution of gromomycins. Gromomycins, doxorubicin as reference, as well as the solvent control (DMSO) were tested as duplicates in two independent experiments. After 5 d of incubation (37 °C, 5% CO_2_), a total of 20 μL of 5 mg/ml MTT (thiazolyl blue tetrazolium bromide) in phosphate-buffered saline (PBS) were added to each well and the cells were further incubated for 2 h at 37 °C before the supernatant was discarded. Subsequently, the cells were washed with 100 μL of PBS and treated with 100 μl of 2-propanol/10 N HCl (250:1) to dissolve formazan granules. Cell viability was measured as a percentage relative to the respective solvent control by measuring the absorbance at 570 nm using a microplate reader (Tecan Infinite M200Pro). GraphPad Prism (version 10.0.3, GraphPad, Boston, MA, USA) was used for sigmoidal curve fitting to determine the IC_50_ values.

## Maximum tolerated concentration

Husbandry of adult zebrafish was performed according to internal guidelines set out in the German Animal Welfare Act (§11 Abs. 1 TierSchG). Experiments were carried out with wild type AB (obtained from the European Zebrafish Resource Center at Karlsruhe Institute of Technology) embryos within the first 120 hours post fertilization (hpf) as these early life stages are not considered as animal experiments according to the EU Directive 2010/63/EU.^[13]^ Embryos were maintained in fresh 0.3x Danieau’s (17.4 mM NaCl, 0.21 mM KCl, 0.12 mM MgSO_4_, 0.18 mM Ca(NO_3_)_2_, 1.5 mM HEPES, 1.2 µM methylene blue, pH 7.1-7.3) at 28 °C. At a maximum of 120 hpf, embryos were euthanized by submersion in ice water for at least 12 h.

For evaluation of the maximum tolerated concentration (MTC), embryos were dechorionated at 30 hpf using 1 mg/mL pronase and placed in a flat-bottom 96-well plate with one embryo per well. Excess medium was removed and 150 µL of gromomycin dilutions (in 0.3x Danieau’s, maximum of 1% DMSO) and of the solvent control (1% DMSO in 0.3x Danieau’s) were added. Ten embryos were used per condition. Exposed embryos were maintained at 28 °C until 120 hpf and they were monitored daily under a stereo microscope (Stemi 508, Zeiss) in order to record survival as well as anomalies, pigmentation, heartbeat and locomotor responses. An embryo was considered dead when no heartbeat could be observed. The maximum tolerated concentration (MTC) was defined as the highest concentration of the antibiotic with more than 90% survival of zebrafish embryos. Kaplan-Meier curves were generated using GraphPad Prism (version 10.0.3, GraphPad, Boston, MA, USA).

# Structure elucidation

## General

Chiroptical measurements ($\text{[α]}_{\text{D}}^{\text{20}}$) were obtained on a Perkin Elmer (Model 341) polarimeter in a 100 × 2mm cell at 20°C. NMR spectra were obtained for Gromomycin A on a Bruker AVANCE III 700 MHz spectrometer equipped with a helium cooled cryoprobe and for all the other compounds on a Bruker AVANCE NEO 500 spectrometer equipped with a nitrogen cooled cryoprobe system (Bruker Biospin GmbH, Germany), in the solvents indicated and referenced to residual ^1^H signals in deuterated solvents.

## Isolated compounds and their analytical data

**Gromomycin A** (**2**) (3*S*, 4*S*, 6*S*, 7*S*, 10*R*, 11*S*, 19*R*) white powder, $\text{[α]}_{\text{D}}^{\text{20}}\text{ +9° (c 0.20, MeOH)}$. For NMR see Table S1 (700 MHz, CD3OD) and S2 (700 MHz, DMSO-d_6_). HRESIMS *m/z* 478.3804 [M+H]^+^, (calcd for C_31_H_48_N_3_O, 478.3792).

**Gromomycin B** (**3**) (3*S*, 4*S*, 6*S*, 7*S*, 10*R*, 11*S*, 14*S*, 19*R*) white powder, $\text{ }\text{[α]}_{\text{D}}^{\text{20}} \text{-28° (c 0.20, MeOH).}$ For NMR see Table S3 (500 MHz, CD_3_OD). HRESIMS *m/z* 494.3752 [M+H]^+^, (calcd for C_31_H_48_N_3_O_2_, 494.3741).

**Gromomycin C** (**4**) (3*S*, 4*S*, 6*S*, 7*S*, 10*R*, 11*S*, 14*S*, 19*R*) white powder, $\text{[α]}_{\text{D}}^{\text{20}}\text{ -14° (c 0.64, MeOH)}$. For NMR see Table S4 (500 MHz, DMSO-d_6_). HRESIMS *m/z* 480.3953 [M+H]^+^, (calcd for C_31_H_50_N_3_O, 480.3948).

**Gromomycin D** (**5**) (3*S*, 4*S,* 6*S*, 7*S*, 10*R*, 11*S*, 19*R*) white powder, $\text{[α]}_{\text{D}}^{\text{20}}\text{ -23° (c 0.11, MeOH). }$ For NMR see Table S5 (500 MHz, CD_3_OD). HRESIMS *m/z* 494.3751 [M+H]^+^, (calcd for C_31_H_48_N_3_O_2_, 494.3741).

**Gromomycin E** (**6**) (3*S*, 4*S*, 6*S*, 7*S*, 10*R*, 11*S*, 19*R*, 22*ξ*) white powder, $\text{[α]}_{\text{D}}^{\text{20}}\text{ -1° (c 0.27, MeOH). }$For NMR see Table S6 (500 MHz, CD_3_OD). HRESIMS *m/z* 492.3950 [M+H]^+^, (calcd for C_32_H_50_N_3_O, 492.3948).

**Gromomycin F** (**7**) (3*S*, 4*S*, 6*S*, 7*S*, 10*R*, 11*S*, 14*S*, 19*R*, 22*ξ*) white powder, $\text{ [α]}_{\text{D}}^{\text{20}}\text{ -39° (c 0.16, MeOH). }$For NMR see Table S7 (500 MHz, CD_3_OD). HRESIMS *m/z* 508.3895 [M+H]^+^, (calcd for C_32_H_50_N_3_O_2_, 508.3898).

**Gromomycin G** (**8**) (3*S*, 4*S*, 6*S*, 7*S*, 10*R*, 11*S*, 14*S*, 19*R*, 22*ξ*) white powder, $\text{[α]}_{\text{D}}^{\text{20}}\text{ 0°(c 0.20, MeOH) .}$ For NMR see Table S8 (500 MHz, CD_3_OD). HRESIMS *m/z* 494.4105 [M+H]^+^, (calcd for C_32_H_52_N_3_O, 494.4105).

**Gromomycin H** (**9**) (3*S*, 4*S*, 6*S*, 7*S*, 10*R*, 11*S*, 19*R*, 22*ξ*) white powder, $\text{[α]}_{\text{D}}^{\text{20}}\text{ -13° (c 0.21, MeOH). }$ For NMR see Table S9 (500 MHz, CD_3_OD). HRESIMS *m/z* 508.3899 [M+H]^+^, (calcd for C_32_H_50_N_3_O_2_, 508.3898).

**Hexaprenylguanidine** (**10**): 1-((2*E*,6*E*,10*E*,14*E*,18*E*)-3,7,11,15,19,23-hexamethyltetra-cosa-2,6,10,14,18,22-hexaen-1-yl) guanidine. White powder. For NMR see Table S10 (500 MHz, DMSO-d_6_). HRESIMS *m/z* 468.4355 [M+H]^+^, (calcd for C_31_H_54_N_3_, 468.4318).

## Mosher Analysis of Gromomycin B

The absolute stereochemistry was determined according to Mosher et al.^[14]^ In brief, two glass vials with 500 µg dried Gromomycin B in 100 µL dry CDCl3, 10 µL dry pyridine and 10 µL *R*-MTPA-Cl or *S*-MTPA-Cl, respectively, were prepared. The vials were flushed with nitrogen, closed airtight and were incubated with occasional shaking at room temperature while the reaction progress was monitored by LC-MS analysis by a Bruker Amazon Ion trap. Full conversion to the Mosher conjugate (*m/z* 710.44, Fig.1) was observed after 45 min. Subsequently, the samples were dried, dissolved in methanol and purified by HPLC using an Agilent 1100 system equipped with a Phenomenex Synergi C18 column and a 15 min gradient of 40-95% acetonitrile/water containing 0.1% formic acid. The purified samples were dissolved in CD_3_OD and NMR spectroscopy was performed.

494 Mosher R-MTPA_GB1_01_90538.d

494 Mosher-S-MTPA_GA3_01_91574.d

0

1

2

3

8

x10

Intens.

0.0

0.5

1.0

1.5

9

x10

Intens.

0

2

4

6

8

10

12

14

16

18

Time [min]

710.44

1+

711.42

1+

712.42

1+

+MS, 13.9min

0.0

0.5

1.0

1.5

9

x10

Intens.

708

710

712

714

m/z

Structure of the Mosher conjugate (m/z 710.44).

## Isotopic enrichment of 13C-labelled gromomycin B

Distribution of the isotopic label was determined by comparing ^13^C NMR spectra of the enriched and natural abundance gromomycin B.^[15]^ All compounds were recorded under identical experimental conditions (zgpr pulse program from Bruker pulse library with D1=3) corresponding to identical nuclear Overhauser effects and relaxation times for both measurements. As it was impossible to work with identical concentrations for the unlabelled reference and the biosynthetically labelled compounds, the spectra were normalized with reference to the signal of DMSO-d_6_ as internal standard.

# List of Tables

## Table S1. NMR Data for Gromomycin A (2) in CD_3_OD.

| Nr. | δ_C_ | δ_H_ (*J* in Hz) | HHCOSY | HMBC (C→H) | NOESY Key Correlations |
| --- | --- | --- | --- | --- | --- |
| 1 | 116.07 CH | 5.85 br s |  |  | 8ab, |
| 2 | 132.32 C |  |  | 1, 25, 26 |  |
| 3 | 59.74 C |  |  | 1, 4, 5ab, 25 |  |
| 4 | 79.78 CH | 3.66 dd (*12, 4.5*) | 5ab, 6 | 5ab, 6, 25 | 6 |
| 5 | 29.15 CH_2_ | a: 1.73 dd* (*12, 4.5*)  b: 1.58 ddd* (*12, 12, 12*) | 4, 6  4, 6 | 4, 6 | 25, 26, 28 |
| 6 | 41.55 CH | 1.67 d (*12*) | 5ab | 4, 5ab, 12ab, 26, 27, 28 | 4, |
| 7 | 38.54 CH |  |  | 26 |  |
| 8 | 35.14 CH_2_ | a: 1.45 m  b: 1.21 m | 8b, 9ab  8a, 9ab | 9ab | 1  1 |
| 9 | 29.02 CH_2_ | a: 1.77 m  b: 1.70 m | 9b, 8ab  9a, 8ab | 8ab, 27 |  |
| 10 | 41.56 C |  |  | 27, 28 |  |
| 11 | 38.11 C |  |  | 6, 13a, 7, 27, 28 |  |
| 12 | 31.15 CH_2_ | a: 1.60 m  b: 1.50 m | 12b,13ab  12a,13ab | 6, 13ab, 28 |  |
| 13 | 22.89 CH_2_ | a: 2.04 dd (*19.0, 5.5*)  b : 1.87 m | 13b,12ab, 18a  13a,12ab, 18a | 12ab, |  |
| 14 | 137.80 C |  |  | 12a, 13a, 16, 17, 18ab, 20ab, 29 |  |
| 15 | 133.81 C |  |  | 9b, 13ab, 17, 27 |  |
| 16 | 124.69 CH | 5.99 d (*9.5*) | 17, 18ab | 18b | 9a, 17, 27 |
| 17 | 125.34 CH | 5.75 dt (*9.5, 4.5*) | 16, 18ab | 18b | 16, 18ab |
| 18 | 36.25 CH_2_ | a: 2.20 dt (*17.0, 3.0*)  b: 1.81 m | 13ab, 16,17, 18b  16,17, 18a | 17, 20ab, 29 | 17, 20ab, 29  17 |
| 19 | 38.57 C |  |  | 18b, 29 |  |
| 20 | 39.91 CH_2_ | a: 1.44 m  b: 1.29 m | 21ab, 22  21ab, 22 | 18b, 29 | 18  18 |
| 21 | 25.28 CH_2_ | a: 1.99 m  b: 1.83 m | 20ab, 22  20ab, 22 | 20ab |  |
| 22 | 126.39 CH | 4.99 tsept (*6.0, 1.2*) | 20ab, 21ab, 24, 30 | 20ab, 24, 30 | 20ab, 21ab, 24 |
| 23 | 131.95 C |  |  | 24, 30 |  |
| 24 | 25.92 CH_3_ | 1.64 s (3H) | 20ab, 21ab, 22, 24 | 30 | 22, 30 |
| 25 | 23.73 CH_3_ | 1.39 s (3H) |  | 1, 4 | 5b, 26, |
| 26 | 24.91 CH_3_ | 1.20 s (3H) |  | 1, 6 | 5b, 8a, 25, 28 |
| 27 | 24.70 CH_3_ | 0.92 s (3H) |  |  | 9a, 13a, 28 |
| 28 | 20.68 CH_3_ | 0.88 s (3H) |  | 6, 12a | 5b, 2ab, 26, 27 |
| 29 | 24.32 CH_3_ | 0.90 s (3H) |  | 18b, 20ab | 18, 26 |
| 30 | 17.90 CH_3_ | 1.54 s (3H) | 20ab, 21ab, 22, 30 | 24 | 24 |
| 1´ | 152.53 C | ------ |  |  |  |
| *Coupling pattern taken from selective 1D TOCSY (fig S7) | | | | | |

| Table S2. NMR Data for Gromomycin A (2) in DMSO-*d_6_* + 1 drop TFA. | | | | | | |  |
| --- | --- | --- | --- | --- | --- | --- | --- |
| Nr. | δ_C_ | | δ_H_ (*J* in Hz) | HHCOSY | HMBC (C→H) | NOESY Key Correlations |  |
| 1 | 115.07 CH | | 5.86 d (*5.0*) | N_1_-H |  | 8ab, NHa |  |
| 2 | 130.11 C | |  |  | 1, 25, 26, N_1_-H, N_2_-H |  |  |
| 3 | 57.86 C | |  |  | 1, 4, 5ab, 25, N_2_-H |  |  |
| 4 | 78.05 CH | | 3.50 m | 5ab | 5ab, 6, 25 | 6, OH |  |
| 5  5 | 28.00 CH_2_ | | a: 1.60 m  b: 1.46 m | 4,6  4 | 4, 6 | 25, 26, 28 |  |
| 6 | 40.07 CH | | 1.48 m | 5a | 4, 5ab, 12ab, 26 | 4 |  |
| 7 | 37.09 C | |  |  | 1, 5ab, 26 |  |  |
| 8 | 33.87 CH_2_ | | a: 1.39 m  b: 1.02 m | 8b, 9ab  8a, 9ab | 6, 9ab, 26 | 1  1 |  |
| 9  9 | 27.71 CH_2_ | | a: 1.67 m  b: 1.58 m | 9b, 8ab  9a, 8ab | 8a, 27, 28 | 16 |  |
| 10 | 40.22 C | |  |  | 16, 27, 28 |  |  |
| 11 | 36.67 C | |  |  | 6, 9a, 12b, 13b, 27,28 |  |  |
| 12 | 29.92 CH_2_ | | a: 1.49 m  b: 1.38 m | 12b, 13ab  12a, 13ab | 13ab, 28 |  |  |
| 13  13 | 21.58 CH_2_ | | a: 1.98 dd (*19.0, 6.0*)  b: 1.77 m | 13b, 12ab  13a, 12ab | 12ab, 28 |  |  |
| 14 | 136.33 C | |  |  | 12ab, 13ab,16, 17, 18ab, 29 |  |  |
| 15 | 132.20 C | |  |  | 9b, 13ab, 17, 27 |  |  |
| 16 | 123.70 CH | | 5.96 d (*10.0*) | 17, 18ab | 13a, 18ab | 9a, 17, 27 |  |
| 17 | 124.10 CH | | 5.71 dt (*10.0, 4.5*) | 16, 18ab | 18ab | 16, 18ab |  |
| 18  8 | 34.87 CH_2_ | | a: 2.12 dt (*17.0, 2.5*)  b: 1.75 m | 16,17  16,17 | 16, 17, 20ab, 29 | 17,  17 |  |
| 19 | 37.27 C | |  |  | 13b, 17, 21b, 29 |  |  |
| 20 | 38.53 CH_2_ | | a: 1.37 m  b: 1.25 m | 21ab  21ab | 18a, 21ab, 22, 29 |  |  |
| 21 | 23.77 CH_2_ | | a: 1.90 m  b: 1.77 m | 20ab, 22  20ab, 22 | 20ab, 29 |  |  |
| 22 | 125.30 CH | | 4.95 tsept (*6.5, 1.2*) | 21ab, 24, 30 | 20ab, 21ab, 24, 30 | 24 |  |
| 23 | 130.72 C | |  |  | 21, 24, 30 |  |  |
| 24 | | 25.55 CH_3_ | | 1.60 s (3H) | 22 | 22, 30 | 22, 30 |
| 25 | 23.42 CH_3_ | | 1.26 s (3H) |  | 4 | 5b, 26 |  |
| 26 | 24.19 CH_3_ | | 1.10 s (3H) |  | 6 | 5b, 25, 28 |  |
| 27 | 24.26 CH_3_ | | 0.86 s (3H) |  |  |  |  |
| 28 | 20.14 CH_3_ | | 0.80 s (3H) |  | 6, 12a | 5b, 26 |  |
| 29 | 23.79 CH_3_ | | 0.85 s (3H) |  |  |  |  |
| 30 | 17.55 CH_3_ | | 1.48 s (3H) | 22 | 22, 24 | 24 |  |
| 1´ | 150.88 C | |  |  | 1 |  |  |
| OH |  | | 5.43 d (*5.0*) |  |  | 4 |  |
|  |  | |  |  | |  |  |
| Nr. | δ_N_ | | δ_H_ (*J* in Hz) | ^15^N-HMBC (N→H) | | NOESY Key Correlations |  |
| N_1_ | 96.2* |  | 9.15 dd (*5.0, 2.0*) | 1, N_2_-H, | | 1 |  |
| N_2_ | 101.7* |  | 7.92 d (*2.0*) | 25 | |  |  |
| N_3_ | 72.6* |  | 7.04 s (2H) |  | |  |  |

* taken from the ^1^H-^15^N HSQC

| Table S3. NMR Data for Gromomycin B (3) in CD_3_OD. | | | | | | |
| --- | --- | --- | --- | --- | --- | --- |
|  |  |  |  |  |  |  |
| Nr. |  | δ_C_ | δ_H_ (*J* in Hz) | HHCOSY | HMBC (C→H) | NOESY Key Correlations |
| 1 |  | 116.52 CH | 5.89 s |  |  | 8ab |
| 2 |  | 131.98 C |  |  | 1, 25, 26 |  |
| 3 |  | 59.71 C |  |  | 1, 4, 25 |  |
| 4 |  | 79.54 CH | 3.70 dd (*11.8, 4.5*) | 5ab | 5b, 6, 25 | 6 |
| 5 |  | 29.32 CH_2_ | a: 1.86 m  b: 1.63 m | 4, 6  4,6 | 6 |   25. 26, 28 |
| 6 |  | 47.70 CH | 1.39 m | 5ab | 12b, 26, 28 | 4, 14 |
| 7 |  | 38.68 C |  |  | 1, 6, 26, 27, 28 |  |
| 8 |  | 35.16 CH_2_ | a: 1.57 m  b: 1.34 m | 8b, 9ab  8a, 9ab | 26 |  |
| 9 |  | 33.10 CH_2_ | a: 1.99 m  b: 1.87 m | 9b, 8ab  9a, 8ab | 27 |  |
| 10 |  | 45.98 C |  |  | 27, 28 |  |
| 11 |  | 38.68 C |  |  | 6, 26, 27, 28 |  |
| 12 |  | 33.50 CH_2_ | a: 1.75 m  b: 1.51 m | 12b, 13ab  12a, 13ab | 28 |  |
| 13 |  | 19.50 CH_2_ | a: 1.72 m  b: 1.61 m | 12ab, 14  12ab, 14 |  |  |
| 14 |  | 43.74 CH | 2.84 td (*8.5, 2.5*) | 13ab | 12a, 13a, 18b, 29 | 6, 13a, 18a |
| 15 |  | 176.67 C |  |  | 27 |  |
| 16 |  | 126.77 CH | 6.15 d (*2.5*) | 14, 18b | 18b | 9b, 27 |
| 17 |  | 202.84 C |  |  | 16, 18ab |  |
| 18 |  | 50.58 CH_2_ | a: 2.54 d (16.3)  b: 2.11 d (16.3) | 18b, 29  18a | 29 | 14, 18b  18a, 29 |
| 19 |  | 42.57 C |  |  | 18ab, 29 |  |
| 20 |  | 41.07 CH_2_ | a: 1.49 m  b: 1.32 m | 21ab  21ab | 18a, 29 |  |
| 21 |  | 23.57 CH_3_ | a: 2.01 m  b: 1.95 m | 22, 20ab  22, 20ab |  |  |
| 22 |  | 125.44 CH | 5.11 t (7.3) | 21ab, 24, 30 | 24, 30 | 24 |
| 23 |  | 132.47 C |  |  | 24, 30 |  |
| 24 |  | 17.81 CH_3_ | 1.61 s (3H) |  | 30 | 22 |
| 25 |  | 23.66 CH_3_ | 1.41 s (3H) |  |  | 26 |
| 26 |  | 25.01 CH_3_ | 1.24 s (3H) |  |  | 5b, 25; 28 |
| 27 |  | 28.18 CH_3_ | 1.14 s (3H) |  | 28 | 28 |
| 28 |  | 21.95 CH_3_ | 0.94 s (3H) |  | 6 | 5b, 21a, 26, 27, 29 |
| 29 |  | 19.23 CH_3_ | 0.82 s (3H) | 18a | 18a | 12a, 13b, 18b, 20b, 28 |
| 30 |  | 25.89 CH_3_ | 1.67 s (3H) |  | 24 |  |
| 1´ |  | 152.5 C |  |  | 1 |  |

| Table S4. NMR Data for Gromomycin C (4) in DMSO-*d_6._* | | | | | |
| --- | --- | --- | --- | --- | --- |
|  |  |  |  |  |  |
| Nr. | δ_C_ | δ_H_ (*J* in Hz) | HHCOSY | HMBC (C→H) | NOESY Key Correlations |
| 1 | 115.34 CH | 5.82 s |  |  | 8ab |
| 2 | 129.87 C |  |  | 1, 6. 25, 26 |  |
| 3 | 57.66 C |  |  | 1, 4, 5ab, 25 |  |
| 4 | 78.03 CH | 3.55 dd (*11.0, 4.3*) | 5ab, 6 | 5ab, 6, 25 | 6 |
| 5 | 27.85 CH_2_ | a: 1.61 m  b: 1.45 m | 4, 6  4, 6 | 4, 6 |   25, 26, 28 |
| 6 | 45.34 CH | 1.37 m | 4, 5ab | 4, 5ab, 8a, 12ab, 26, 28 | 4 |
| 7 | 36.94 C |  |  | 1, 5ab, 26 |  |
| 8 | 33.58 CH_2_ | a: 1.43 m  b: 1.32 m | 8b, 9ab  8a, 9ab | 6,, 9ab, 26 |  |
| 9 | 32.59 CH_2_ | a: 1.73 m  b: 1.64 m | 9b, 8ab  9a, 8ab | 27 |  |
| 10 | 42.04 C |  |  | 8a, 9b, 12ab, 16, 27, 28 |  |
| 11 | 37.26 C |  |  | 27,28 |  |
| 12 | 32.13 CH_2_ | a: 1.54 m  b: 1.27 m | 12b, 13ab  12a, 13ab | 13ab, 28 |  |
| 13 | 18.76 CH_2_ | a: 1.45 m  b: 1.33 m | 12ab, 14  12ab, 14 | 12ab, 28 |  |
| 14 | 40.02 CH | 2.25 m | 13ab | 12a, 13a, 16, 18b, 20ab | 6, 20ab, 21ab |
| 15 | 143.48 C |  |  | 9ab, 13a, 17, 27 |  |
| 16 | 120.27 CH | 5.60 q (*2.5*) | 13ab, 14, 17ab | 14, 17, 18ab | 9ab,17ab, 27 |
| 17 | 22.98 CH_2_ | 2.04 m (2H) | 14, 18ab | 16, 18ab |  |
| 18 | 32.77 CH_2_ | a: 1.39 m  b: 1.33 m | 17ab  17ab | 16, 17ab, 20ab, 29 |  |
| 19 | 34.80 C |  |  | 13b, 20ab, 21ab, 29 |  |
| 20 | 40.61 CH_2_ | 1.22 m (2H) | 21ab | 21, 22, 29 |  |
| 21 | 21.72 CH_2_ | 1.85 q (*7.5*, 2H) | 20ab, 22 |  |  |
| 22 | 125.10 CH | 5.07 t (*7.5*) | 21ab, 24, 30 | 20ab 21ab, 24, 30 | 24, 21ab |
| 23 | 130.21 C |  |  | 21ab, 24, 30 |  |
| 24 | 17.44 CH_3_ | 1.53 br s (3H) | 22, 30 | 22,30 | 21ab, 30 |
| 25 | 23.31 CH_3_ | 1.25 s (3H) |  | 1, 4 | 5b, 26, |
| 26 | 24.36 CH_3_ | 1.10 s (3H) |  | 1, 6 | 5b, 8a, 9a, 25, 28 |
| 27 | 21.64 CH_3_ | 0.76 s (3H) |  | 9ab, 16 | 12ab, 28, |
| 28 | 29.14 CH_3_ | 0.96 s (3H) |  |  | 5b, 9ab, 26, 27 |
| 29 | 18.04 CH_3_ | 0.68 s (3H) |  | 14, 18ab | 13ab, 20ab, 18b, 21ab |
| 30 | 25.50 CH_3_ | 1.62 br s (3H) |  | 22, 24 | 24 |
| 1´ | 151.71* C |  |  | 1 |  |
| OH |  | 5.47 br s |  |  |  |
| N_1_-H |  | 11.30 br s |  |  |  |
| N_2_-H |  | 8.74 br s |  |  |  |
| N_3_-H |  | 8.06 br s |  |  |  |

* taken from the ^1^H-^13^C HMBC

| Table S5. NMR Data for Gromomycin D (5) in CD_3_OD. | | | | |
| --- | --- | --- | --- | --- |
|  |  |  |  |  |
| Nr. | δ_C_ | δ_H_ (*J* in Hz) | HHCOSY | HMBC (C→H) |
| 1 | 116.25 CH | 5.91 s |  |  |
| 2 | 132.15 C |  |  | 1, 25, 26 |
| 3 | 59.73 C |  |  | 1, 4, 5a, 25 |
| 4 | 79.65 CH | 3.68 m | 5ab | 5ab, 25 |
| 5 | 29.01 CH_2_ | a: 1.76 m  b: 1.61 m | 4  4 | 4, 6 |
| 6 | 41.94 CH | 1.64 m |  | 5ab, 26, 28 |
| 7 | 38.33 C |  |  | 1, 8a, 9a, 26 |
| 8 | 35.58 CH_2_ | a: 1.59 m  b: 1.20 m |  | 26 |
| 9 | 28.33 CH_2_ | a: 1.69 m  b: 1.58 m |  | 27 |
| 10 | 42.94 C |  |  | 6, 8a, 12ab, 27, 28 |
| 11 | 38.64 C |  |  | 27, 28 |
| 12 | 30.85 CH_2_ | a: 1.65 m  b: 1.56 m |  | 28 |
| 13 | 23.18 CH_2_ | a: 2.09 m  b: 1.84 m |  |  |
| 14 | 137.10 C |  |  | 12a, 18ab, 20b, 29 |
| 15 | 133.23 C |  |  | 18b, 27 |
| 16 | 41.14 CH_2_ | a: 2.94 dd (*20.0, 2.5*)  b: 2.80 br d (*20.0*) | 13a, 16ab, 18a  16a, 18b | 18ab |
| 17 | 214.05 C |  |  | 18ab |
| 18 | 52.00 CH_2_ | a: 2.63 d (*13.5*)  b: 2.10 dd (*13.5,1.0*) | 16a, 18b,  18ab,16b, | 29 |
| 19 | 44.60 C |  |  | 18ab, 29 |
| 20 | 41.32 CH_2_ | a: 1.58 m  b: 1.22 m | 20b, 21ab  20a, 21ab | 18ab, 29 |
| 21 | 25.22 CH_2_ | a: 1.98 m  b: 1.79 m | 20ab, 21b  20ab, 21a |  |
| 22 | 125.35 CH | 4.99 t (*6.0*) | 21ab, 24, 30 | 20b, 21a, 24, 30 |
| 23 | 132.76 C |  |  | 24, 30 |
| 24 | 17.98 CH_3_ | 1.54 s (3H) |  | 22, 30 |
| 25 | 23.72 CH_3_ | 1.40 s (3H) |  | 4 |
| 26 | 24.97 CH_3_ | 1.22 s (3H) |  |  |
| 27 | 23.72 CH_3_ | 0.95 s (3H) |  |  |
| 28 | 20.94 CH_3_ | 0.90 s (3H) |  | 6 |
| 29 | 27.52 CH_3_ | 1.00 s (3H) |  | 18ab |
| 30 | 25.87 CH_3_ | 1.64 s (3H) |  | 22 |
| 1´ | 152.50 C |  |  | 1 |

| Table S6. NMR Data for Gromomycin E (6) in CD_3_OD. | | | | |
| --- | --- | --- | --- | --- |
|  |  |  |  |  |
| Nr. | δ_C_ | δ_H_ (*J* in Hz) | HHCOSY | HMBC (C→H) |
| 1 | 116.18 CH | 5.85 s |  |  |
| 2 | 132.30 C |  |  | 1, 6, 25,26 |
| 3 | 59.78 C |  |  | 1, 4, 5ab, 25 |
| 4 | 79.72 CH | 3.71 dd (*11.0, 4.5*) | 5ab | 5ab, 6, 25 |
| 5 | 29.16 CH_2_ | a: 1.74 m  b: 1.61 m | 4  4 | 4, 6 |
| 6 | 41.77 CH | 1.63 m |  | 26, 28 |
| 7 | 38.57 C |  |  | 1, 26 |
| 8 | 35.26 CH_2_ | a: 1.47 m  b: 1.21 m | 8b, 9ab  8a, 9ab |  |
| 9 | 29.01 CH_2_ | a: 1.73 m  b: 1.63 m | 9b, 8ab  9a, 8ab | 6, 27 |
| 10 | 41.66 C |  |  | 8b, 9ab, 27, 28 |
| 11 | 38.12 C |  |  | 27 |
| 12 | 31.26 CH_2_ | a: 1.61 m  b: 1.50 m | 12b, 13ab  12a, 13ab |  |
| 13 | 22.59 CH_2_ | a: 2.04 m  b: 1.86 m | 13b, 12b  13a, 12ab | 12ab |
| 14 | 138.00 C |  |  | 12a, 13ab, 16, 17, 18ab, 29 |
| 15 | 133.60 C |  |  | 13ab, 16, 17, 18ab, 27 |
| 16 | 124.76 CH | 5.99 d (*9.5*) | 17, 18ab | 18ab |
| 17 | 125.32 CH | 5.73 dt (*9.5, 4.5*) | 16, 18ab | 18ab |
| 18 | 35.75 CH_2_ | a: 2.21 dd (*17.0, 5.0*)  b: 1.77 d (*17.0*) | 16, 17, 18b,  16, 17, 18a | 16, 17, 20ab |
| 19 | 38.23 C | -------- |  | 18ab |
| 20 | 37.35 CH_2_ | a: 1.38 m  b: 1.12 m |  | 18b, 21ab, 22 |
| 21 | 31.89 CH_2_ | a: 1.38 m  b: 1.11 m | 22  22 | 20ab, 22, 31 |
| 22 | 43.58 CH | 2.01 m | 21ab, 31 | 21ab, 24ab, 30, 31 |
| 23 | 150.97 C |  |  | 22, 24ab, 30, 31 |
| 24 | 110.20 CH_2_ | 4.63 s  4.60 s | 24b  24a | 22, 30 |
| 25 | 23.96 CH_3_ | 1.40 s (3H) |  | 3 |
| 26 | 24.91 CH_3_ | 1.20 s (3H) |  | 6 |
| 27 | 24.82 CH_3_ | 0.91 s (3H) |  |  |
| 28 | 20.75 CH_3_ | 0.87 s (3H) |  |  |
| 29 | 20.75 CH_3_ | 0.87 s (3H) |  |  |
| 30 | 18.98 CH_3_ | 1.59 s (3H) |  | 24ab |
| 31 | 20.47 CH_3_ | 0.98 d (7.0, 3H) | 22 | 21ab, 22 |
| 1´ | 152.57 C |  |  | 1 |

| Table S7. NMR Data for Gromomycin F (7) in CD_3_OD. | | | | |  |
| --- | --- | --- | --- | --- | --- |
|  |  |  |  |  |  |
| Nr. | δ_C_ | δ_H_ (*J* in Hz) | HHCOSY | HMBC (C→H) | NOESY Key Correlations |
| 1 | 116.58 CH | 5.89 s |  |  | 8ab |
| 2 | 131.93 C |  |  | 1, 25, 26 |  |
| 3 | 59.73 C |  |  | 1, 4, 5a, 25 |  |
| 4 | 79.60 CH | 3.70 dd (*12.0, 4.3*) | 5ab | 5ab, 6, 25 | 6 |
| 5 | 29.32 CH_2_ | a: 1.86 m  b: 1.64 m | 4, 6  4, 6 | 6 | 25, 26, 28 |
| 6 | 47.76 CH | 1.39 m | 5ab | 12b, 26, 28 | 4, 5a, 14, 26 |
| 7 | 38.71 C |  |  | 1, 6, 26, 27, 28 |  |
| 8 | 35.15 CH_2_ | a: 1.56 m  b: 1.34 m | 8b, 9ab  8a, 9ab | 26 |  |
| 9 | 33.08 CH_2_ | a: 1.97 m  b: 1.86 m | 9b, 8ab  9a, 8ab | 27 |  |
| 10 | 45.97 C |  |  | 12a, 16, 27, 28 |  |
| 11 | 38.70 C |  |  | 6, 26, 27, 28 |  |
| 12 | 33.54 CH_2_ | a: 1.75 m  b: 1.51 m | 12b, 13ab  12a, 13ab | 13ab, 28 |  |
| 13 | 19.50 CH_2_ | a: 1.68 m  b: 1.59 m | 12ab, 13b, 14  12ab, 13a, 14 |  | 13b, 20a, 21b  13a |
| 14 | 44.13 CH | 2.81 td (*8.5, 2.7*) | 13ab, 14 | 12ab, 13ab, 16,18b, 29 | 6, 13a, 18a |
| 15 | 176.64 C |  |  | 9a, 13a, 14, 27 |  |
| 16 | 121.57 CH | 6.14 d (*2.5*) | 14, 18a |  | 9a, 27 |
| 17 | 202.85 C |  |  | 18ab |  |
| 18 | 50.58 CH_2_ | a: 2.46 d (*16.5*)  b: 2.08 d (*16.5*) | 18b, 29  18a | 16, 29 | 14, 18b, 20  18a, 20a |
| 19 | 42.32 C |  |  | 14, 18ab, 29 |  |
| 20 | 39.07 CH_2_ | a: 1.38 m  b: 1.12 m |  | 18ab, 29 | 18a, 2 |
| 21 | 29.75 CH_2_ | a: 1.38 m  b: 1.24 m |  | 20a, 22, 31 |  |
| 22 | 43.00 CH | 2.10 m | 21ab, 31 | 24ab, 30, 31 | 24b, 31 |
| 23 | 150.83 C |  |  | 23, 31 |  |
| 24 | 110.53 CH_2_ | a: 4.69 br s  b: 4.67 br s | 30  30 | 31 | 30  22 |
| 25 | 23.65 CH_3_ | 1.41 s (3H) |  | 4 | 5b, 26 |
| 26 | 25.04 CH_3_ | 1.23 s (3H) |  | 6 | 5b, 25, 28 |
| 27 | 28.18 CH_3_ | 1.13 s (3H) |  | 12b | 12b, 28 |
| 28 | 21.94 CH_3_ | 0.94 s (3H) |  | 6 | 27, 29 |
| 29 | 19.01 CH_3_ | 0.80 s (3H) | 18a | 18ab | 13b, 18b, 20b, 28 |
| 30 | 18.92 CH_3_ | 1.64 s (3H) | 24ab | 24ab | 24a |
| 31 | 20.43 CH_3_ | 1.03 d (*7*, 3H) | 22 |  | 22 |
| 1´ | 152.65 C |  |  |  |  |

| Table S8. NMR Data for Gromomycin G (8) in CD_3_OD. | | | | |
| --- | --- | --- | --- | --- |
|  |  |  |  |  |
| Nr. | δ_C_ | δ_H_ (*J* in Hz) | HHCOSY | HMBC (C→H) |
| 1 | 116.19 CH | 5.91 s |  |  |
| 2 | 132.70 C |  |  | 1, 25, 26 |
| 3 | 59.80 C |  |  | 1, 4, 5a, 25 |
| 4 | 80.03 CH | 3.65 m | 5ab, 6 | 5a, 6, 25 |
| 5 | 29.47 CH_2_ | a: 1.85 m  b: 1.59 m | 4, 5b, 6  4, 5a, 6 | 6 |
| 6 | 47.13 CH | 1.57 m | 4, 5ab | 5ab, 12b, 26, 28 |
| 7 | 38.73 C |  | 8 | 1, 5a, 6, 26 |
| 8 | 35.06 CH_2_ | 1.49 m (2H) | 7 | 26 |
| 9 | 34.15 CH_2_ | a: 1.82 m  b: 1.73 m | 8, 9b  8, 9a | 27 |
| 10 | 43.54 C |  |  | 27, 28 |
| 11 | 39.02 C |  |  | 27, 28 |
| 12 | 33.38 CH_2_ | a: 1.65 m  b: 1.38 m | 13a | 28 |
| 13 | 20.15 CH_2_ | a: 1.50 m  b: 1.43 m | 12a,  14 | 12ab |
| 14 | 41.78 CH | 2.32 m | 13ab | 13ab |
| 15 | 145.00 C |  |  |  |
| 16 | 122.41 CH | 5.65 m | 17, 18b |  |
| 17 | 24.44 CH_2_ | 2.08 m (2H) | 16, 18a |  |
| 18 | 34.57 CH_2_ | a: 1.41 m  b: 1.34 m | 17  16 |  |
| 19 | 35.97 C |  |  | 29 |
| 20 | 39.87 CH_2_ | 1.19 m (2H) |  | 20 |
| 21 | 29.67 CH_2_ | a: 1.31 m  b: 1.18 m | 21b  21a |  |
| 22 | 43.23 CH | 2.04 m | 31 | 24ab, 30, 31 |
| 23 | 151.28 C |  |  | 30, 31 |
| 24 | 110.12 CH_2_ | a: 4.66 br s  b: 4.65 br s | 24b  24a |  |
| 25 | 23.66 CH_3_ | 1.40 s (3H) |  | 6 |
| 26 | 25.07 CH_3_ | 1.20 s (3H) |  |  |
| 27 | 29.56 CH_3_ | 1.02 s (3H) |  |  |
| 28 | 22.28 CH_3_ | 0.84 s (3H) |  | 6 |
| 29 | 18.88 CH_3_ | 0.73 s (3H) |  |  |
| 30 | 18.96 CH_3_ | 1.63 s (3H) |  | 22, 24a |
| 31 | 20.44 CH_3_ | 0.99 d (*7,* 3H) | 22 | 22 |
| 1´ | 152.67 C |  |  | 1 |

| Table S9. NMR Data for Gromomycin H (9) in CD_3_OD. | | | | |
| --- | --- | --- | --- | --- |
|  |  |  |  |  |
| Nr. | δ_C_ | δ_H_ (*J* in Hz) | HHCOSY | HMBC (C→H) |
| 1 | 116.27 CH | 5.92 s |  |  |
| 2 | 132.16 C |  |  | 1, 25, 26 |
| 3 | 59.77 C |  |  | 1, 4, 5a, 25 |
| 4 | 79.65 CH | 3.73 dd (*11.5, 4.3*) | 5ab, 6 | 5ab, 25 |
| 5 | 29.03 CH_2_ | 1.77 m  1.63 m | 4, 5a, 6  4, 5b | 6 |
| 6 | 42.13 CH | 1.59 m | 4, 5a, 6 | 5ab, 26, 28 |
| 7 | 38.38 C |  |  | 1, 26 |
| 8 | 35.57 CH_2_ | 1.60 m  1.19 m | 9a  9a | 9a, 26 |
| 9 | 28.32 CH_2_ | 1.70 m  1.58 m | 8ab | 27 |
| 10 | 42.93 C |  |  | 12b,27, 28 |
| 11 | 38.65 C |  |  | 27, 28 |
| 12 | 30.87 CH_2_ | 1.68 m  1.58 m | 13a | 28 |
| 13 | 22.90 CH_2_ | 2.09 m  1.82 m | 12b  16a | 12ab, 28 |
| 14 | 137.12 C |  |  | 12a, 18ab, 29 |
| 15 | 132.88 C |  |  | 9a, 16ab, 27 |
| 16 | 41.16 CH_2_ | 2.91 br d (*20.0*)  2.80 br d (*20.0*) | 13b, 16b, 18b  16a | 18b, 27 |
| 17 | 214.11 C |  |  | 16ab, 18ab |
| 18 | 51.80 CH_2_ | 2.61 d (*13.5*)  2.07 dd (*13.5, 1.0*) | 18b, 29  18a | 20a, 29 |
| 19 | 43.24 C |  |  |  |
| 20 | 39.42 CH_2_ | 1.45 m  1.11 m | 21a | 18ab, 29 |
| 21 | 31.62 CH_2_ | 1.32 m  1.10 m | 20a, 22  22 | 22, 31 |
| 22 | 43.25 CH | 2.07 m | 21ab, 31 | 24ab, 30, 31 |
| 23 | 150.54 C |  |  | 30, 31 |
| 24 | 110.51 CH_2_ | 4.65 br s  4.62 br s | 30  30 | 21. 30 |
| 25 | 23.79 CH_3_ | 1.41 s (3H) |  |  |
| 26 | 25.03 CH_3_ | 1.23 s (3H) |  | 6 |
| 27 | 23.86 CH_3_ | 0.95 s (3H) |  |  |
| 28 | 21.07 CH_3_ | 0.90 s (3H) |  | 6, 12ab |
| 29 | 27.90 CH_3_ | 0.99 s (3H) | 18a | 18ab |
| 30 | 18.91 CH_3_ | 1.59 s (3H) | 24ab | 24ab |
| 31 | 20.62 CH_3_ | 0.98 d (*7.0*, 3H) | 22 | 22 |
| 1´ | 152.54 C | ----- |  | 1 |

| Table S10. NMR Data for Hexaprenylguanidine (10) in DMSO-*d_6._* | | | | |
| --- | --- | --- | --- | --- |
|  |  |  |  |  |
| Nr. | δ_C_ | δ_H_ (*J* in Hz) | HHCOSY | HMBC (C→H) |
| 1 | 38.62 CH_2_ | 3.66 t* (*5.7,* 2H) | H-N_a_, H-2 |  |
| 2 | 119.17 CH | 5.19 t (*6.5*) | H-1, H-25, | H-1, H-4, H-25 |
| 3 | 138.91 C |  |  | H-1, H-4, H-5, H-25 |
| 4 | 38.97 CH_2_ | 1.99 m (2H) |  |  |
| 5 | 26.21 CH_2_ | 2.05 m (2H) |  |  |
| 6 | 124.13 CH | 5.06 m |  |  |
| 7 | 134.67 C |  |  |  |
| 8 | 39.00 CH_2_ | 1.93 m (2H) |  |  |
| 9 | 26.08 CH_2_ | 2.02 m (2H) |  |  |
| 10 | 124.00 CH | 5.06 m |  |  |
| 11 | 134.30 C | ----- |  |  |
| 12 | 39.00 CH_2_ | 1.93 m (2H) |  |  |
| 13 | 26.04 CH_2_ | 2.02 m (2H) |  |  |
| 14 | 123.97 CH | 5.06 m |  |  |
| 15 | 134.30 C |  |  |  |
| 16 | 39.00CH_2_ | 1.93 m (2H) |  |  |
| 17 | 26.00 CH_2_ | 2.02 m (2H) |  |  |
| 18 | 123.92 CH | 5.06 m |  |  |
| 19 | 134.24 C |  |  |  |
| 20 | 39.00 CH_2_ | 1.93 m (2H) |  |  |
| 21 | 25.94 CH_2_ | 2.02 m (2H) |  |  |
| 22 | 123.67 CH | 5.06 m |  |  |
| 23 | 130.65 C |  |  |  |
| 24 | 25.53 CH_3_ | 1.63s (3H) |  |  |
| 25 | 16.17 CH_3_ | 1.63 s (3H) | H-1, H-2 | H-2, H-4 |
| 26 | 15.80 CH_3_ | 1.55 s (3H) |  |  |
| 27 | 15.80 CH_3_ | 1.55 s (3H) |  |  |
| 28 | 15.80 CH_3_ | 1.55 s (3H) |  |  |
| 29 | 15.80 CH_3_ | 1.55 s (3H) |  |  |
| 30 | 17.57 CH_3_ | 1.55 s (3H) |  |  |
| 1´ | 157.29 C |  |  | H-1 |
|  | δ_N_ |  |  |  |
| N_a_ | 85.5** | 8.61 t (5.0) | H-1 |  |
| N_b,c_ | 75.5** | 7.79 br s (3H) |  |  |

*t= pseudo triplet; formed by two doublets with J_HNa,H-1_ =5.0 Hz and J_H-1,H-2_ =6.5 Hz

**= taken from the ^15^N HSQC

## Table S11. ^1^H NMR (CDCl_3_) data of S-MTPA ester (5a) and R-MTPA ester (5b) derived from Gromomycin B (3) and their Δδ_S-R_ values.

| **5a**  δ_H_ (*J* in Hz) | | **5b**  δ_H_ (*J* in Hz) | Δδ_S-R_  (δS -δR) |
| --- | --- | --- | --- |
| 1 | 5.93 s | 5.91 s | +0.02 |
| 4 | 4.83 dd (*11.8, 4.4*) | 4.84 dd (*11.8, 4.4*) | - 0.01 |
| 5 | 1.93 m  1.69 m | 2.03 m  1.71 m | - 0.10  - 0.02 |
| 6 | 1.69 d (*12.5*) | 1.73 d (*12.5*) | - 0.04 |
| 8 | 1.54 m  1.16 m | 1.54 m  1.16 m | 0.00  0.00 |
| 9 | 1.57 m (2H) | 1.57 m (2H) | 0.00 |
| 12 | 1.50 m (2H) | 1.53 m (2H) | - 0.03 |
| 13 | 2.06 m  1.85 m | 2.06 m  1.89 m | 0.00  - 0.04 |
| 16 | 2.83 d (*20.0*)  2.75 d (*20.0*) | 2.83 d (*20.0*)  2.75 d (*20.0*) | 0.00  0.00 |
| 18 | 2.65 d (*13.0*)  2.10 d (*13.0*) | 2.66 d (*13.0*)  2.10 d (*13.0*) | - 0.01  0.00 |
| 20 | 1.63 m  1.25 m | 1.63 m  1.25 m | 0.00  0.00 |
| 21 | 2.03 m  1.56 m | 2.03 m  1.63 m | 0.00  - 0.07 |
| 22 | 5.05 t (*6.3*) | 5.09 t (*6.3*) | - 0.04 |
| 24 | 1.53 s (3H) | 1.54 s (3H) | - 0.01 |
| 25 | 1.36 s (3H) | 1.35 s (3H) | +0.01 |
| 26 | 1.15 s (3H) | 1.17 s (3H) | - 0.02 |
| 27 | 0.82 s (3H) | 0.85 s (3H) | - 0.03 |
| 28 | 0.99 s (3H) | 0.99 s (3H) | 0.00 |
| 29 | 0.91 s (3H) | 0.92 s (3H) | - 0.01 |
| 30 | 1.64 s (3H) | 1.65 s (3H) | - 0.01 |

| Table S12. Incorporation of [2-13C]pyruvate in gromomycin B (3). | |
| --- | --- |
|  | |
| Nr. | Isotopic enrichment in % |
| 3 | 8 |
| 7 | 8 |
| 11 | 8 |
| 15 | 7 |
| 19 | 11 |
| 23 | 8 |

All positions which are not mentioned in this table were not labelled, with the exception of C-20,

which showed an moderate enrichment of 3%

| Table S13. Incorporation of [3-13C]pyruvate in gromomycin B (3). | |
| --- | --- |
|  | |
| Nr. | Isotopic enrichment in % |
| 25 | 5 |
| 26 | 5 |
| 27 | 6 |
| 28 | 6 |
| 29 | 10 |
| 30 | 7 |

All positions which are not mentioned in this table were not labelled

## Table S14. Bacterial strains used in this work.

| **Strains** | **Description** | **Reference or Source** |
| --- | --- | --- |
| *Streptomyces* sp. Je 1-332 | Gromomycin producing wild-type strain | This work |
| *S. flavoviridis* NRRL ISP-5153 | Type strain | Agricultural Research Service Culture Collection (NRRL) |
| *S. albus* Del14 | Cluster-free heterologous host strain | ^[2]^ |
| *S. lividans* Del8 | Cluster-free heterologous host strain | ^[3]^ |
| *Streptomyces* sp. Je 1-332_Tn5_mut_148 | Tn5 transposon mutant of *Streptomyces* sp. Je 1-332, no gromomycin producing strain | This work |
| *Streptomyces* sp. Je 1-332_Tn5_mut_355 | Tn5 transposon mutant of *Streptomyces* sp. Je 1-332, no gromomycin producing strain | This work |
| *Streptomyces* sp. Je 1-332del_ fdps | Strain *Streptomyces* sp. Je 1-332 with the deletion of the farnesyl diphosphate synthase gene | This work |
| *Streptomyces* sp. Je 1-332del_ mppO | Strain *Streptomyces* sp. Je 1-332 with the deletion of the putative enduracidin beta-hydroxylase gene | This work |
| *Streptomyces* sp. Je 1-332del_hypprot | Strain *Streptomyces* sp. Je 1-332 with the deletion of the hypothetical protein gene downstream to mppO gene | This work |
| *Streptomyces* sp. Je 1-332del_PAP2-hypprot-P450 | Strain *Streptomyces* sp. Je 1-332 with the deletion of the three genes (a PAP2 superfamily protein, hypothetical protein, and a cytochrome P450) | This work |
| *Streptomyces* sp. Je 1-332del_PrnD3-2 | Strain *Streptomyces* sp. Je 1-332 with the deletion of the two genes annotated as putative aminopyrrolnitrin oxygenases PrnD | This work |
| *S. albus* STS10 | S*. albus* strain Del14 containing the pHSU-STS10 vector | This work |
| *S. lividans* STS10 | *S. lividans* Del8 containing the pHSU-STS10 vector | This work |
| *S. albus* STS-22 | S*. albus* strain Del14 containing the pHSU-STS22 vector | This work |
| *S. albus* STS-23 | S*. albus* strain Del14 containing the pHSU-STS23 vector | This work |
| *S. albus* STS-24 | S*. albus* strain Del14 containing the pHSU-STS24 vector | This work |
| *S. albus* STS-25 | S*. albus* strain Del14 containing the pHSU-STS25 vector | This work |
| *S. albus* STS-29 | S*. albus* strain Del14 containing the pHSU-STS29 vector | This work |
| *S. albus* STS-30 | S*. albus* strain Del14 containing the pHSU-STS30 vector | This work |
| *S. albus* STS-31 | S*. albus* strain Del14 containing the pHSU-STS31 vector | This work |
| *S. albus* STS-32 | S*. albus* strain Del14 containing the pHSU-STS32 vector | This work |
| *S. albus* STS-33 | S*. albus* strain Del14 containing the pHSU-STS33 vector | This work |
| *S. albus* STS-33delgroA | S*. albus* strain Del14 containing the pHSU-STS33_KOgroA vector | This work |
| *S. albus* STS-33delgroB | S*. albus* strain Del14 containing the pHSU-STS33_KOgroB vector | This work |
| *S. albus* STS-33delgroC | S*. albus* strain Del14 containing the pHSU-STS33_KOgroC vector | This work |
| *S. albus* STS-33delgroD | S*. albus* strain Del14 containing the pHSU-STS33_KOgroD vector | This work |
| *S. albus* STS-33delgroE | S*. albus* strain Del14 containing the pHSU-STS33_KOgroE vector | This work |
| *S. albus* STS-33delgroF | S*. albus* strain Del14 containing the pHSU-STS33_KOgroF vector | This work |
| *S. albus* STS-33delgroG | S*. albus* strain Del14 containing the pHSU-STS33_KOgroG vector | This work |
| *S. albus* STS-33delgroH | S*. albus* strain Del14 containing the pHSU-STS33_KOgroH vector | This work |
| *S. albus* STS-33delgroI | S*. albus* strain Del14 containing the pHSU-STS33_KOgroI vector | This work |
| *E. coli* ET12567 pUB307 | Donor strain for intergeneric conjugation | ^[16]^ |
| *E. coli* GB05-red | Strain used for Red/ET | ^[17]^ |
| *E. coli* TransforMax EC100D pir-116 | Strain used for high-copy propagation of rescue plasmid copies | Lucigen |
| *E.coli* EPI300-T1R | Strain used for Construction of inducible-copy-number genomic libraries using the CopyControl™ Cloning System, with clones that are resistant to contaminating phage T1 and T5 | Lucigen |

## Table S15. Plasmids used in this work.

| **Plasmids** | **Description** | **Reference or Source** |
| --- | --- | --- |
| pTNM | *ts-*orip^SG5^ *aac(3)IV* (Amr) *hygB* (Hyr), carries synthetic gene *tnp(a)* of Tn5 transposase, apramycin resistance *gene aac(3)IV* flanked with mosaic ends for Tnp(a) | ^[9]^ |
| pCos15A_gusA | Cloning vector | Lucigen |
| pCos15A_gus_AmInt | pCos15A_gusA, which contains the *oriT* and integrase genes | This work |
| pCos15A_AmInt | pCos15A_gus_AmInt, where *gus* gene was deleted | This work |
| patt-saac-oriT | Resistance cassette plasmid containing a synthetic fragment with *aac(3)IV*, *oriT*, B-CC, P-GG and loxP sites | ^[18]^ |
| phygattB | Resistance cassette plasmid containing hygromycin resistance marker flanked with MssI restriction sites and attB sequence | ^[2]^ |
| pHSU-STS2 | Cosmid P05_A07 containing gene cluster (antismash Region 14) in which T-muurolol synthase gene is replaced by *aac(3)IV* and *oriT* | This work |
| pHSU-STS3 | Cosmid P10_C04 containing gene cluster (antismash Region 15) in which Squalene-hopene cyclase gene is replaced by aac(3)IV and oriT | This work |
| pHSU-STS6 | Cosmid P05_A10 in which farnesyl diphosphate gene is replaced by *aac(3)IV* and *oriT* | This work |
| pSTS-del-mppO | Cosmid P05_A10 in which the putative enduracidin beta-hydroxylase gene is replaced by *aac(3)IV* and *oriT* | This work |
| pSTS-del-hypprot | Cosmid P05_A10 in which the hypothetical protein gene is replaced by *aac(3)IV* and *oriT* | This work |
| pSTS-del-PAP2-hypprot-P450 | Cosmid P05_A10 in which the PAP2 superfamily protein, hypothetical protein, and a cytochrome P450 genes are replaced by *aac(3)IV* and *oriT* | This work |
| pSTS-del-PrnD3-2 | Cosmid P05_A10 in which putative aminopyrrolnitrin oxygenases (PrnD) genes are replaced by *aac(3)IV* and *oriT* | This work |
| pHSU-STS10 | Cosmid P04_E01 containing putative gromomycin gene cluster with *aac(3)IV*, *oriT*, and integrase | This work |
| pHSU-STS22 | The construct based on the pHSU-STS10 plasmid for deletion 1 and 2 genes upstream to putative gromomycin gene cluster | This work |
| pHSU-STS23 | The construct based on the pHSU-STS10 plasmid for deletion 26 – 33 genes downstream to putative gromomycin gene cluster | This work |
| pHSU-STS24 | The construct based on the pHSU-STS10 plasmid for deletion 22 – 33 genes downstream to putative gromomycin gene cluster | This work |
| pHSU-STS25 | The construct based on the pHSU-STS10 plasmid for deletion 18 – 33 genes downstream to putative gromomycin gene cluster | This work |
| pHSU-STS29 | The construct based on the pHSU-STS10 plasmid for deletion 1-5 genes upstream to putative gromomycin gene cluster | This work |
| pHSU-STS30 | The construct based on the pHSU-STS10 plasmid for deletion 1-6 genes upstream to putative gromomycin gene cluster | This work |
| pHSU-STS31 | The construct based on the pHSU-STS25 plasmid for deletion 17 gene downstream to putative gromomycin gene cluster | This work |
| pHSU-STS32 | The construct based on the pHSU-STS25 plasmid for deletion 16 and 17 genes downstream to putative gromomycin gene cluster | This work |
| pHSU-STS33 | The construct based on the pHSU-STS25 plasmid for deletion 15 – 17 genes downstream to putative gromomycin gene cluster | This work |
| pHSU-STS33_KOgroA | The construct based on the pHSU-STS33 plasmid for deletion *groA* gene | This work |
| pHSU-STS33_KOgroB | The construct based on the pHSU-STS33 plasmid for deletion *groB* gene | This work |
| pHSU-STS33_KOgroC | The construct based on the pHSU-STS33 plasmid for deletion *groC* gene | This work |
| pHSU-STS33_KOgroD | The construct based on the pHSU-STS33 plasmid for deletion *groD* gene | This work |
| pHSU-STS33_KOgroE | The construct based on the pHSU-STS33 plasmid for deletion *groE* gene | This work |
| pHSU-STS33_KOgroF | The construct based on the pHSU-STS33 plasmid for deletion *groF* gene | This work |
| pHSU-STS33_KOgroG | The construct based on the pHSU-STS33 plasmid for deletion *groG* gene | This work |
| pHSU-STS33_KOgroH | The construct based on the pHSU-STS33 plasmid for deletion *groH* gene | This work |
| pHSU-STS33_KOgroI | The construct based on the pHSU-STS33 plasmid for deletion *groI* gene | This work |

## Table S16. Primers used in this work.

| **Primer name** | **Sequence (5’-3’)** | **Description** | **Source** |
| --- | --- | --- | --- |
| pMODseq-f  pMODseq-r | GCCAACGACTACGCACTAGCCAAC  GAGCCAATATGCGAGAACACCCGAGAA | For Tn5 insertion loci identification | ^[2]^ |
| OTC60  OTC61 | TGCTTAATGAATTACAACAGTACTGCGATGAGTGGCAGGGCGGGGCGTAAGGTTCATGTGCAGCTCCA  CTGTCGTTTCCTTTCTCTGTTTTTGTCCGTGGAATGAACAATGGAAGTCCCTACGCCGCTACGTCTTC | Amplification of apramycin-oriT-Integrase cassettes from pCos15A_gus_AmInt | This work |
| STS3  STS4 | CGTGACTCATCCCTCATTCCTCATCCTTGCGCCGGAAGGTGTGTGCCGCCTTCCGGGGATCCGTCGACCC  GGGCGAGGACGGCCTCTGGTGGGCGGTGGATCTGGCCGCGCCCCGATGAGTGTAGGCTGGAGCTGCTTCG | For T-muurolol synthase deletion (antismash Region 14) | This work |
| STS13  STS14 | GGCCGACGGCGGACCCACAGCAGAGAAGACGACTGCACGAAGGGGAAGCCTTCCGGGGATCCGTCGACCC  GCAGGCGATCAGCAGCGGCGCGGGGCCCGGCACGGCCGGGGTCCTCTCCATGTAGGCTGGAGCTGCTTCG | For squalene-hopene cyclase deletion (antismash Region 15) | This work |
| STS21  STS22 | AGCCGAGGCGGGGACGGTGTGCACGTCCTCCTGGGAGCCGTCCGCGCCGATTCCGGGGATCCGTCGACCC  TGTCTTTCGCGATTGACAACACCCAAGCAGAAACAGACTTTCCGGTTCCGTGTAGGCTGGAGCTGCTTCG | For farnesyl diphosphate deletion | This work |
| Fdps_del_chk_F  Fdps_del_chk_R | GTTCCAGGCGCCCATCGCAT  ATCCGCACCGTCCAGGCTTG | To test for farnesyl diphosphate and *groE* genes deletion | This work |
| Del_mppO_F  Del_mppO_R | GAGACTGCAGAGATGACGACAGGTGGATCGCGGTGCGTCAGTGGATCCGGTGTAGGCTGGAGCTGCTTCG ACTGCTCTTCGCGGCTTCGATCTTGTCCGACCAACCACGGGGGACTCGCATTCCGGGGATCCGTCGACCC | For putative enduracidin beta-hydroxylase gene deletion | This work |
| mppO_del_chk_F  mppO_del_chk_R | GCTCTTGATCGCGGCATA  GTCTCTAATTCGTCGCCG | To test for mppO and *groD* genes deletion | This work |
| Del_hupprot_F  Del_hupprot_R | ACGGGTCTGACGCACTGTGCGGGGCTCGGCTCTAGGAGACTGGATTGAGCTTCCGGGGATCCGTCGACCC  CTTCCTGACGTGCGAGCGCGGACCGCGGCCCCGCTGCGGAGCGGAGATGCTGTAGGCTGGAGCTGCTTCG | For hypothetical protein gene deletion | This work |
| Hp_del_chk_F  Hp_del_chk_R | AGCCTGGACGGTGCGGATCA  GCGGATCCGAGGAGATGCCG | To test for hypothetical protein gene deletion | This work |
| Del_pap2-p450_F  Del_pap2-p450_R | GCTCCGCAGCGGGGCCGCGGTCCGCGCTCGCACGTCAGGAAGGAGAGGTGTTCCGGGGATCCGTCGACCC  TCGCGGTTCCGGAGTTCCGCTGCCCCGGAGGCCCGCGCCGCGTACGACGGTGTAGGCTGGAGCTGCTTCG | For PAP2 superfamily protein, hypothetical protein, and a cytochrome P450 genes deletion | This work |
| Pap2-P450_del_chk_F  Pap2-P450_del_chk_R | TACTGCGTGCTGCTCCACCG  TCGACCTGGACGACGGCGAG | To test for PAP2 superfamily protein, hypothetical protein, and a cytochrome P450 genes deletion | This work |
| Del_prnD3-prnD2_F  Del_prnD3-prnD2_R | CTCCTCCCGAACACTCCGCTGCCGTTCGTGCGCTGCCCTCGGTCGGTCTCTGTAGGCTGGAGCTGCTTCG  CGGCTCCCAGGAGGACGTGCACACCGTCCCCGCCTCGGCTGCTCCGCCGATTCCGGGGATCCGTCGACCC | For putative aminopyrrolnitrin oxygenases (PrnD) genes deletion | This work |
| prnD3-prnD2_del_chk_F  prnD3-prnD2_del_chk_R | CCCGAGGCGAGGTTCGTGGT  CGGACTGATCACGCAGGCAG | To test for putative aminopyrrolnitrin oxygenases (PrnD) genes deletion | This work |
| STS40  STS41 | GTCCACGAGCTGATCACC  CTGCCAGGAGATCGAGTAC | For verification of heterologous constructs integration | This work |
| STS51  STS52 | CCGGCAAGGAGCTGCGCAAGCTCATGTCGTGGGTGAACGAAGAGGCCTGAGATACAGTTTAAACTTCAAATATGTATCCGCTCA  GGCGAACTCGGCGGAGCCGGTGATGACCGGCAGCCGAAGCCGCGGGGTTATCTATGGTTTAAACTTACCAATGCTTAATCAGTGAG | For construction pHSU-STS22 | This work |
| STS47  STS49 | CGTGCCCCGCTGCGGGCGTCGGCCCGTAGCGCCGGGCCTCGTACGTACCCGATACAGTTTAAACTTCAAATATGTATCCGCTCA  GTCGACGTCGATGGAGCCGGTGCGGGAGTTGAGCCGCAGGTCGGCGGGGCTCTATGGTTTAAACTTACCAATGCTTAATCAGTGAG | For construction pHSU-STS23 | This work |
| STS48  STS49 | GTGGGGGAGTCTCGCAGTACATCCTTTTTCCCTCAAGGAGTGGATCTCCCGATACAGTTTAAACTTCAAATATGTATCCGCTCA  GTCGACGTCGATGGAGCCGGTGCGGGAGTTGAGCCGCAGGTCGGCGGGGCTCTATGGTTTAAACTTACCAATGCTTAATCAGTGAG | For construction pHSU-STS24 | This work |
| STS50  STS49 | GGCCATGCCGGCGAGGCCCCGCCCCGCAGACGAAGCGGTTCCGCCCGGCAGATACAGTTTAAACTTCAAATATGTATCCGCTCA  GTCGACGTCGATGGAGCCGGTGCGGGAGTTGAGCCGCAGGTCGGCGGGGCTCTATGGTTTAAACTTACCAATGCTTAATCAGTGAG | For construction pHSU-STS25 | This work |
| STS51  STS71 | CCGGCAAGGAGCTGCGCAAGCTCATGTCGTGGGTGAACGAAGAGGCCTGAGATACAGTTTAAACTTCAAATATGTATCCGCTCA  GCCGAGGCCGCGGACCTGCCCGTCGAACGGGTGGGGGGCAAGGCCAAGGGTCTATGGTTTAAACTTACCAATGCTTAATCAGTGAG | For construction pHSU-STS29 | This work |
| STS51  STS72 | CCGGCAAGGAGCTGCGCAAGCTCATGTCGTGGGTGAACGAAGAGGCCTGAGATACAGTTTAAACTTCAAATATGTATCCGCTCA  CGACCGAGGGCAGCGCACGAACGGCAGCGGAGTGTTCGGGAGGAGGCCGGTCTATGGTTTAAACTTACCAATGCTTAATCAGTGAG | For construction pHSU-STS30 | This work |
| STS67  STS68 | ATGGACGCCGTCACCCAGGTCCCCGCGCCGGTCAACGAGCCGGTGCACGGGATACAGTTTAAACTTCAAATATGTATCCGCTCA  TCAGCCCATGTGCGGGTACGCGTAGTCCGTCGGCGAGACCAGCGTCTCCTTCTATGGTTTAAACTTACCAATGCTTAATCAGTGAG | For construction pHSU-STS31 | This work |
| STS69  STS68 | GTTTCGCATATCCAGGTACGGCCCTGTTTCTACTCTCCGTGGAGGTGCCCGATACAGTTTAAACTTCAAATATGTATCCGCTCA  TCAGCCCATGTGCGGGTACGCGTAGTCCGTCGGCGAGACCAGCGTCTCCTTCTATGGTTTAAACTTACCAATGCTTAATCAGTGAG | For construction pHSU-STS32 | This work |
| STS70  STS68 | TACGCGGCGCGGGCCTCCGGGGCAGCGGAACTCCGGAACCGCGAATGCTCGATACAGTTTAAACTTCAAATATGTATCCGCTCA  TCAGCCCATGTGCGGGTACGCGTAGTCCGTCGGCGAGACCAGCGTCTCCTTCTATGGTTTAAACTTACCAATGCTTAATCAGTGAG | For construction pHSU-STS33 | This work |
| KO_groA_F  KO_groA_R | ATGAGGCGCAGTACGTCTGAGCGGATCGCTGCGTCCGCGAGGCCGACCAGGCGCGCCAATACTTGACATATCACTGT  TCAGCCATGCTCTGCCACCTCCACCCACTGCTGATAGAAACGCCGGAATCGTCAGGCGCCGGGGGCGGTGT | For construction pHSU-STS33_KOgroA | This work |
| KO_groB_chk_F  KO_groB_chk_R | ACCAGGTCCTCACCTTCG  TACGGGAAATCCCGCTGC | To test for *groA* gene deletion | To test for *groA* gene deletion |
| KO_groB_F  KO_groB_R | ATGAGTCTCGCGCCGGTACGAGGGCAGCCTCCCCTCGATCTCGAACCGCGGCGCGCCAATACTTGACATATCACTGT  TCACGTGTCCCCCCGCCCCGTCCAACGGCGATGGAACTGGCGGAATCGCAGGGCGCGCCTCAGGCGCCGGGGGCGGTGT | For construction pHSU-STS33_KOgroB | This work |
| KO_groB_chk_F  KO_groB_chk_R | AGGTGATCATGGGCATCGTC  AAGACACATGCTCCGGACTC | To test for *groB* gene deletion | To test for *groB* gene deletion |
| KO_groC_F  KO_groC_R | ATGATCGGCGCGGACGGCTCCCAGGAGGACGTGCACACCGTCCCCGCCTGGCGCGCCAATACTTGACATATCACTGT  TCATCGGCCGACCTTTCCGTCTCCCGCGGCGCGGTCGACCCATGACTGGTAGGCGCGCCTCAGGCGCCGGGGGCGGTGT | For construction pHSU-STS33_KOgroC | This work |
| KO_groC_chk_F  KO_groC_chk_R | GAAGGACGACATAGCCCACC  ACATCCCGTCACTGTCGAAC | To test for *groC* gene deletion | This work |
| KO_groD_F  KO_groD_R | ATGGAAGAGTTCCGTCGCCAGGTCAACGAAAGACTGCGCTCCTCCATCGGGCGCGCCAATACTTGACATATCACTGT  TCATGCCGCCGCCCGGCCGTGAACCACCTGCGCCGCCCAGCGAAGCATCGCGGCGCGCCTCAGGCGCCGGGGGCGGTGT | For construction pHSU-STS33_KOgroD | This work |
| KO_groE_F  KO_groE_R | ATGGACGTCTTATTAGGGCACTCTCCGTCTGTTCTGGGATGCGTTCTGTGGCGCGCCAATACTTGACATATCACTGT  TCAGATCGTCCGTACCCGCGCGTCCGCGCCGGGCATCGTCGTGGACAGCGGGGCGCGCCTCAGGCGCCGGGGGCGGTGT | For construction pHSU-STS33_KOgroE | This work |
| KO_groF_F  KO_groF_R | ATGCGGCGAAATCTCAGGCTGGCGGCTTCTCAACGCGCCAAACGGTTGTGGCGCGCCAATACTTGACATATCACTGT  TCAGAGCCGGCGCCAAGGCGACATACCGGCGGCCTTGAGGGAGCGGTGGAGGGCGCGCCTCAGGCGCCGGGGGCGGTGT | For construction pHSU-STS33_KOgroF | This work |
| KO_groF_chk_F  KO_groF_chk_R | CAGAACAGACGGAGAGTGCC  CACCAGTACGCCGAGGAC | To test for *groF* gene deletion | This work |
| KO_groG_F  KO_groG_R | GTGAACGTGCCCAGCCAACAGGACGCCGACCACGCCGTGACCGGCGCCGGGCGCGCCAATACTTGACATATCACTGT  TCATCGGGTTTCCCTTCCAGAGGAGTGCGGGGGCCCGGCGTCGACACGCCAGGCGCGCCTCAGGCGCCGGGGGCGGTGT | For construction pHSU-STS33_KOgroG | This work |
| KO_groG_chk_F  KO_groG_chk_R | CTCAAGATGCTGGAGGACCG  ACATCGTCGTTCCATGCTCC | To test for *groG* gene deletion | This work |
| KO_groH_F  KO_groH_R | ATGAACAGCTTGGCCCGCCTGGCGGAGACCCCGTTGTGGAAGGCGGCGGGGCGCGCCAATACTTGACATATCACTGT  TCATGCGGTGTGTCCTCTCGTCTTGCGAAGCGATGAAGGGGTGCGCGAGGGGGCGCGCCTCAGGCGCCGGGGGCGGTGT | For construction pHSU-STS33_KOgroH | This work |
| KO_groH_chk_F  KO_groH_chk_R | CGACTCCGGGTACAGCTTC  GAGACGAGTTTGCGCAACC | To test for *groH* gene deletion | This work |
| KO_groI_F  KO_groI_R | ATGAGCAGCGCGATGCGCCCCACCCCCGTCATGTTCAACCCGCTCTCCGGGCGCGCCAATACTTGACATATCACTGT  TCAGACGTGGACGGGAAGGTGAGTGGCCCCGCGCAGGCTCAGAAGGCCGTTGGCGCGCCTCAGGCGCCGGGGGCGGTGT | For construction pHSU-STS33_KOgroI | This work |
| KO_groI_chk_F  KO_groI_chk_R | TCTTCGTGAAGCTCTACGGC  TGAATCAGGCGCCTTAGACC | To test for *groI* gene deletion | This work |
| 9_flavoviridis_F  9_flavoviridis_R | GTAGTGGCGGAGGTCGTTG  GTCCTGGTGCTCCTGCTG | To test for *groH* homolog gene presence | This work |
| Left_flavoridis_F  Left_flavoridis_R  Right_flavor_F  Right_flavor_R | CGCAGTCTTCGGGGAGAC  GGTCCACTCCGTCGTTGG  GTTCCAGCAGGACGTCGAA  GACCATCATCGGCACCATGA | To identify the cosmid containing whole gromomycin-like cluster | This work |
| cosmid-chk-For_DB  cosmid-chk-rev_DB | GATCTCCATCGACTAAACGT  GTTAACTGCGGTCAAGATAT | To sequence the insert containing gromomycin-like cluster from the left of right side | This work |

## Table S17. Clusters of secondary metabolism genes identified in the genome of Streptomyces sp. strain Je 1-332 by the type of biosynthesis and corresponding profile using antiSMASH 6.0 software.

| **antiSMASH**  **region** | **Type** | **Most similar known cluster/MIBiG BGC-ID** | **Similarity (%)** |
| --- | --- | --- | --- |
| 1 | Other | А-503083/ BGC0000288 | 7 |
| 2 | T1PKS | Salinomycin/ BGC0000144 | 12 |
| 3 | Ectoine | Ectoine/ BGC0000853 | 100 |
| 4 | Lanthipeptide class III | AmfS/BGC0000496 | 80 |
| 5 | Siderophore | Desferrioxamin B/ BGC0000940 | 83 |
| 6 | NRPS | Friulimicin A/ BGC0000354 | 78 |
| 7 | Butyrolactone | Granaticin/ BGC0000227 | 10 |
| 8 | T1PKS, Butyrolactone, Ectoine | Streptazone E/ BGC0001296 | 91 |
| 9 | Melanin | Melanin/ BGC0000910 | 100 |
| 10 | Terpene | Albaflavenone/ BGC0000660 | 100 |
| 11 | T3PKS, T1PKS, siderophore | RK-682/ BGC0000140 | 45 |
| 12 | RiPP-like | - | - |
| 13 | NRPS-terpene | Griseobactin/ BGC0000368 | 52 |
| 14 | Terpene | - | - |
| 15 | Terpene | Hopene/ BGC0000663 | 92 |
| 16 | T3PKS | BE-14106/ BGC0000029 | 17 |
| 17 | Siderophore | - | - |
| 18 | RiPP-like | - | - |
| 19 | Terpene | 2-methyllisoborneol/ BGC0000658 | 100 |
| 20 | Terpene | Isorenieratene/ BGC0000664 | 100 |
| 21 | T3PKS | Alkylresorcinol/ BGC0000282 | 100 |

Table S18. Toxicity assessment of gromomycin derivatives.

*In vitro* cytotoxicity was evaluated performing a MTT assay (half maximal inhibitory concentration IC_50_ ± standard deviation). *In vivo* toxicity was assessed by determination of the maximum tolerated concentration (MTC) in zebrafish (*Danio rerio*) embryos. Kaplan-Meier curves of embryos exposed to gromomycins can be found in Supplementary Fig. 68. CHO: chinese hamster ovary.

| Gromomycin derivative | *In vitro* IC_50_ [µg mL^-1^] | | *In vivo* MTC [µg mL^-1^] |
| --- | --- | --- | --- |
|  | **HepG2** | **CHO-K1** | ***Danio rerio*** |
| Gromomycin A | 9.0 ± 6.6 | 23.9 ± 0.7 | 1 |
| Gromomycin B | > 37 | > 37 | 5 |
| Gromomycin E | 19.4 ± 1.2 | 31.2 ± 2.5 | 5 |
| Gromomycin F | > 37 | 27.0 ± 2.5 | 5 |
| Gromomycin G | > 37 | > 37 | 10 |
| Gromomycin H | 2.2 ± 0.4 | 4.1 ± 1.1 | nd^a^ |

^a^ not determined (nd) due to poor solubility in the incubation medium

# List of Figures


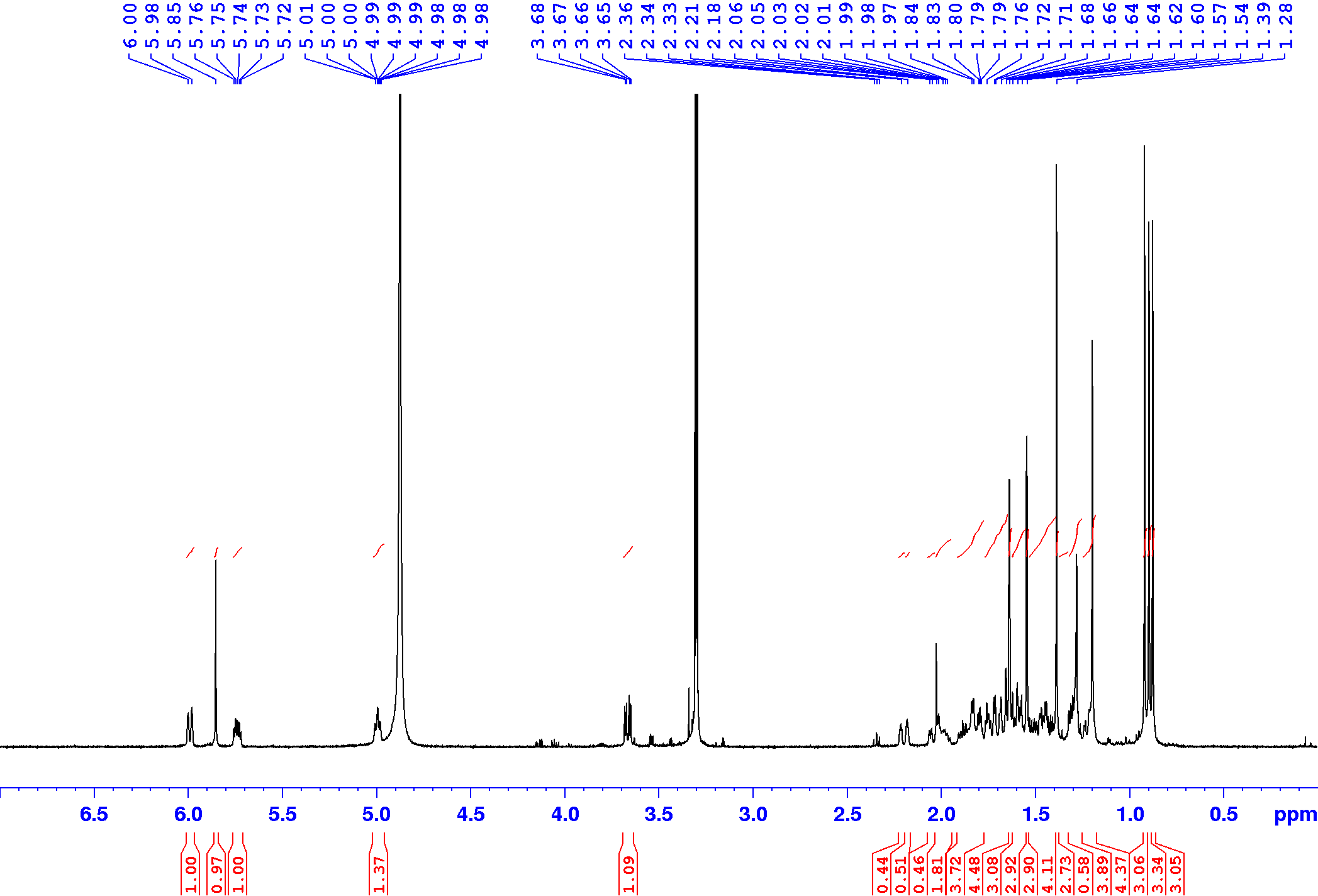


## Figure S1. ^1^H NMR spectrum (500 MHz) of Gromomycin A (2) in CD_3_OD.

**
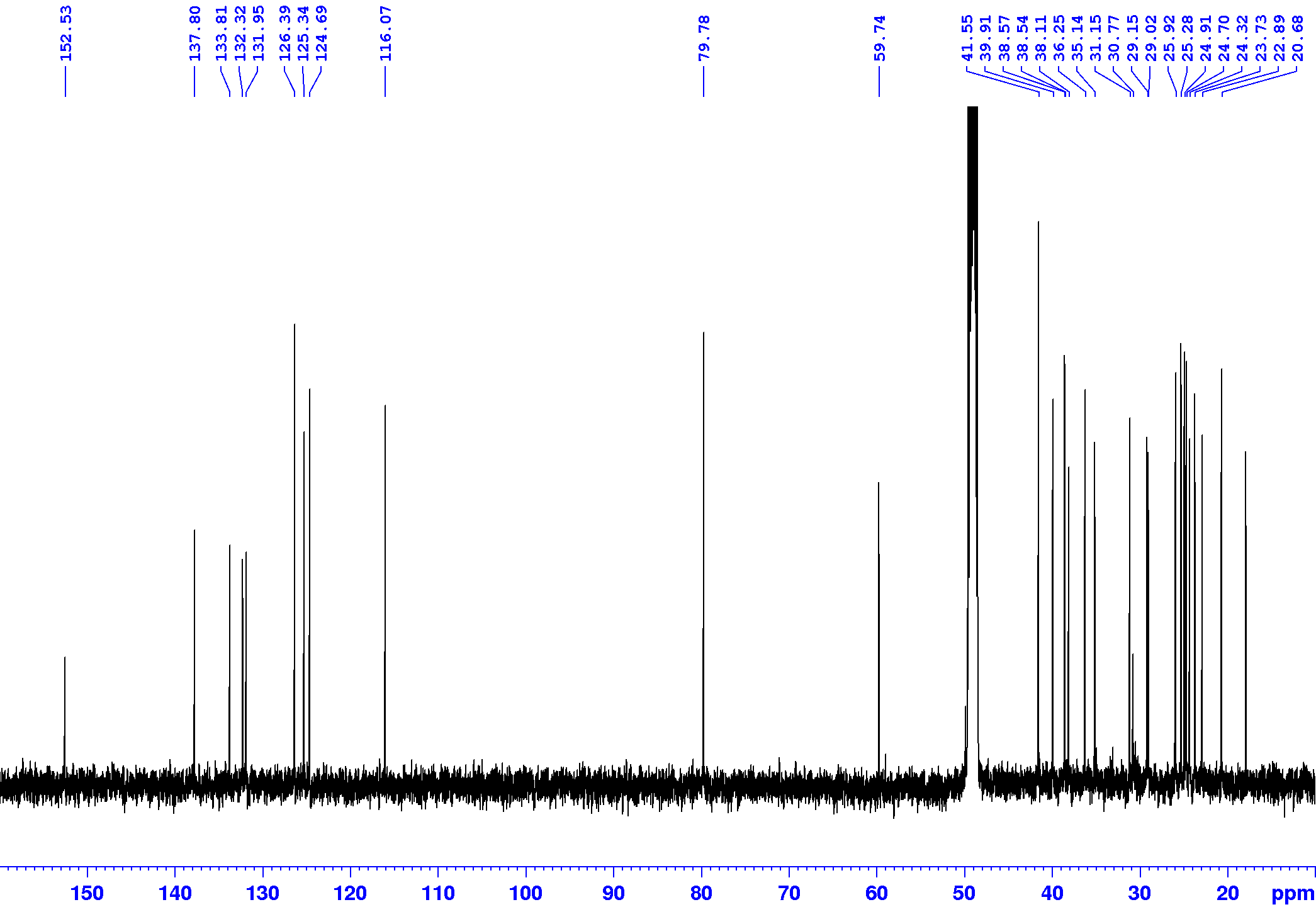
**

## Figure S2. ^13^C NMR spectrum (125 MHz) of Gromomycin A (2) in CD_3_OD.

**
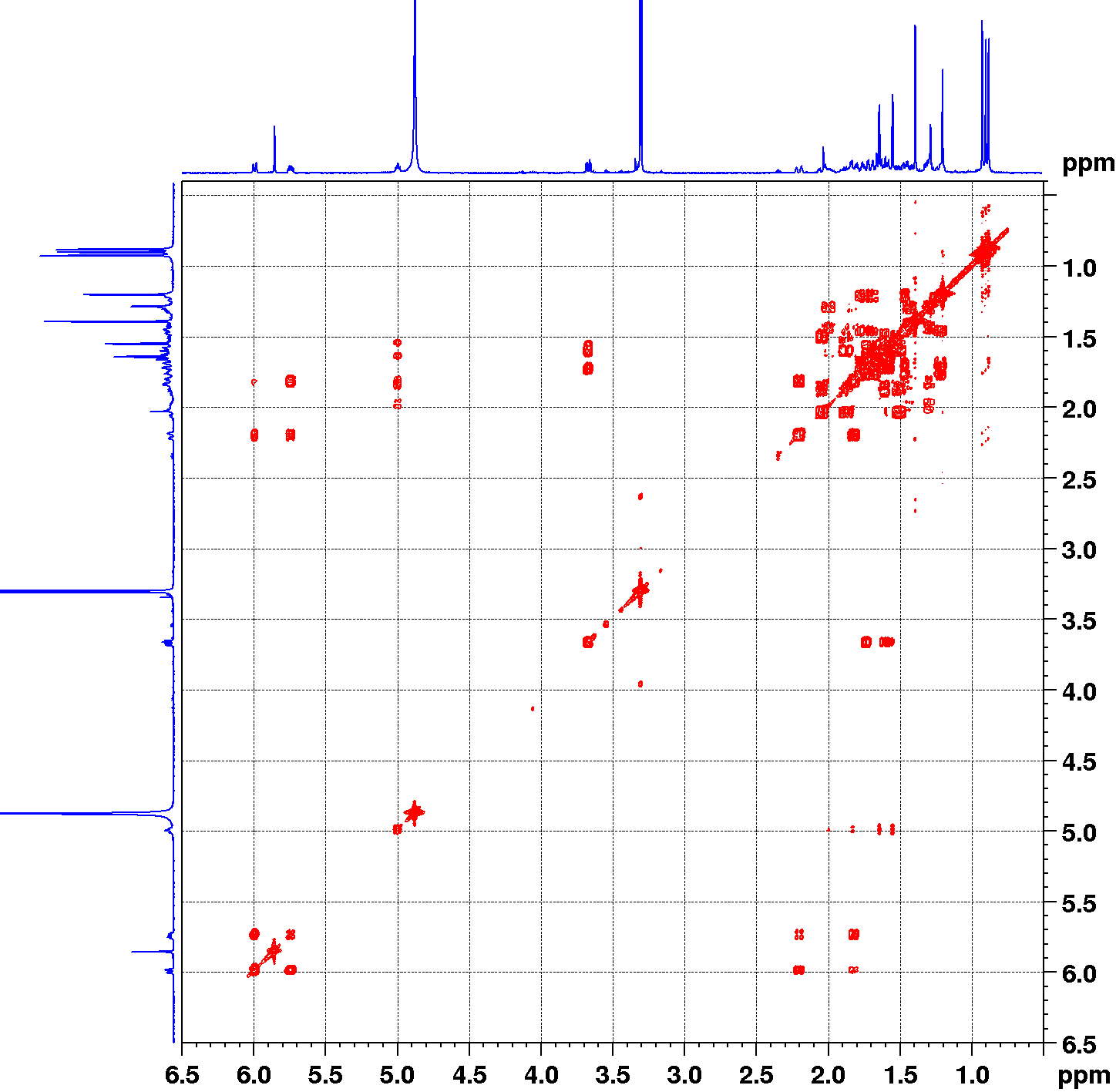
**

## Figure S3. COSY spectrum (500 MHz) of Gromomycin A (2) in CD_3_OD.

**
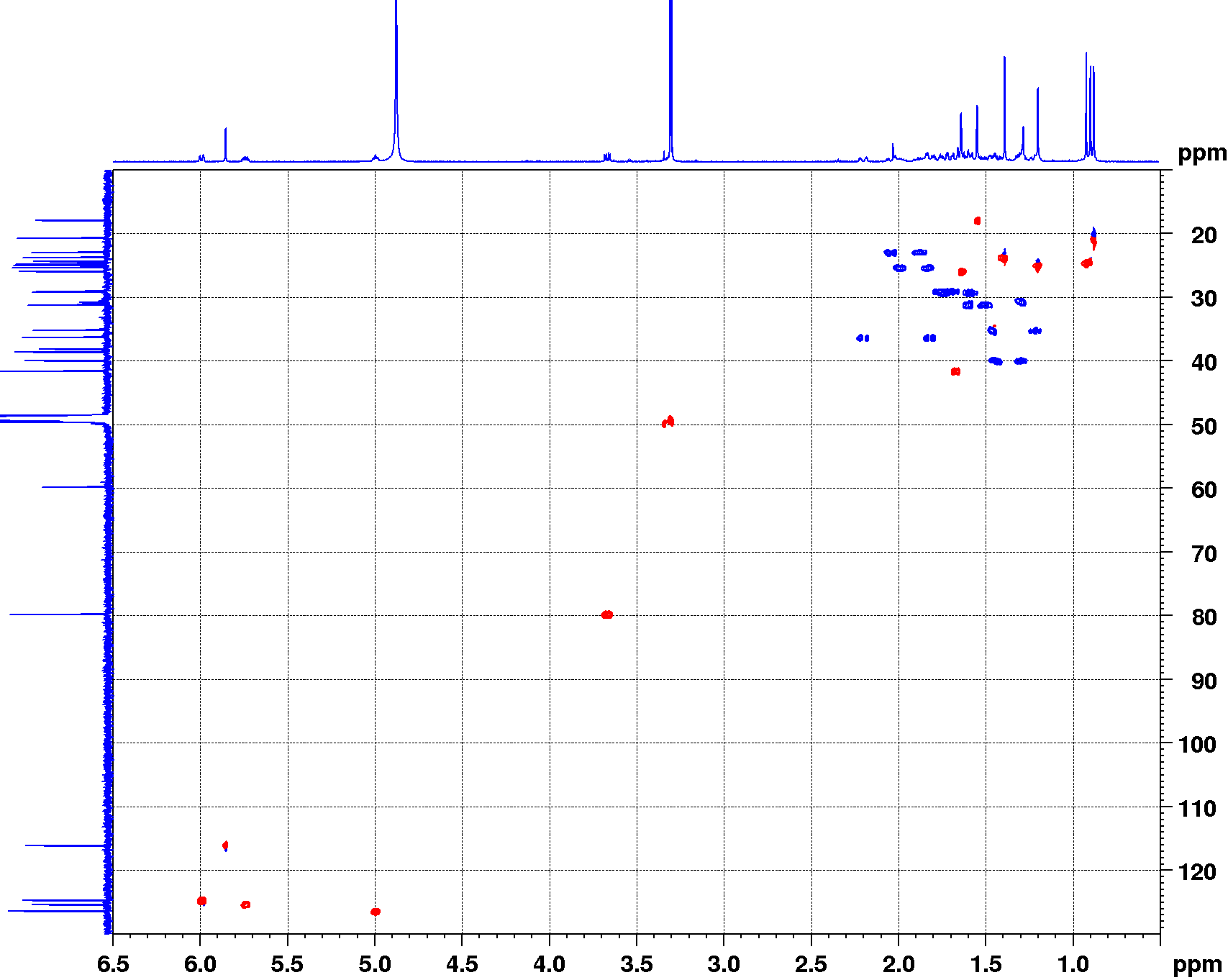
**

## Figure S4. ^1^H-^13^C HSQC spectrum (500 MHz) of Gromomycin A (2) in CD_3_OD.

**
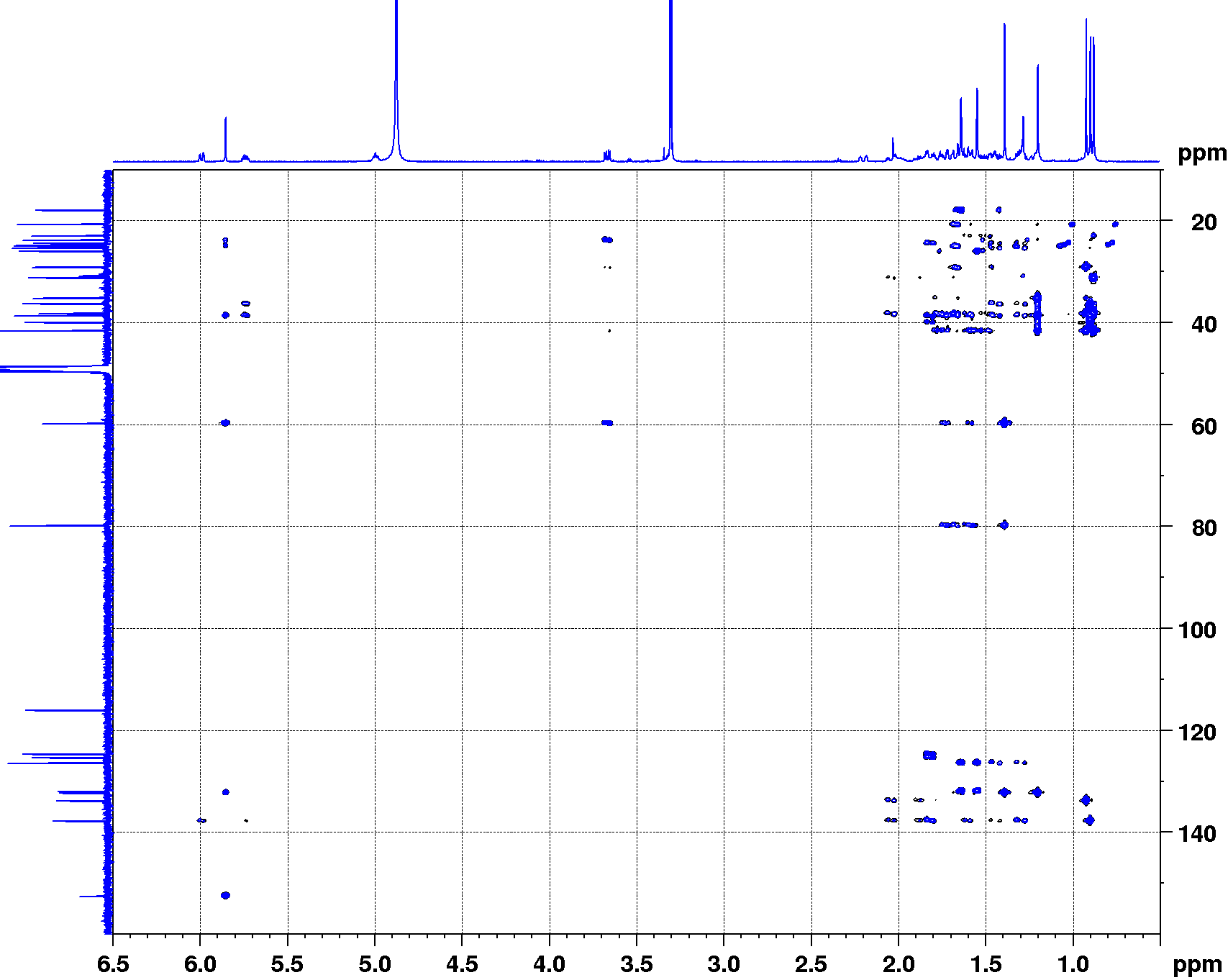
**

## Figure S5. ^1^H-^13^C HMBC spectrum (500 MHz) of Gromomycin A (2) in CD_3_OD.

_
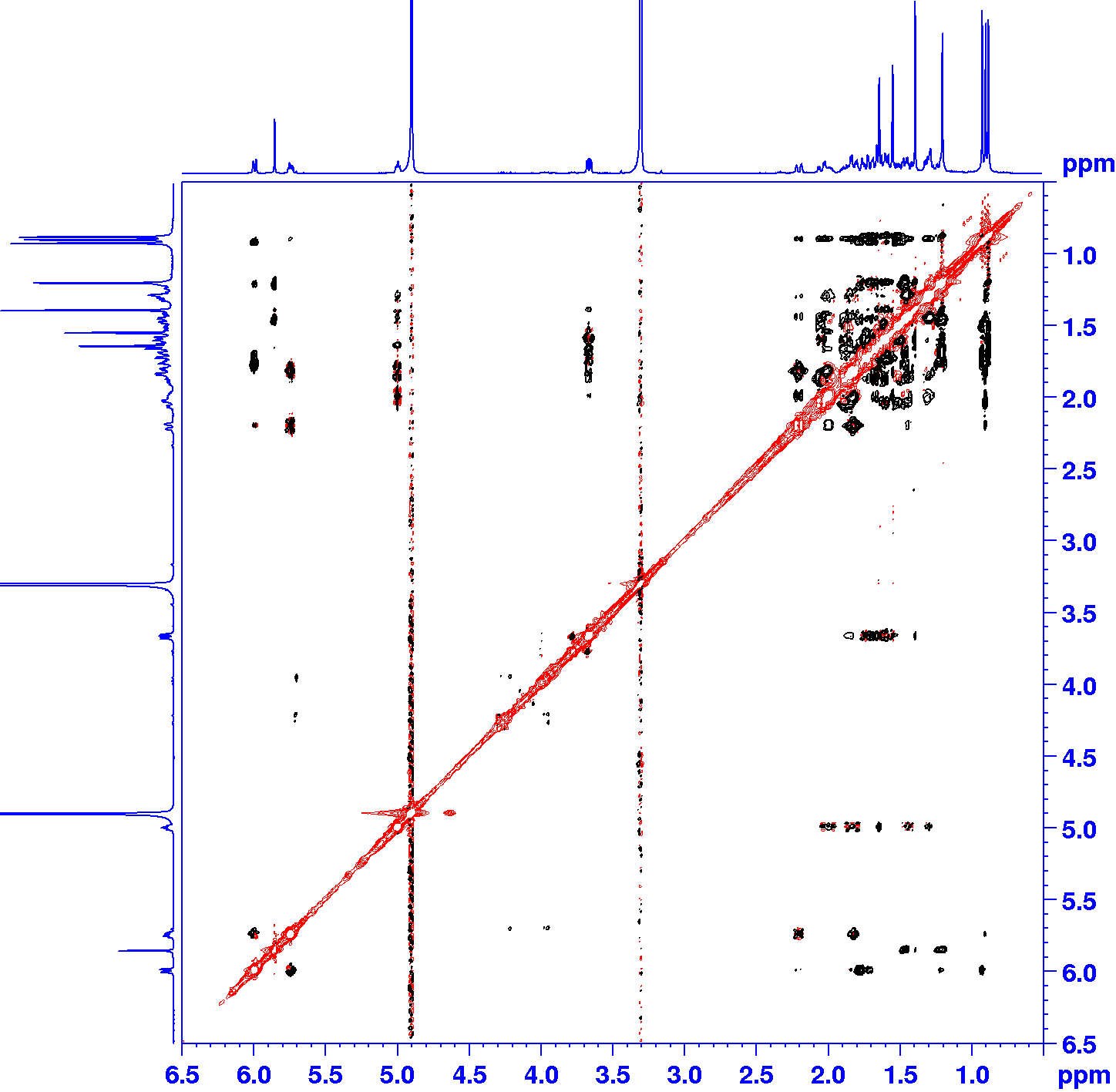
_

## Figure S6. NOESY spectrum (500 MHz) of Gromomycin A (2) in CD_3_OD.


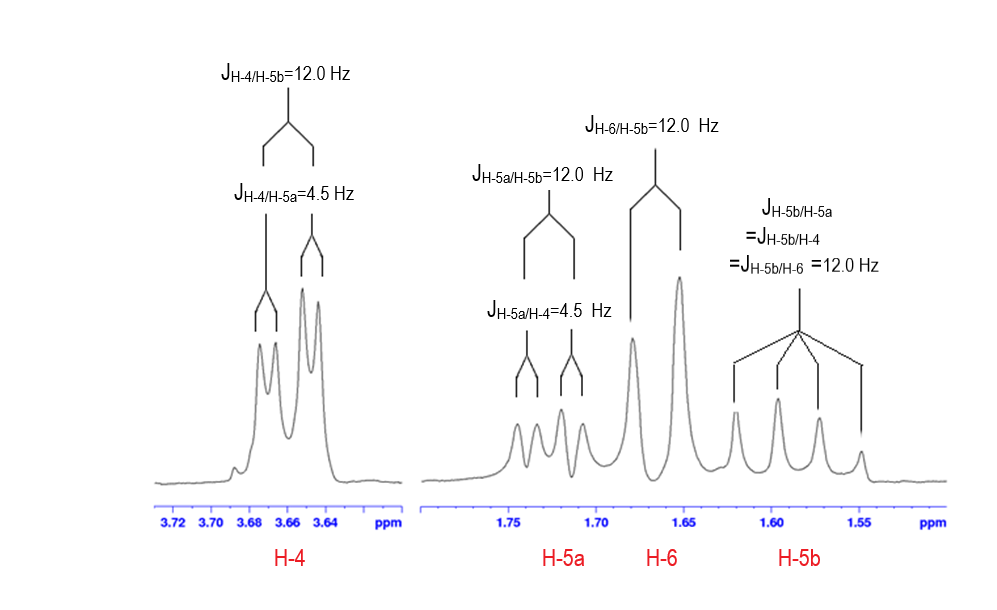


**a)**

**
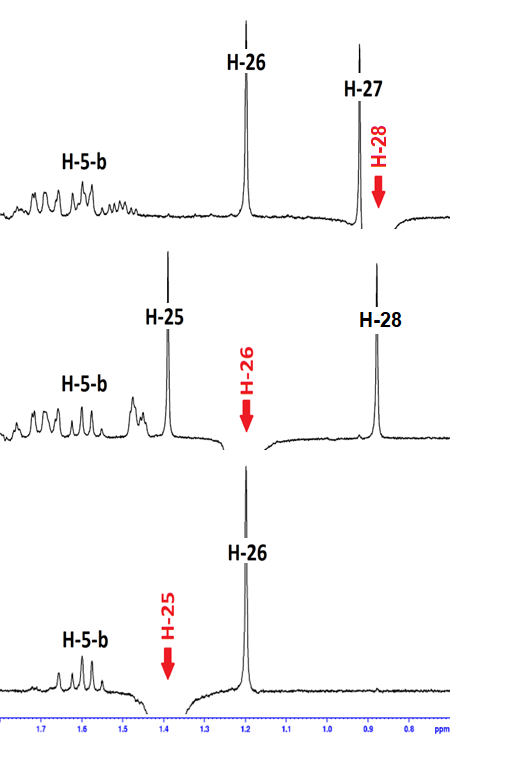
**

**b.)**

## Figure S7. Selective 1D experiments of gromomycin (2). a) selective TOCSY with excitation of H-4 (δ_H_ 3.66). b) 1D selective NOESY´s with excitations of H-25 (δ_H_ 1.40), H-26 (δ_H_ 1.20) and H-28 (δ_H_ 0.88).


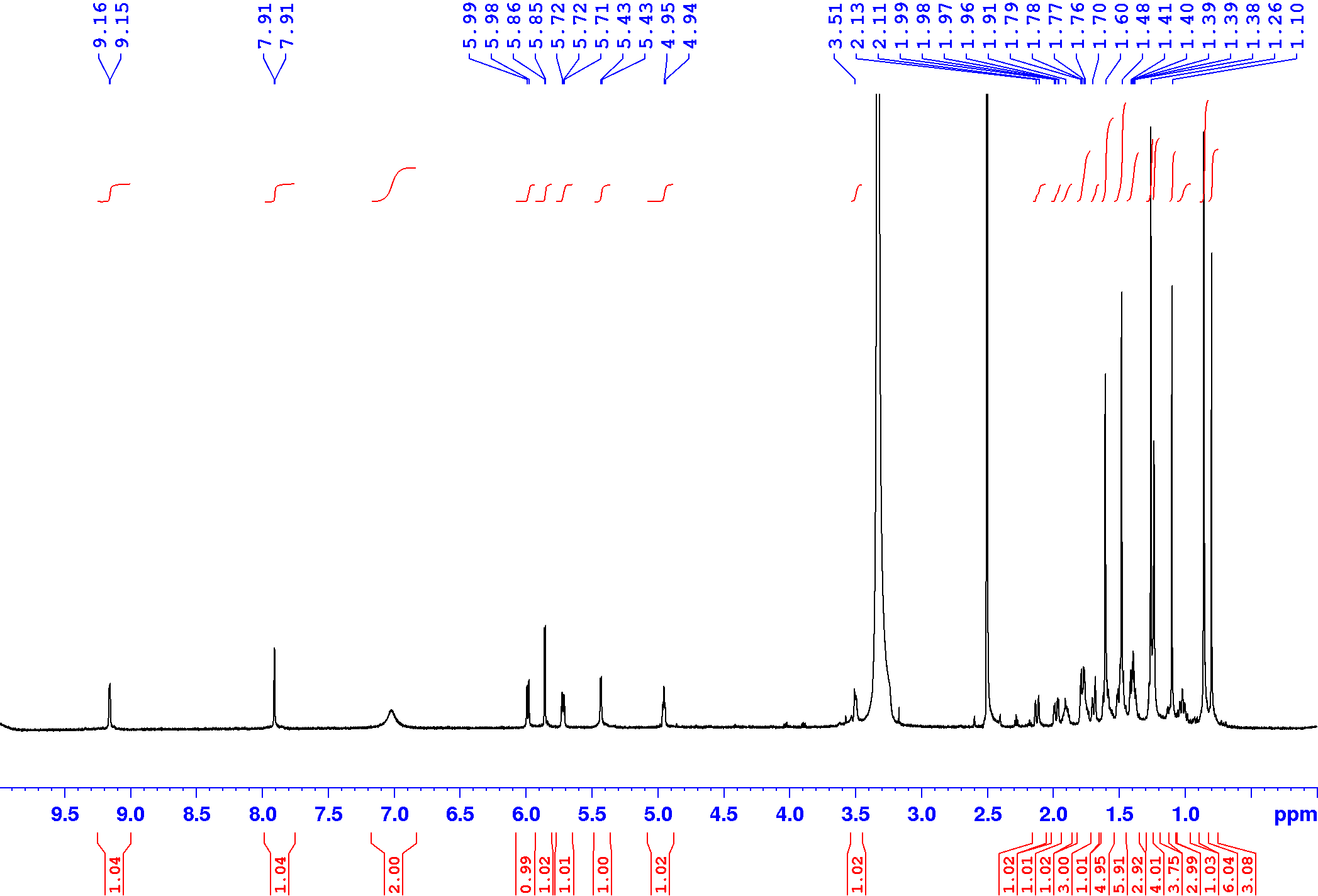


## Figure S8. ^1^H NMR spectrum (700 MHz) of Gromomycin A (2) in DMSO-d_6_/1 drop TFA-d_1._


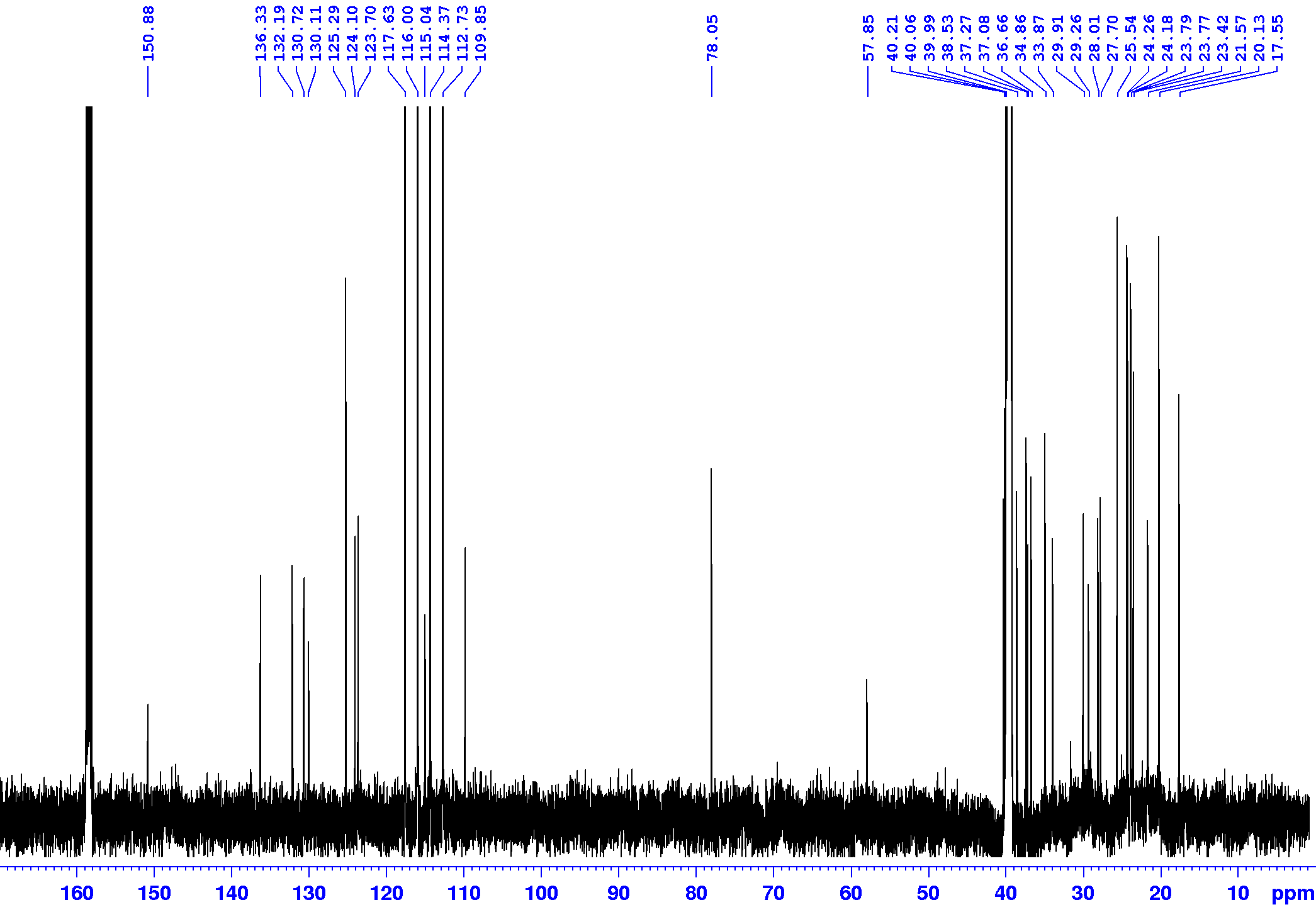


## Figure S9. ^13^C NMR spectrum (175 MHz) of Gromomycin A (2) in DMSO-d_6_/1 drop TFA.


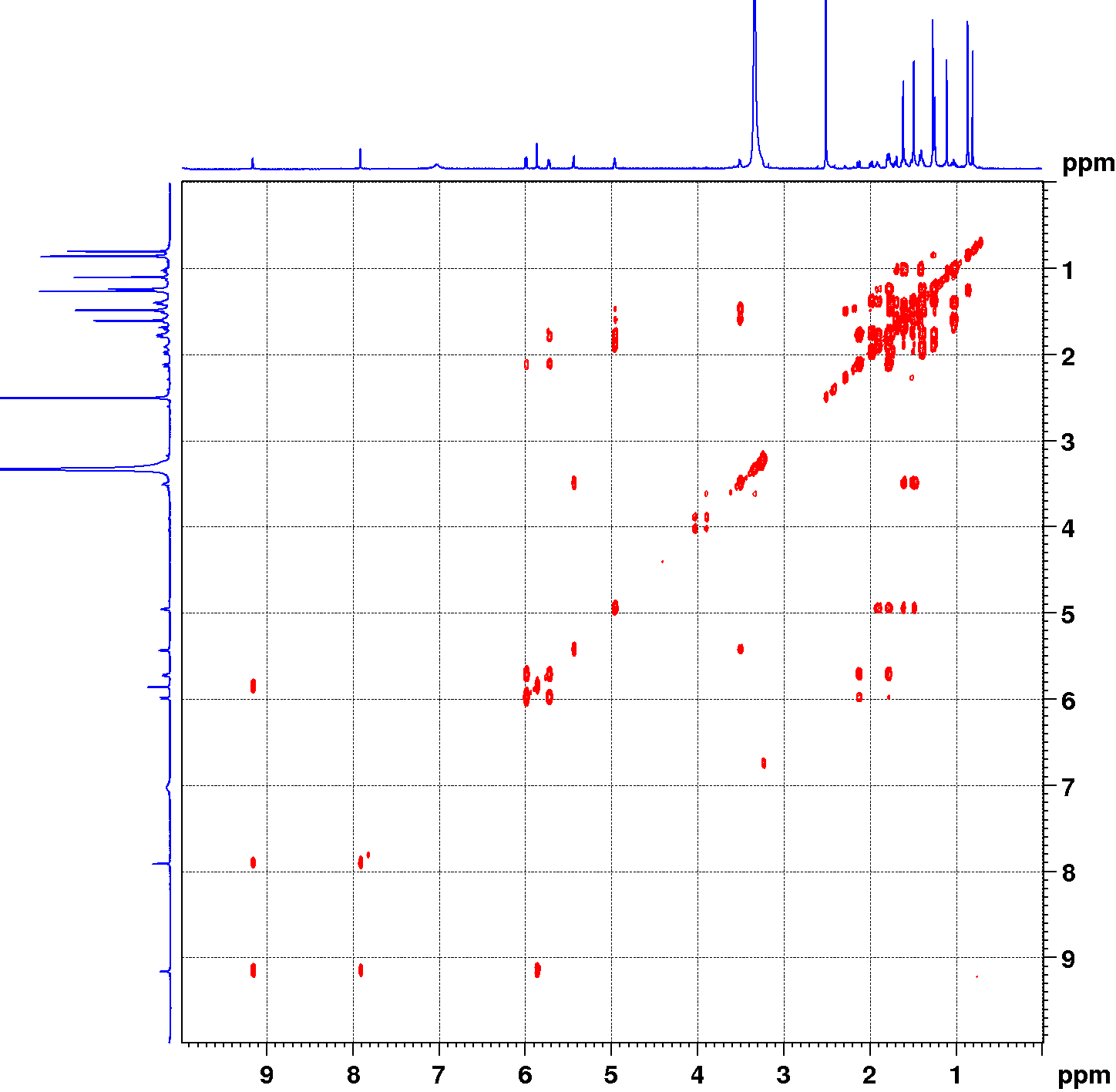


##
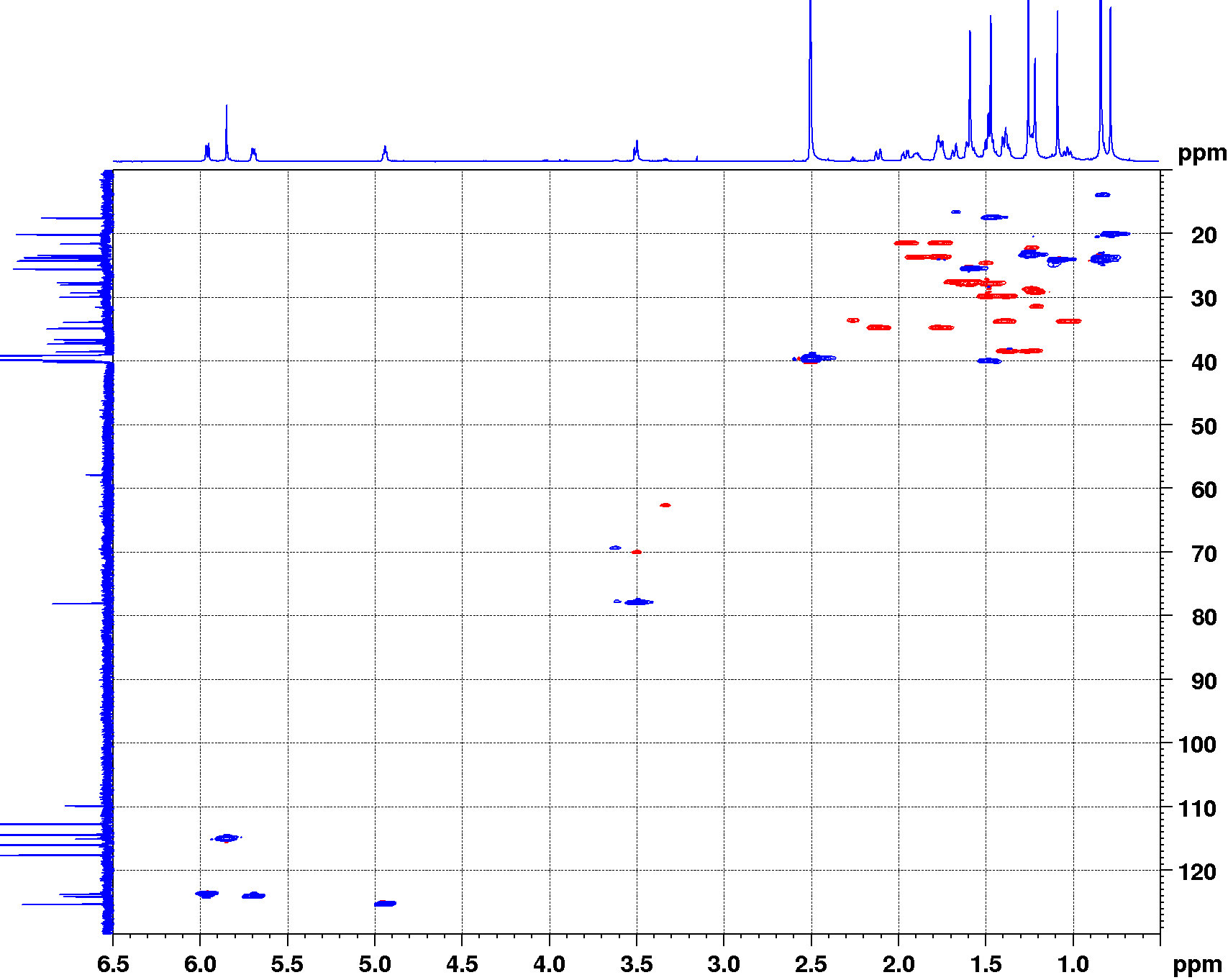
Figure S10. COSY spectrum (700 MHz) of Gromomycin A (2) in DMSO-d_6_/1 drop TFA.

## Figure S11. ^1^H-^13^C HSQC spectrum (700 MHz) of Gromomycin A (2) in DMSO-d_6_/1 drop TFA.


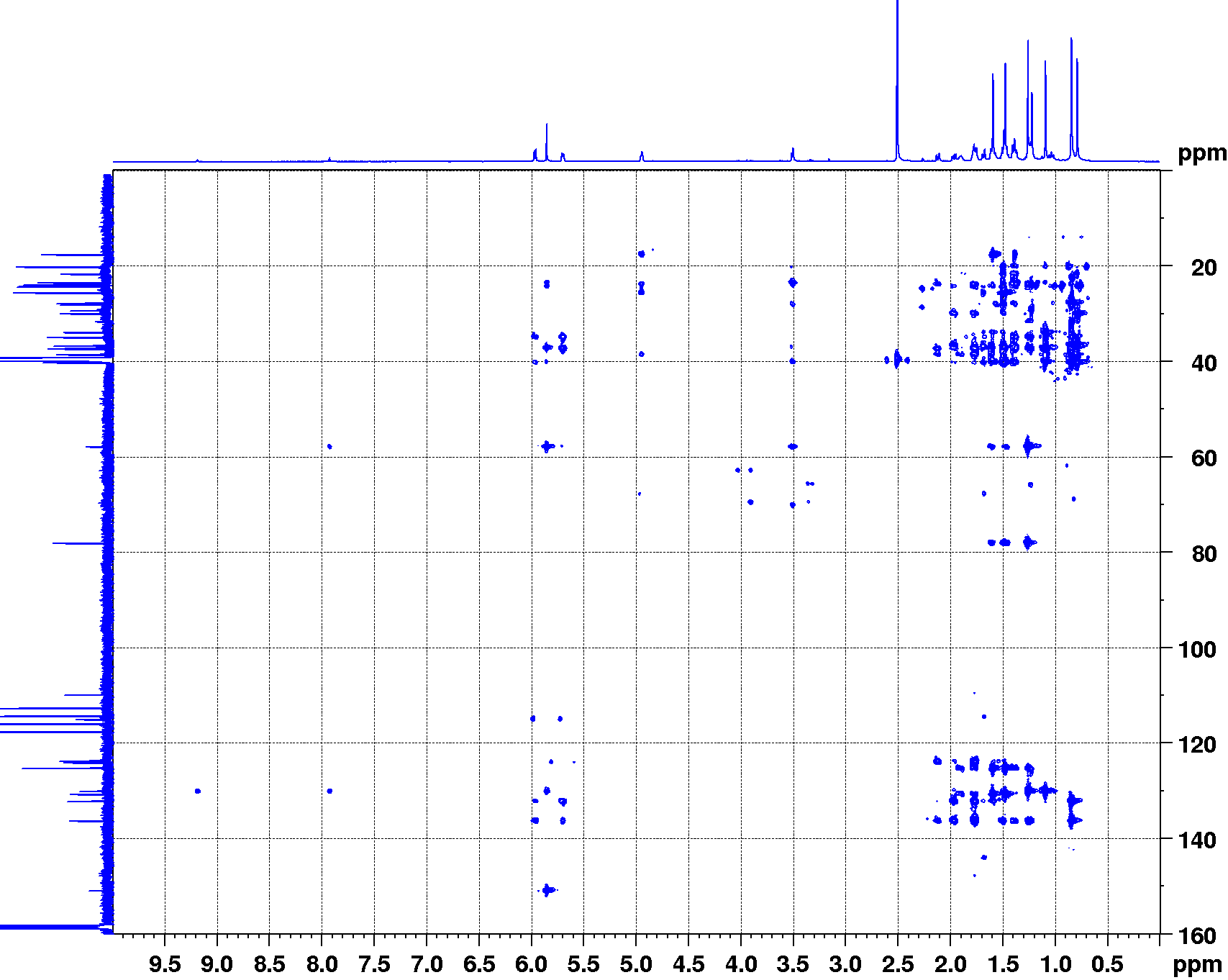


## Figure S12. ^1^H-^13^C HMBC spectrum (700 MHz) of Gromomycin A (2) in DMSO-d_6_/1 drop TFA.


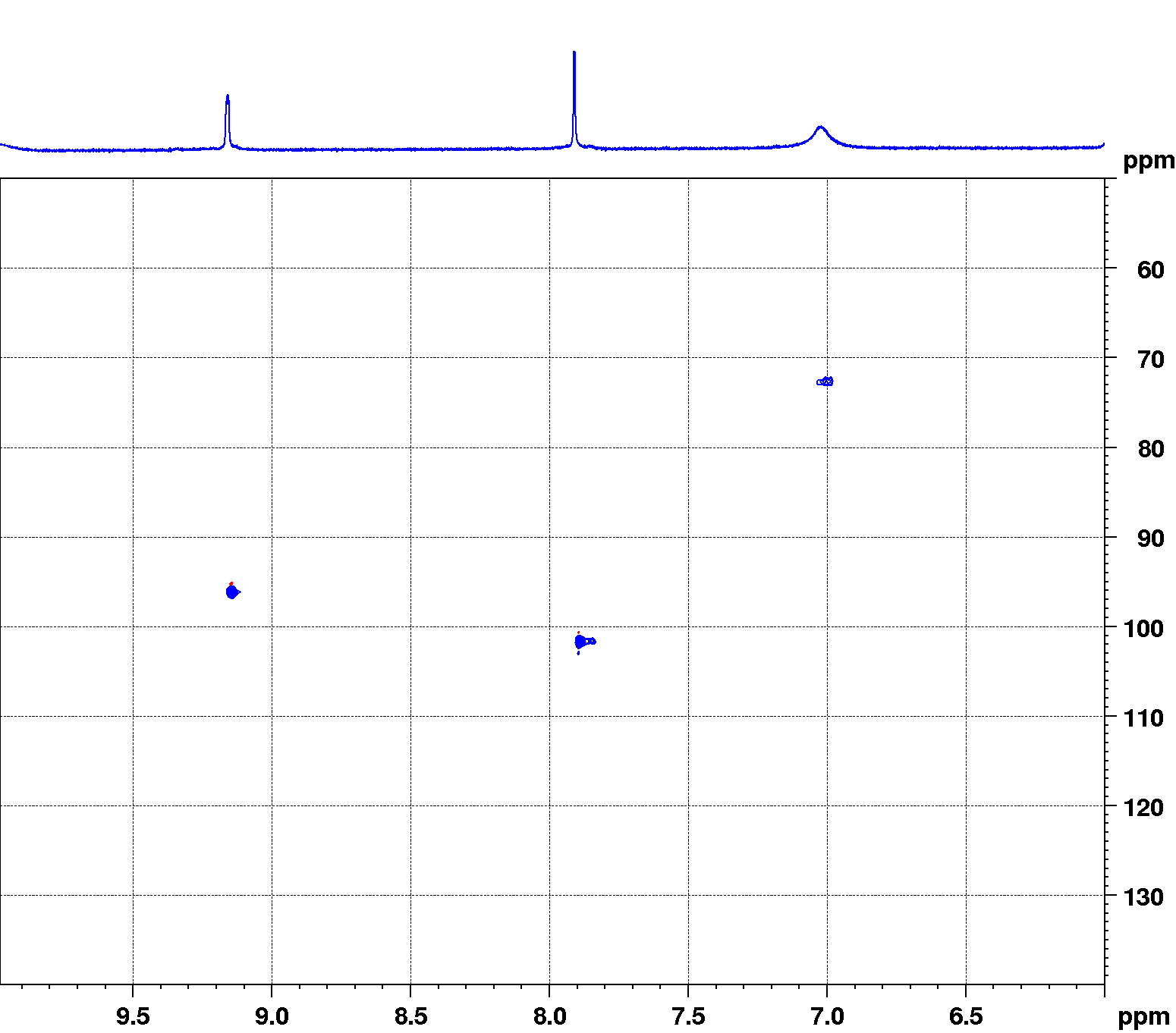


## Figure S13. 1H-^15^N HSQC spectrum of Gromomycin A (2) in DMSO-d_6_/1 drop TFA.

_
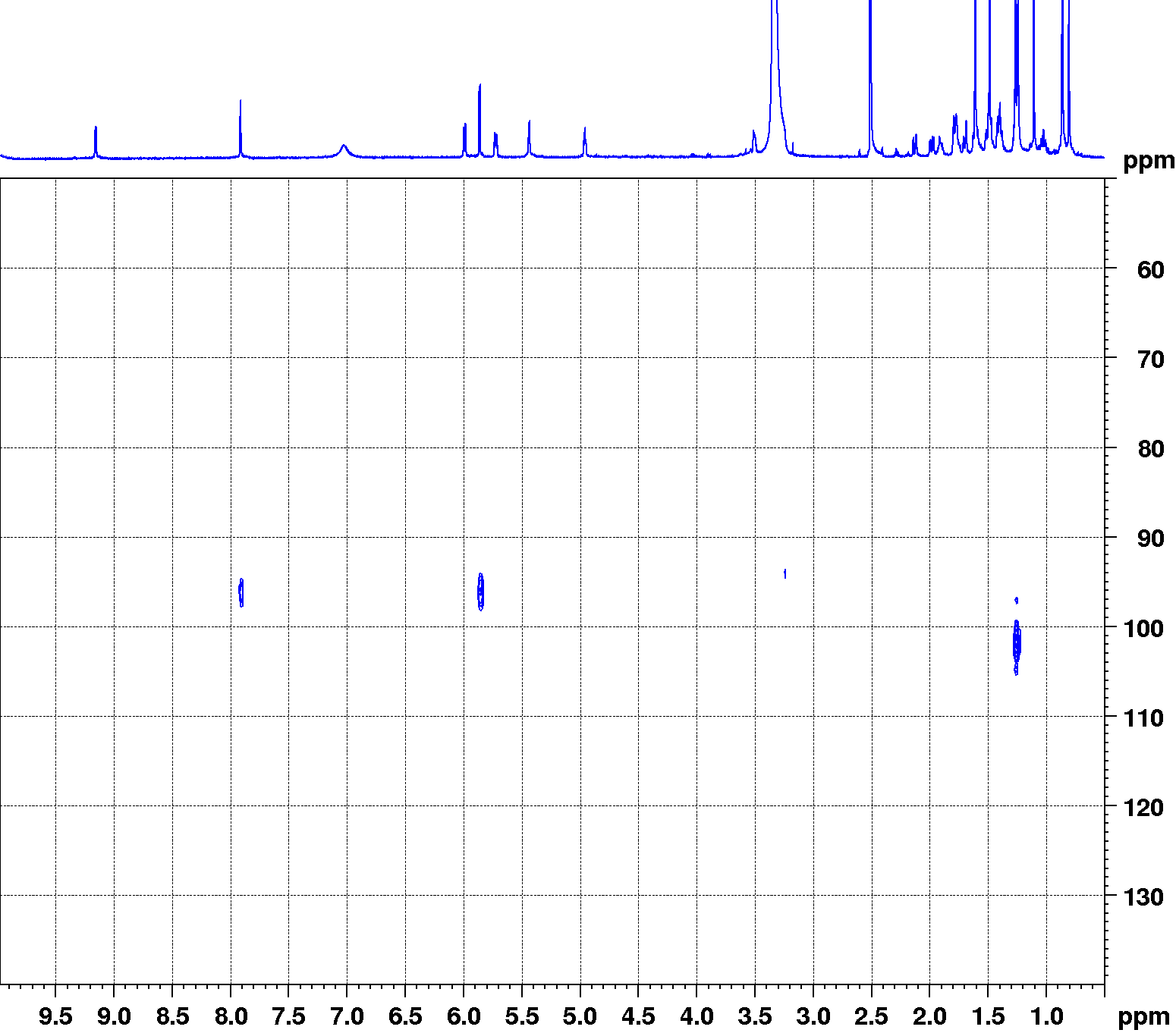
_

## Figure S14. 1H-^15^N HMBC spectrum of Gromomycin A (2) in DMSO-d_6_/1 drop TFA.

**
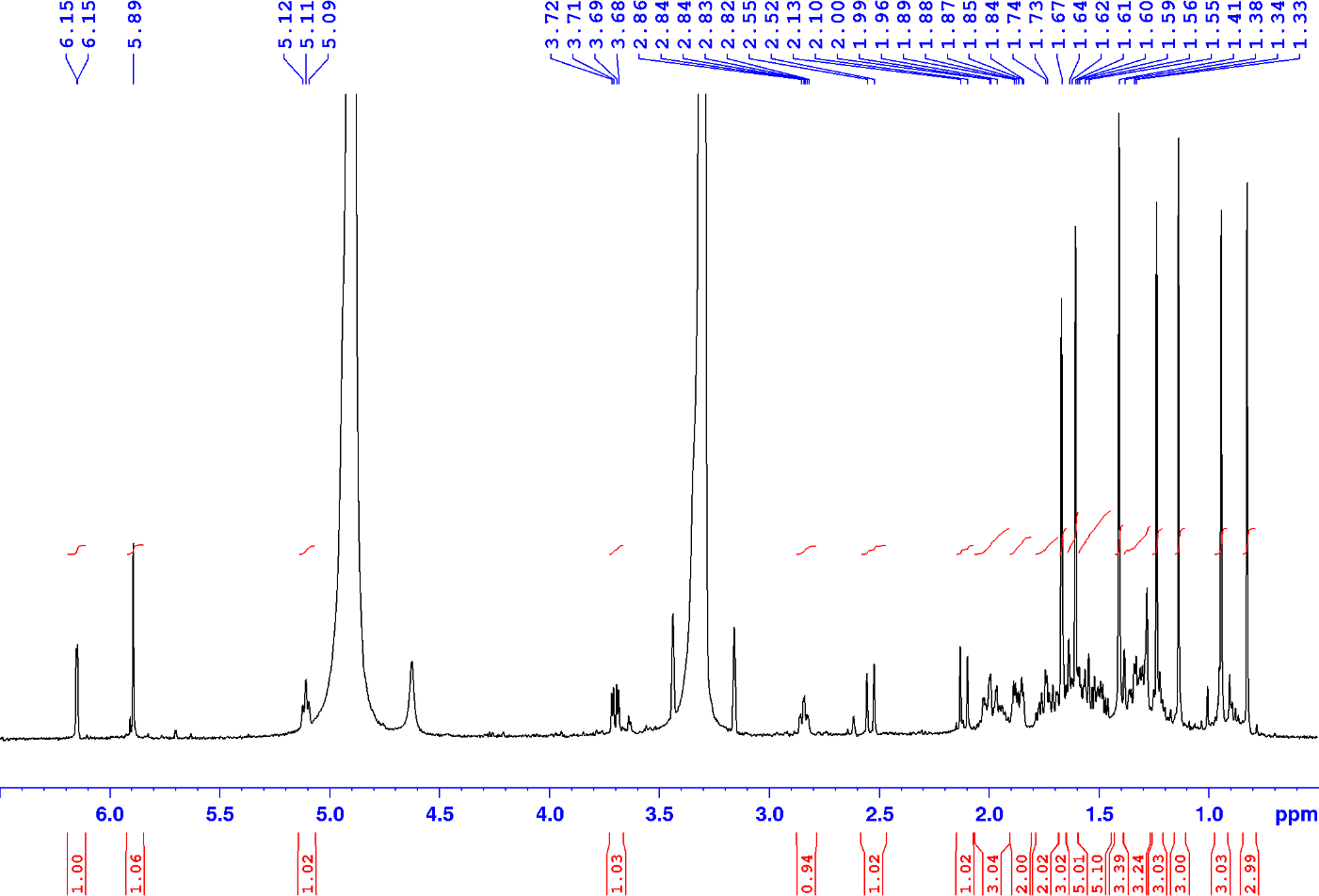
**

## Figure S15. ^1^H NMR spectrum (500 MHz) of Gromomycin B (3) in CD_3_OD.

**
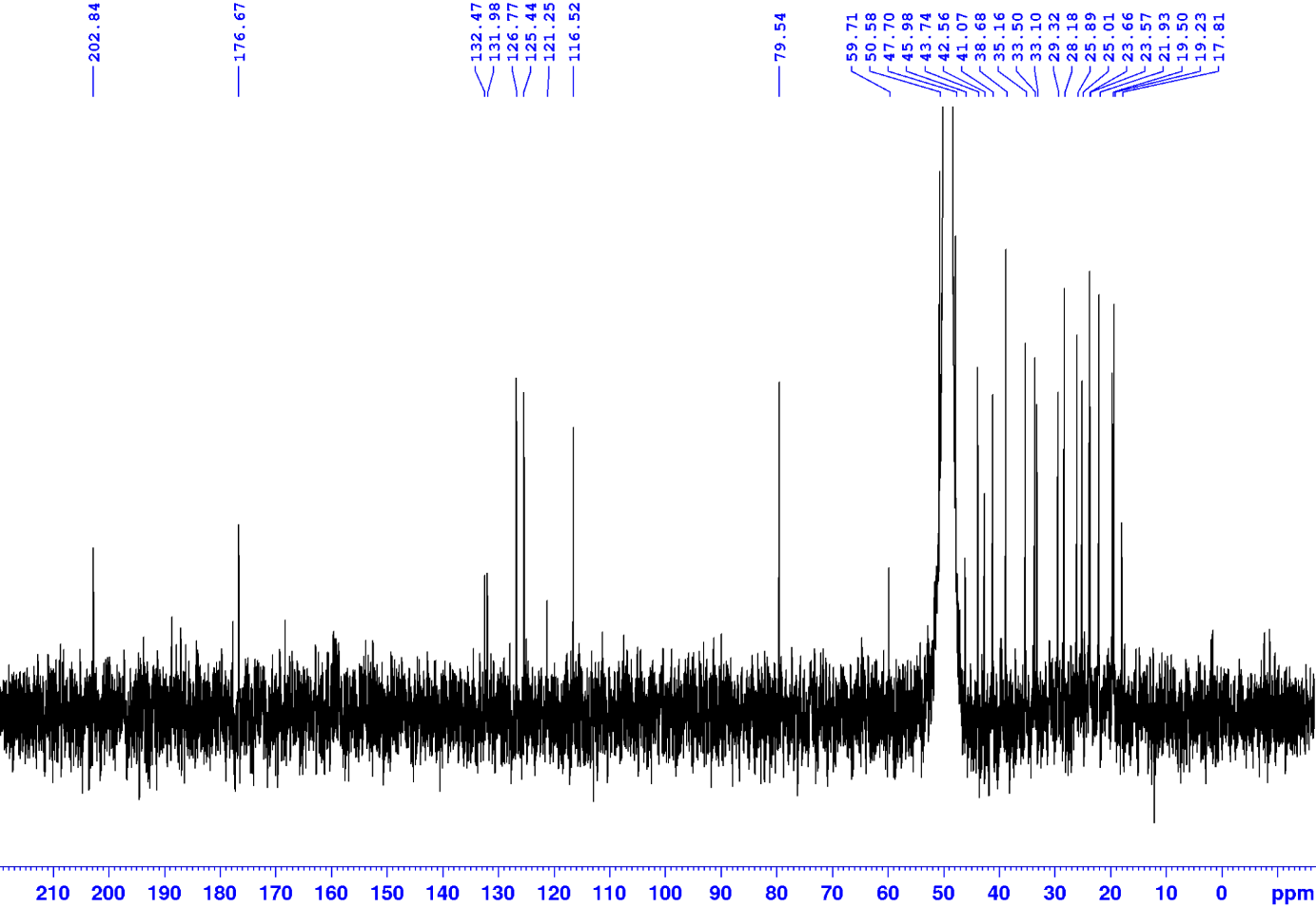
**

## Figure S16. ^13^C NMR spectrum (125 MHz) of Gromomycin B (3) in CD_3_OD.

**
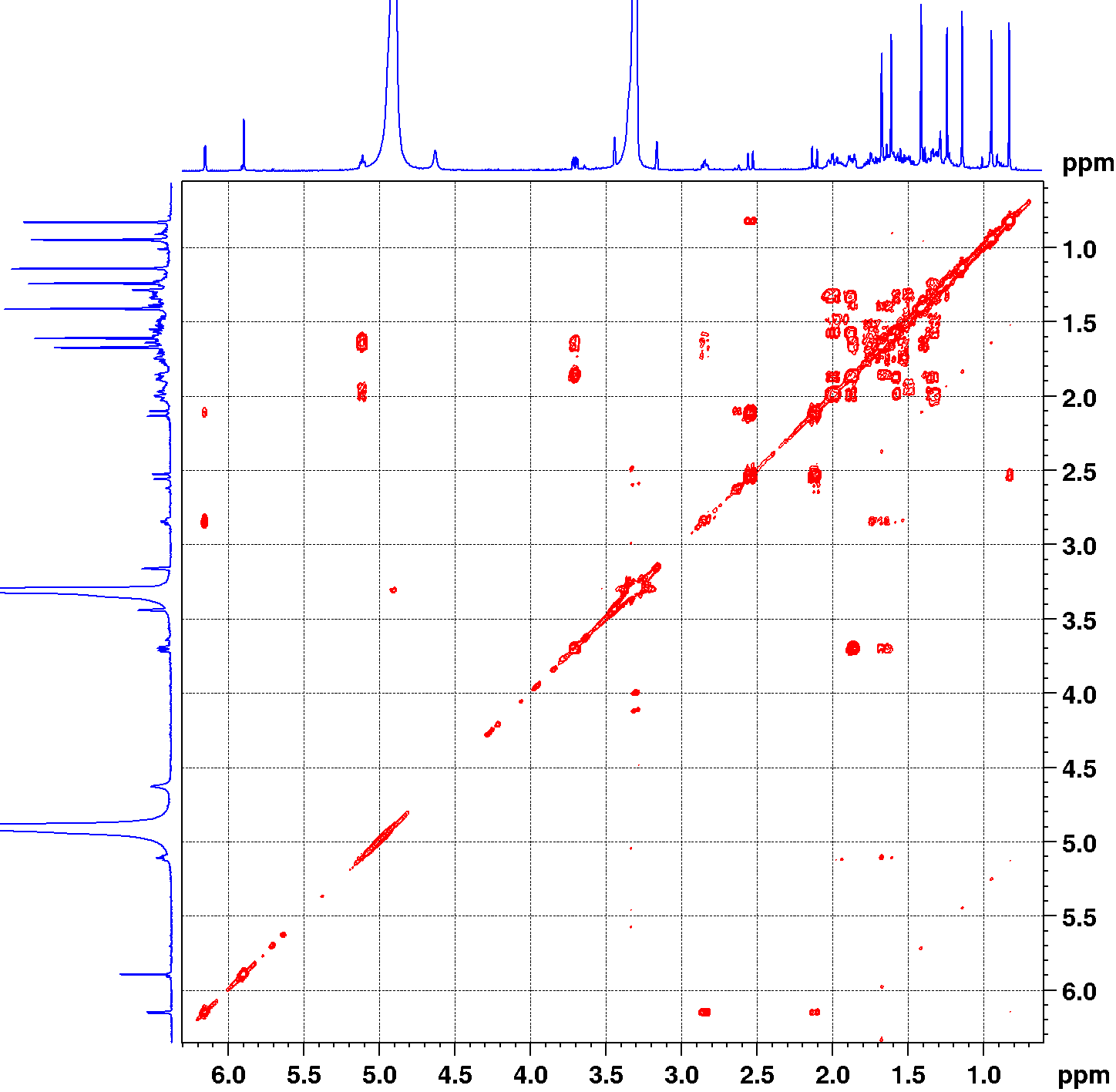
**

## Figure S17. COSY spectrum (500 MHz) of Gromomycin B (3) in CD_3_OD.

**
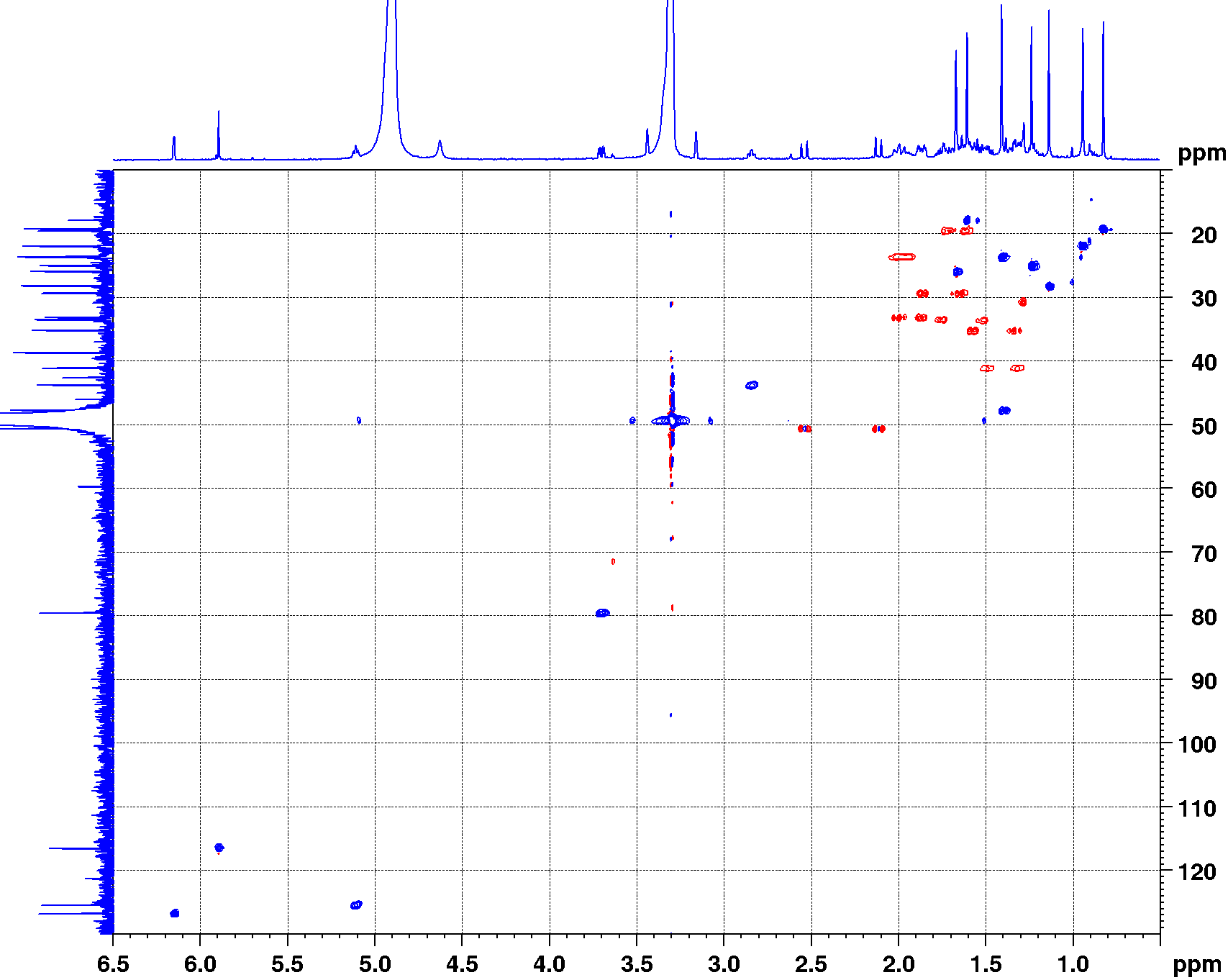
**

## Figure S18. ^1^H-^13^C HSQC spectrum (500 MHz) of Gromomycin B (3) in CD_3_OD.

**
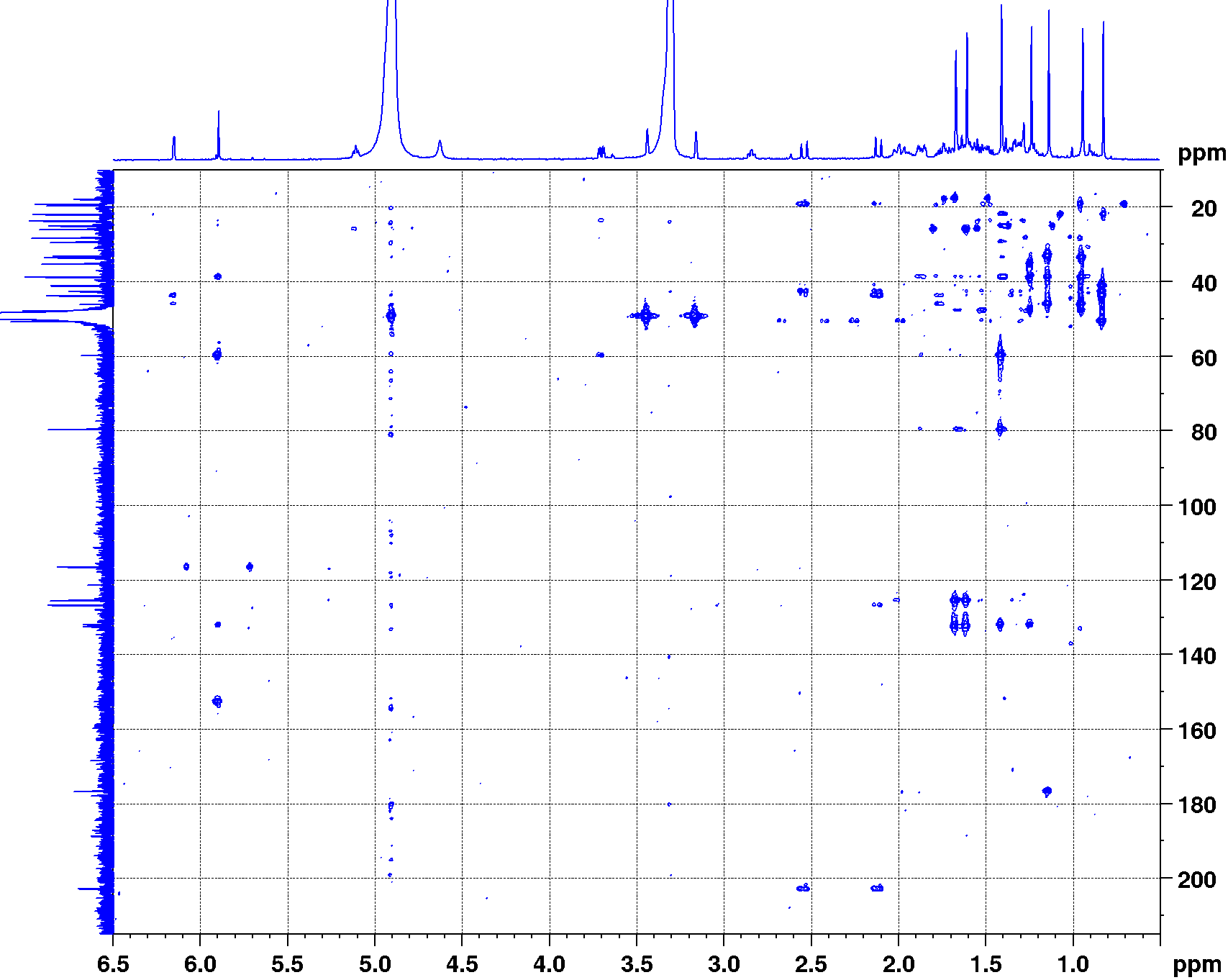
**

## Figure S19. ^1^H-^13^C HMBC spectrum (500 MHz) of Gromomycin B (3) in CD_3_OD.

**
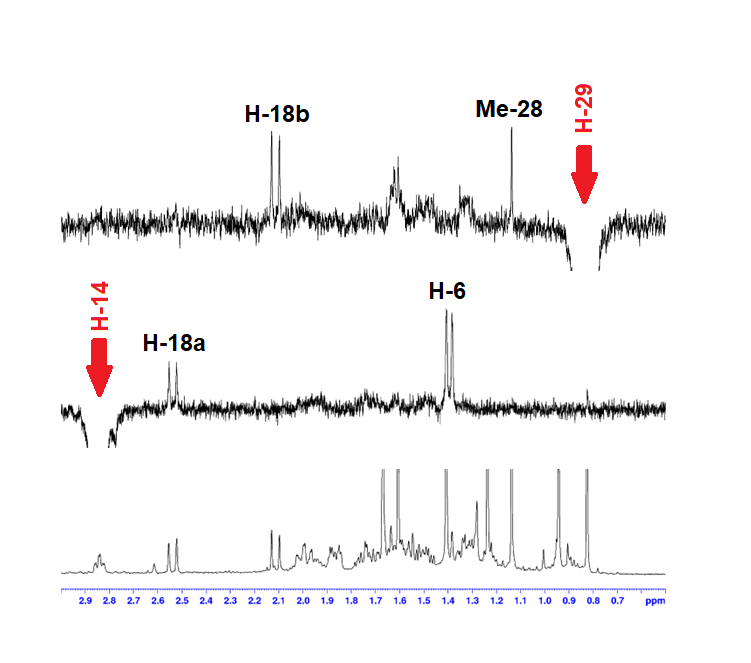
**

## Figure S20. 1D selective NOESY´s of gromomycin B (3) with excitations of H-14 (δ_H_ 2.84) and H-29 (δ_H_ 0.85).


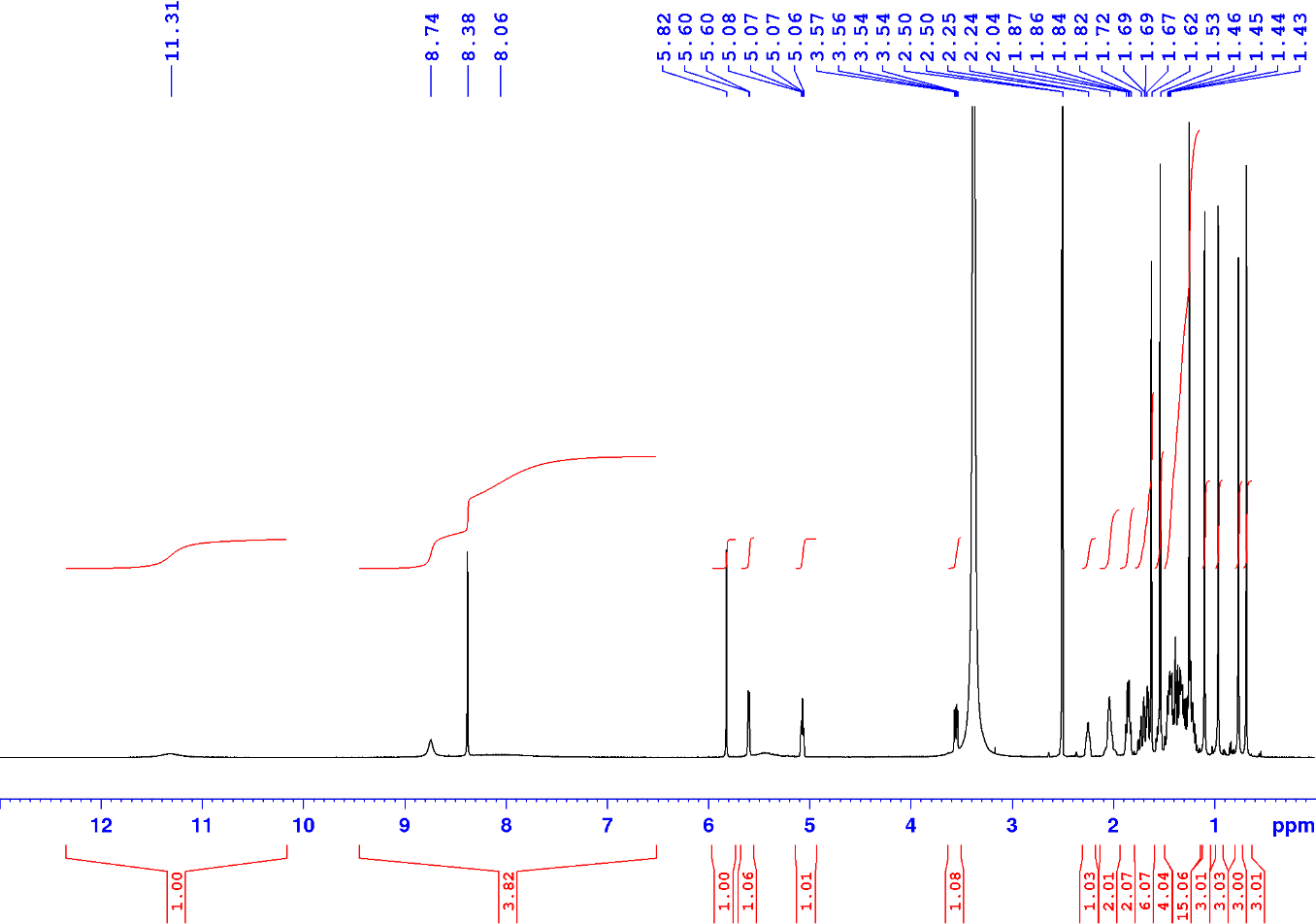


## Figure S21. ^1^H NMR spectrum (500 MHz) of Gromomycin C (4) in DMSO-d_6._


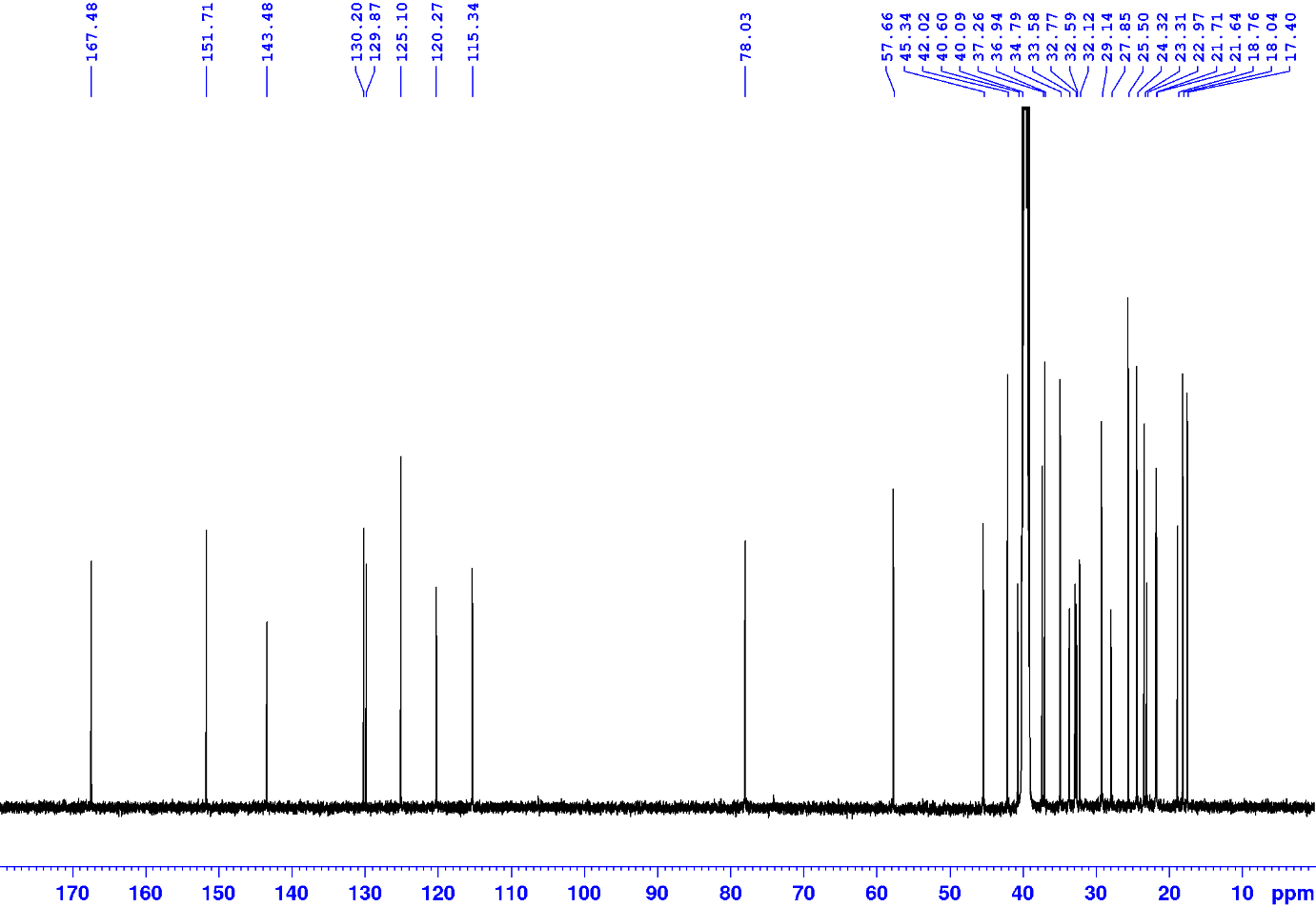


## Figure S22. ^13^C NMR spectrum (125 MHz) of Gromomycin C (4) in DMSO-d_6._

**
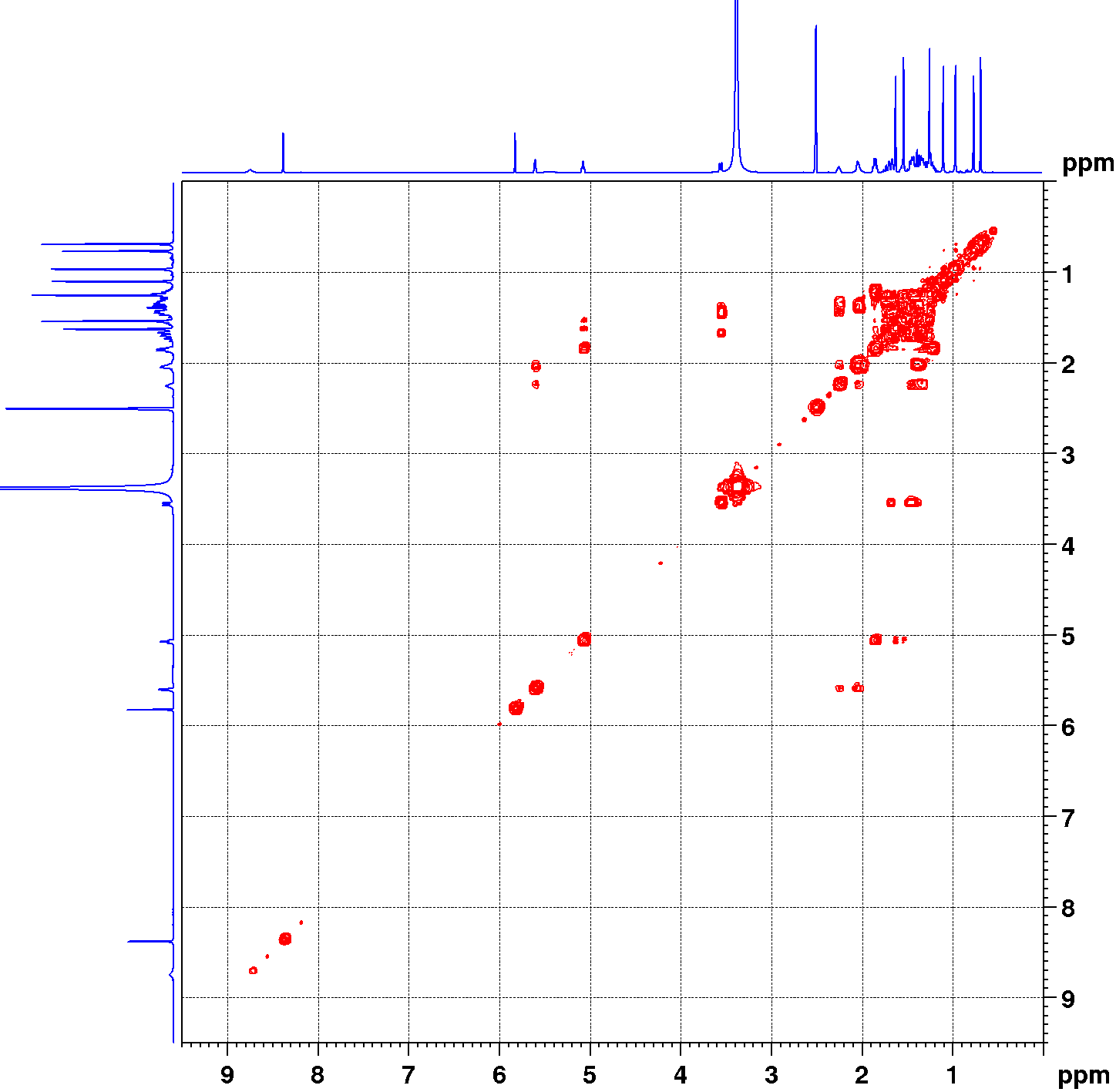
**

## Figure S23. COSY spectrum (500 MHz) of Gromomycin C (4) in DMSO-d_6._

**
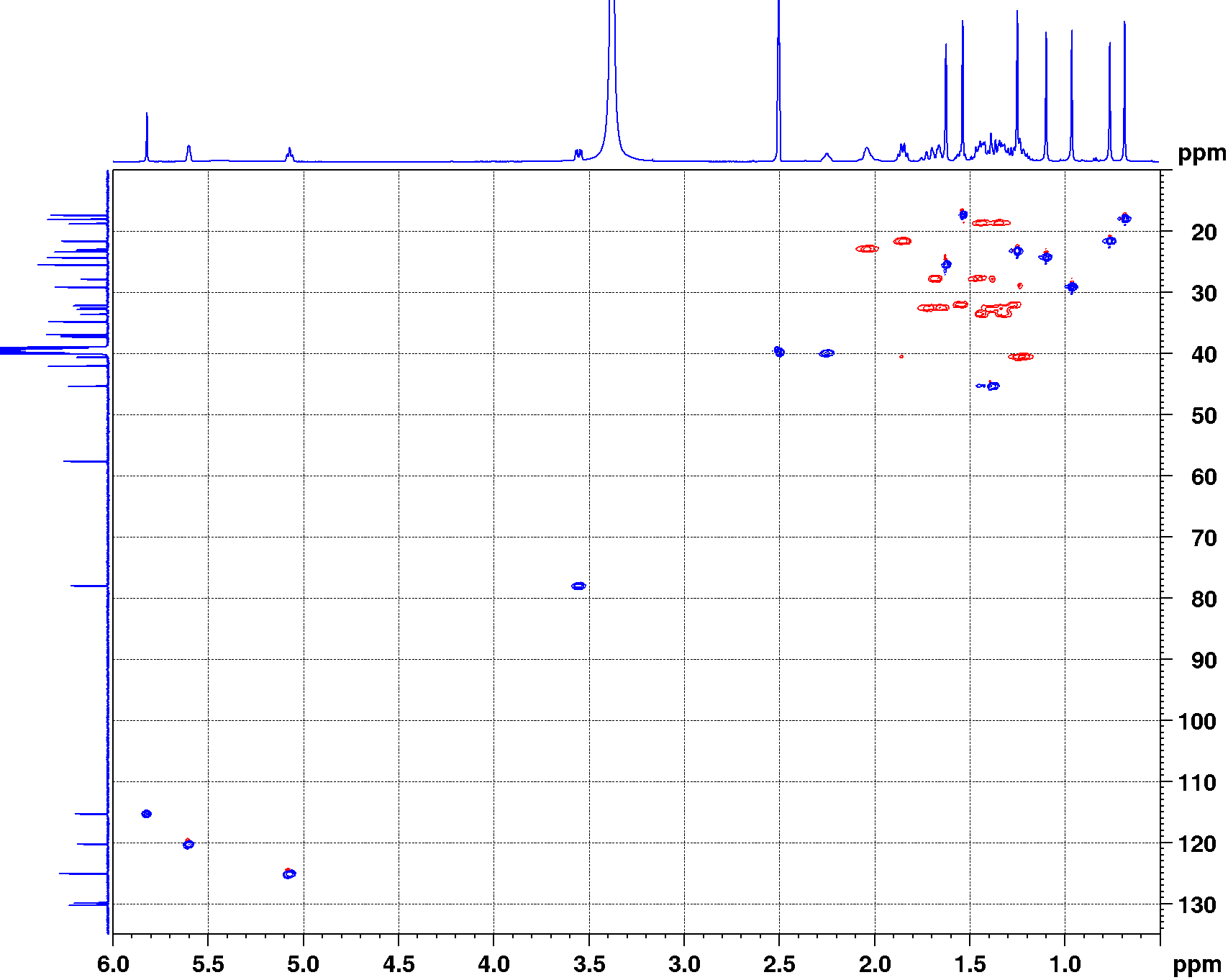
**

## Figure S24. ^1^H-^13^C HSQC spectrum (500 MHz) of Gromomycin C (4) in DMSO-d_6._

**
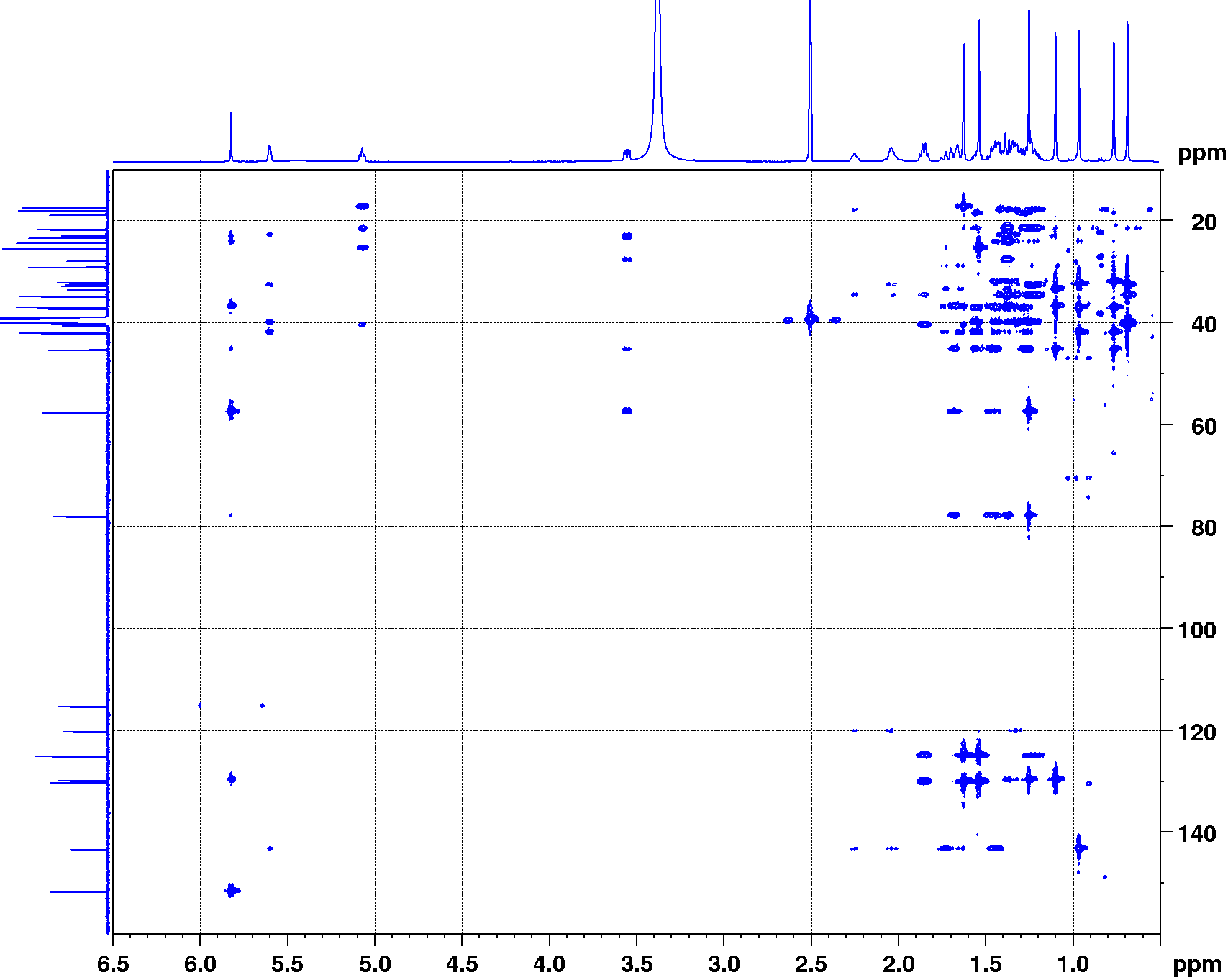
**

## Figure S25. ^1^H-^13^C HMBC spectrum (500 MHz) of Gromomycin C (4) in DMSO-d_6._


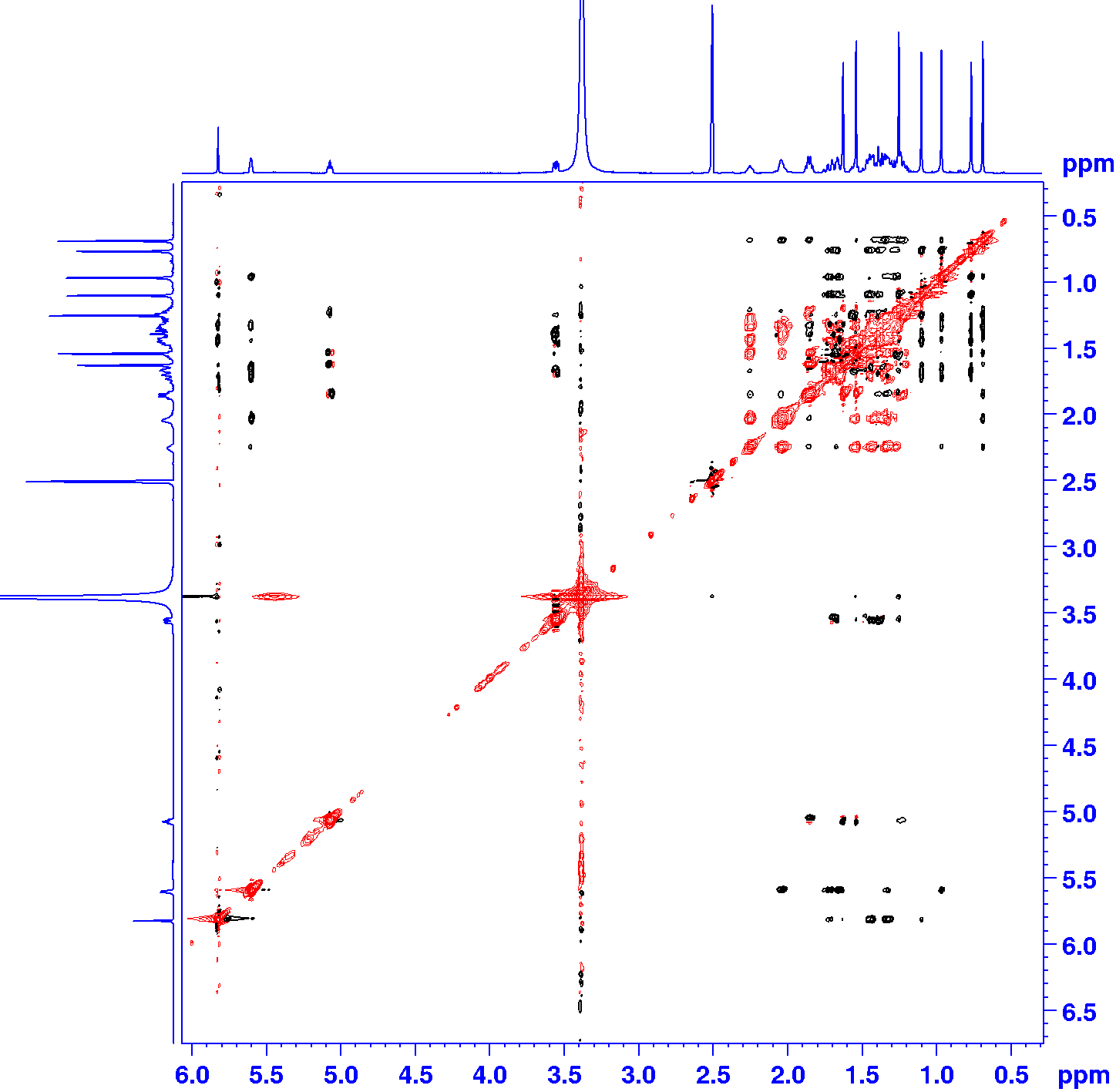


## Figure S26. ROESY spectrum (500 MHz) of Gromomycin C (4) in DMSO-d_6._


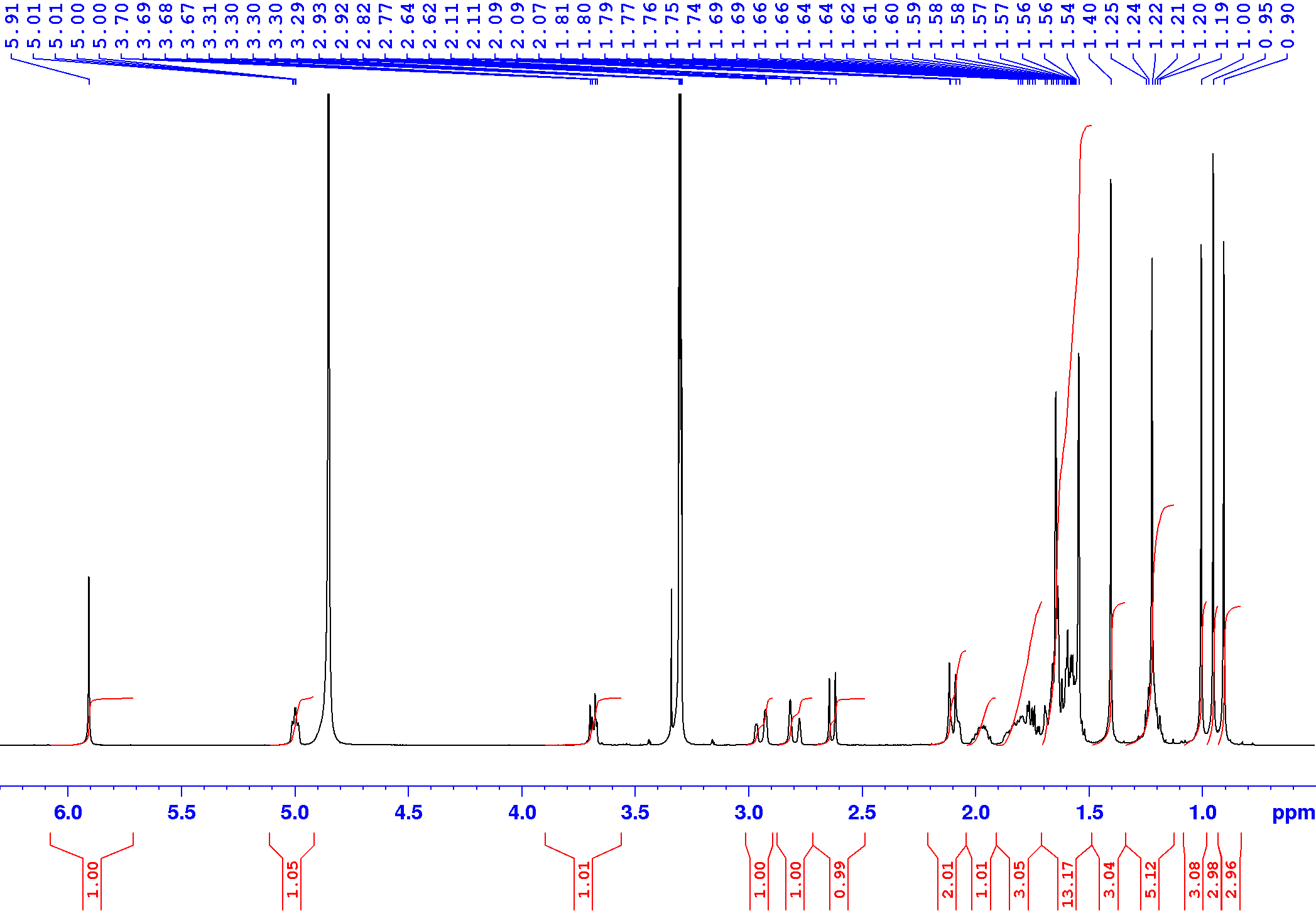


## Figure S27. ^1^H NMR spectrum (500 MHz) of Gromomycin D (5) in CD_3_OD.

**
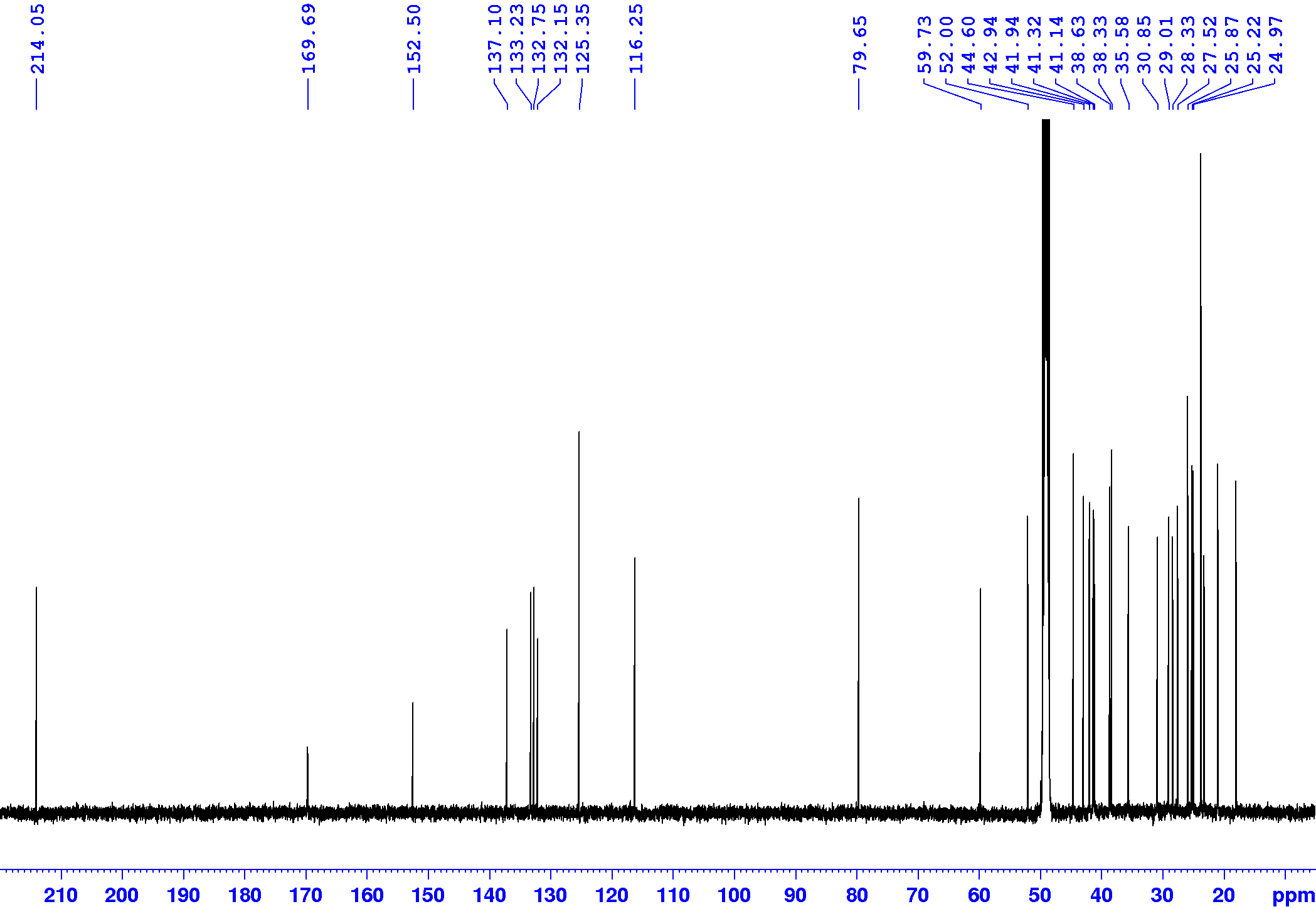
**

## Figure S28. ^13^C NMR spectrum (125 MHz) of Gromomycin D (5) in CD_3_OD.

_
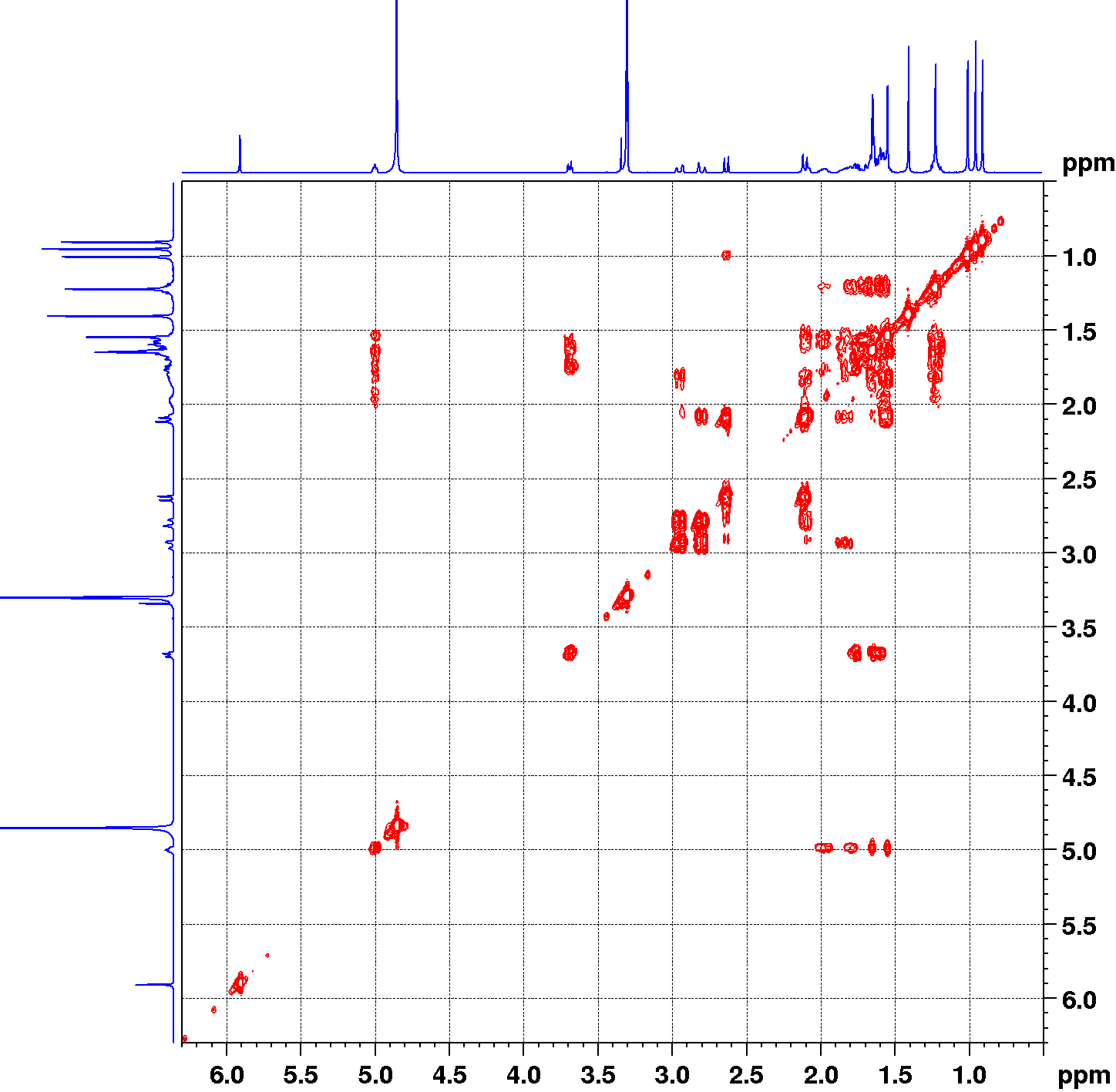
_

## Figure S29. COSY spectrum (500 MHz) of Gromomycin D (5) in CD_3_OD.


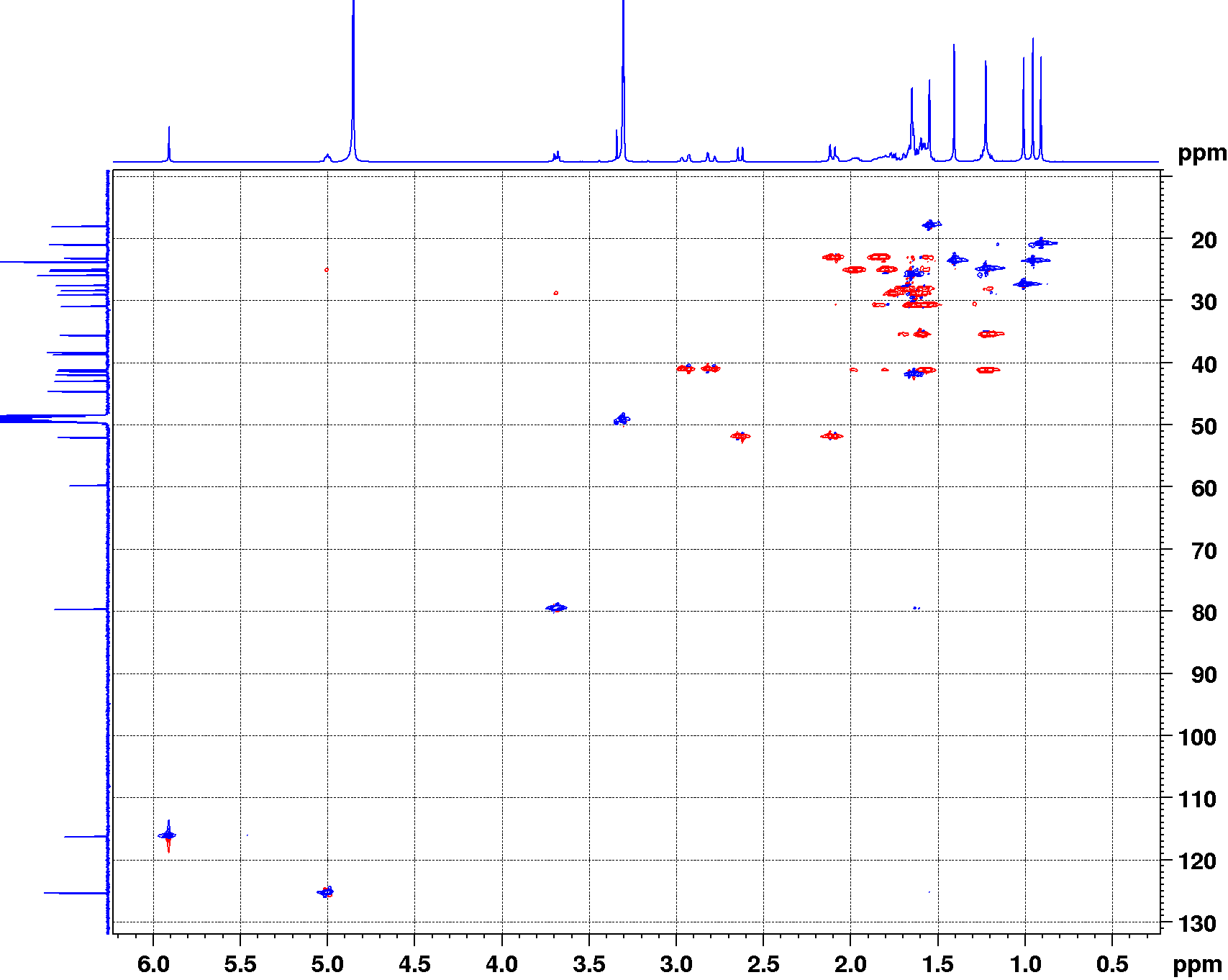


## Figure S30.^1^H-^13^C HSQC spectrum (500 MHz) of Gromomycin D (5) in CD_3_OD.

**
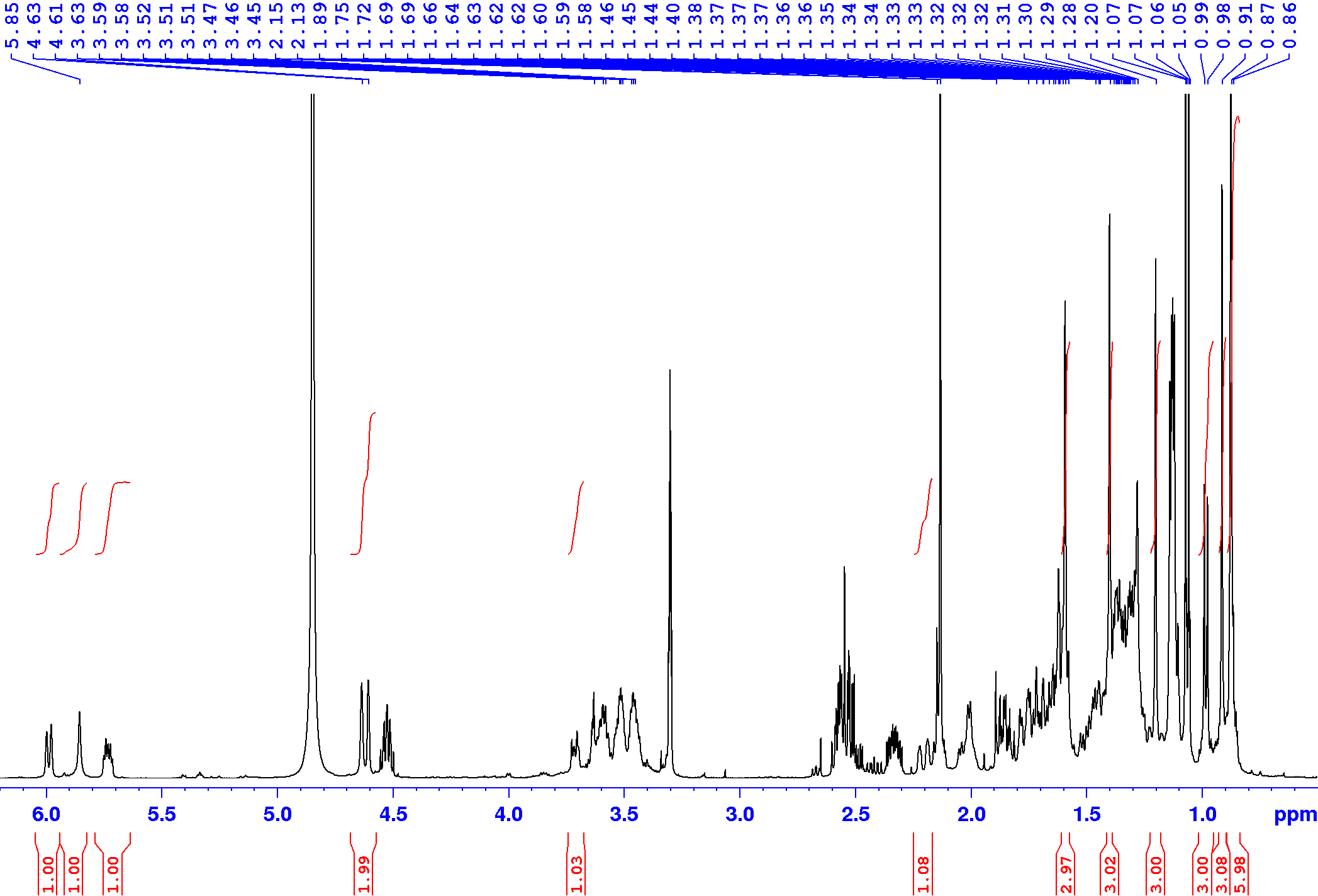
**

## Figure S31. ^1^H NMR spectrum (500 MHz) of Gromomycin E (6) in CD_3_OD.

**
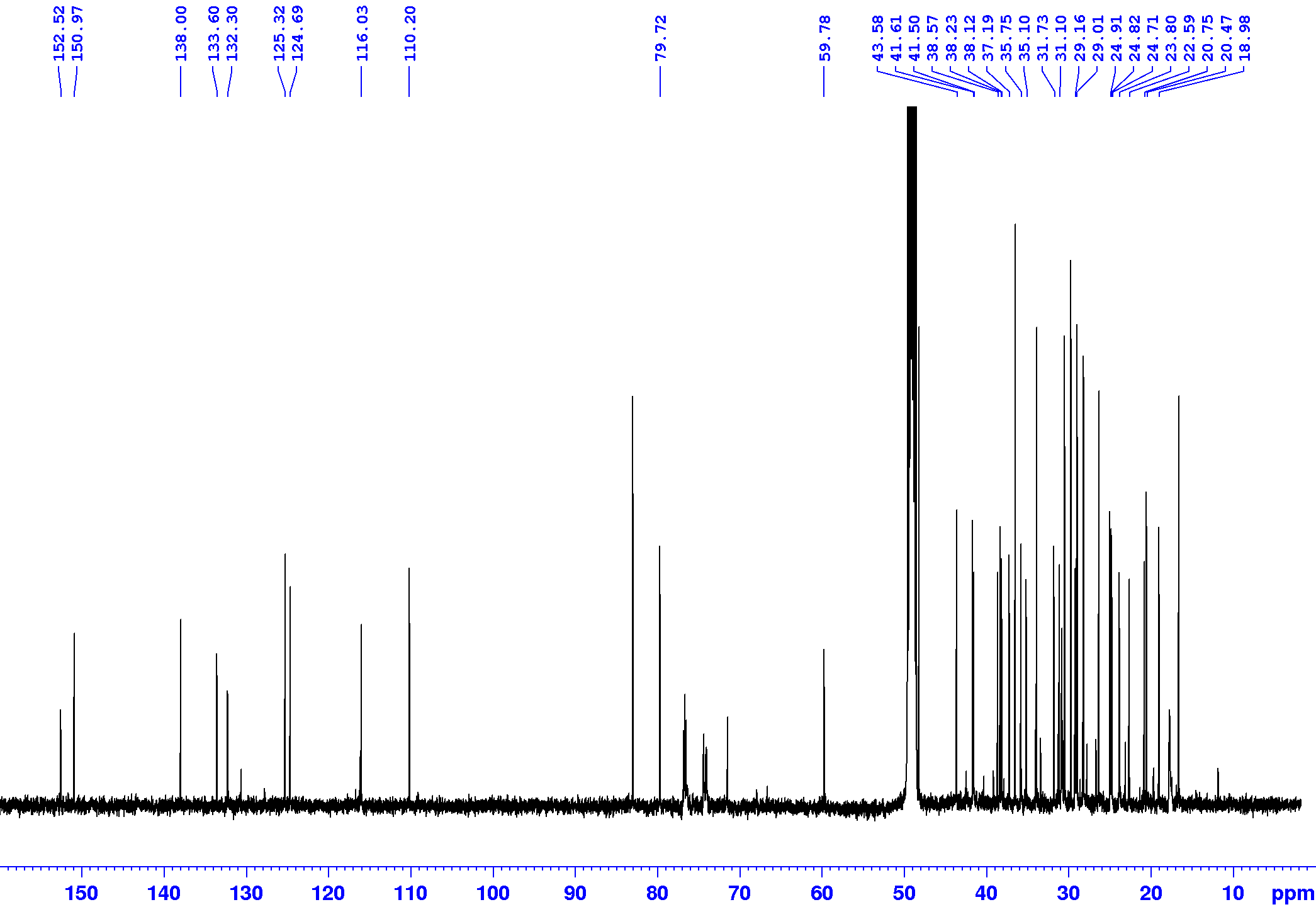
**

## Figure S32. ^13^C NMR spectrum (125 MHz) of Gromomycin E (6) in CD_3_OD.

_
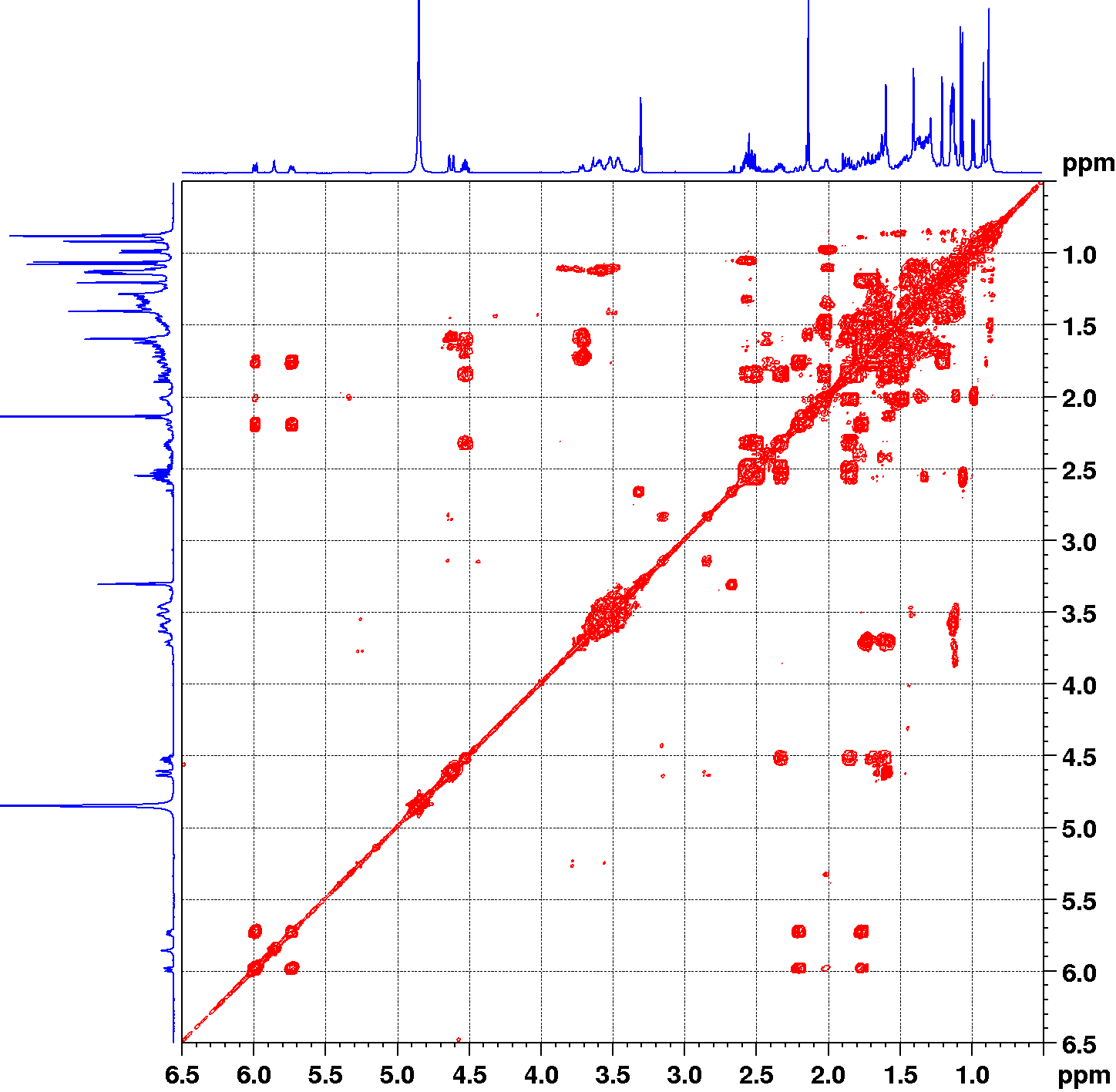
_

## Figure S33. COSY spectrum (500 MHz) of Gromomycin E (6) in CD_3_OD.

**
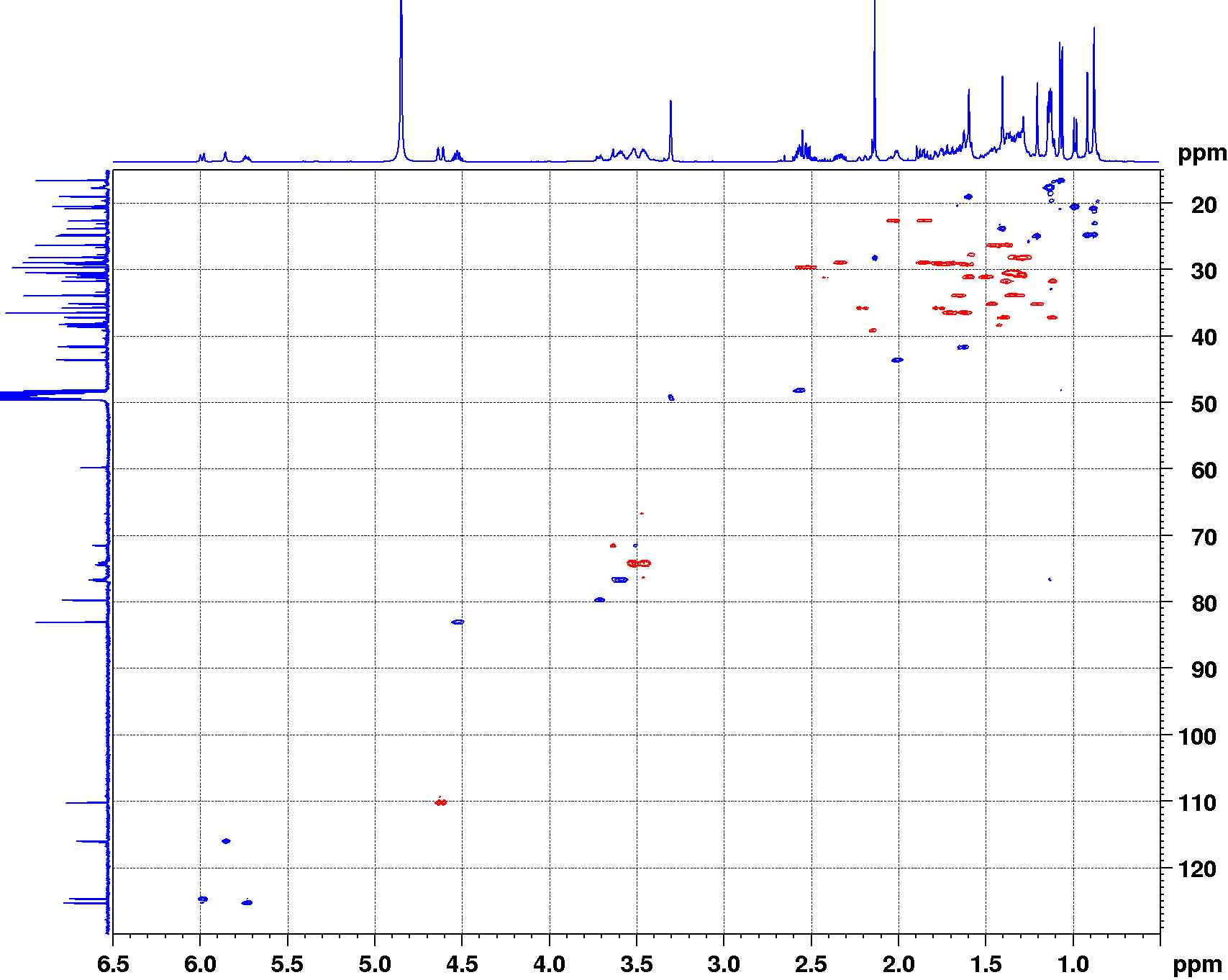
**

## Figure S34. ^1^H-^13^C HSQC spectrum (500 MHz) of Gromomycin E (6) in CD_3_OD.

_
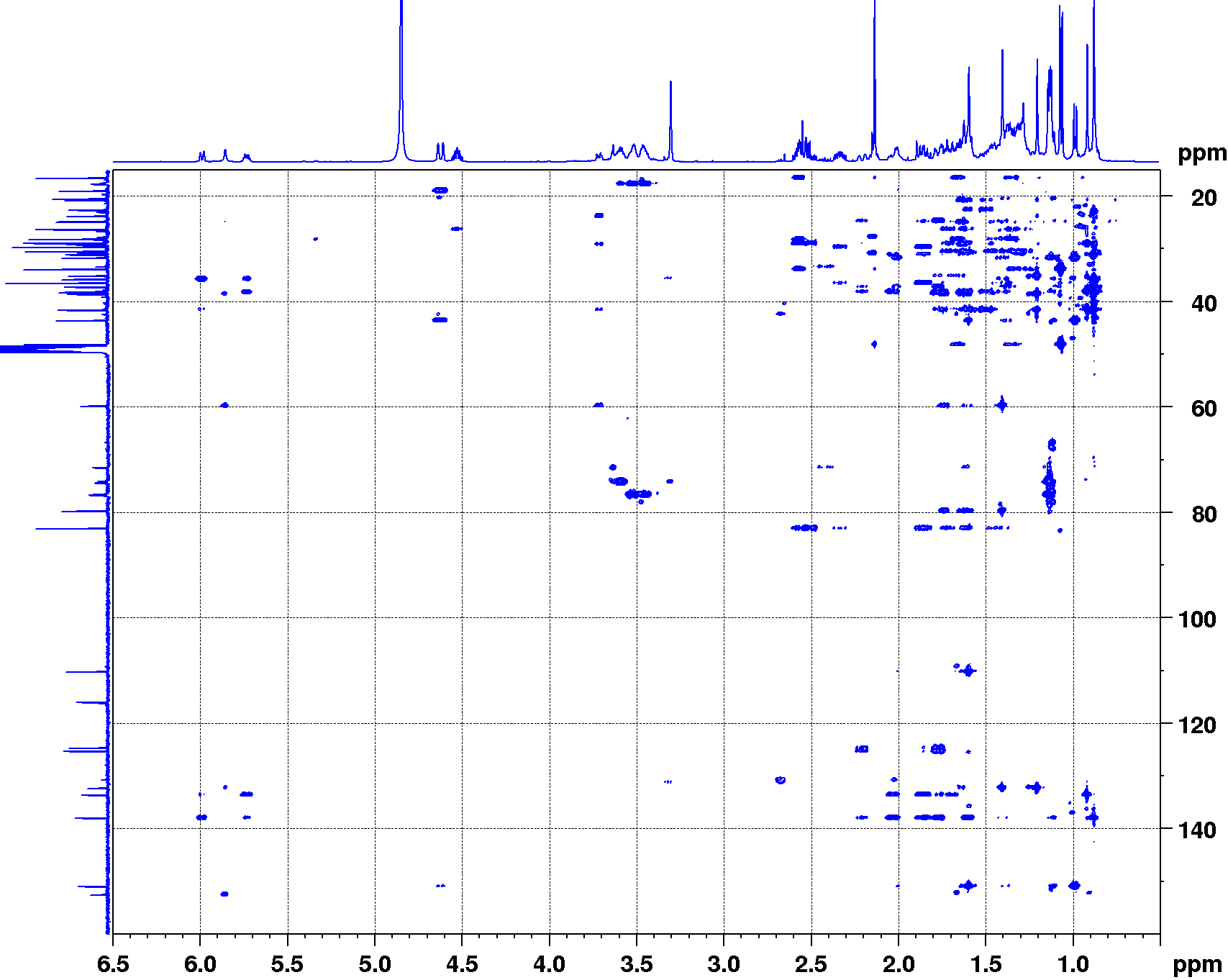
_

## Figure S35. ^1^H-^13^C HMBC spectrum (500 MHz) of Gromomycin E (6) in CD_3_OD.


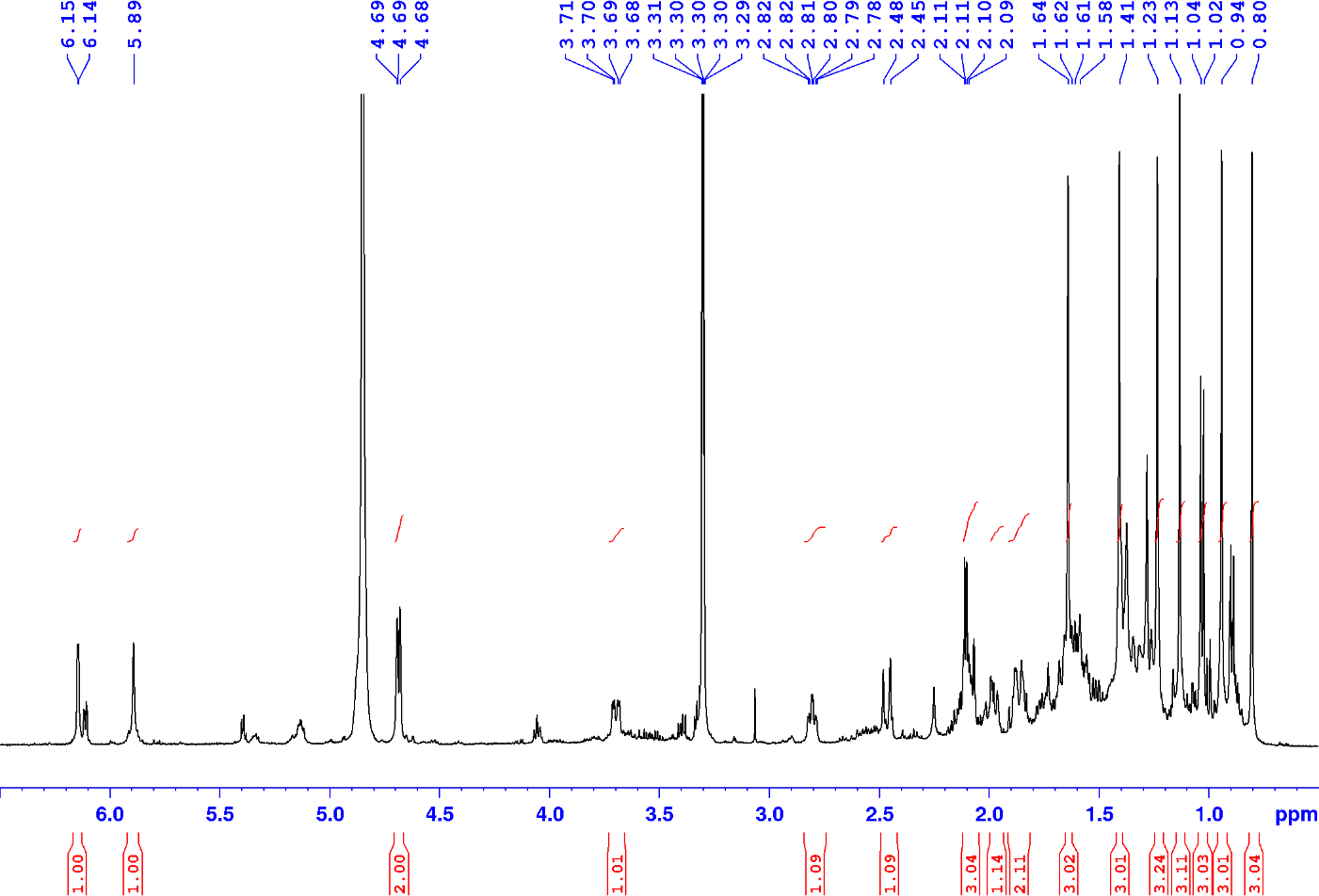


## Figure S36. ^1^H NMR spectrum (500 MHz) of Gromomycin F (7) in CD_3_OD_._

_
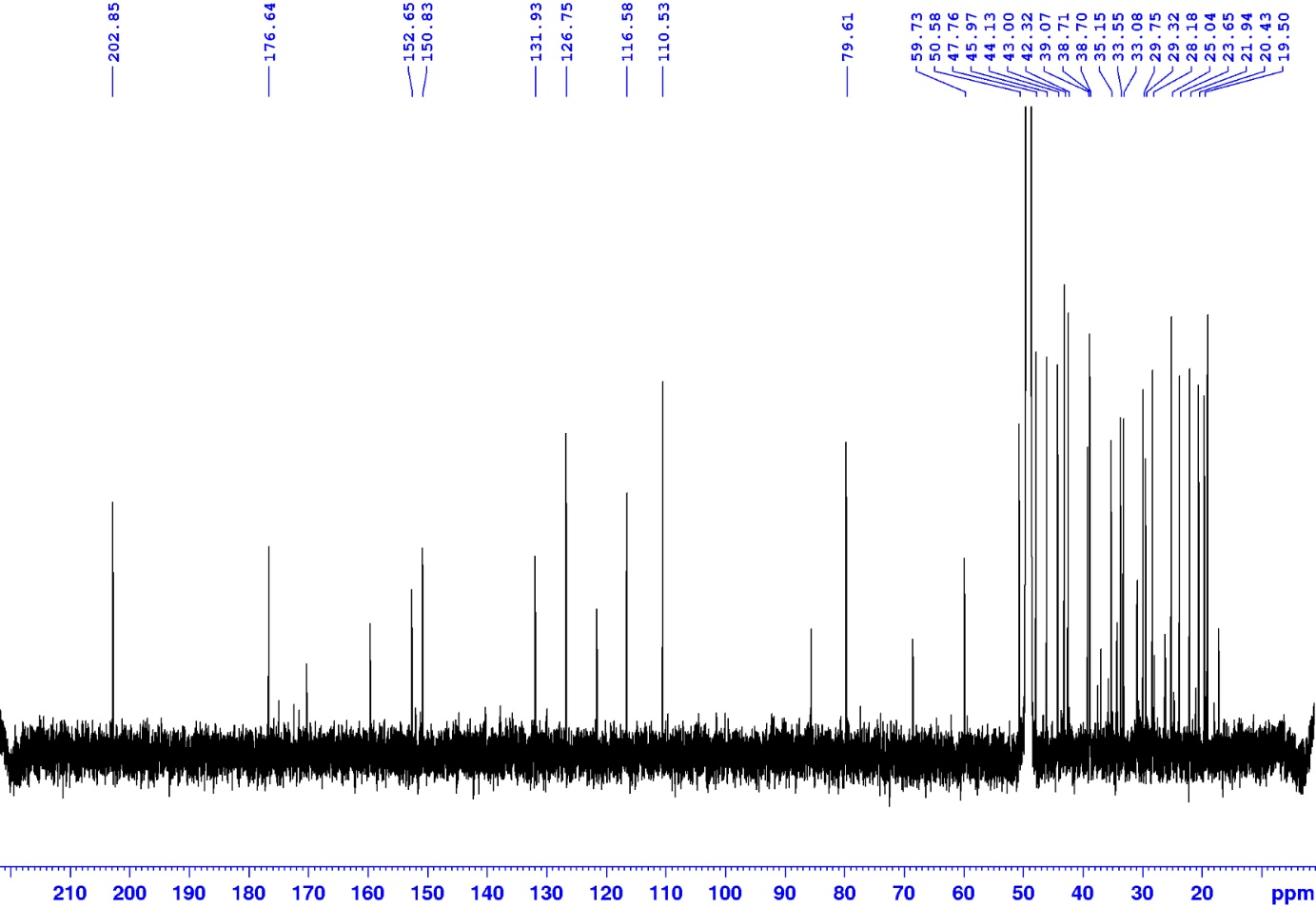
_

## Figure S37. ^13^C NMR spectrum (125 MHz) of Gromomycin F (7) in CD_3_OD.


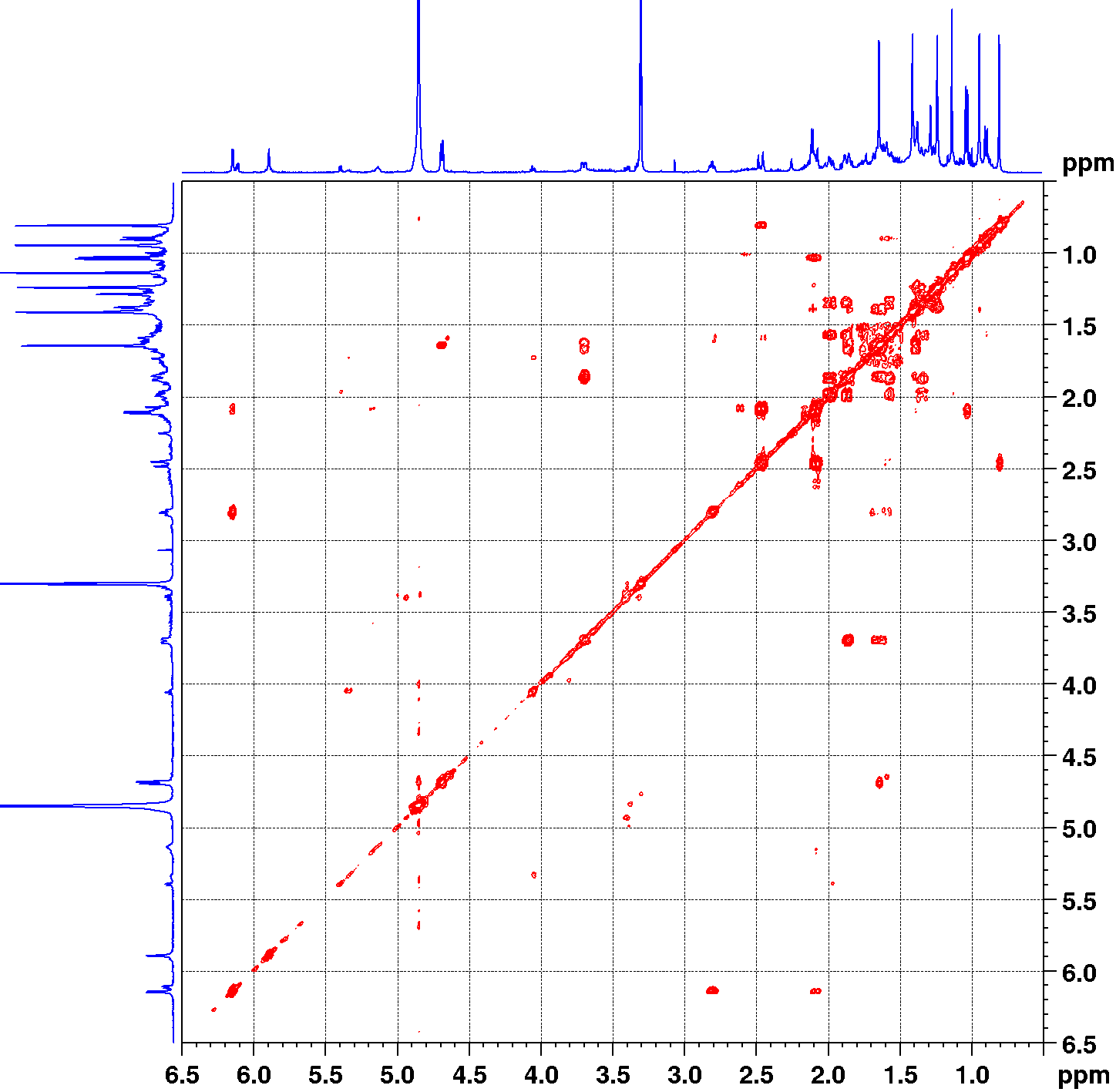


## Figure S38. COSY spectrum (500 MHz) of Gromomycin F (7) in CD_3_OD.


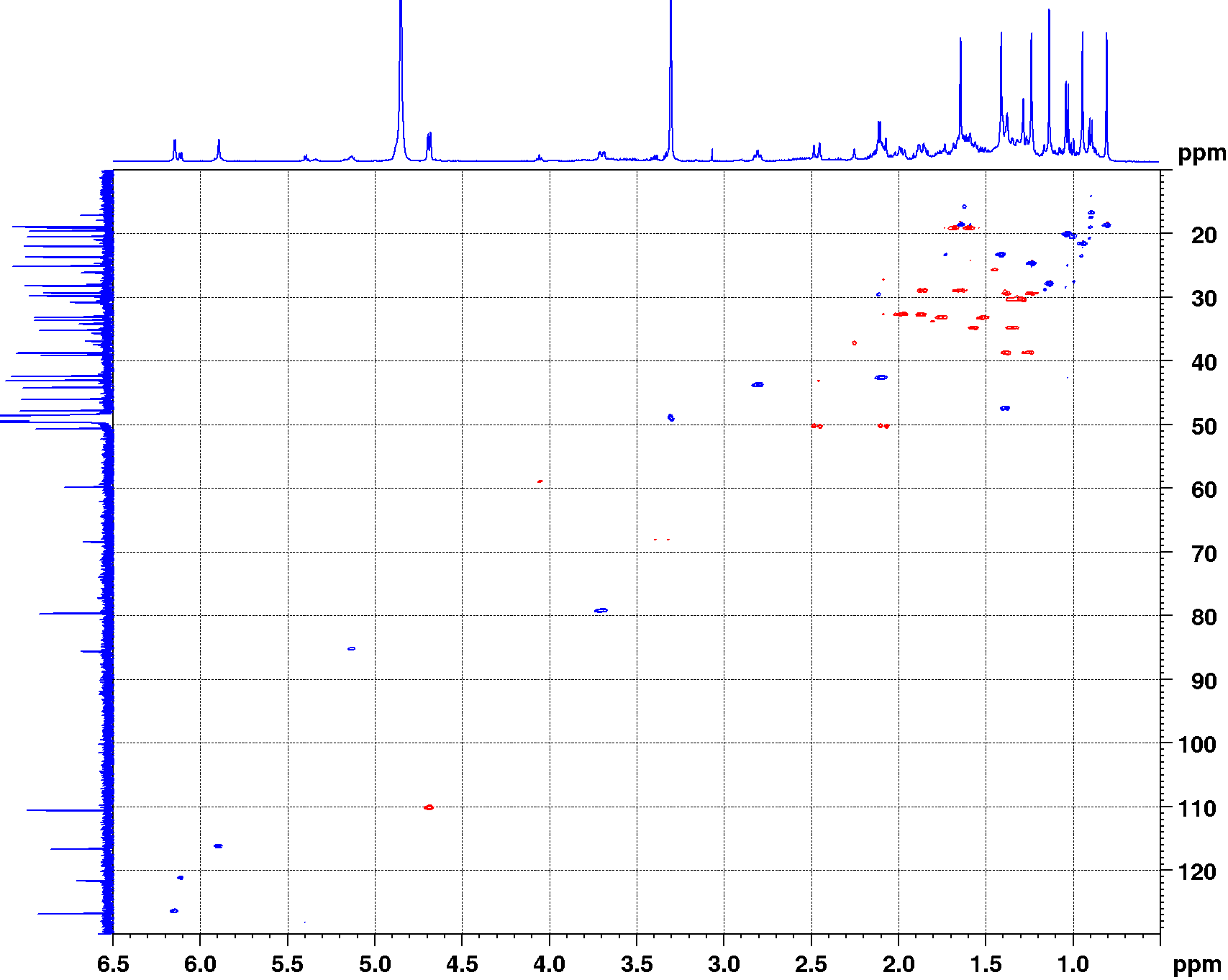


## Figure S39. ^1^H-^13^C HSQC spectrum (500 MHz) of Gromomycin F (7) in CD_3_OD.

**
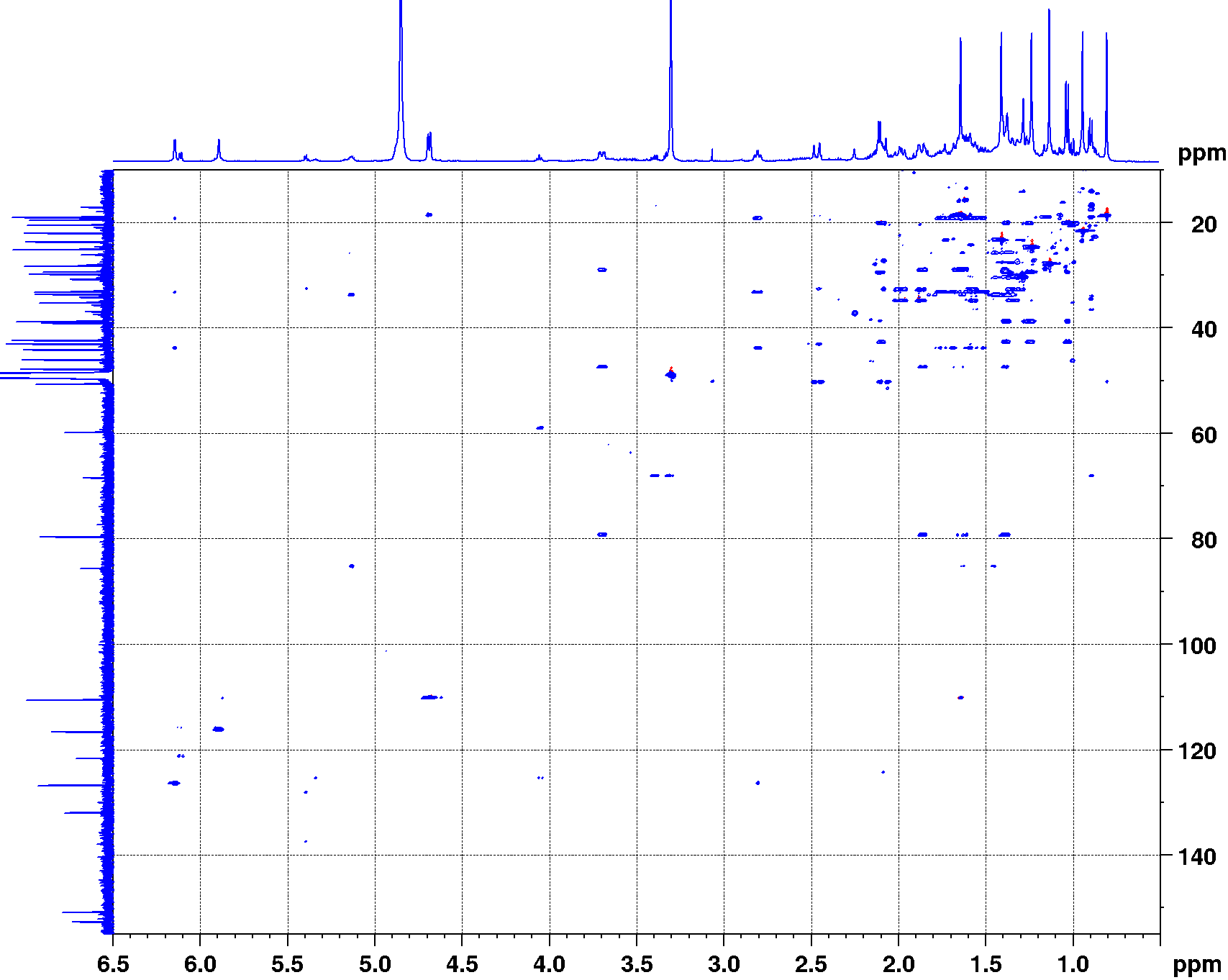
**

## Figure S40. ^1^H-^13^C HMBC spectrum (500 MHz) of Gromomycin F (7) in CD_3_OD.


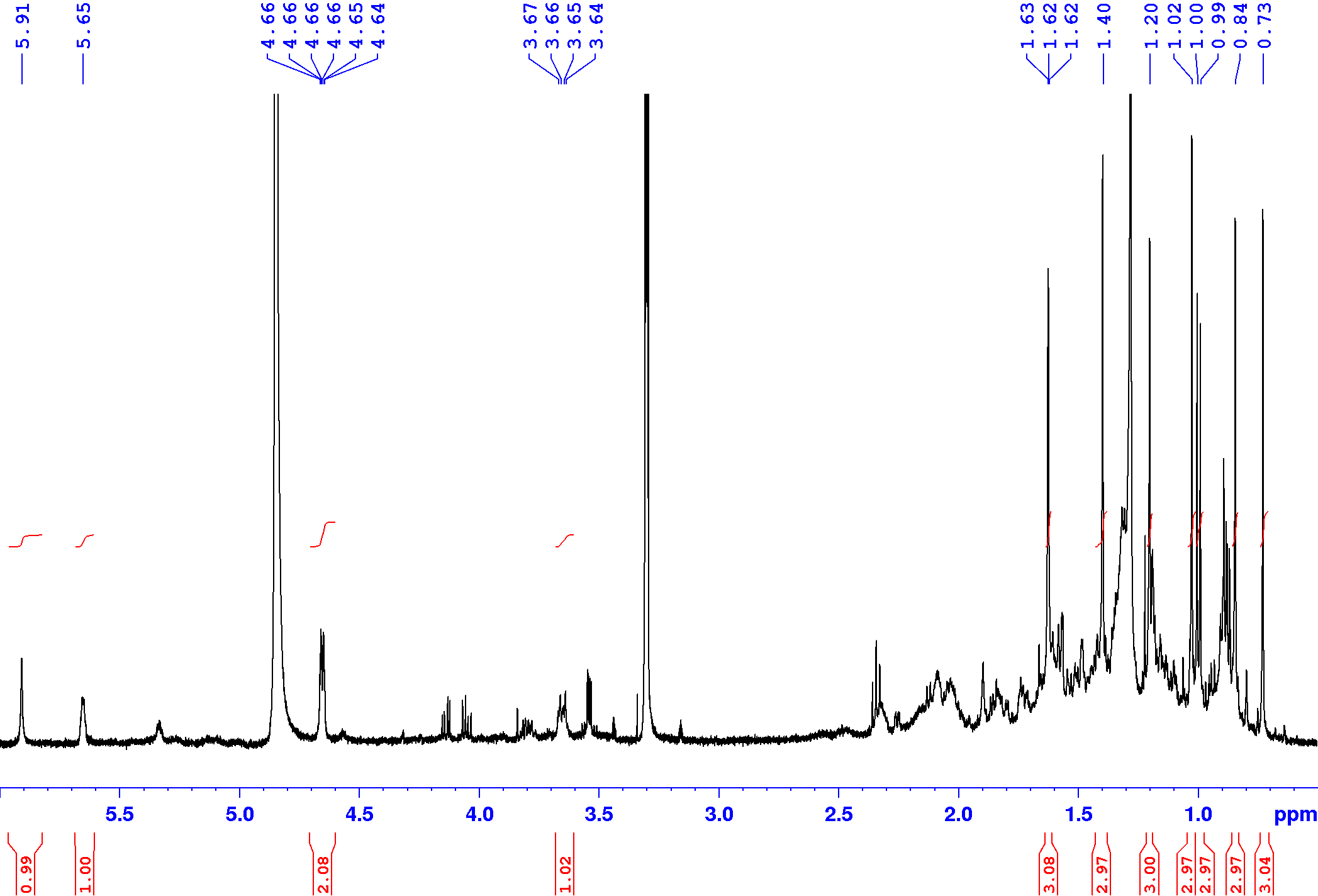


## Figure S41. ^1^H NMR spectrum (500 MHz) of Gromomycin G (8) in CD_3_OD.


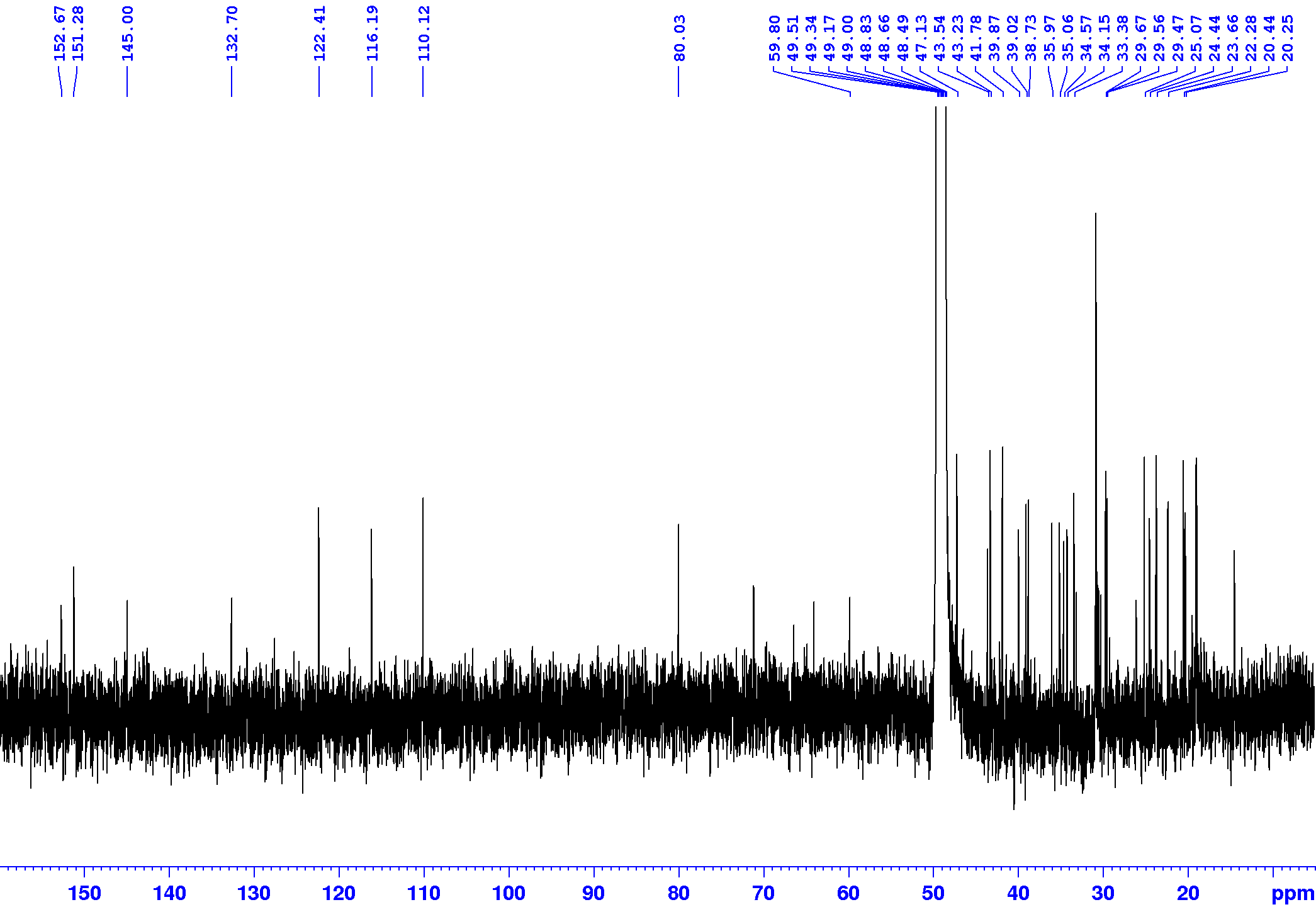


## Figure S42. ^13^C NMR spectrum (125 MHz) of Gromomycin G (8) in CD_3_OD.


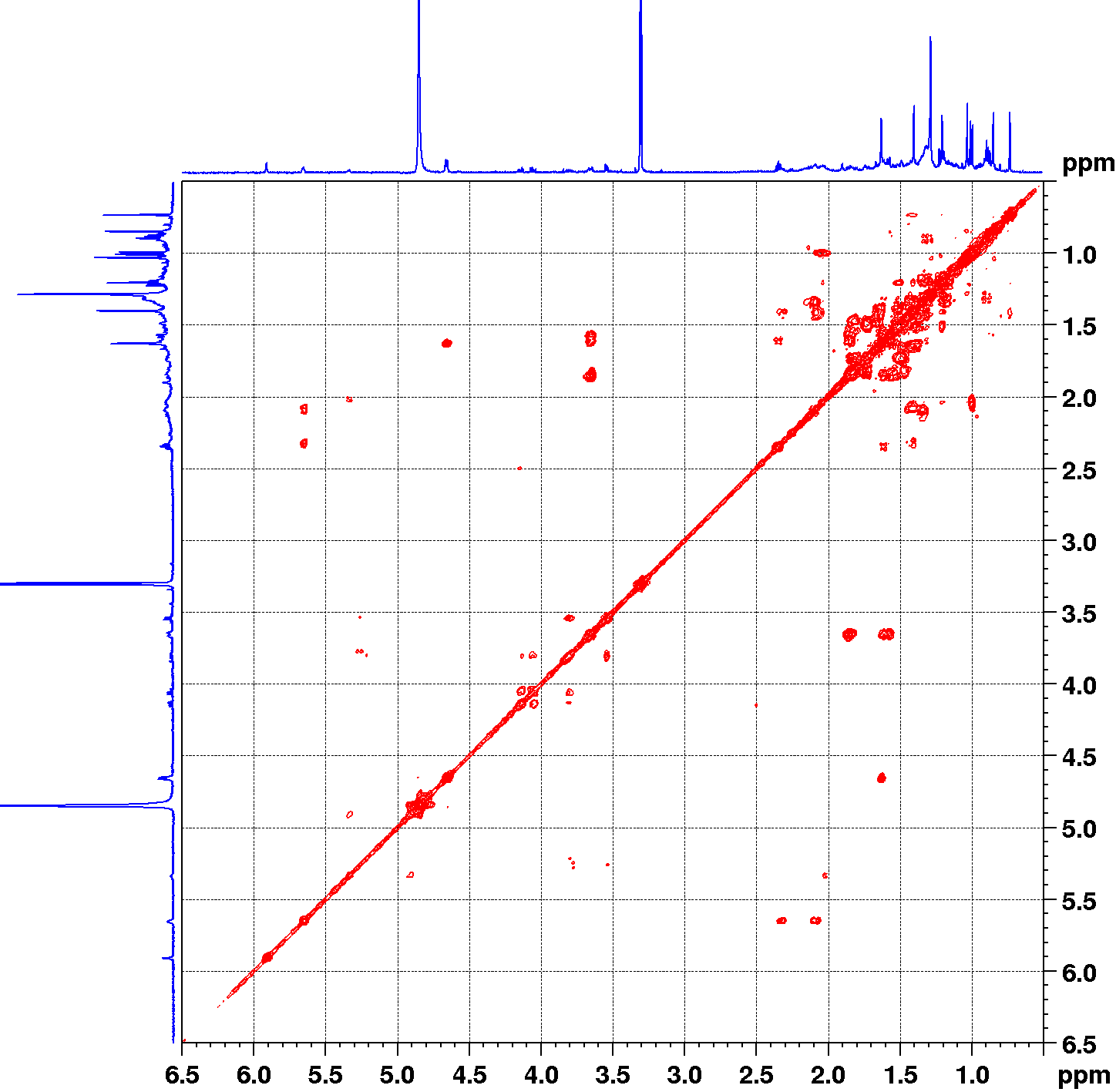


##
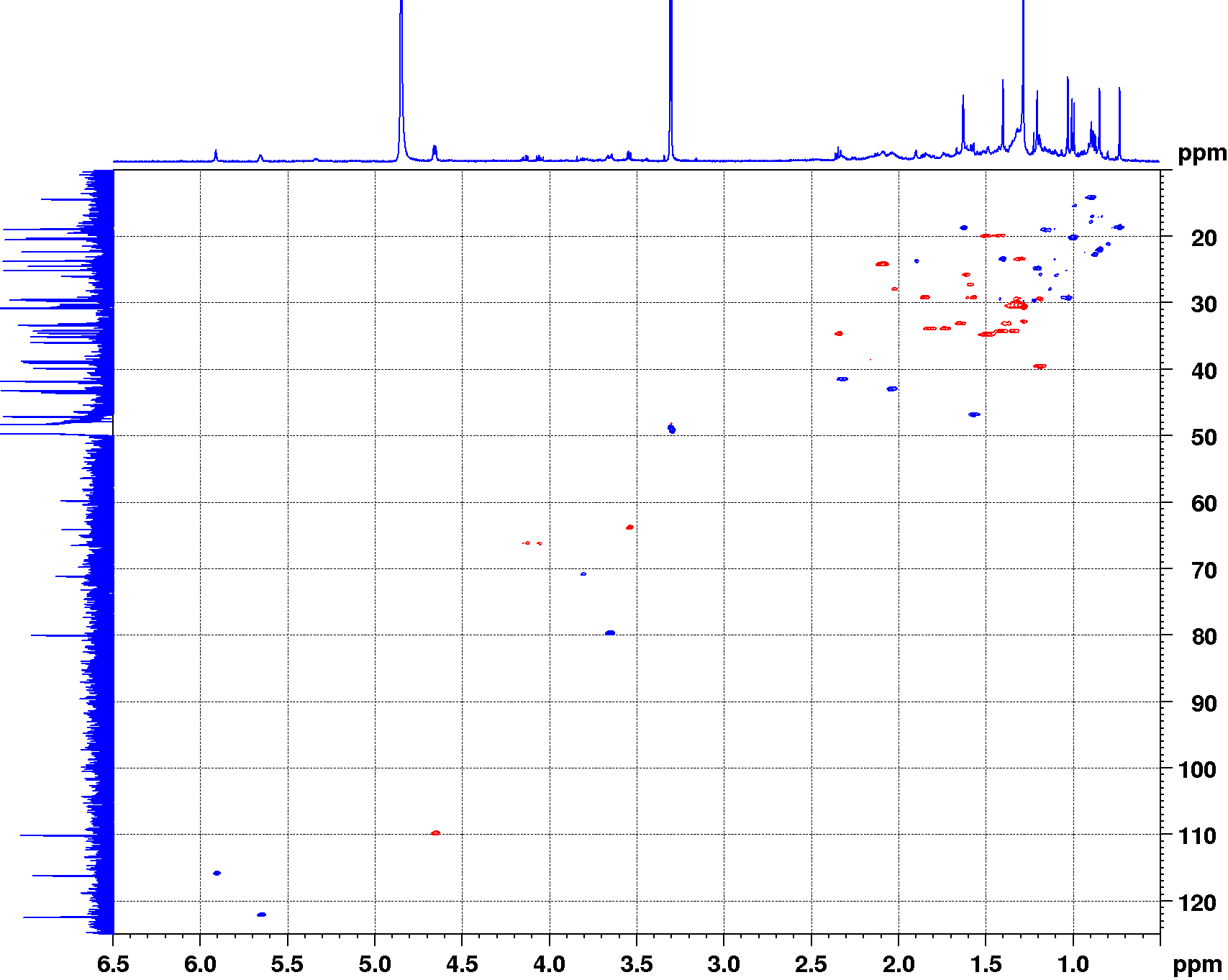
Figure S43. COSY spectrum (500 MHz) of Gromomycin G (8) in CD_3_OD.

## Figure S44. ^1^H-^13^C HSQC spectrum (500 MHz) of Gromomycin G (8) in CD_3_OD.


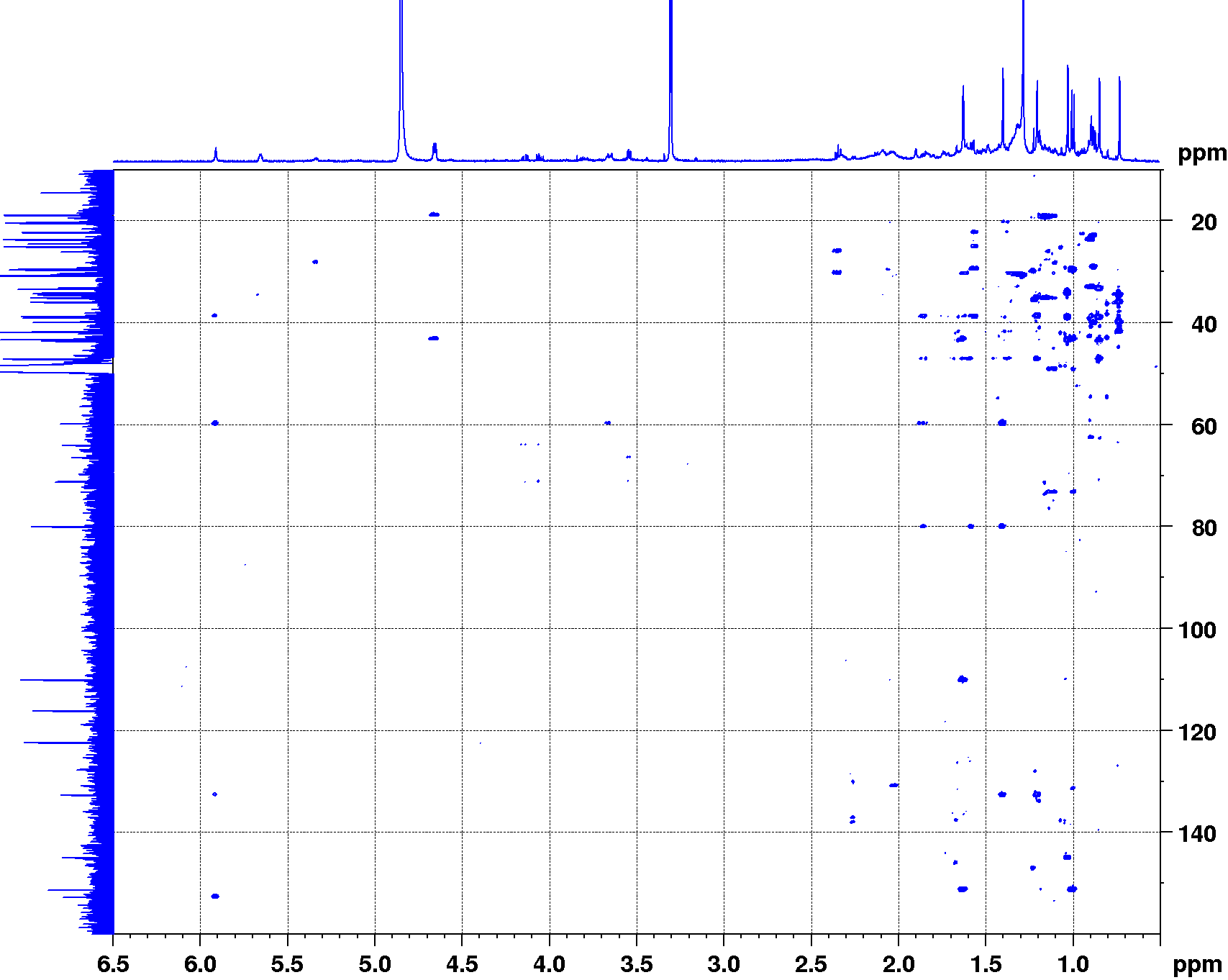


## Figure S45. ^1^H-^13^C HMBC spectrum (500 MHz) of Gromomycin G (8) in CD_3_OD.


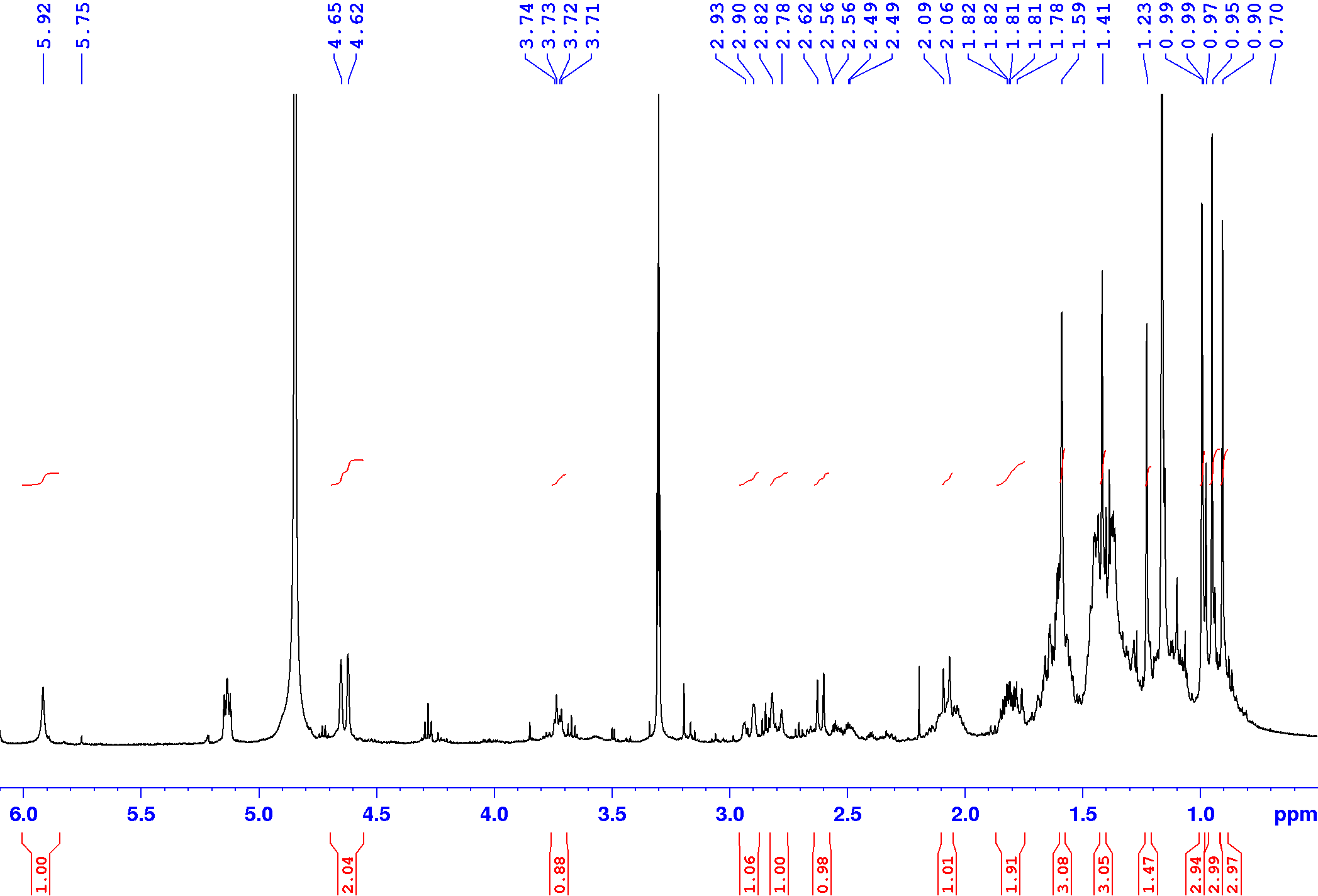


## Figure S46. ^1^H NMR spectrum (500 MHz) of Gromomycin H (9) in CD_3_OD.

**
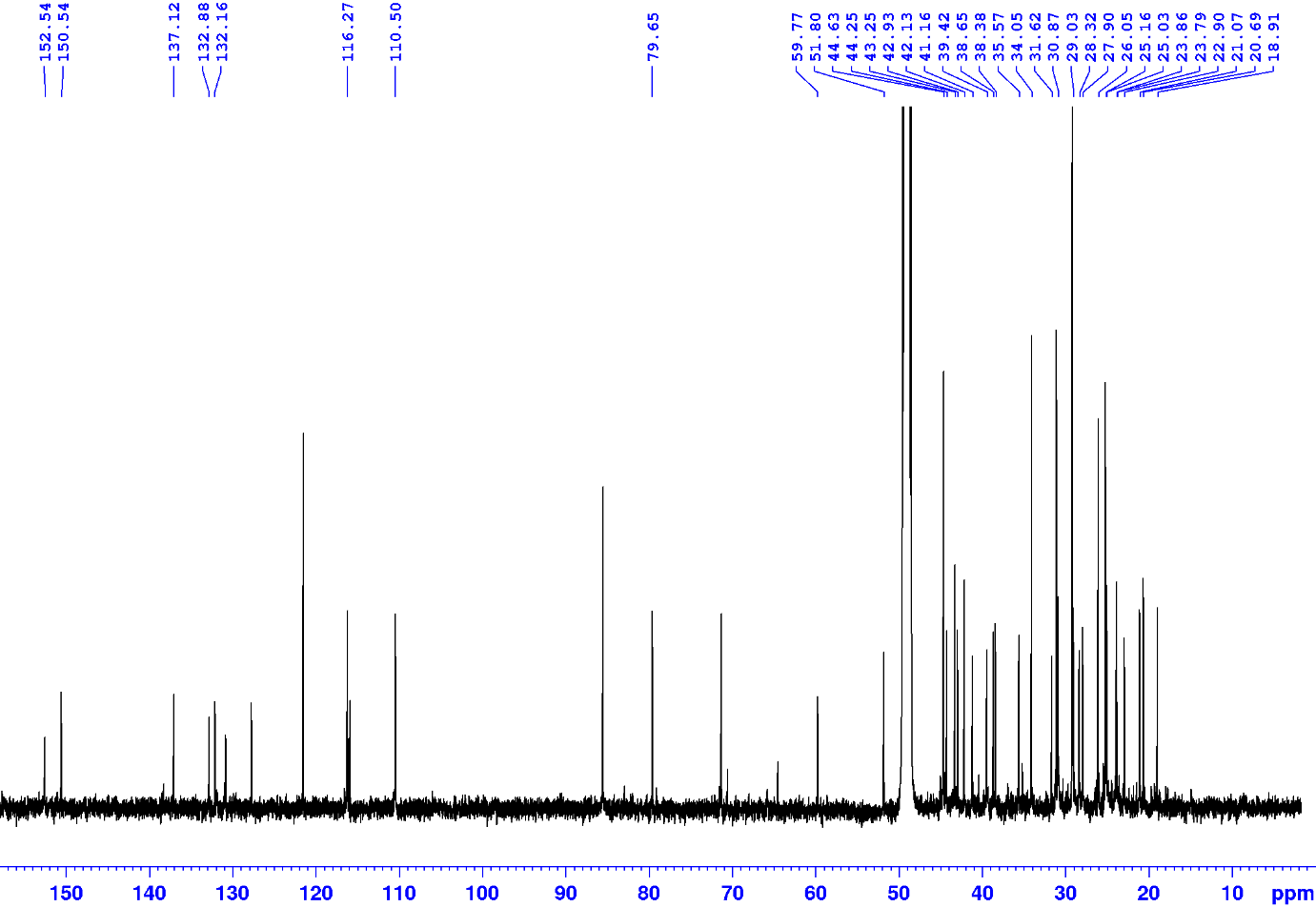
**

## Figure S47. ^13^C NMR spectrum (125 MHz) of Gromomycin H (9) in CD_3_OD.

**
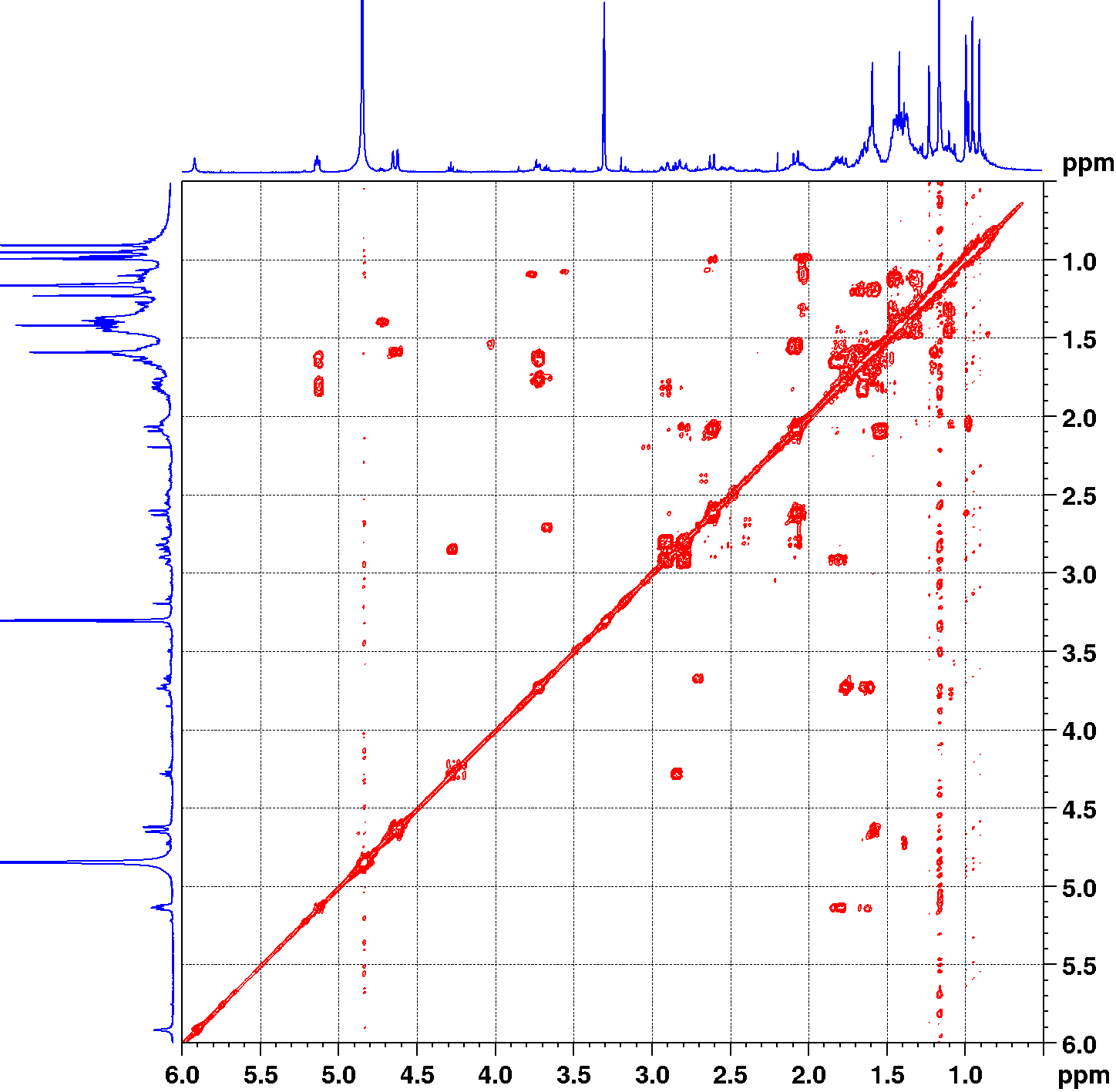
**

## Figure S48. COSY spectrum (500 MHz) of Gromomycin H (9) in CD_3_OD.

**
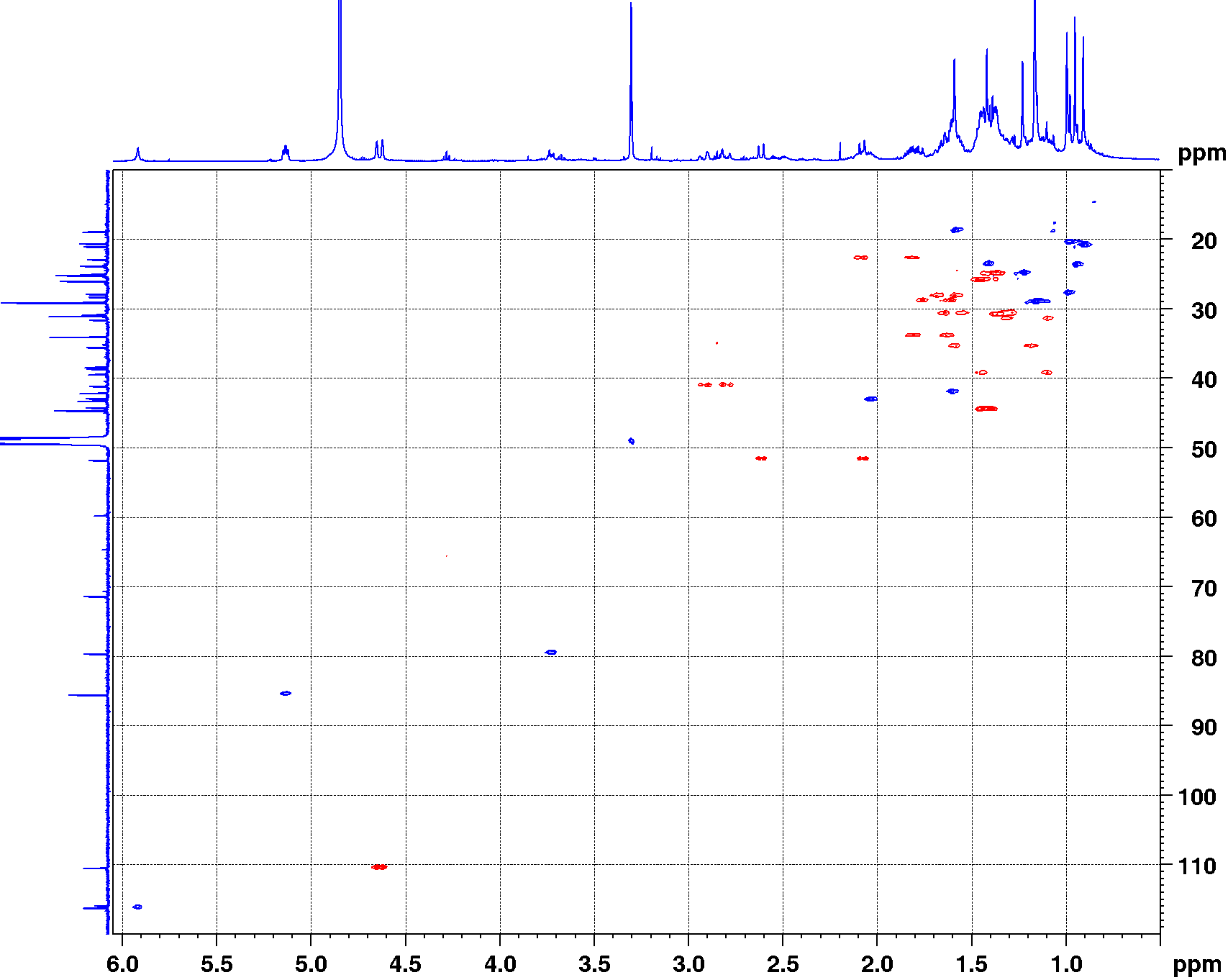
**

## Figure S49. ^1^H-^13^C HSQC spectrum (500 MHz) of Gromomycin H (9) in CD_3_OD.

**
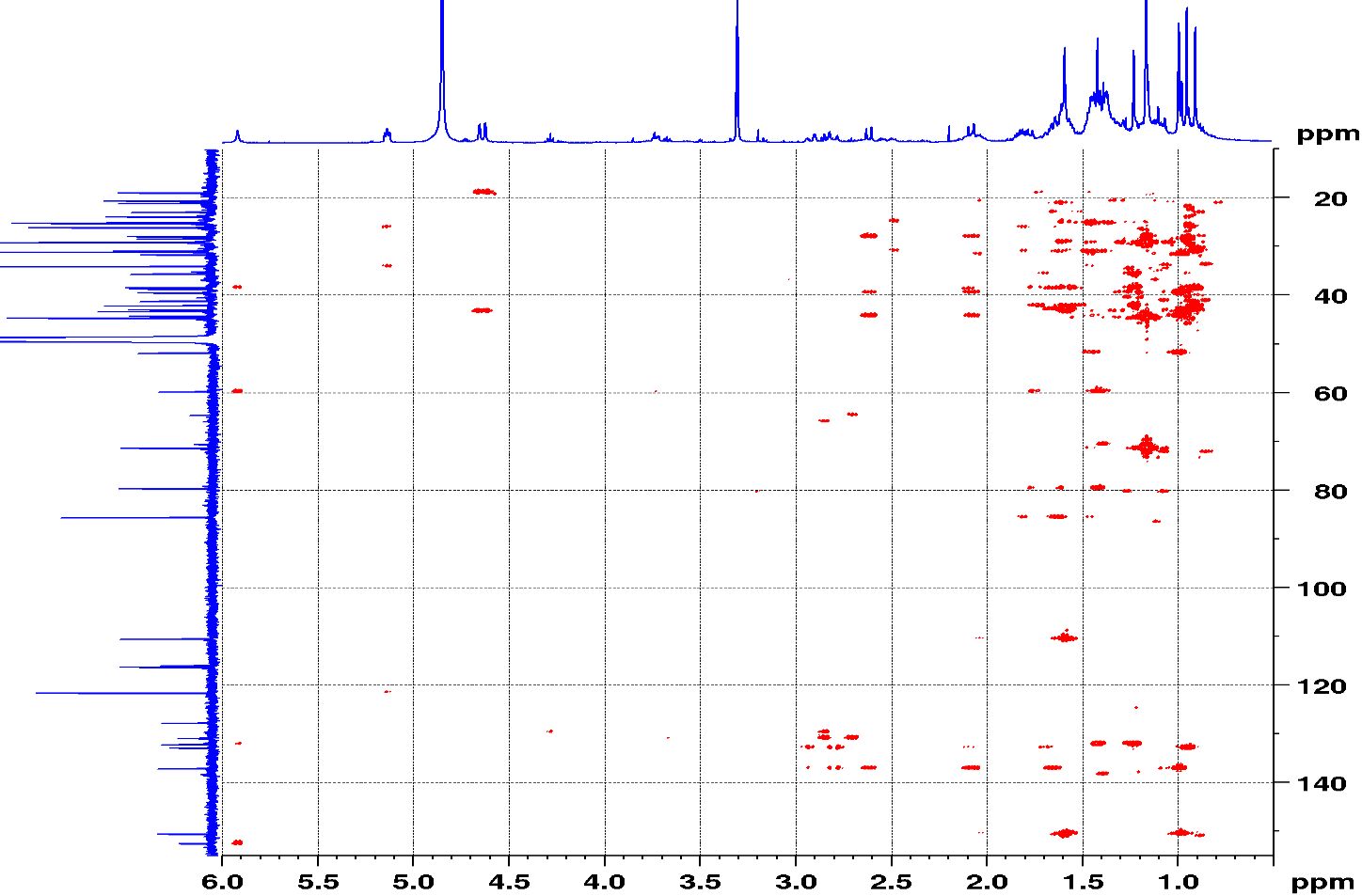
**

## Figure S50. ^1^H-^13^C HMBC spectrum (500 MHz) of Gromomycin H (9) in CD_3_OD.


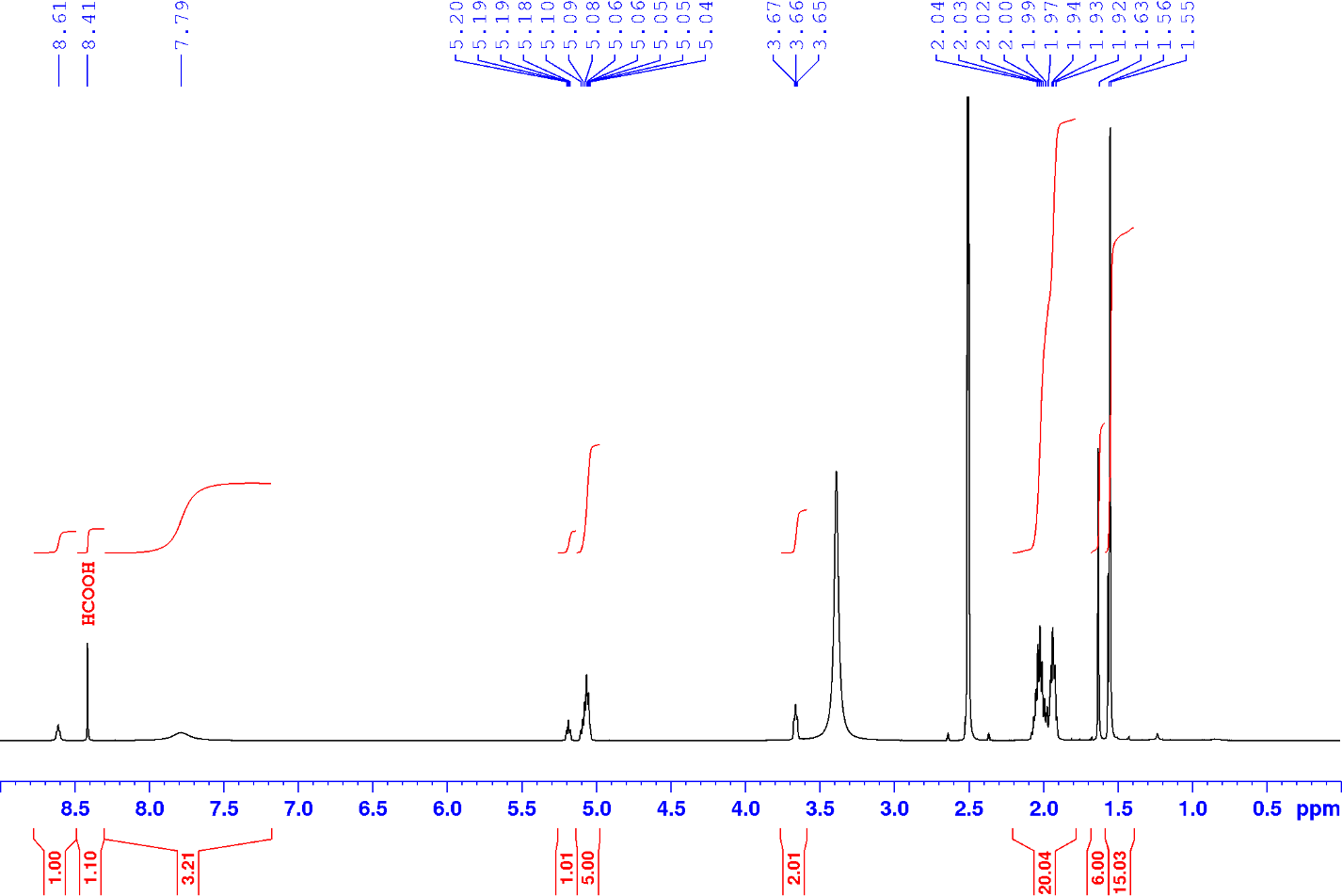


## Figure S51. ^1^H NMR spectrum (500 MHz) of Hexaprenylguanidine (10) in DMSO-d_6._

**
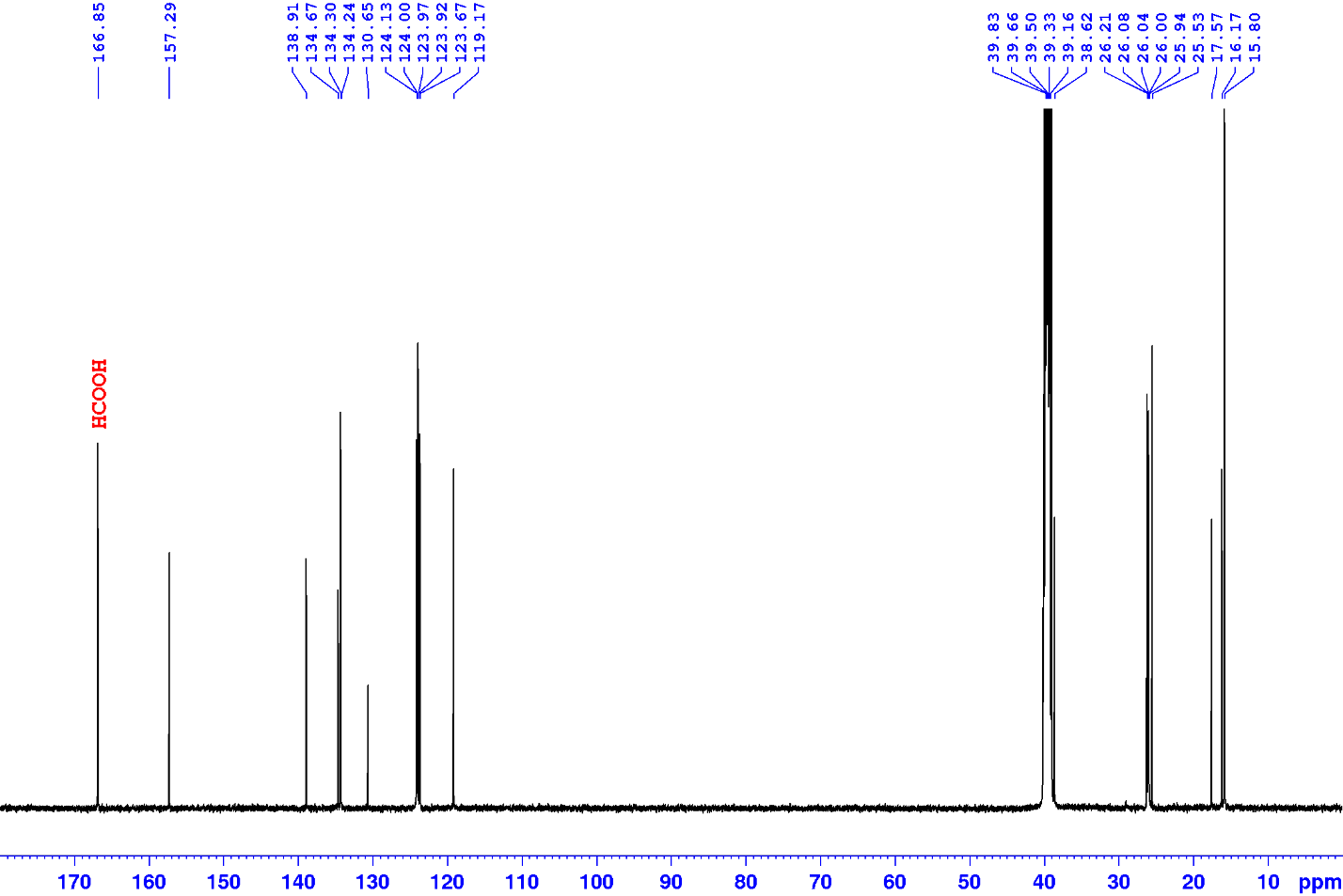
**

## Figure S52. ^13^C NMR spectrum (125 MHz) of Hexaprenylguanidine (10) in DMSO-d_6._


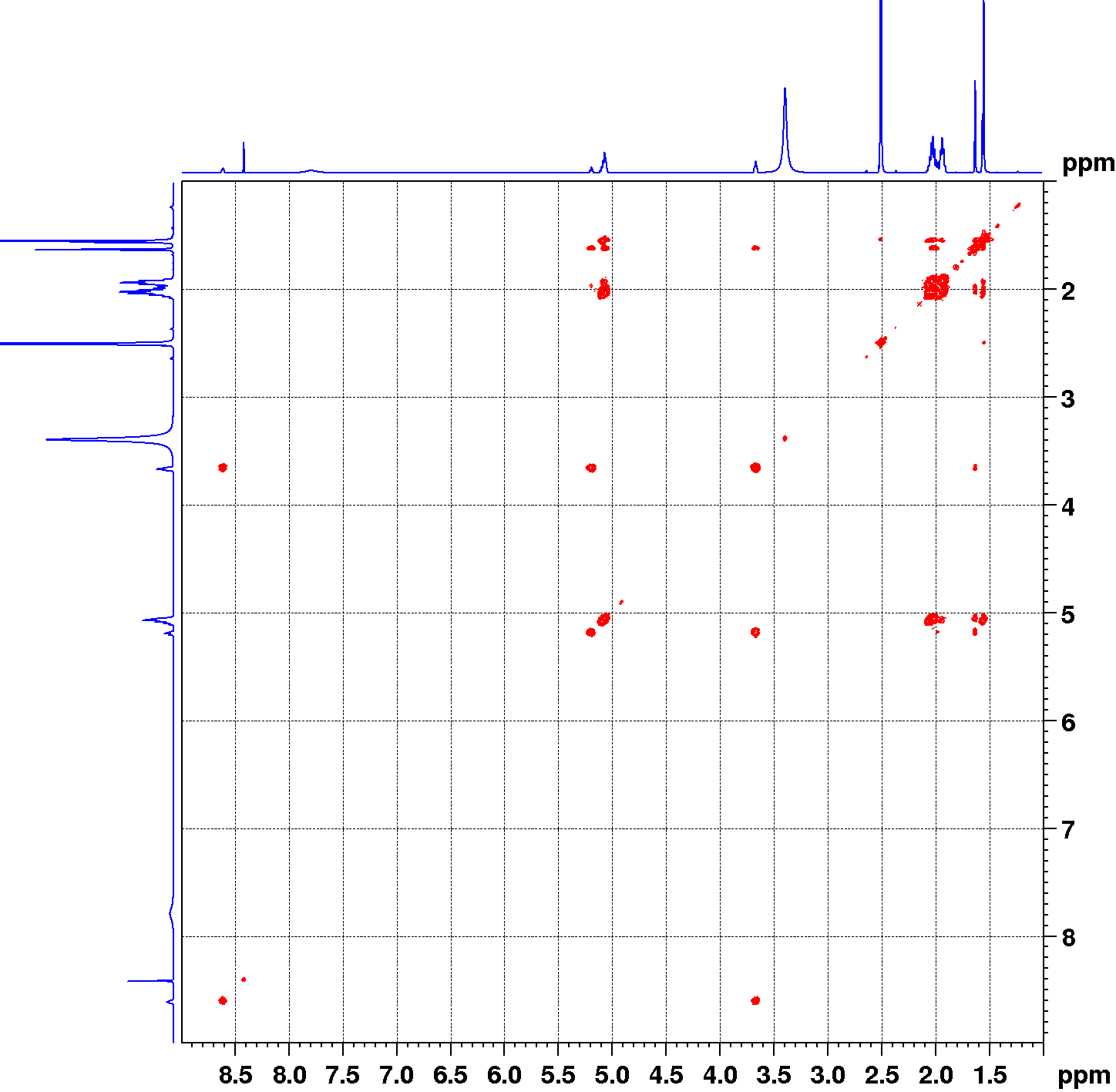


## Figure S53. COSY spectrum (500 MHz) of Hexaprenylguanidine (10) in DMSO-d_6._


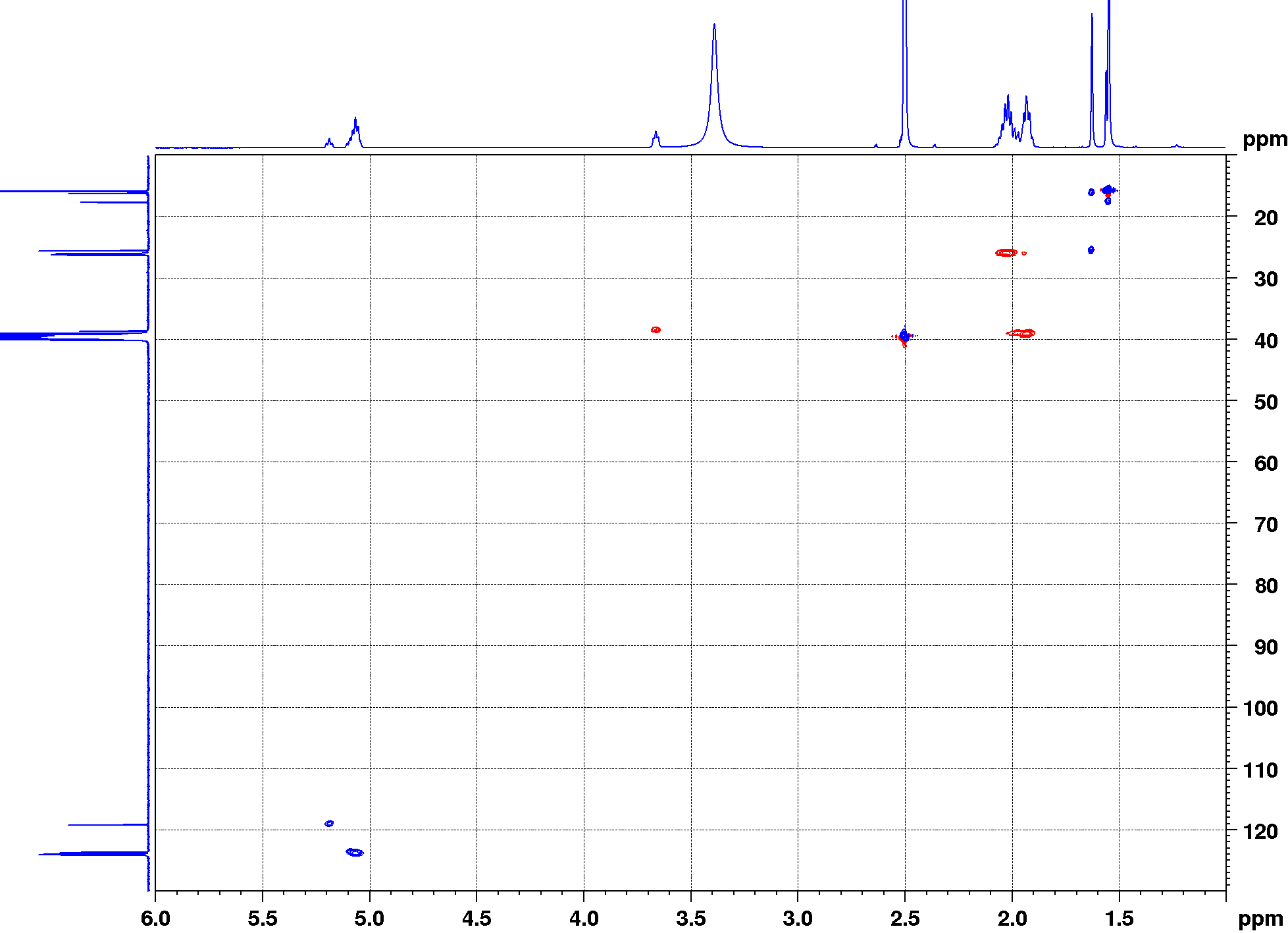


##
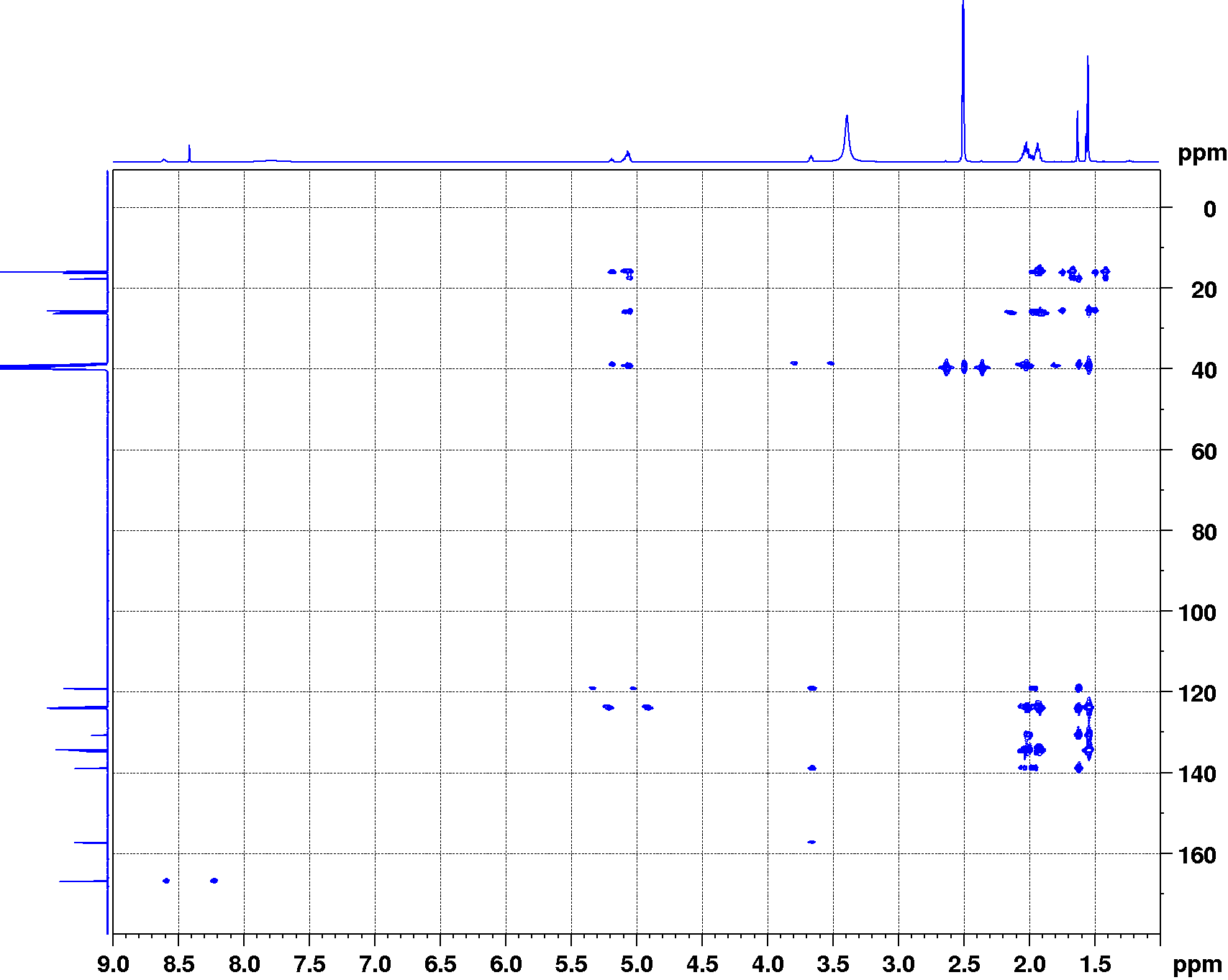
Figure S54. ^1^H-^13^C HSQC spectrum (500 MHz) of Hexaprenylguanidine (10) in DMSO-d_6._

## Table S55. ^1^H-^13^C HMBC spectrum (500 MHz) of Hexaprenylguanidine (10) in DMSO-d_6._


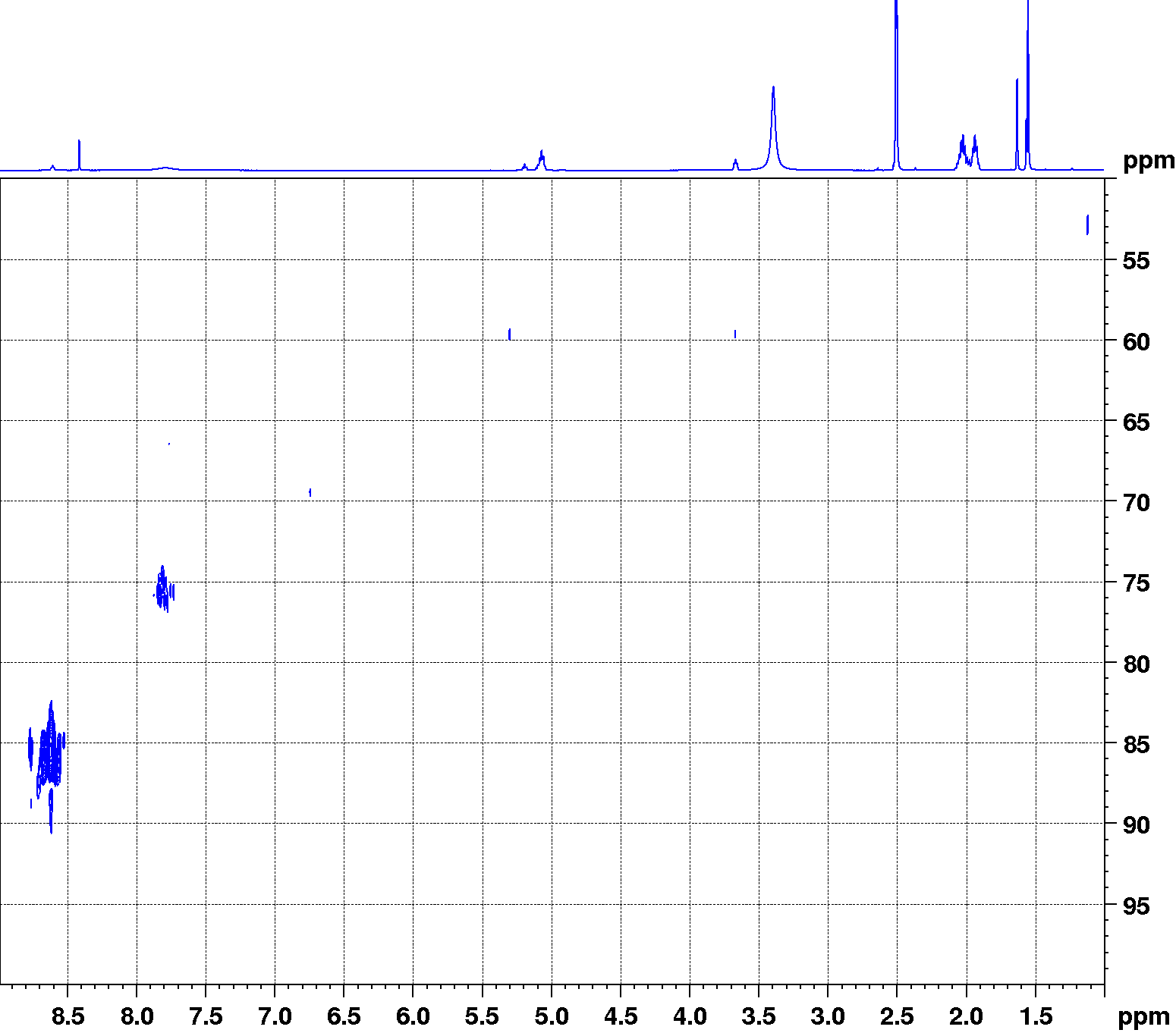


## Table S56. ^1^H-^15^N HSQC spectrum (500 MHz) of Hexaprenylguanidine (10) in DMSO-d_6._

**
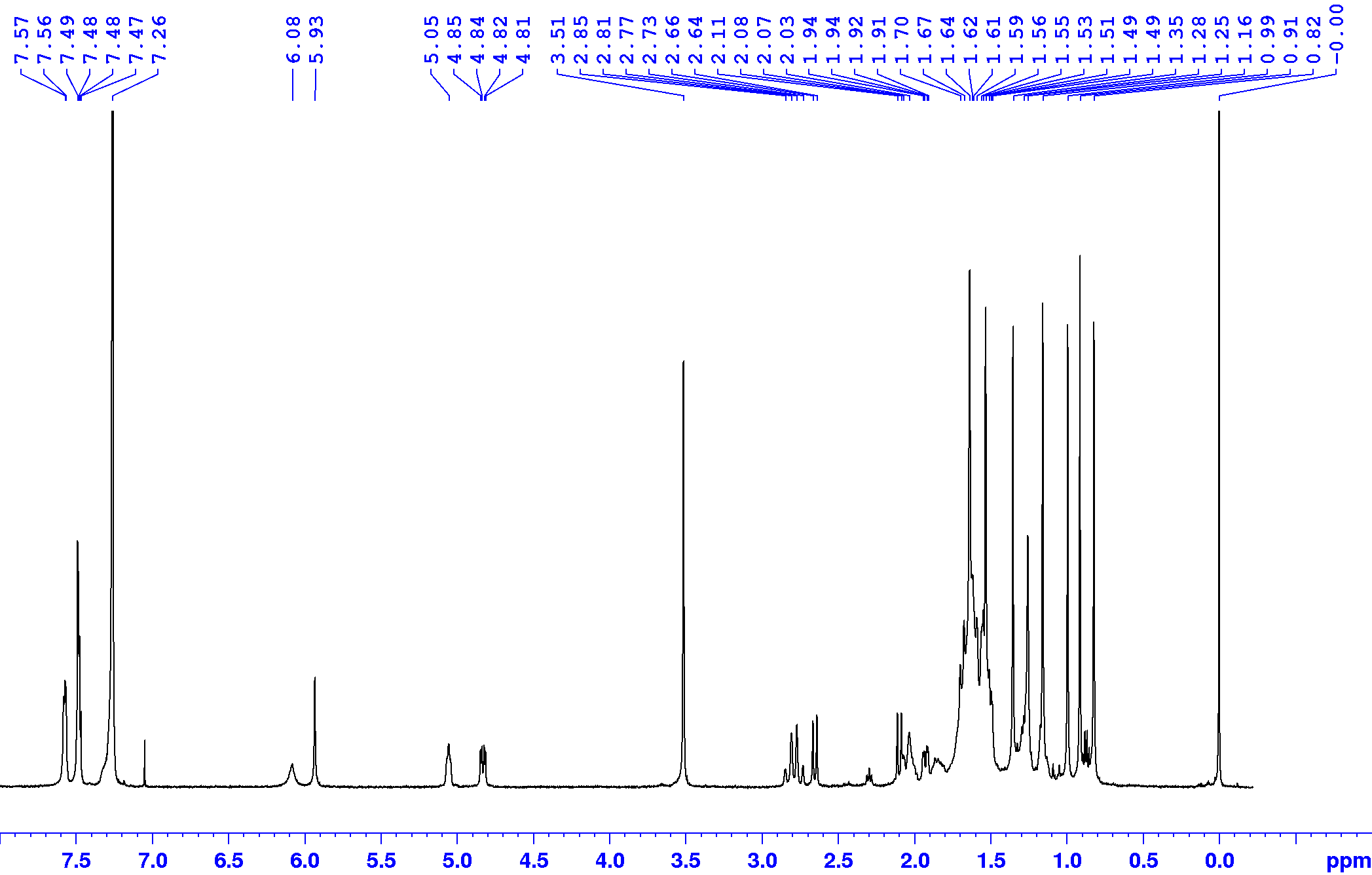
**

## Figure S57. ^1^H NMR spectrum (500 MHz) of Gromomycin D (*R*)-MTPA ester (5a) in CDCl_3._

**
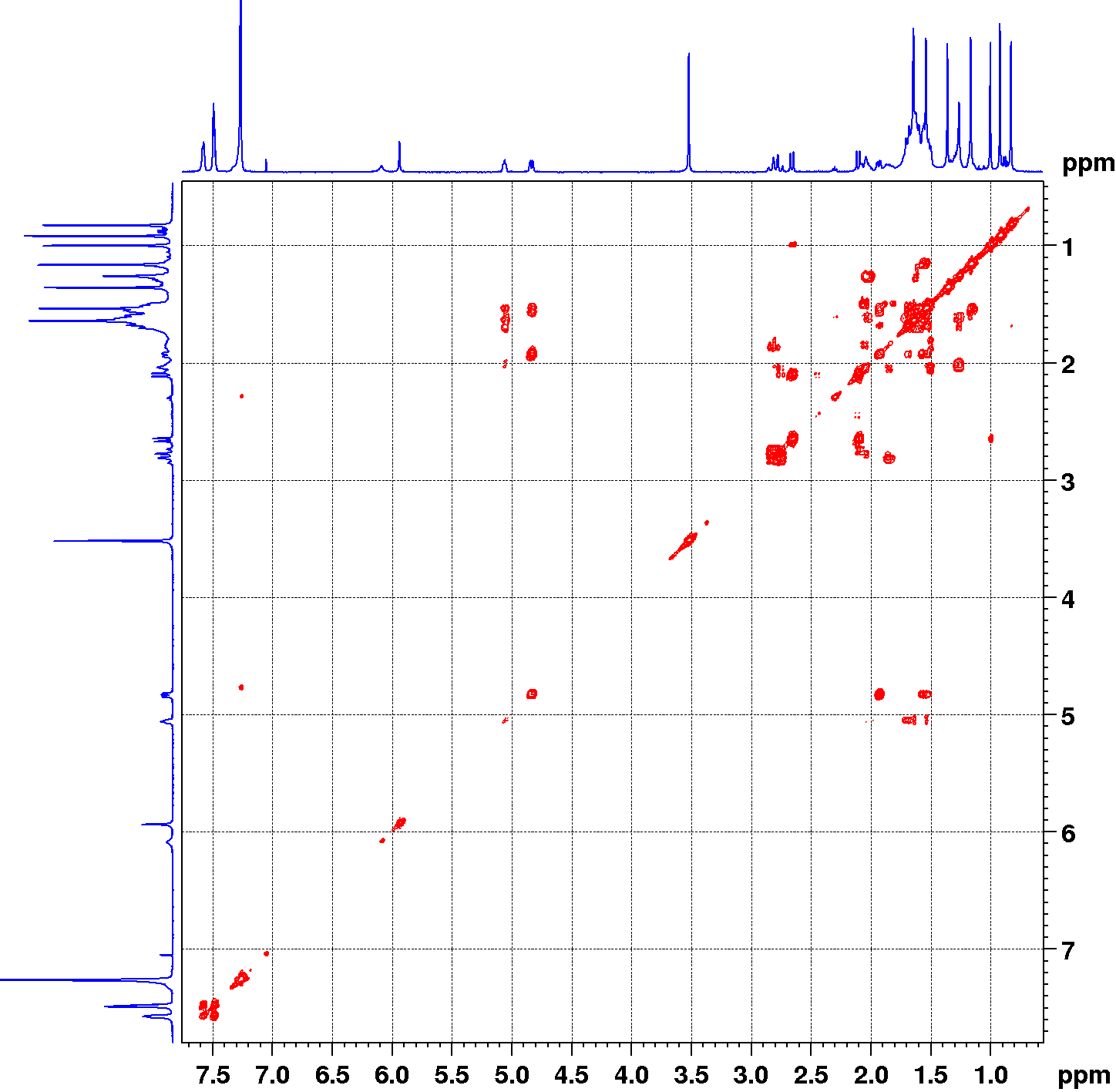
**

## Figure S58. COSY spectrum (500 MHz) of Gromomycin D (*R*)-MTPA ester (5a) in CDCl_3._

**
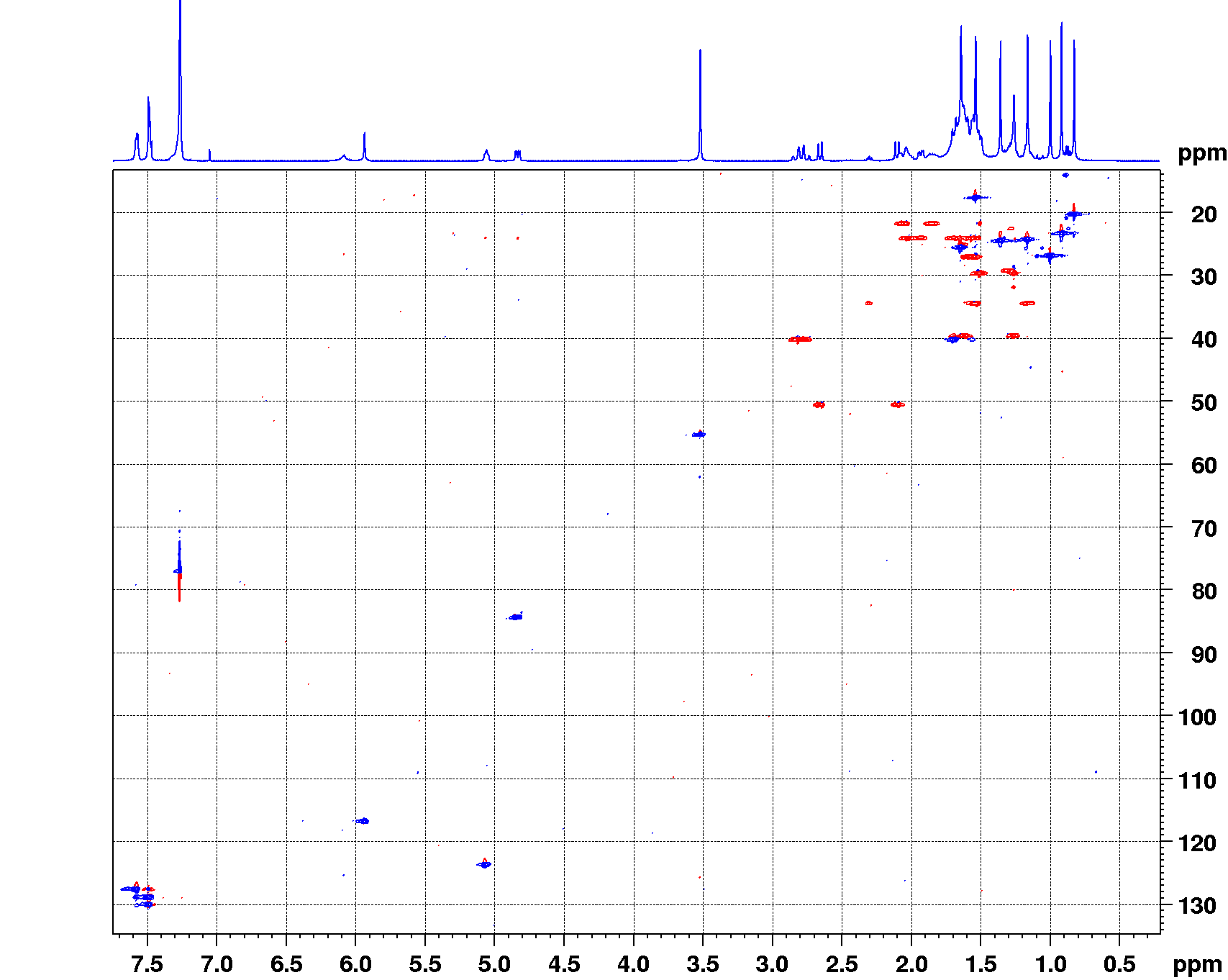
**

## Figure S59. ^1^H-^13^C HSQC spectrum (500 MHz) of Gromomycin D (*R*)-MTPA ester (5a) in CDCl_3._

**
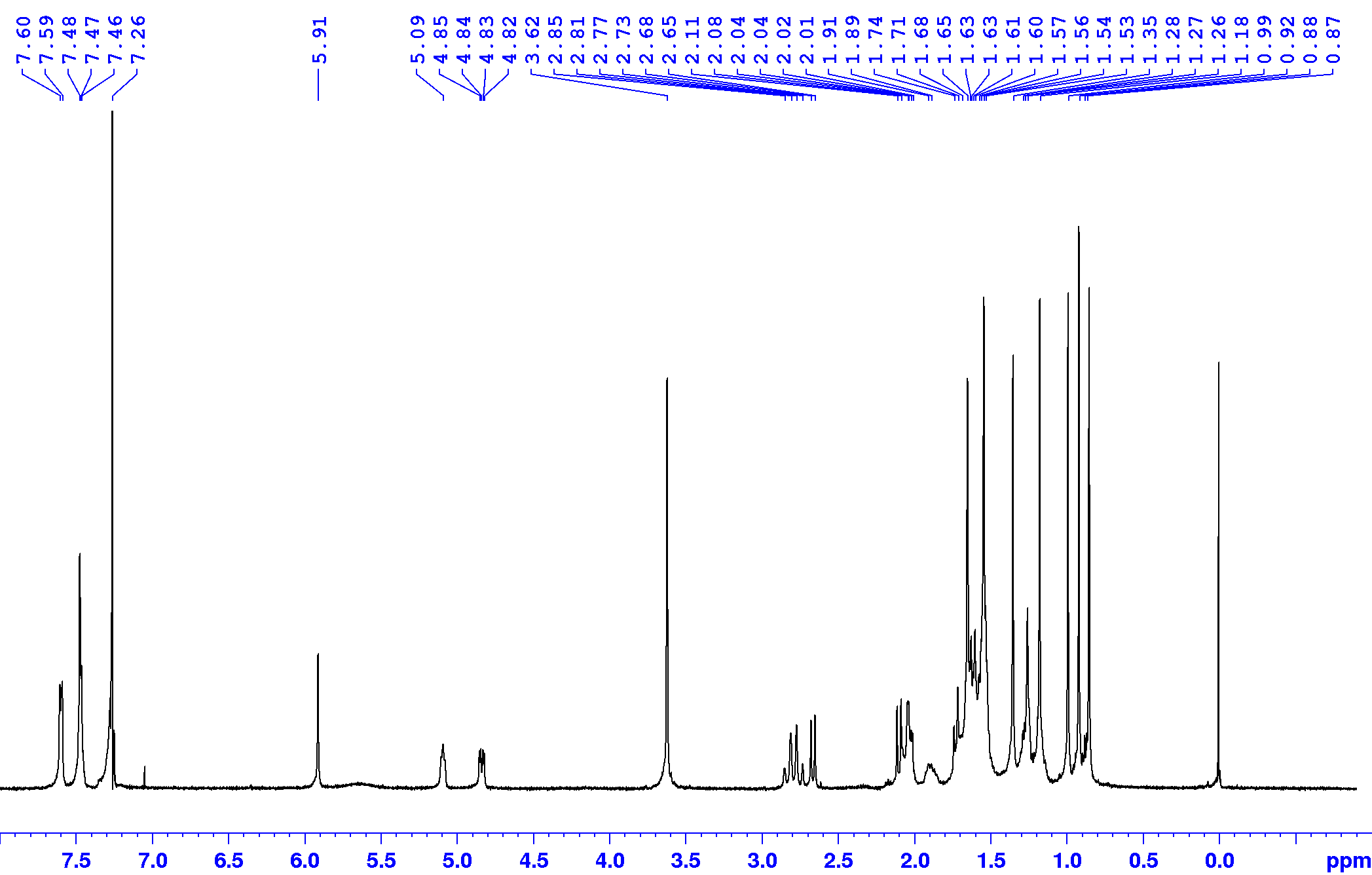
**

## Figure S60. ^1^H NMR spectrum (500 MHz) of Gromomycin D (*S*)-MTPA ester (5b) in CDCl_3._

**
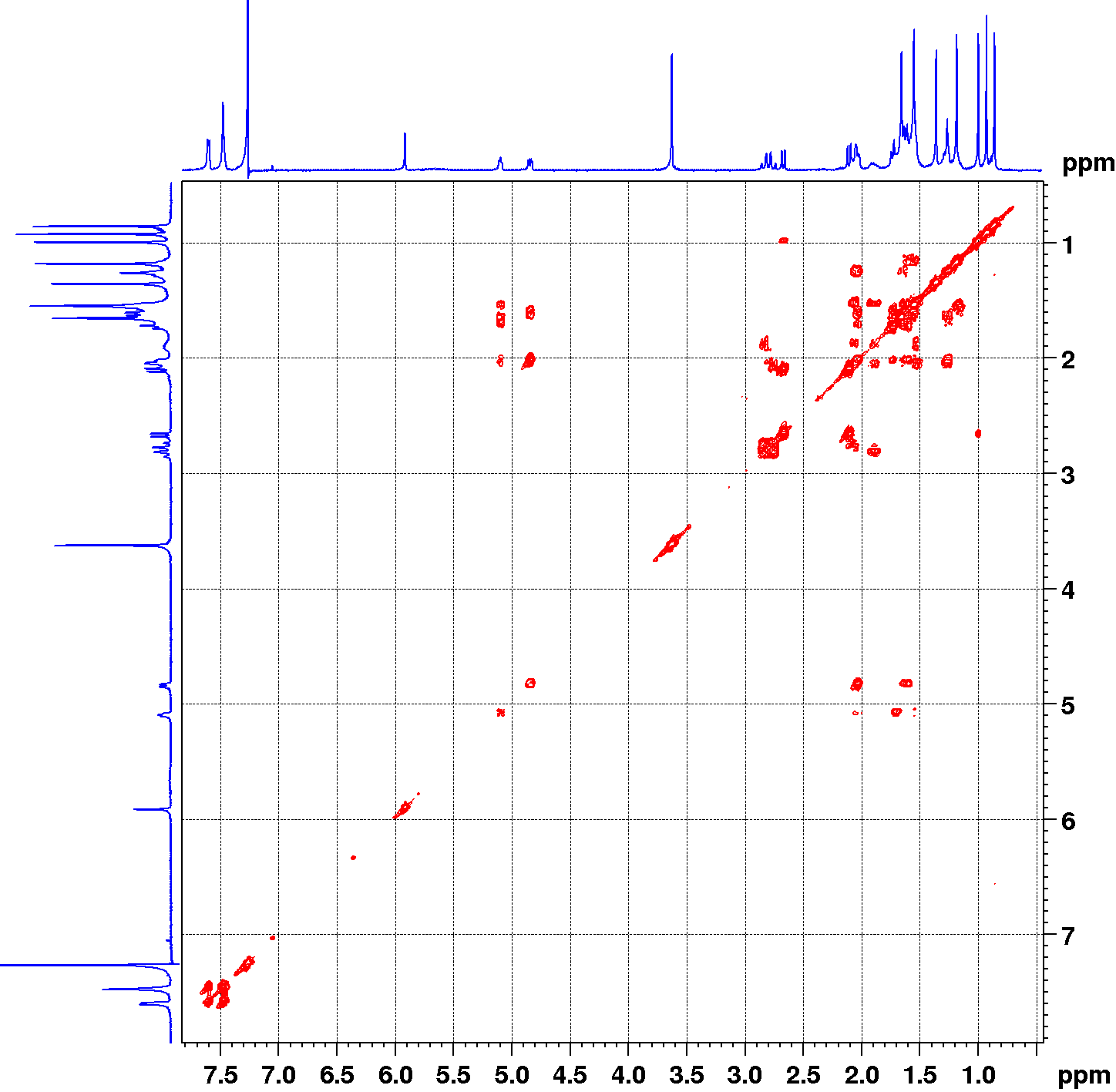
**

## Figure S61. COSY spectrum (500 MHz) of Gromomycin D (*S*)-MTPA ester (5b) in CDCl_3._

**
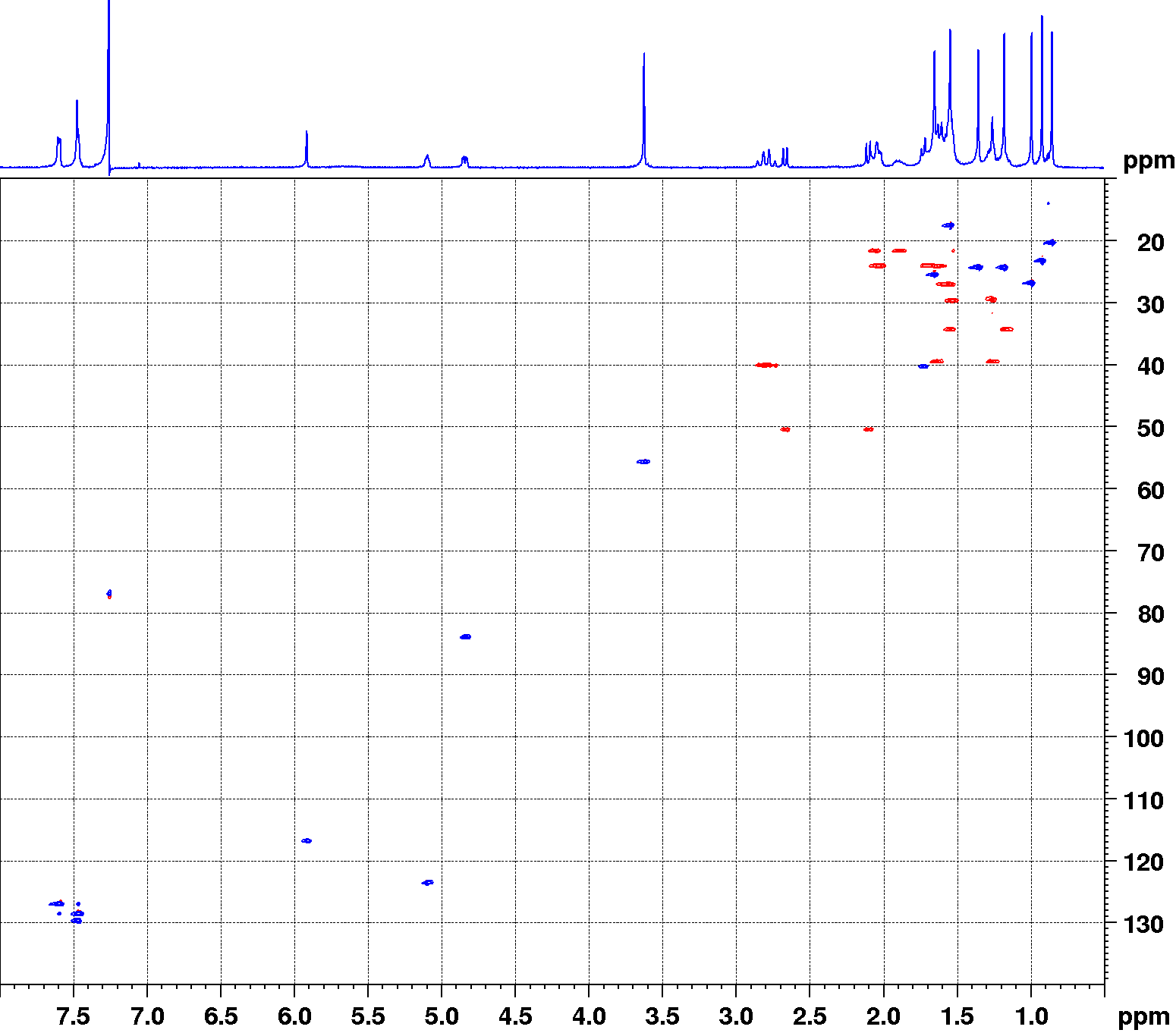
**

## Figure S62. ^1^H-^13^C HSQC spectrum (500 MHz) of Gromomycin D (*S*)-MTPA ester (5b) in CDCl3.

_l_

_
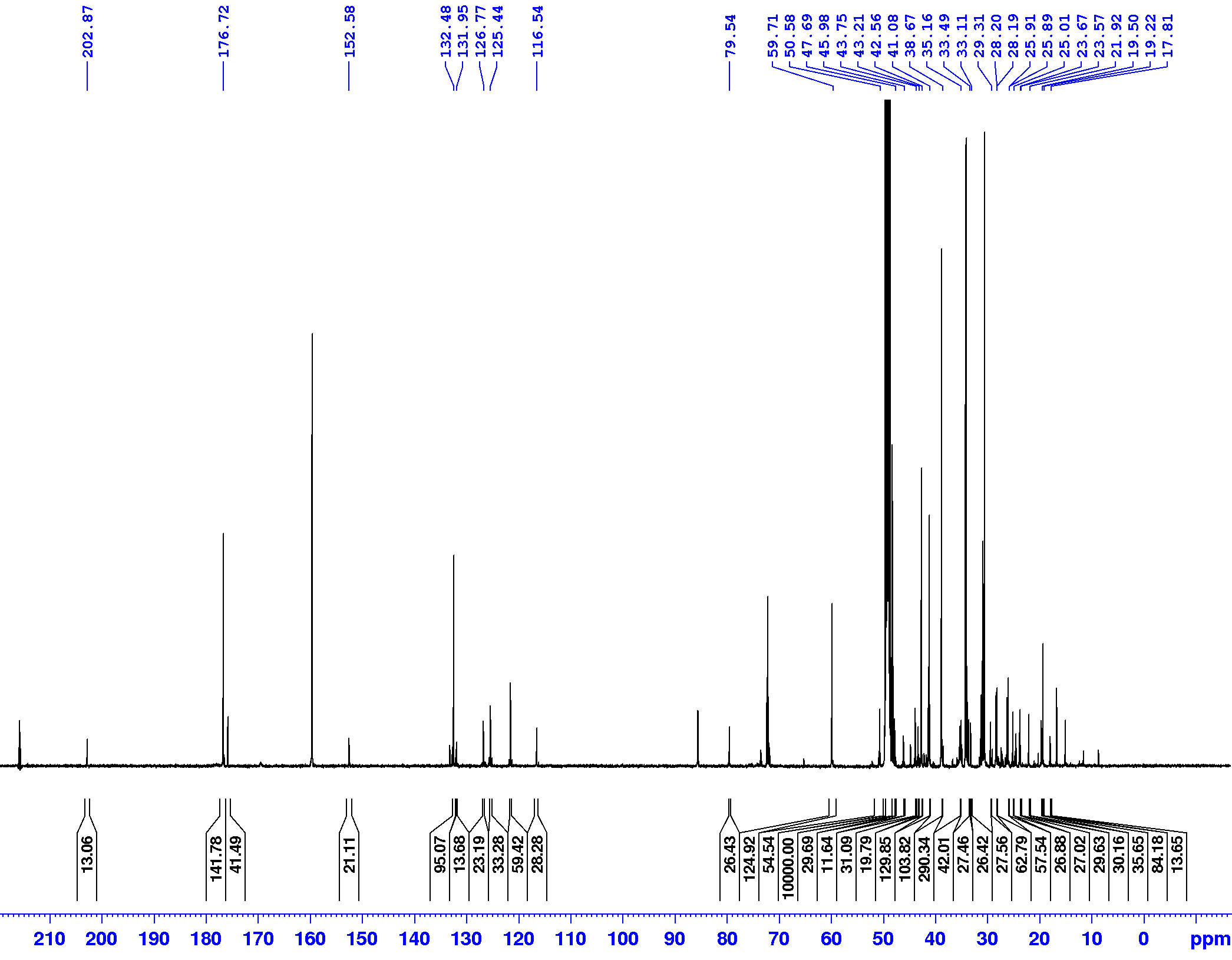
_

## Figure S63. ^13^C NMR spectrum (125 MHz) of Gromomycin B (3) in CD_3_OD from feeding experiment with [2-^13^C] pyruvate.


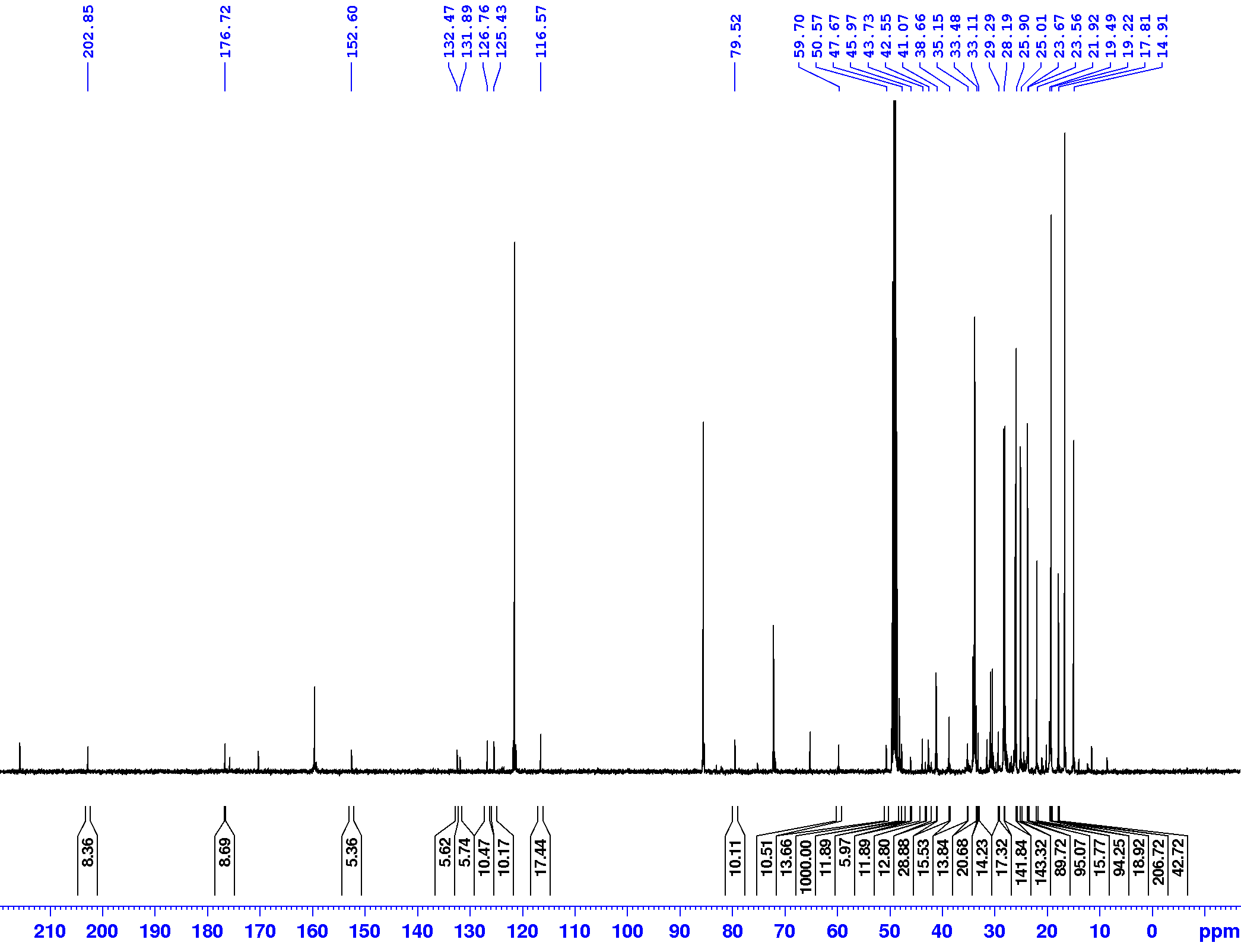


## Figure S64. ^13^C NMR spectrum (125 MHz) of Gromomycin B (3) in CD_3_OD from feeding experiment with [3-^13^C] pyruvate.


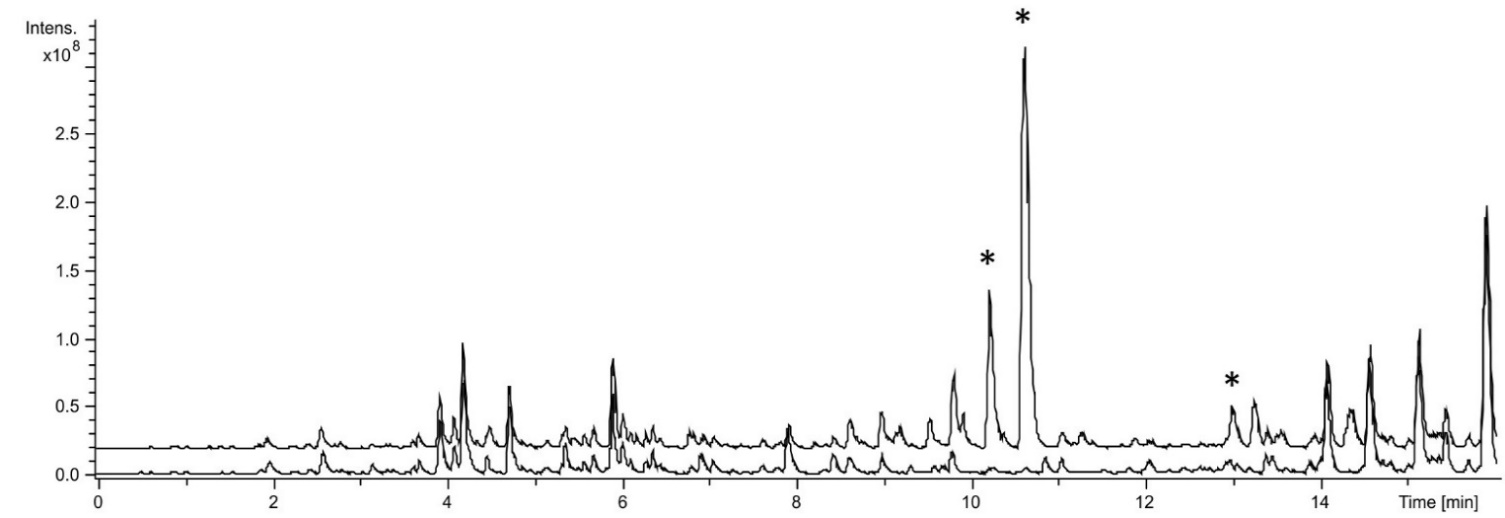


## Figure S65. Heterologous expression of the cosmid P04_E01 with gromomycin cluster into *S. albus* Del14. Peaks induced by gromomycin are indicated by asterisks.


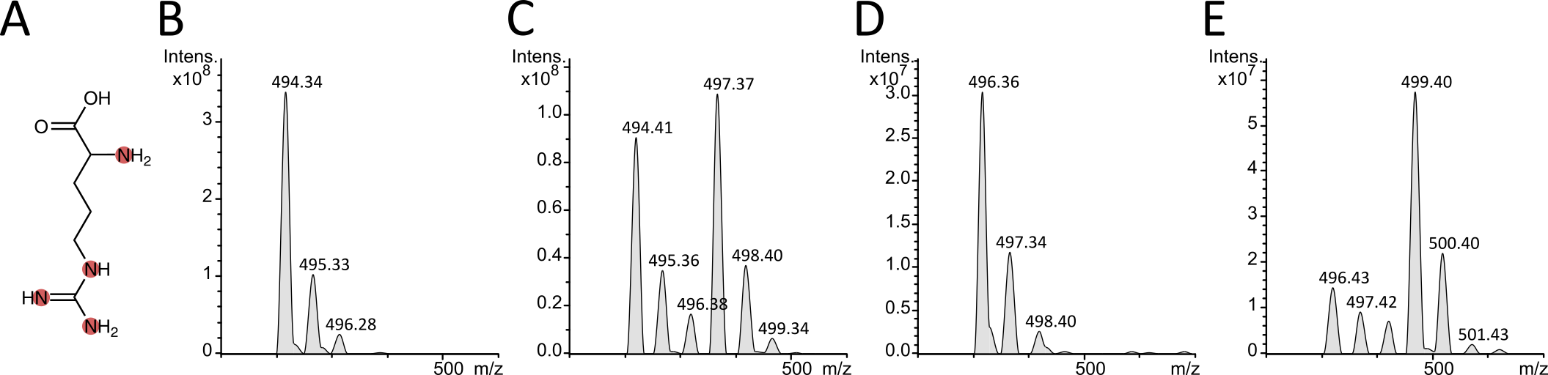


Figure S66. Incorporation of labelled L-arginine into gromomycins. A, Structure of labeled arginine (the labeled nitrogen atoms is indicated by the red circle). B, C, D, E Mass peaks of gromomycins without (B, D) and with (C, E) feeding of labelled L-arginine.


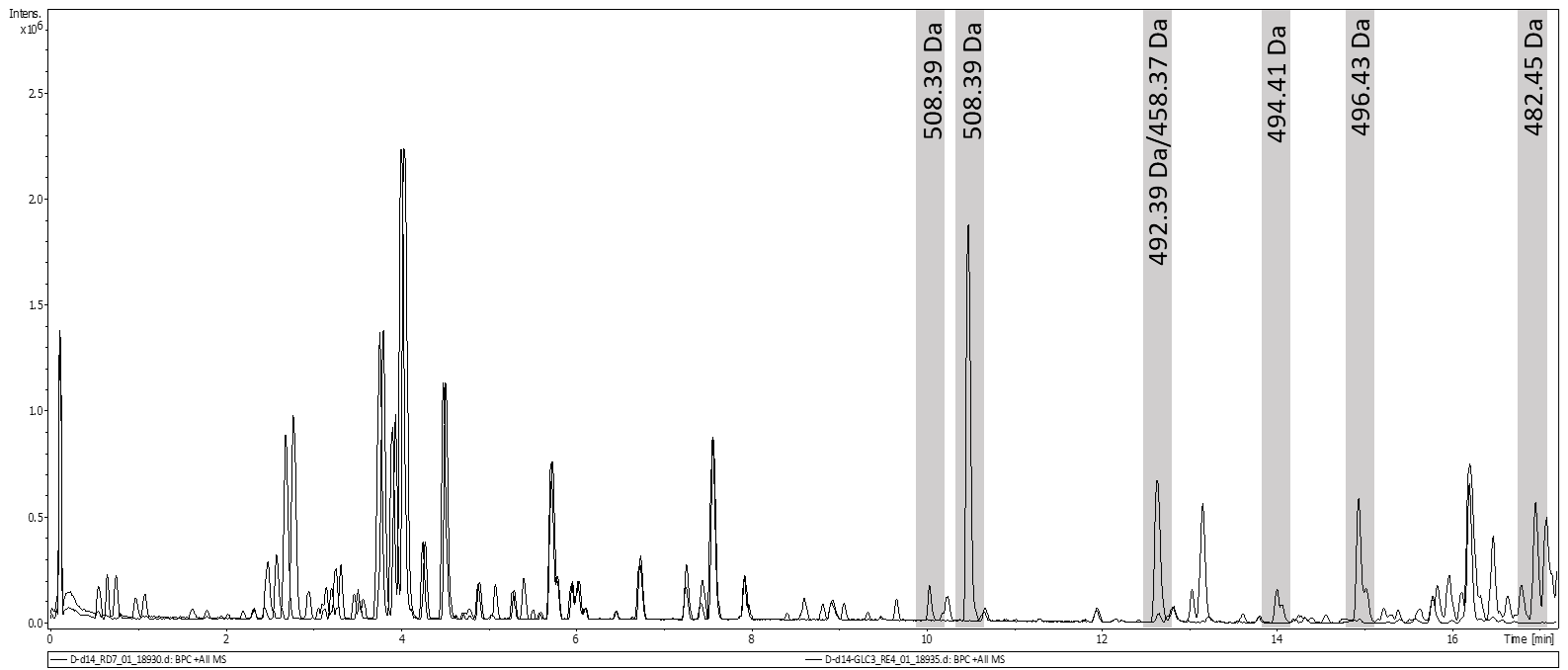


Figure S67. Heterologous expression of the P03_G02 cosmid with gromomycin-like cluster into *S. albus* Del14 strain. New methylated gromomycins are indicated by grey rectangles.


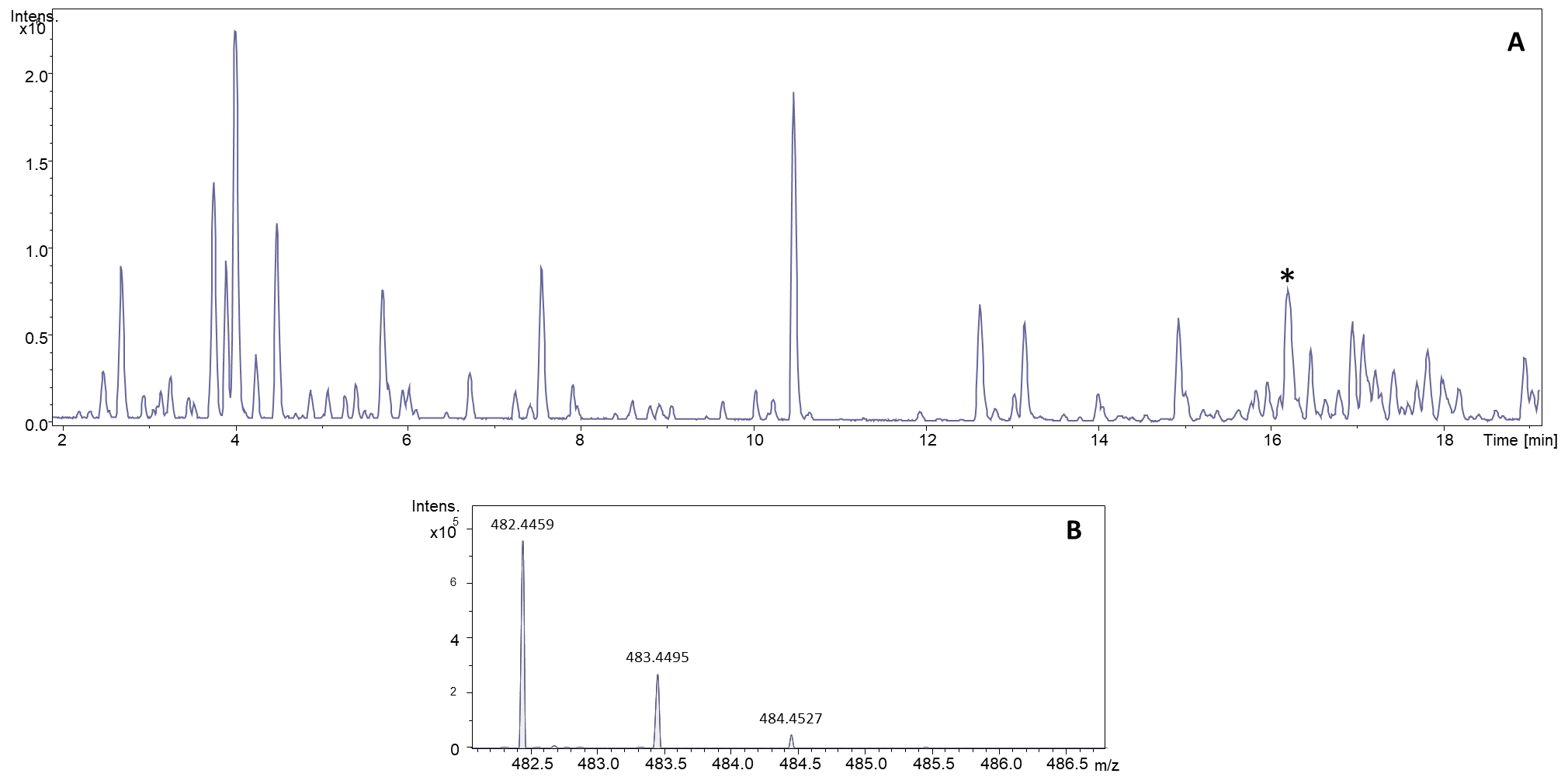


Figure S68. Linear methylated hexaprenylguanidine (marked with asterisks). A, chromatogram of extract from *S. albus Del14* with gromomycin-like cluster from *Streptomyces flavoviridis*; B, mass range of linear methylated hexaprenylguanidine.


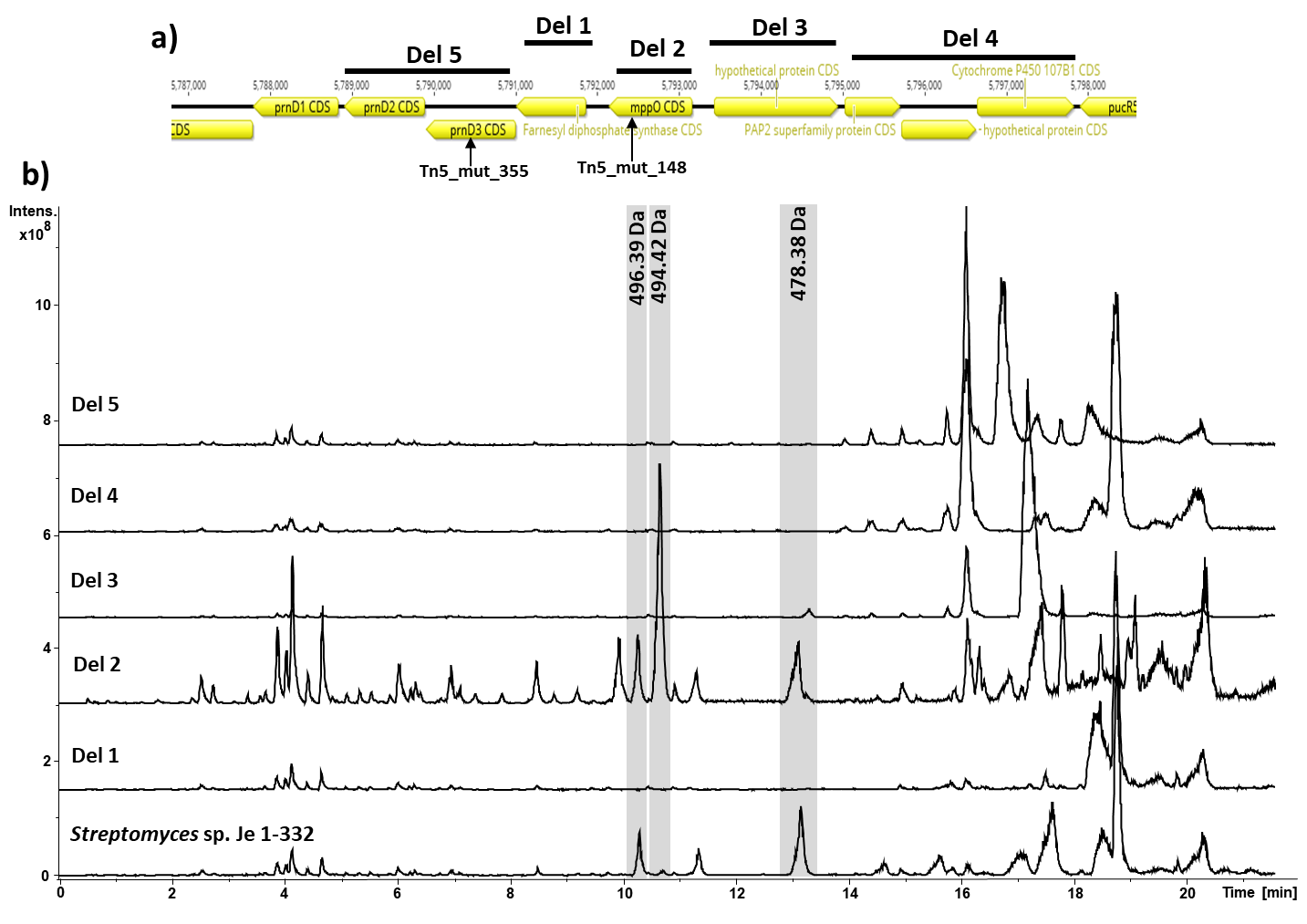


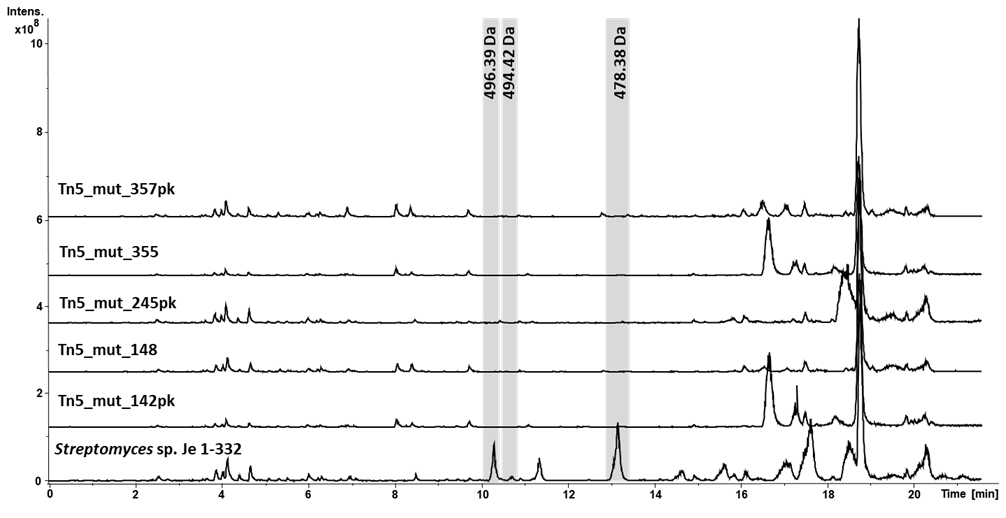


Figure S69. Deletion into *Streptomyces sp.* Je 1-332 strain. a) Deletion scheme into chromosome. b) HPLC-MS chromatograms.


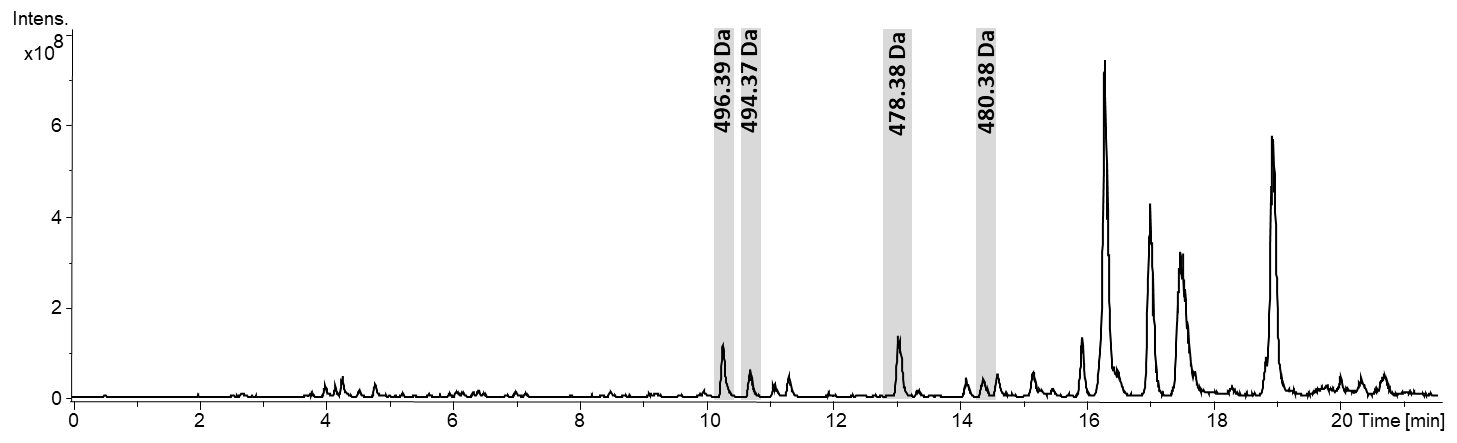


## Figure S70. HPLC-MS chromatogram of crude extract of *Streptomyces sp.* Je 1-332 strain.


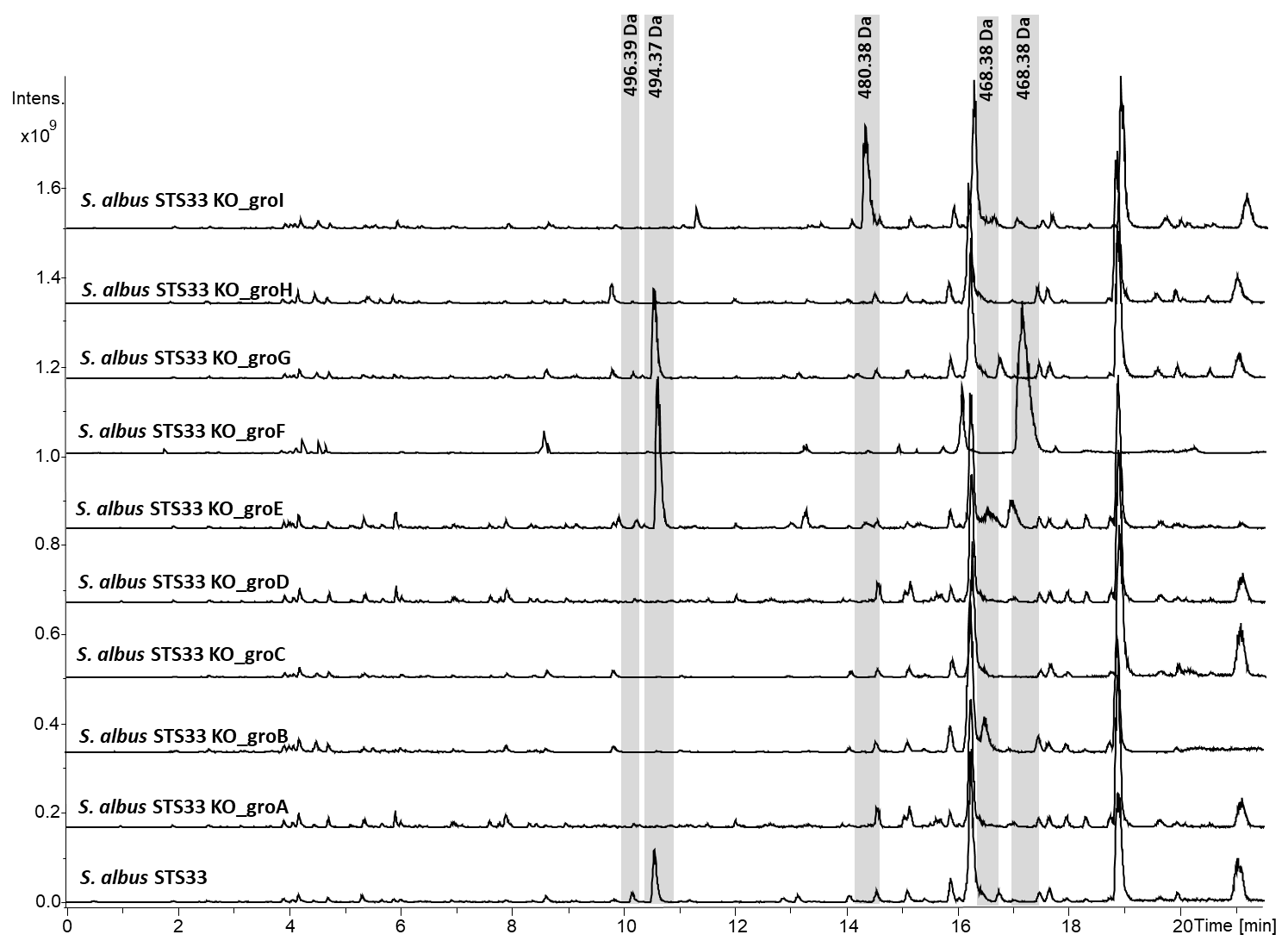


## Figure S71. HPLC-MS chromatograms of the *S. albus* with the *gro*-gene deletion.


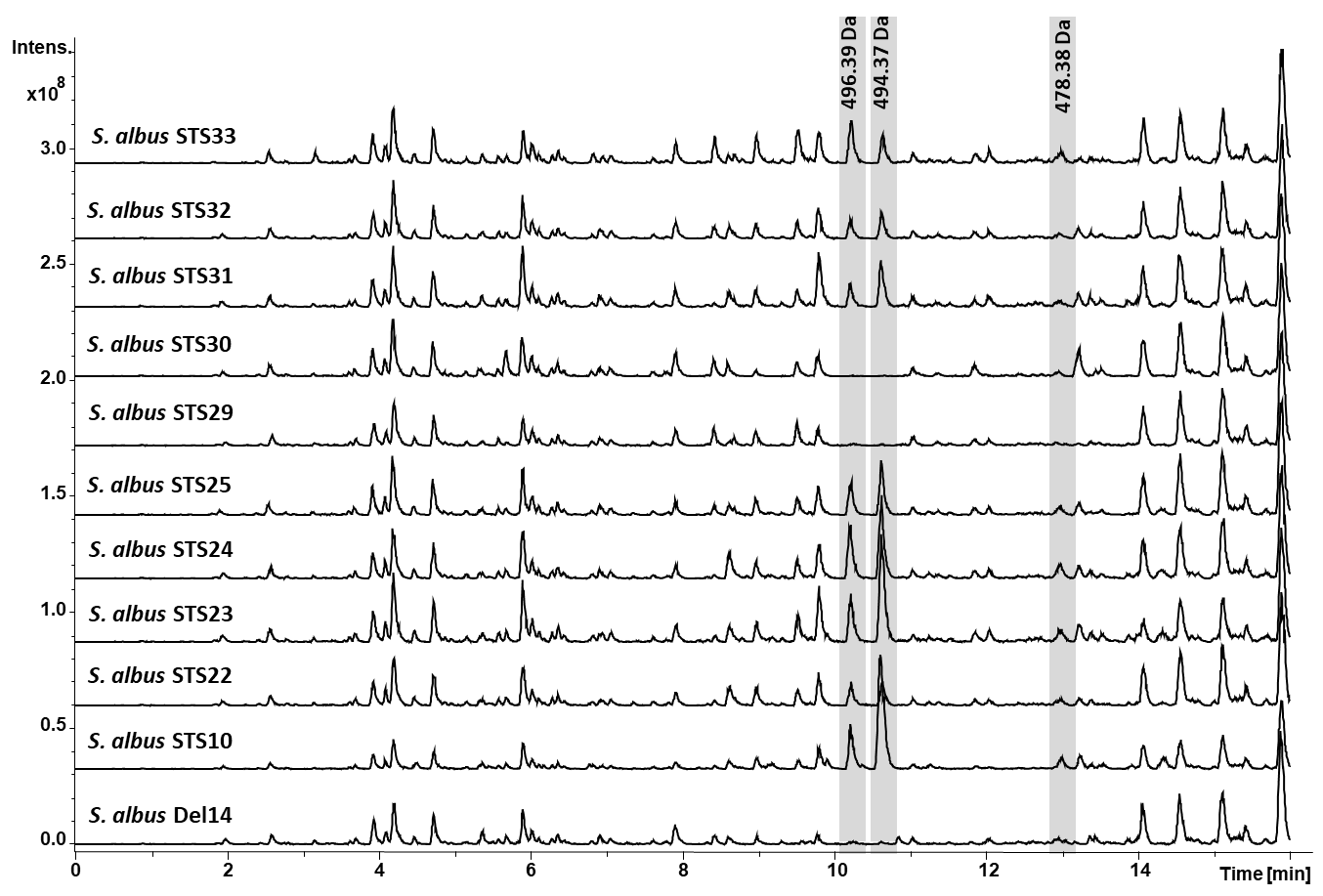
Figure S72. HPLC-MS chromatograms of the deletion mutants which are shown in Figure 4.


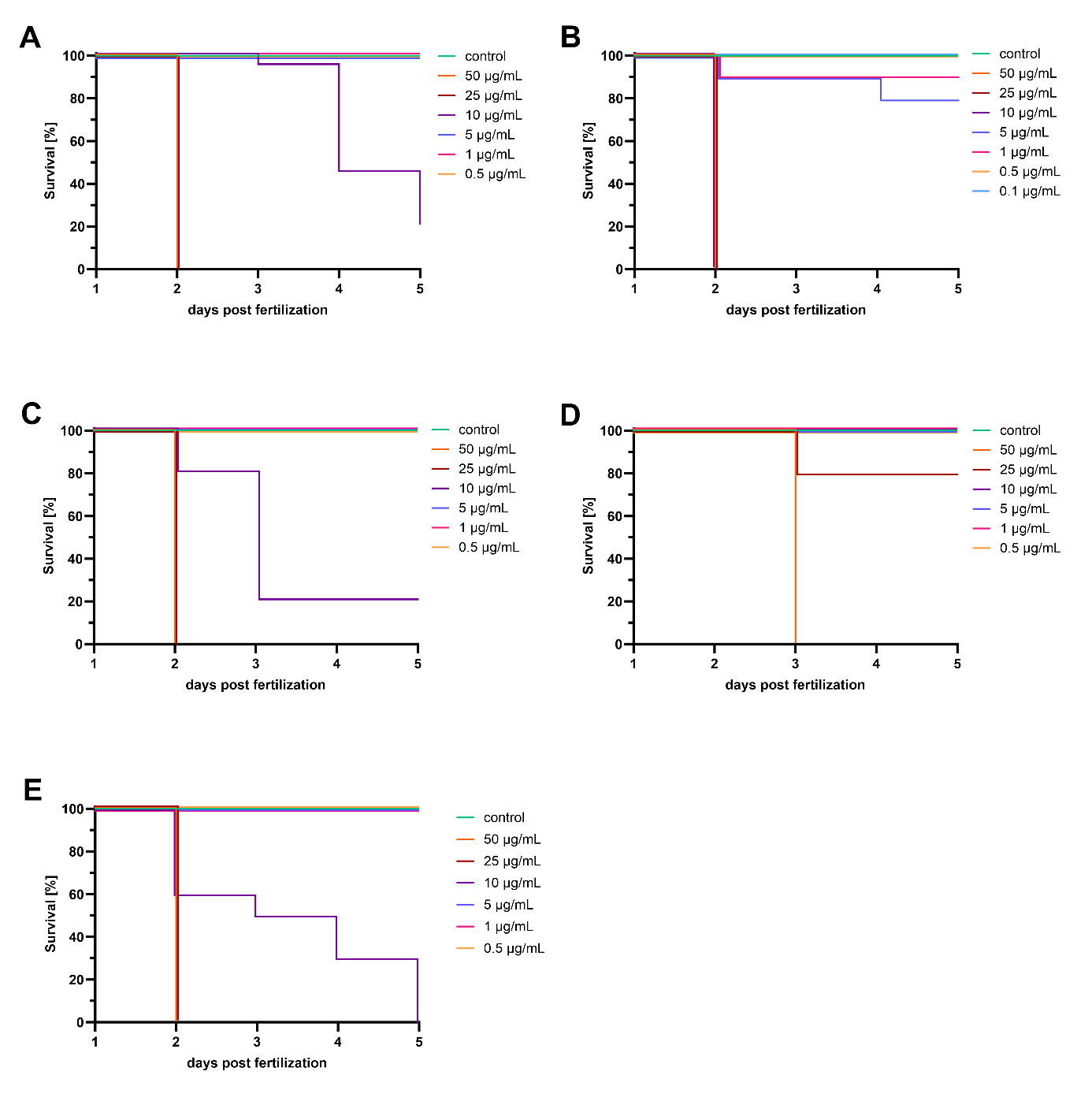


Figure S73. Kaplan-Meier curves of zebrafish embryos exposed to different concentrations of gromomycin B (A), gromomycin A (B), gromomycin F (C), gromomycin G (D) and gromomycin E (E). Zebrafish embryos (n = 10) were treated with a series of concentrations of different gromomycin derivatives (aquatic exposure) and were monitored daily until 5 days post fertilization (dpf). Embryos exposed to the solvent (1% DMSO in 0.3x Danieau’s) served as control. Kaplan-Meier curves were generated using GraphPad Prism.


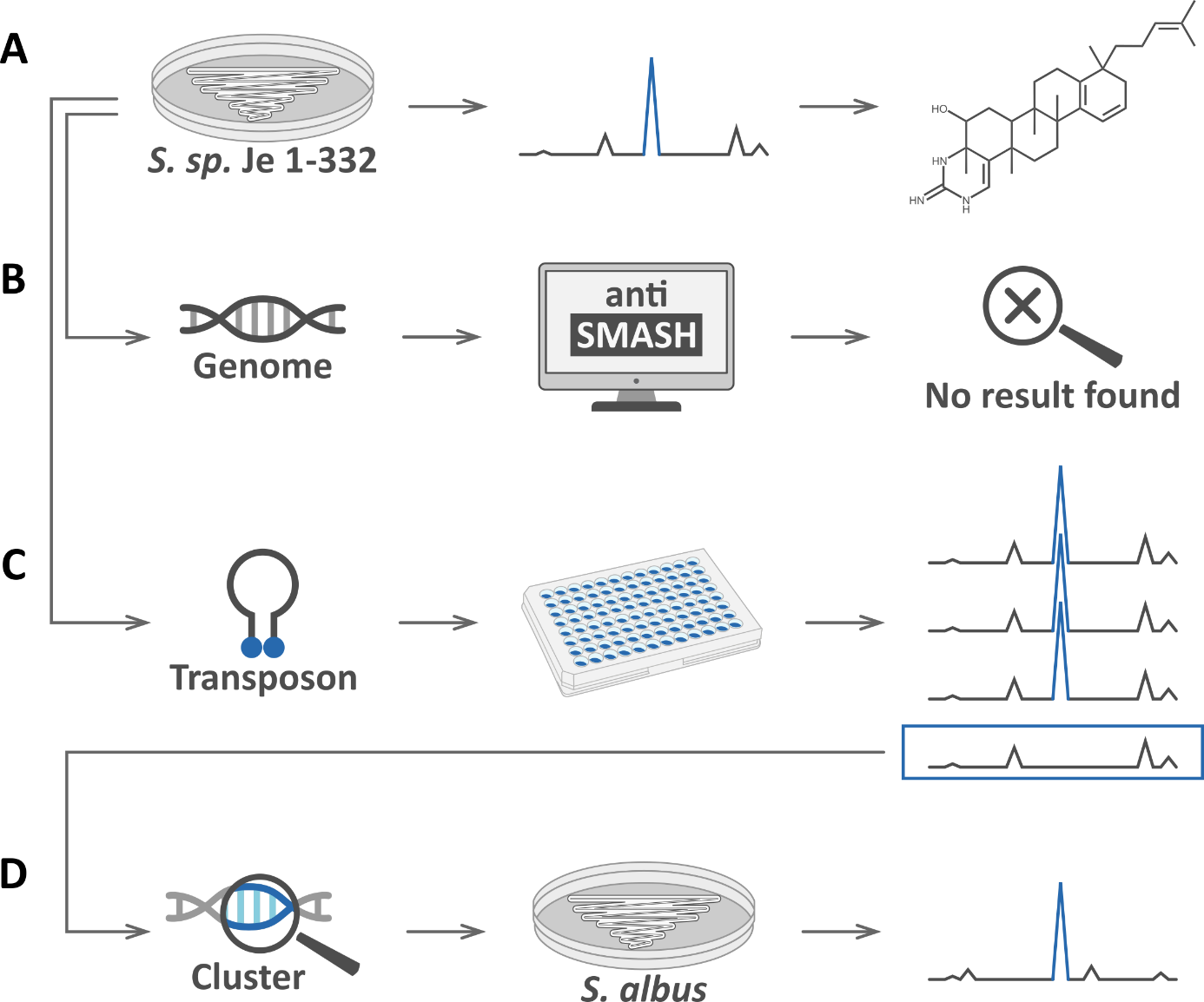


Figure S74. Scheme for identifying gene cluster involved in gromomycin biosynthesis. A, Chemical screening of crude extract. B, C, Identification of biosynthetic gene clusters using bioinformatics tools (B) and transposon mutagenesis (C). D, Validation and heterologous expression of the identified gene cluster.

References

[1] S. Tistechok, I. Roman, V. Fedorenko, A. Luzhetskyy, O. Gromyko, *Folia Microbiologica* **2023**, *68*, 645-653.

[2] M. Myronovskyi, B. Rosenkränzer, S. Nadmid, P. Pujic, P. Normand, A. Luzhetskyy, *Metabolic Engineering* **2018**, *49*, 316-324.

[3] Y. Ahmed, Y. Rebets, M. R. Estévez, J. Zapp, M. Myronovskyi, A. Luzhetskyy, *Microbial Cell Factories* **2020**, *19*, 1-16.

[4] T. Kieser, M. J. Bibb, M. J. Buttner, K. F. Chater, D. A. Hopwood, *Practical Streptomyces Genetics, Vol. 291*, John Innes Foundation Norwich, **2000**.

[5] A. Bankevich, S. Nurk, D. Antipov, A. A. Gurevich, M. Dvorkin, A. S. Kulikov, V. M. Lesin, S. I. Nikolenko, S. Pham, A. D. Prjibelski, *Journal of Computational Biology* **2012**, *19*, 455-477.

[6] F. Meyer, A. Goesmann, A. C. McHardy, D. Bartels, T. Bekel, J. Clausen, J. Kalinowski, B. Linke, O. Rupp, R. Giegerich, *Nucleic Acids Research* **2003**, *31*, 2187-2195.

[7] T. Seemann, *Bioinformatics* **2014**, *30*, 2068-2069.

[8] K. Blin, S. Shaw, A. M. Kloosterman, Z. Charlop-Powers, G. P. Van Wezel, M. H. Medema, T. Weber, *Nucleic Acids Research* **2021**, *49*, W29-W35.

[9] L. Petzke, A. Luzhetskyy, *Applied Microbiology and Biotechnology* **2009**, *83*, 979-986.

[10] M. Steinegger, J. Söding, *Nature Biotechnology* **2017**, *35*, 1026-1028.

[11] A. Müller, F. Grein, A. Otto, K. Gries, D. Orlov, V. Zarubaev, M. Girard, X. Sher, O. Shamova, T. Roemer, *International Journal of Medical Microbiology* **2018**, *308*, 335-348.

[12] R. Müller, C. Fu, Y. Liu, C. Walt, S. Rasheed, C. Bader, P. Lukat, M. Neuber, J. Haeckl, W. Blankenfeldt, **2023**.

[13] U. Strähle, S. Scholz, R. Geisler, P. Greiner, H. Hollert, S. Rastegar, A. Schumacher, I. Selderslaghs, C. Weiss, H. Witters, *Reproductive Toxicology* **2012**, *33*, 128-132.

[14] T. R. Hoye, C. S. Jeffrey, F. Shao, *Nature Protocols* **2007**, *2*, 2451-2458.

[15] G. Flesch, M. Rohmer, *European Journal of Biochemistry* **1988**, *175*, 405-411.

[16] F. Flett, V. Mersinias, C. P. Smith, *FEMS Microbiology Letters* **1997**, *155*, 223-229.

[17] J. Fu, M. Teucher, K. Anastassiadis, W. Skarnes, A. F. Stewart, in *Methods in Enzymology, Vol. 477*, Elsevier, **2010**, pp. 125-144.

[18] M. Myronovskyi, B. Rosenkränzer, A. Luzhetskyy, *Applied Microbiology and Biotechnology* **2014**, *98*, 4557-4570.
